# *Trichomonas vaginalis *vast BspA-like gene family: evidence for functional diversity from structural organisation and transcriptomics

**DOI:** 10.1186/1471-2164-11-99

**Published:** 2010-02-08

**Authors:** Christophe J Noël, Nicia Diaz, Thomas Sicheritz-Ponten, Lucie Safarikova, Jan Tachezy, Petrus Tang, Pier-Luigi Fiori, Robert P Hirt

**Affiliations:** 1 Institute for Cell and Molecular Biosciences, Newcastle University, Newcastle upon Tyne, NE2 4HH, UK; 2 Department of Biomedical Sciences, Division of Experimental and Clinical Microbiology, University of Sassari, Italy, 07100 Sassari, Italy; 3 Center for Biological Sequence Analysis, Department of Systems Biology BioCentrum-DTU, Technical University of Denmark, DK-2800 Lyngby, Denmark; 4 Department of Parasitology, Charles University, Vinicna 7, 128 44 Prague 2, Czech Republic; 5 Bioinformatics Center/Molecular Medicine Research Center, Chang Gung University, Taoyuan 333, Taiwan

## Abstract

**Background:**

*Trichomonas vaginalis *is the most common non-viral human sexually transmitted pathogen and importantly, contributes to facilitating the spread of HIV. Yet very little is known about its surface and secreted proteins mediating interactions with, and permitting the invasion and colonisation of, the host mucosa. Initial annotations of *T. vaginalis *genome identified a plethora of candidate extracellular proteins.

**Results:**

Data mining of the *T. vaginalis *genome identified 911 BspA-like entries (TvBspA) sharing TpLRR-like leucine-rich repeats, which represent the largest gene family encoding potential extracellular proteins for the pathogen. A broad range of microorganisms encoding BspA-like proteins was identified and these are mainly known to live on mucosal surfaces, among these *T. vaginalis *is endowed with the largest gene family. Over 190 TvBspA proteins with inferred transmembrane domains were characterised by a considerable structural diversity between their TpLRR and other types of repetitive sequences and two subfamilies possessed distinct classic sorting signal motifs for endocytosis. One TvBspA subfamily also shared a glycine-rich protein domain with proteins from *Clostridium difficile *pathogenic strains and *C. difficile *phages. Consistent with the hypothesis that TvBspA protein structural diversity implies diverse roles, we demonstrated for several TvBspA genes differential expression at the transcript level in different growth conditions. Identified variants of repetitive segments between several TvBspA paralogues and orthologues from two clinical isolates were also consistent with TpLRR and other repetitive sequences to be functionally important. For one TvBspA protein cell surface expression and antibody responses by both female and male *T. vaginalis *infected patients were also demonstrated.

**Conclusions:**

The biased mucosal habitat for microbial species encoding BspA-like proteins, the characterisation of a vast structural diversity for the TvBspA proteins, differential expression of a subset of TvBspA genes and the cellular localisation and immunological data for one TvBspA; all point to the importance of the TvBspA proteins to various aspects of *T. vaginalis *pathobiology at the host-pathogen interface.

## Background

*Trichomonas vaginalis *is a flagellated protist responsible for the most prevalent non-viral sexually transmitted infection (STI), with an annual estimate of 174 millions new infections worldwide [1], corresponding to at least the combined estimates of *Chlamydia trachomatis *and *Neisseria gonorea *infections, and which has, paradoxically, attracted so far relatively little attention from health agencies worldwide [2,3]. The parasite is capable of causing severe vaginal, ectocervical, prostatic and urethral inflammations, and is linked with sterility, pelvic inflammatory disease, adverse pregnancy outcomes, postnatal complications and cervical cancers [4-7]. Furthermore *T. vaginalis *also contributes, along with other STI, to the HIV pandemic by boosting the efficiency of virus transmission through several possible mechanisms including induction of inflammatory response resulting in neutrophils and macrophages recruitment into urogenital mucosa, compromising the mucosal barrier through microhaemoragia, increasing viral load in urogenital mucosa secretions and as a carrier (a Trojan horse) of infective HIV particles [6,8,9]. Hence, *T. vaginalis *is capable of invading and colonising the heavily defended host urogenital mucosa from both sexes, braking through the primary innate defences and withstanding induced innate and adaptive responses, about which little is known in relation to *T. vaginalis *infections [10]. Notably, *T. vaginalis *infections are often considered non-self limiting in females and recent data even suggest that persistent, undetected infections can persist even after successful treatments [11].

The pathobiology of *T. vaginalis *is complex and multifaceted with adhesion to, and alterations of, the various mucosal landmarks (mucus, epithelial cell barrier, extracellular matrix [ECM], innate and adaptive immune cells, bacterial microflora) thought to be essential to initiate and maintain infections [4,12,13]. *T. vaginalis *cells are also known to form large cell aggregates (in a process called swarming or rosetting), which could represent an important process for pathogenesis [14], suggesting that specific cell-cell interactions also take place between cells of the parasite. When the mucosal tissue is damaged the parasite can bind to host ECM proteins [15] and during menstruation or parasite induced microhaemoragia, *T. vaginalis *also binds to various plasma proteins [4]. Adhesion to host tissue also induces a cellular differentiation of *T. vaginalis *into amoeboid forms [16,17]. Furthermore the parasite endocytoses host proteins (e.g. lactoferrin and laminin) [4,18], as well as various human viruses [9,19], and phagocytoses the autochthonous mucosal microflora and various host cells [20,21], including spermatozoids [22]; key cellular processes for nutrient uptake thought to dependent on specific surface proteins. However, little is known about the molecular and cellular basis of these various processes, with the pathogen lipophosphoglycan (LPG), various adhesions, surface and secreted enzymes and toxins all thought to be involved, but existing data are limited when not controversial [12,13,15,23-27]. A so far unique human receptor for *T. vaginalis*, galectin-1, was only recently identified when investigating the role of *T. vaginalis *LPG in binding to ectocervical epithelial cell lines [24].

An initial gene survey of expressed sequence tags (EST) identified *T. vaginalis *cDNA encoding 65 distinct proteins we named BspA-like (TvBspA) [28], due to their similarity with the BspA protein from *Tannerella forsythensis *[29], and led us to further characterise *in silico *one complete open reading frame (ORF) encoding a potential surface protein TvBspA625 [28]. TvBspA proteins are characterised by a specific type of leucine-rich repeats (LRR), named TpLRR after a membrane protein from *Treponema pallidium *[30], shared with *T. forsythensis *BspA and *Treponema denticola *LrrA proteins [31]. This discovery was particularly appealing for *T. vaginalis *because the BspA and LrrA proteins were shown to be expressed on the bacteria cell surface and to be involved in the colonisation of the oral mucosa; BspA binds to ECM protein fibronectin and to the clotting factor fibrinogen and both BspA and LrrA stimulate co-aggregation between the two bacteria, and promote their adhesion to and invasion of epithelial cells [31-34]. Furthermore, the TpLRR of the *T. forsythensis *BspA protein was shown to trigger an innate immune response by inducing IL-8 secretion in epithelial cells via toll-like receptor 2 (TLR2) and TLR1 signalling [35]. The BspA protein was also shown to elicit a strong antibody responses in *T. forsythensis *infected patients [29]. Hence, TvBspA proteins could play similar roles and mediate important interactions with mucosal features including its microflora and host cells and proteins. The availability of one *T. vaginalis *genome sequence [36], ~70,000 EST from several different growth conditions and 75 distinct TvBspA genes spotted on microarrays gave us the opportunity to investigate the TvBspA genome complement and perform a first exploration of their corresponding transcripts to gain further insight into their potential importance in host-pathogen interactions. Here, we present an updated survey of genomes encoding BspA-like proteins and the first detailed bioinformatic characterisations of TvBspA genomic distribution and exceptional structural diversity. We demonstrated differential expression at the transcript level of selected TvBspA genes upon *T. vaginalis *binding to ECM proteins or exposed to different iron concentration; and TvBspA625 cell surface expression and host antibody response during infection. Together these data strongly indicate that TvBspA proteins are likely to play several important and distinct roles in *T. vaginalis *pathobiology and provide the fundamental data for future TvBspA genes and proteins comparison between various *T. vaginalis *clinical isolates and to initiate TvBspA proteins detailed functional characterisation.

## Results

### The *T. vaginalis *genome encodes an exceptionally large putative BspA-like protein family

A combination of different protein Blast searches identified a total of 911 distinct TvBspA candidate proteins in the *T. vaginalis *current genome annotation (Table 1; additional files 1, 2, 3, Table S1, S2, S3). Reciprocal BlastP searches with each individual candidate TvBspA confirmed their annotation as BspA-like proteins with top hits being TvBspA proteins (882 entries) or BspA-like proteins from other taxa (additional file 4, Table S4). Of these 911 hypothetical proteins, 479 were positive for a TpLRR profile, 655 were positive for the TpLRR pattern and 699 positive for either (additional file 1, Table S1). For 212 TvBspA entries their TpLRR diverged to the extent that they are neither recognized by the TpLRR profile nor pattern but all recovered other TvBspA or prokaryotic BspA-like proteins as their top BlastP hits (additional file 4, Table S4), hence we defined these as TvBspA entries with divergent TpLRR. In several cases the divergent TvBspA are clearly related to subfamily members that are positive for the TpLRR profile/pattern (additional file 1, Table S1), with some examples listed in Table 2. These analyses expand by ~250 entries the size of the TvBspA protein family identified based on BlastP searches only [26,36]. Notably, all identified TvBspA are clearly differentiated from *T. vaginalis *proteins with alternative LRR (additional file 5, Table S5 list some examples), none of which where hit by either the Wu-Blast or the PHI-/PSI-Blast search that identified the 911 TvBspA entries.

**Table 1:** Taxonomic distribution of genomes encoding BspA-like proteins

Taxa^a^	Best Bit score	No. hit^b^	Higher taxon	Habitat^c^
**Eukaryota**				

*Trichomonas vaginalis *G3	774	595 (908)	Parabasala	Urogenital (human)

*Entamoeba dispar *SAW760	205	34 (298)	Amoebozoa	Intestinal (human)

*Entamoeba histolytica *HM-1:IMSS	143	27 (124)	Amoebozoa	Intestinal (human)

**Archaea**				

*Methanosarcina barkeri *str Fusaro	251	2 (2)	Euryarchaeotes	Aquatic & rumen

*Methanosarcina acetivorans *C2A	244	3 (3)	Euryarchaeotes	Aquatic & rumen

*Methanococcus vannielii SB*	126	1 (2)	Euryarchaeotes	Aquatic

*Methanococcus maripaludis C7*	100	2 (2)	Euryarchaeotes	Aquatic

**Bacteria**				

*Eubacterium siraeum *DSM 15702	249	7 (7)	Firmicutes	Intestine (human, HMP)

*Flavobacterium psychrophilum *JIP02/86	236	15 (15)	CFB group^d^	Fish pathogen

*Clostridium leptum *DSM 753	229	1 (1)	Firmicutes	Intestine (human, HMP)

*bacterium *Ellin514	199	2 (2)	Verrucomicrobia	Soil

*Syntrophomonas wolfei*^e^	193	1 (1)	Firmicutes	Aquatic & rumen

*Clostridium spiroforme *DSM 1552	178	1 (1)	Firmicutes	Intestine (human, HMP)

*Clostridium beijerinckii *NCIMB 8052	174	5 (5)	Firmicutes	Intestine, aquatic

*Epulopiscium *sp 'N.t morphotype B'	174	19 (19)	Firmicutes	Intestine (fish)

*Victivallis vadensis *ATCC BAA-548	167	3 (3)	Verrucomicrobia	Intestine (human)

*Shewanella pealeana *ATCC 700345	155	3 (3)	γ-proteobacteria	Nidamental glands (squid)

*Anaerofustis stercorihominis *DSM 17244	153	12 (15)	Firmicutes	Intestine (human, HMP)

*Bacteroides fragilis *NCTC 9343	148	2 (4)	CFB group	Intestine (human)

*Treponema denticola *ATCC 35405	146	5 (11)	Spirochetes	Oral cavity

*Alistipes putredinis *DSM 17216	146	6 (7)	CFB group	Intestine (human, HMP)

*Synechococcus *sp. WH 7805	141	2 (3)	Cyanobacteria	Marine & host associated

*Ruminococcus torques *ATCC 27756	138	4 (6)	Firmicutes	Intestine (human, HMP)

*Bacteroides ovatus *ATCC 8483	134	4 (5)	CFB group	Intestine (human, HMP)

*Clostridium *sp. L2-50	117	4 (10)	Firmicutes	Intestine (human, HMP)

*Ruminococcus torques *ATCC 27756	113	4 (6)	Firmicutes	Intestine (human, HMP)

*Clostridium butyricum *5521	112	2 (2)	Firmicutes	Intestine (human)

*Coprococcus eutactus *ATCC 27759	107	3 (6)	Firmicutes	Intestine (human, HMP)

*Photobacterium sp. SKA34*	104	1 (1)	γ-proteobacteria	Aquatic, host associated

*Kordia algicida OT-1*	103	1 (1)	CFB group	Aquatic, algae pathogen

*Bacteroides stercoris *ATCC 43183	99	4 (6)	CFB group	Intestine (human, HMP)

*Clostridium phytofermentans *ISDg	99	4 (6)	Firmicutes	Soil

*Clostridium bartlettii *DSM 16795	*96*	2 (3)	Firmicutes	Intestine (human, HMP)

*Desulfitobacterium hafniense *Y51	91	1 (1)	Firmicutes	Soil

^a^The order of appearance of the three Domains of life is defined by the taxon with the highest PHI-Blast bit score. The taxa of a given Domain are then listed by decreasing bit score. Strains/isolates are indicated.

^b^Taxa with bit score ≥ 90 in the initial PHI-Blast (see Methods section) are listed here. additional file 2, Table S2 has the complete taxa and sequences accessions list. The listed eukaryotes and Archaea were the only taxa recovered in the search. The numbers in brackets are the corresponding numbers of hits from a PSI-Blast search - two iterations with e-score ≤ 1e^-5 ^following the aforementioned PHI-Blast search. The complete list of taxa and sequences accessions for the PSI-Blast search are in additional file 3, Tables S3 (*Trichomonas *and *Entamoeba*) and additional file 9, Table S6 (Bacteria and Archaea).

^c^Taxa isolated from human tissues might be present in other mammalian species as well. HMP: indicates species being sequenced in the context of the Human Microbiome Project [101].

^d^CFB, Cytophagales/Green sulfur bacteria group syn. Bacteroidetes/Chlorobi group.

^e^Complete denominations: *Syntrophomonas wolfei *subsp wolfei str Goettingen

**Table 2:** Features of the 18 TvBspA proteins encoded by scaffold DS113361

Locus tag^a^	Align^b^	Protein length	LRR^c^ start	LRR^c^ end	Pa/Pr^c^	E-value^d^	TMD^e^ position	Notable features^f^
**Subfamily #22** (14 entries)								

TVAG_133050	110	837	15	796	1	2.0E-69		Conserved C-terminus

**Subfamily #200**								

TVAG_133260	649	285	56	273	0	3.0E-21		

TVAG_133330	650	666	23	656	0	7.0E-43		

TVAG_133300	651	566	65	517	1	4.0E-46	468-490, 539-561	

TVAG_133380	652	174	38	173	0	9.0E-16		

TVAG_133420	653	392	66	390	0	3.0E-29		SP

TVAG_133230	678	205	3	201	1	9.0E-26		

TVAG_133270	679	205	3	201	1	9.0E-26		

**Subfamily #228**								

TVAG_133390	680	638	3	555	2	4.0E-43		

TVAG_133240	681	601	5	358	0	3.0E-26	513-535	Conserved CT

TVAG_133280	682	583	5	352	0	7.0E-26	495-517	Conserved CT

TVAG_133310	683	595	4	382	1	4.0E-30	505-527	Conserved CT

TVAG_133340	684	448	3	210	1	3.0E-17	358-380	Conserved CT

TVAG_133430	685	734	3	474	1	2.0E-35	744-666	Conserved CT

**Subfamily #275**								

TVAG_133290	689	318	3	227	2	8.0E-34		

TVAG_133410	690	562	1	426	1	8.0E-40		

TVAG_133360	691	333	2	263	1	5.0E-26		

TVAG_133220	692	363	51	362	0	2.0E-31		

^a^The locus tag numbers give an indication of the proximity of the different TvBspA encoding genes on the scaffold, with an incremental of 10 for each neighbouring genes (see additional file 8, Figure S3). The numbers of entries for each of the four proteins subfamilies identified by CLUSS2 (additional file 1, Table S1) are indicated.

^b^Alignment position in the output file generated by ClustalW. The position of the 18 TvBspA proteins in the global TvBspA alignment (additional file 1, Table S1) is indicated.

^c^The position of TpLRR inferred by BlastP (see Methods section). The numbers indicate the start and end of the TpLRR. The entries positive for either the TpLRR profile or pattern (Pa/Pr), or both, are indicated by a 1 and 2 respectively.

^d^E-values for the PSI-Blast search (2^nd ^iteration) that followed a PHI-Blast (see Methods section).

^e^Entries with inferred transmembrane domains (TMD) are indicated with the shown start and end position of the TMD. All such entries had the N-termini with the TpLRR domain inferred to be "external" by two or more TMD predictors (see Methods section).

^f^TVAG_133420 has an inferred signal peptide (SP). The five entries from subfamily #228 with a TMD all share a similar cytoplasmic tail - see Figure 3.

Only one TvBspA gene is annotated to possess two exons and 98 entries are annotated as pseudogenes due to stop codons or frame shifts interrupting the inferred ORF (additional file 1, Table S1). Eight TvBspA putative proteins do not possess a starting methionine and three of these are very similar to longer proteins with a starting methionine. There were also 14 TvBspA genes with some ambiguous sequencing data (with tandem repeats of N: A, C, G or T). In addition, a total of 17 TvBspA proteins were derived from ORF that start or end at the extremity of a scaffold suggesting they represent partial sequences, two of which have EST support (additional file 1, Table S1). Hence a total of 137 TvBspA ORF correspond to either mis-annotated, error-containing sequences, derived from genes with overlooked introns, pseudogenes, gene fragments, partially sequenced genes, or have sequencing errors/ambiguities. We focused our more detailed analyses on TvBspA protein sequences most likely derived from full-length genes based on their sequence features, genome context and evidence for transcription.

To allow comparisons of TvBspA protein sequence features and to provide estimates of their phylogenetic relationships, protein alignments and protein subfamilies were computed. These data were used to rationalise the vast structural diversity of the TvBspA proteins and contextualise their genomic organization. Two multi alignments of TvBspA proteins were generated to allow their comparisons: (i) all 911 sequences (additional file 6, Figure S1) and (ii) all 193 proteins with transmembrane domains (TMD) and inferred C-terminal cytoplasmic tails (CT, CCT) (additional file 7, Figure S2). The order of the sequences in the alignment is a reflection of their relatedness with the most similar sequences typically being aligned next to each other and the more divergent sequences overall tend to be aligned last during the alignment estimation and these are located towards the bottom of the alignment [37] (additional file 1, Table S1). The position of the TvBspA sequences in the two alignments were contrasted with TvBspA protein subfamilies derived from an alignment-free clustering algorithm designed to deal, to some extent, with hard to cluster (and align) repeat containing proteins [38]. A total of 397 subfamilies were identified for the 911 TvBspA (additional file 1, Table S1). In numerous cases there was a good agreement between the subfamilies membership and their juxtaposed position in the alignments (additional file 1, Table S1).

Due to the highly repetitive nature of the *T. vaginalis *genome the current genome sequence data are fragmented over 17,000 scaffolds [36], consequently it was only possible to generate a partial picture of the genome organisation for the TvBspA gene family. The 911 TvBspA candidate genes were scattered over 440 scaffolds (size range 1 kbp to 585 kbp, mean 101.4 kbp), with 245 scaffolds encoding one TvBspA and 195 scaffolds encoding two or more TvBspA (up to 18 TvBspA, Table 2) making up the majority and remaining 666 entries. Many TvBspA genes are organised in clusters either in tandem repeats, or in close proximity to each other, with the largest cluster made of 17 TvBspA genes (and five unrelated interspersed genes) over a genomic segment of 46.5 kbp (Table 2; additional file 8, Figure S3). Some of the clustered genes encoded proteins highly similar to each other and are also recovered in the same protein subfamily and/or were aligned beside each other in the global (911 TvBspA) alignment (additional file 6, Figure S1; additional file 1, Table S1). Such patterns suggest local gene duplications generating tandem repeats [39]. We also identified in several cases closely related paralogues (additional file 1, Table S1), which were encoded by genes present on different scaffolds, reminiscent of ectopic duplications events (Table 2 lists one example)[39].

The extensive size of the TvBspA gene family is currently unparalleled. The combined PHI- and PSI-Blast searches that recovered 908 *T. vaginalis *proteins recovered one to 298 BspA-like sequences in 154 additional RefSeq annotated genomes (Table 1; additional file 2, Table S2, additional file 3, Table S3 and additional file 9, Table S6). The next largest BspA-like gene families were found in *Entamoeba dispar *(298 EdBspA entries) and *Entamoeba histolytica *(124 EhBspA entries), the only other eukaryotic genomes, with *T. vaginalis*, currently known to encode proteins with TpLRR [26,40] (Tables 1, additional file 3, Table S3). In contrast, prokaryotes encoded fewer BspA-like genes (one to 19 entries) but their taxonomic diversity was much broader including five archaeal species and 147 bacterial species/strains, with the majority of bacterial taxa being members of Firmicutes (70%) or the Bacteroidetes (12%) (additional file 9, Table S6).

### Structural diversity of TvBspA proteins

**Figure 1:**
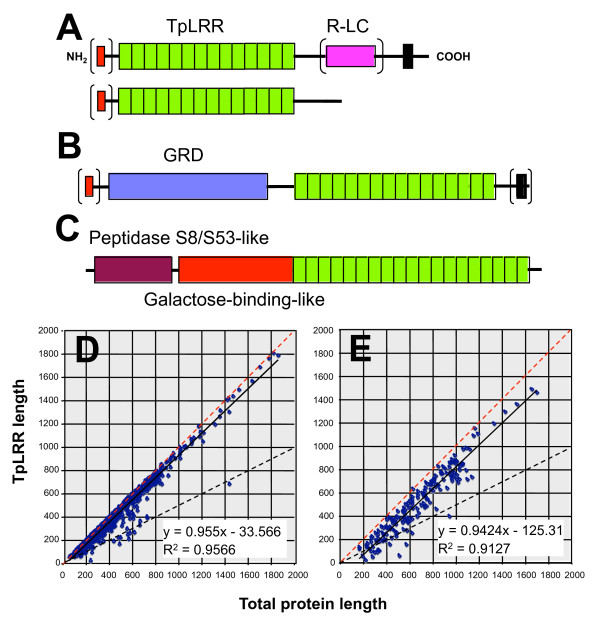
**Structural diversity of the TvBspA proteins**. **(A) **Proteins with an inferred TMD (black boxes) are contrasted with those without them. In both case a fraction of entries are inferred to posses a SP (red boxes, brackets illustrate variability). A few TvBspA have more then one TMD but these are not depicted. In addition to the TpLRR (green boxes) some proteins also possessed other type of repeats (R) or low complexity segments (LC) of various lengths (pink box) typically located between the TpLRR and the TMD (additional file 10, Table S7). Notable structural variants are illustrated in panels B and C and variation in the length of the TpLRR in panels D and E. **(B) **The 12 TvBspA entries forming a subfamily that share a glycine-rich domain (blue box, GRD). Their TpLRR lengths range from 16 to 20 repeats. Four proteins have an inferred SP and one has a TMD. **(C) **A TvBspA entry with InterProScan hits indicating a partial peptidase S8/S53-like domain (purple box - Pfam domain PF00082) and a galactose-binding domain (red box - SUPERFAMILY domain SSF49785), both of which are typically found in extracellular proteins. One TvBspA proteins was also identified to possess ankirin-like repeats at its C-terminus end (data not shown). **(D) **Graph illustrating the relationship between the TvBspA TpLRR length and corresponding total length for proteins without TMD. The linear regression is shown (black line) and is contrasted with the y = x line (red dashed) and y = 1/2 x line (black dashed). **(E) **Corresponding graph (as shown in D) for the 193 TvBspA with TMD-CCT.

**Figure 2:**
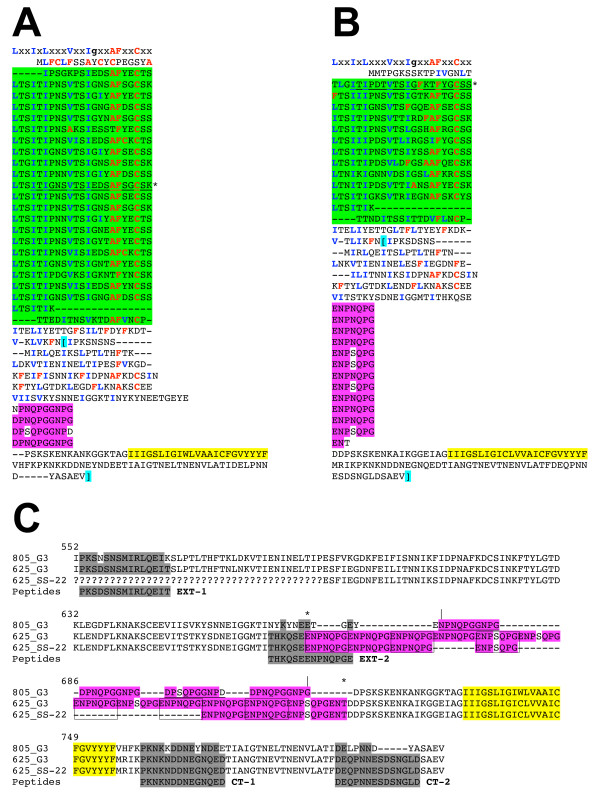
**Comparison of two TvBspA paralogues in isolate G3 and TvBspA orthologue between isolate G3 and SS-22**. The two proteins TvBspA625 (TVAG_073760) and TvBspA805 (TVAG_154640) have the highest level of identity, 72%, between members of subfamily #13 (see text). **(A) **The TvBspA805 protein sequence with the TpLRR (green) and P/NRR (violet) aligned to compare individual repeats and their corresponding features in TvBspA625. The star indicates the first TpLRR that is part of the BlastP TvBspA625/TvBspA805 alignment (underlined segment) and that extends up to the C-termini. The inferred TMD is highlighted in yellow. **(B) **The corresponding TpLRR and P/NRR alignments for TvBspA625. The number of TpLRR is greater in TvBspA805, whereas the P/NRR is more extensive in TvBspA625. **(C) **Alignment comparing the C-terminal end of the TvBspA805 (G3 isolate: 805_G3) and TvBspA625 (G3 isolate: 625_G3, identical to sequence of isolate C-1:NIH, GenBank accession: AAM51159) proteins with the corresponding partial sequence of TvBsp625 derived from the clinical isolate SS-22 (625_SS-22, GenBank accession FJ881695). In TvBspA805 every second P/NRR (aligned in A) are underlined whereas for the TvBspA625 proteins every second P/NRR (aligned in B) are boxed. TvBspA625_SS-22 (7 P/NRR) has five fewer P/NRR compared to TvBspA625_G3 (12 P/NRR). The alignments of repeats were manually adjusted to maximise identity levels between repeats. Missing sequence data for the sequence from isolate SS-22 are indicated by question marks. The aligned sequences correspond to the segments between [----] in panels A and B. The sequences of the four peptides synthesized to generate mouse antisera and used to test antibody response in *T. vaginalis *infected patients are shaded in grey with differences highlighted in white (see text).

Although some TvBspA proteins are structurally very similar to each other, the TvBspA protein family overall is characterised by important variations in length and sequence of their TpLRR and other sequence features when present, including other tandem repeats or low complexity segments, TMD and CT (Figures 1; Figure 2; additional file 6, Figure S1; additional file 1, Table S1, additional file 10, Table S7). The start and end positions of the TpLRR of each TvBspA proteins were compared to investigate their length and contextual position with other sequence features (Figure 1; additional file 1, Table S1). The TvBspA protein lengths ranged from 58 to 1865 residues (mean 539) with the TpLRR representing overall the major contributor to protein length (mean 84%) with the TpLRR length correlating well with protein overall length (Figure 1D, E). For the 193 TvBspA proteins with a TMD and CCT (TMD-CCT) the contribution of the TpLRR to total protein length is overall reduced (mean 72%) and more scattered due to the contribution of the TMD, CT and linker sequences between the TMD and the TpLRR (TpLRR-TMD linker) (Figure 1A, E). The TpLRR-TMD linker sequences for 24 TvBspA with TMD-CCT were characterised by additional repetitive or low complexity segments often enriched in proline or serine residues (Figures 1; additional file 10, Table S7; additional file 11, Figure S4). A few cases of TvBspA proteins were inferred to possess distinctive domains identified by visual inspection of their alignments (Figure 1B and see below) or through an InterProScan search (Figure 1C).

TpLRR containing proteins are thought to be extracellular either as surface exposed or secreted proteins [26,41]. Notably, *T. vaginalis *encodes none of the enzymes required for glycosylphosphatidylinositol (GPI)-anchors synthesis and mediating their anchoring to proteins [36], hence we focused our more detailed sequence analyses on entries with potential signal peptides (SP), TMDs or conserved features located towards the C-terminal end in the absence of evidence for TMD (Figure 1; additional file 1, Table S1). The 193 entries with TMD-CCT (additional file 7, Figure S2) have an inferred membrane topology implying that the entire TpLRR would be exposed to the extracellular milieu if these were to be expressed on the cell surface. Of these 193 TvBspA-TMD-CCT entries 35 also had an inferred SP, defining type I membrane proteins and the 158 entries with no inferred SP defined potential membrane proteins of type III [42]. Among the 719 TvBspA proteins that were considered not to possess TMD, 92 had a detectable SP (Figure 1) and several of these also formed co-aligned subfamilies characterised by conserved C-termini often ending with hydrophobic residues and possessing conserved cysteines and other residues within motifs located ~20-30 residues from the C-terminus (additional file 6, Figure S1).

To initiate the rationalisation of the potential functional significance of the considerable TvBspA protein family, we further investigated their structural diversity and gene expression at the transcript level in different *in vitro *culture conditions. Selected TvBspA proteins with notable sequence features or included in large subfamilies were also investigated in more details. Our bioinformatic analyses also included the identification of repetitive sequences (in addition to the TpLRR) that are often linked with surface proteins important for host-pathogen interactions in many pathogenic bacteria and microbial eukaryotes and are directly implicated in virulence and pathogenicity, including adhesion to host tissues and immune evasion [43,44].

The first investigated TvBspA protein sequence, TvBspA625 [28] (Genbank accession AAM51159, corresponding to TVAG_073760 and XP_001321233, TrichDB and RefSeq accession numbers respectively, see additional file 1, Table S1 for all 911 accessions numbers) was recovered among the nine sequences of subfamily #13. TvBspA805 (TVAG_154640) (the number after TvBspA indicates the inferred number of amino acids) was the most similar to TvBspA625 (72% identity) when compared to other members of subfamily #13 (range of pairwise identity: 37% to 54%). The two sequences share a TpLRR-TMD linker segment made of proline and asparagines-rich repeats (P/NRR) (Figure 2) and they are encoded on different scaffolds and separated by at least 67 kbp (additional file 1, Table S1) if not located on different chromosomes. Variations in the number of TpLRR and the P/NRR between TvBspA625 and TvBspA805 indicate differential contractions and expansions of these repetitive segments between the two paralogues (Figure 2). The variations in repetitive sequences of proteins from microbial pathogens are an important source of genetic variations between species/isolate/strains and are thought to correspond to dynamic adaptive responses in host-pathogen interactions (e.g. [43,44]). Hence we PCR cloned a 3'end segment of the TvBspA625 gene from the clinical isolate SS-22 [45] that encompasses the P/NRR, TMD and CT to compare it with the corresponding G3 sequence (Figure 2C). Although the TvBspA625 protein sequences of isolate G3 and C-1:NIH were identical (Figure 2C) the amplicon of isolate SS-22 was slightly smaller compared to the control amplicon obtained from isolate G3 (data not shown). Sequencing revealed that this difference was due to a reduced number of ENP [NS]QPG repeats (12× P/NRR in protein from isolate G3 and C-1:NIH) with five fewer repeats in isolate SS-22 (7× P/NRR) (Figure 2C). For reason we currently don't understand, several attempts to PCR clone the entire TvBspA625 ORF from strain SS-22 failed using both genomic DNA and cDNA as template. As the entire TvBspA625 ORF could be amplified and sequenced from isolate G3 genomic DNA in control PCR this suggests differences in the 5'end of the TvBspA625 gene between the two clinical isolates and together with the differences in P/NRR indicate that this gene readily accumulate changes between clinical isolates. All members of subfamily #13 did also co-align in the global TvBspA alignment with one intercalated aligned sequence not included in subfamily #13 (additional file 1, Table S1). Four members of subfamily #13 possess a TMD-CCT. In addition to TvBspA625 and TvBspA805, TvBspA786 (TVAG_234090) was also characterised by a PRR in the TpLRR-TMD linker (16 prolines over 49 residues including two NPTPETP repeats) (additional file 6, Figure S1; additional file 10, Table S7).

Two highly similar paralogues TvBspA515 and TvBspA575 (92% identity - TVAG_244780 and TVAG_244800, respectively, members of subfamily #384) were characterised by TpLRR-TMD linker sequences with serine-rich repeats (SRR). The length variation between the two proteins was essentially restricted to the SRR (additional file 6, Figure S1; additional file 11, Figure S4) reminiscent of the variation identified between TvBspA625 P/NRR from two clinical isolates (Figure 2C).

**Figure 3:**
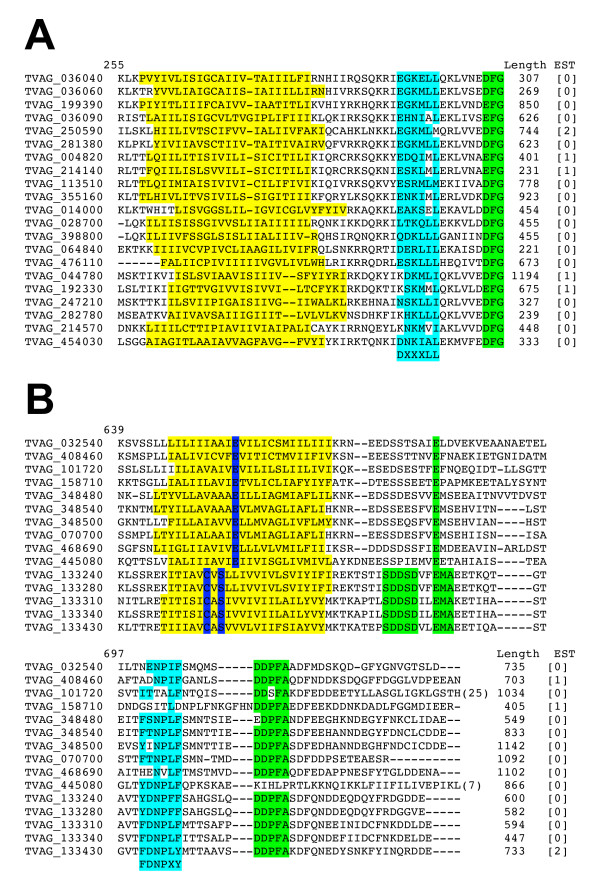
**Alignment of TvBspA with TMD and similar cytoplasmic tails with potential signal for endocytosis**. **(A) **The 21 identified TvBspA with similar CT (sequence and length) all ending with the pattern [DE]FG (green). Most sequences possess a motif related to [DE]XXXL [LI] (blue), a dileucine-based type endocystosis and lysososmal-like sorting signal (see text). Residues replacing [DE] in known functional endocytosis signal in other species are also shown in blue (see text). **(B) **The 15 identified TvBspA sequences with a similar TMD (yellow) and CT. Most entries share the FDNPX[YF] (blue) an NPXY-type endocytosis signal (see text). The five entries that are part of subfamily #228 (bottom five sequences, see text and Table 2) also share a more similar TMD compared to the other 10 entries. Conserved residues in the TMD differentiating the two sets of sequences are highlighted in dark blue.

Inspecting the alignment of the 193 TvBspA with TMD-CCT identified several related sequences (co-aligned) based on shared CT sequences. The largest group was made of 21 sequences sharing related TMDs and a CTs ending with the pattern [DE]FG and most of these also possessed the dileucine-like motif DXXXLL known to function as sorting signals for rapid endocytosis and lysosomal targeting in other eukaryotes [46] (Figure 3A). The next largest group with a shared TMD-CCT was made of 15 proteins sharing the motifs [DE]D [PS]FA and the minimal NPXY-like signal (12 entries) for rapid endocytosis [46] (Figure 3B).

Among the 55 sequences with reduced contribution of the TpLRR (≤ 55%) to overall protein length (additional file 1, Table S1), 12 were characterised by distinct glycine-rich domains (GRD) located at the N-terminus, followed by the TpLRR of various lengths (Figures 1B; additional file 6, Figure S1; additional file 12, Figure S5). These 12 TvBspA-GRD proteins were the exclusive members of subfamily #168. Six TvBspA-GRD proteins were encoded by two gene clusters (additional file 1, Tables S1). The 12 TvBspA-GRD proteins were co-aligned together indicating relatively recent gene duplication events, which in two cases corresponded to local duplications. BlastP searches with the GRD domain from the TvBspA-GRD proteins as query recovered 12 none-*Trichomonas *sequences in RefSeq, all annotated as hypothetical proteins and encoded by a taxonomically very restricted set of only nine genomes (Table 3; additional file 13, Table S8). The Blast hit list also included additional *T. vaginalis *sequences without TpLRR (additional file 13, Table S8), these were not further investigated here. Visual inspection of the GRD alignment indicated that the different GRDs are likely to be homologous to each other with conserved positions including residues beyond the shared glycines (additional file 12, Figure S5). One TvBspA-GRD member possessed a TMD and four entries without TMD possessed a potential SP, as did the protein from *Flavobacteirum bacteria *(Figures 1B; additional file 12, Figure S5) suggesting that these could be expressed on the surface or secreted. The four TvBspA-GRD with an inferred SP corresponded to proteins with an N-terminal extension that is shared between a total of nine TvBspA-GRD (eight being very similar to each other), suggesting that all the sequences with an extension could possess functional SP with some currently not recognized as such by SignalP or PHOBIUS (additional file 12, Figure S5). Three TvBspA-GRD did not possess such extension, as did the proteins from *C. difficile *and the phages (additional file 12, Figure S5).

**Table 3:** Taxonomic distribution of proteins sharing a glycine-rich domain found in 12 TvBspA.

Taxa	Bit Score^a^	E-value^a^	No. of hits^b^	Top hit annotation
*Trichomonas vaginalis *G3	335	2.0E-90	12 (51)	Surface antigen BspA-like

*Flavobacteria bacterium MS024-3C*	84	8.0E-15	1	Hypothetical protein

*Clostridium *phage phiCD27	71	6.0E-11	1	Hypothetical protein

*Clostridium *phage phi CD119	71	7.0E-11	1	Hypothetical protein

*Clostridium difficile *ATCC 43255	70	7.0E-10	2	Hypothetical protein

*Clostridium difficile *QCD-63q42	70	2.0E-10	3	Hypothetical protein

*Clostridium difficile *QCD-37×79	68	6.0E-10	1	Hypothetical protein

*Clostridium difficile *630	65	6.0E-09	2	Hypothetical protein

*Bacillus cereus Rock4-18*	58	4.0E-07	1	FG-GAP repeat protein

^a^The values for the top BlastP hits (query: TVAG_174900 GRD, residues 1-267, E-value <1.0E-4) only are shown. The complete taxonomic report listing all hits is given in additional file 13, Table S8. The taxa are listed by decreasing bit scores. The shown values for *T. vaginalis *correspond to the non-self top hit, entry TVAG_191460.

^b^The values give the number of hits per genome. The 12 TvBspA possessing the GRD were hit and include the query sequence. There is an additional 51 *T. vaginalis *proteins that possess a related GRD that do not possess TpLRR.

### Evidence for TvBspA genes transcription

**Figure 4:**
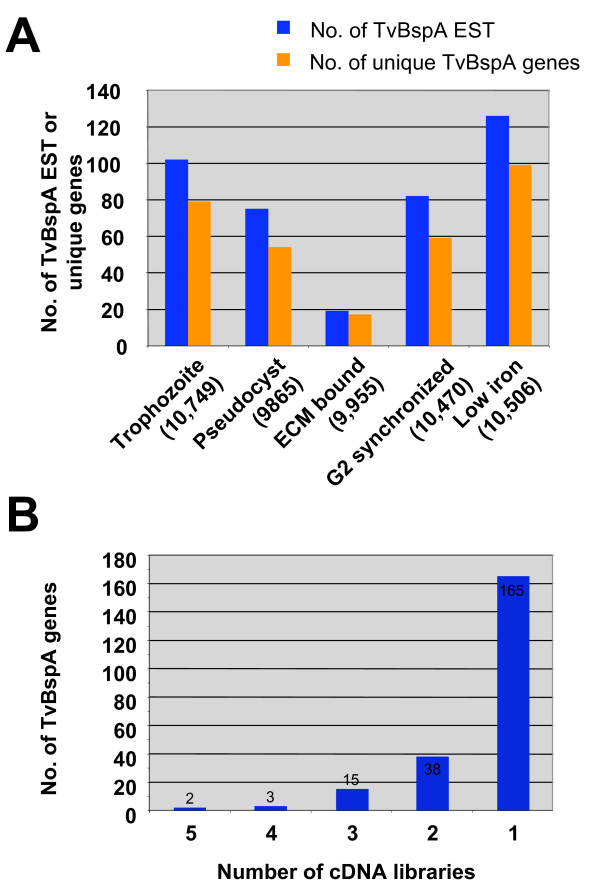
**Evidence for differential TvBspA gene expression from EST**. **(A) **EST derived from different growth conditions were scanned for entries corresponding to TvBspA genes. The total numbers of distinct TvBspA EST (blue bar) or different unique TvBspA genes (orange bar) for a given condition are indicated. For each condition ~10,000 EST were compared (numbers in brackets). **(B) **The different libraries are characterised by little overlap in terms of EST encoding TvBspA with only two TvBspA genes shared between all five compared conditions and most found in only one library.

To investigate TvBspA genes transcription we explored three sources of data including EST, semi-quantitative RT-PCR and microarrays with quantitative RT-PCR (qRT-PCR) (additional file 14, Table S9). A total of 270 TvBspA genes (29.6% of all 911 entries) had hits on one ore more EST, with the majority having only one hit (57.8%), with approximately the same proportion of TvBspA without and with TMD (30% and 27% respectively) having EST support indicating that both structural types are transcribed and likely to be functional (additional file 15, Table S10). Different EST libraries derived from five different culture conditions were characterised by little redundancy and overlap in terms of their TvBspA transcripts suggesting differential expression of the TvBspA genes for the compared growth conditions (Figure 4).

A total of 74 different TvBspA cDNA were spotted on microarrays among a total of 4938 distinct genes and these were used to contrast transcripts concentration in *T. vaginalis *cultures exposed to high and low iron concentration, as iron is an important factor known to influence the expression level of many genes (at both transcript and protein level), the virulence, morphology and growth of the parasite [18,47,48]. A total of 13 TvBspA genes showed significant variation in their mRNA concentration between the tested conditions (Table 4) and the remaining 61 entries showed expression but without significant differences between the tested conditions (data not shown). For three TvBspA genes the modulations of transcript concentration was confirmed by qRT-PCR (Table 4).

**Table 4:** Microarray data for 13 TvBspA with significant modulation in their mRNA concentration upon exposure to different iron concentration

Locus tag	Annotation^a^	Subfamily membership^a, b^	EST^a^	**Modul**.^c^	p-value	qRT-PCR^d^
High iron culture condition						

TVAG_397210	BspA-like	#43, 10 members^e^	1	2.2	1.5 × 10-4	2.16 ± 0.002

TVAG_441420	BspA-like, SP, TMD	#374, singleton, divergent TpLRR	1	1.55	8.4 × 10-3	2.02 ± 0.04

TVAG_191490	BspA-like, SP, PG, GRD	#168, 12 members	1	1.29	5.5 × 10-3	---

TVAG_396970	BspA-like	#44, 15 members*	2	1.23	4.8 × 10-3	---

TVAG_080240	BspA-like	#44, 15 members*	6	1.18	8.5 × 10-3	---

						

Low iron culture condition						

TVAG_093850	BspA-like	#184, 3 members	2	-1.27	2.9 × 10-3	---

TVAG_299910	BspA-like	#69, 3 members *	16	-1.25	6.8 × 10-3	---

TVAG_493590	BspA-like	#384, 6 members	4	-1.24	3.7 × 10-3	---

TVAG_129450	BspA-like, PG	#30, 15 members	1	-1.18	2.4 × 10-5	---

TVAG_530030	BspA-like	#99, 2 members	7	-1.15	2.5 × 10-3	-2.06 ± 0.02

TVAG_341990	BspA-like	#215, 3 members	1	-1.14	3.7 × 10-3	---

TVAG_176920	BspA-like, TMD	#341, singleton, divergent TpLRR	1	-1.13	7.8 × 10-3	---

TVAG_235070	BspA-like	#73, Singleton	1	-1.12	3.9 × 10-4	---

						

Control genes						

TVAG_238830	Hydrogenosomal malic enzyme subunit B	---	---	1.67	1.5 × 10-4	2.51 ± 0.01

TVAG_165030	Cytosolic *malate dehydrogenase*	---	---	-1.33	2.8 × 10-4	-1.62 ± 0.03

^a^See additional file 1, Table S1 for full details. SP, signal peptide; TMD, transmembrane domain; PG, pseudogene; GRD, glycine-rich domain.

^b^SF, subfamilies identified with CLUSS2. *Related sequences identified by visual inspection of the alignment.

^c^Positive values indicate up-regulated genes and negative values down-regulated genes. All values are derived from quadruplets with indicated mean value (Log scale) and significance (p-values) of modulation.

^d^qRT-PCR was performed in quadruplets with indicated mean and standard deviations.

**Figure 5:**
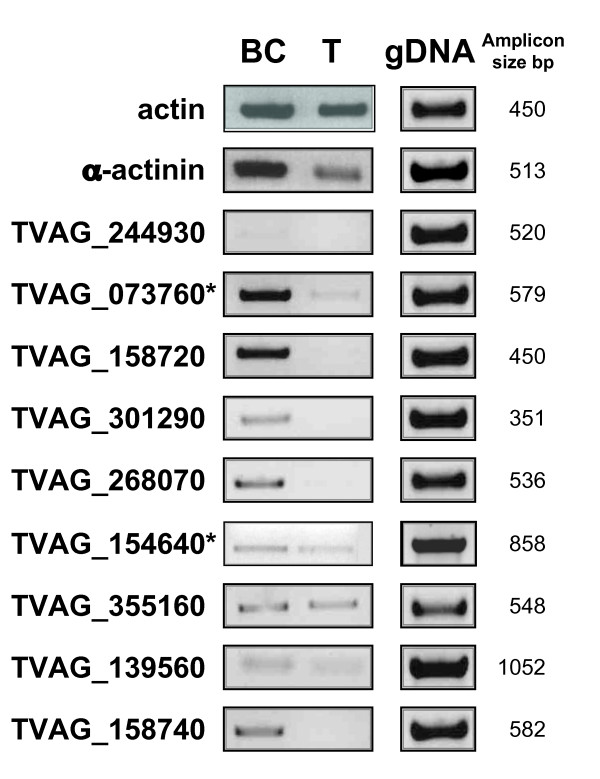
**Semi-quantitative RT-PCR assessment of TvBspA expression in *T. vaginalis *bound to ECM proteins**. Upon contact to the ECM proteins *T. vaginalis *cells strongly bind to the substrate and the majority of cells cannot be washed off after 60 min of incubation. RT-PCR for nine selected TvBspA-like genes and control actin (no major change) and alpha-actinin (up-regulated) genes was performed on *T. vaginalis *ECM proteins bound cells after 60 min of incubation (BC) and control free swiming trophozoites incubated in parallel (T). The shown results are representative of 6 to 15 independent PCR and at least five independent binding experiments/RNA extractions. TvBspA gene-specific primers were designed to produce amplicons of different sizes and avoid cross-amplification between TvBspA genes, in particular between the closely related TvBspA625 (TVAG_073760) and TvBspA805 (TVAG_154640) indicated by a star. A control PCR was also performed on genomic DNA (gDNA) confirming that the designed primers are efficient in generating the specific amplicons (size are indicated). Actin amplicons are equivalent for cDNA preparations from both trophozoite and ECM bound cells indicating similar total cDNA load whereas the cDNA specific for alpha-actinin increases upon *T. vaginalis *binding to substrate as described [97]. Five TvBspA genes show clear increase in the amount of amplicons for the ECM bound cells suggesting transcription up-regulation or higher stability of their mRNA in this condition. For TvBspA605 no amplicon could be detected suggesting that it is not transcribed in either tested conditions. For TvBspA805 the amount of cDNA was doubled to allow the detection of the shown signal indicating that the mRNA encoding this protein is not as abundant as for the paralogue TvBspA625.

Semi-quantitative RT-PCR was also used to contrast the mRNA concentration of nine TvBspA genes upon binding of *T. vaginalis *cells to ECM proteins, which is also known to influence the parasite morphology and gene expression [15,17]. We selected nine TvBspA genes encoding proteins with TMD based on their sequence features including TvBspA625 and TvBspA805 (Figure 2) and seven additional entries including two TvBspA with divergent TpLRR (Figure 5; additional file 11, Figure S4; additional file 16, Table S11). Marked differences of expression patterns were observed among the analysed genes with one showing no expression in both tested conditions and five showing an obvious increase in their mRNA concentration upon binding to ECM proteins (Figure 5). Notable was the striking difference between the two similar paralogues (Figure 2) TvBspA625 (TVAG_073760, up-regulated upon binding to ECM) and TvBspA805 (TVAG_154640, no observable changes and lower amount of transcript) (Figure 5).

Consistent with these differences in pattern of expression for the tested conditions, marked differences in the upstream sequences to the TvBspA start codon could be observed, suggesting different promoters for each gene potentially mediating differential transcriptional regulation (additional file 1, Table S1). The majority of TvBspA genes possessed known *T. vaginalis *core promoter elements including an initiator (Inr) (735 entries) or TATA-boxes only (17 entries) with 159 genes possessing neither (additional file 1, Table S1). Interestingly there was no significant difference in the proportion of TvBspA genes with EST or without EST among entries positive (41%) or negative (47%) for Inr/TATA-box suggesting that other core promoter elements for transcription exist in TvBspA genes. It will be interesting to investigate promoter sequence features for TvBspA and other protein coding genes when global tanscriptomics data will be generated for *T. vaginalis*.

### Cellular localization of TvBspA625 and its expression during infection

In order to investigate the expression and cellular localization of one TvBspA-like candidate surface protein four synthetic peptides were produced derived from the TvBspA625 sequence (Figure 2C) and used to raise mouse antisera. Three peptides are likely to generate antisera specific for TvBspA625 (CT-1, EXT-2 and CT-2) whereas the fourth could possibly lead to antibodies cross-reacting with the paralogues TvBsp805 (EXT-1) (Figure 2 and see Methods). Western blot analyses on *T. vaginalis *total protein extracts with the mouse antisera raised against one of the cytoplasmic located peptides (CT-1) recognized a consistent major protein with an apparent molecular mass of ~52 kDa for both isolate G3 and SS-22, while all presera did not detect any material (additional file 17, Figure S6). However, the other antisera did not identify the same material and were often characterised by more complex banding patterns (G3: CT-2, EXT-1, EXT-2) or had a distinct major band ~40 kDa (SS-22: EXT-1). Since the pre-sera did not detect any proteins and the apparent molecular masses of the proteins detected by the different antisera did not match the theoretical one for TvBspA625 (nor TvBspA805) from the G3 isolate (67 kDa) the lower apparent molecular mass could be explained by aberrant gel migration (sometime observed in proteins with repeats) or represent proteolytic fragments. As we currently don't know the corresponding genome sequence for isolate SS-22 it is difficult to interpret the observed differences between the two isolates.

**Figure 6:**
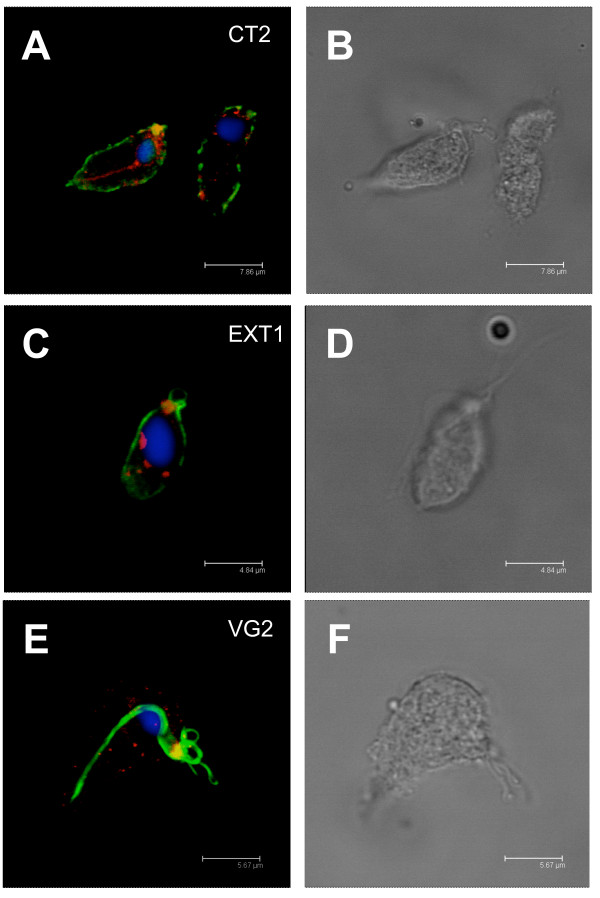
**Cellular localization of TvBspA625 by indirect immunofluorescence analysis**. *T. vaginalis *cells (G3 isolate) grown *in vitro *were processed for IFA (ethanol fixation) with the four mouse antisera raised against the four peptides designed from TvBspA625 sequence (see Figure 2) and imaging performed with a confocal microscope. Three antisera (EXT-1, CT-1 and CT-2) gave consistent signals on the cell surface of the parasites whereas EXT-2 often gave strong labelling over most of the cells structures (additional file 18, Figure S7). **(A) **The mouse antisera raised against a peptide derived from the TvBspA625 cytoplasmic tail (CT-2, green) gave a clear surface labelling. Co-labelling of the hydrogenosomal malic enzyme (red) with a specific rabbit antiserum led to the labelling of intracellular structures as expected [49]. Controls consisted of specific mouse pre-sera or secondary anti-mouse antisera alone (no signal in both cases, data no shown) and a mouse monoclonal antibody raised against tubulin decorated with same secondary anti-mouse antibody (panel C), clearly demonstrated the specificity of the signal attributed to the anti CT-2 peptide antisera. **(B) **The corresponding DIC image of the panel A. DAPI was added to the mounting media to label the nuclei (blue). The scale bar is shown. **(C) **Corresponding signal identified for the antisera raised against the TvBspA625 extracellular peptide (EXT-1, green) and processed as in A. **(D) **The corresponding DIC image of the panel B. **(E) **Corresponding signal identified with the mouse monoclonal VG2 raised against a *T. vaginalis *tubulin protein and processed with the same secondary anti-mouse antisera (green) and anti-ME rabbit antisera (red). **(F) **The corresponding DIC image of the panel E.

Indirect immunofluorescence analyses (IFA) with confocal microscopy demonstrated a clear cell surface localization for the TvBspA625 protein for three out of four mouse antisera with either ethanol fixed cells (Figure 6) or formaldehyde fixed cells (additional file 18, Figure S7). In contrast one antisera (anti-EXT-2) generated a strong signal over most of many cells, making cellular localization inference more difficult (additional file 18, Figure S7). Mouse presera from the corresponding anti-peptide antisera generated no signal and both the hydrogenosomal malic enzyme (HME, rabbit polyclonal [49]) and tubulin (mouse monoclonal VG2 [50]) controls had clear intracellular localizations as expected (Figure 6). In formaldehyde fixed cells we could also observe an intracellular punctuate labelling, typically at the proximity of the cell surface, with anti-CT-2 and EXT-1 antisera, whereas the anti-CT-1 antisera resulted in mainly a cell surface signal (additional file 18, Figure S7).

To investigate whether the cell surface TvBspA625 protein is expressed during infection we measured the presence of antibody recognizing the four peptides (EXT-1/2, and CT-1/2) in sera from patients infected by *T. vaginalis *and contrasted these to control sera from *T. vaginalis *negative patients (Table 5). All four peptides were recognised by similar proportion of patients sera (no statistical differences in pairwise Pearson uncorrected tests) from tested patients (*T. vaginalis *infected vs. non-infected - CT-1: 68% vs. 16%; CT-2: 60% vs. 12%, EXT-1: 78% vs. 18% and EXT-2: 61% vs. 15%) with 85% of these patients recognizing two or more peptides and 88% at least one peptide (Table 5). Sera from *T. vaginalis *positive patients were significantly (female and male combined: Pearson uncorrected χ^2 ^= 105.603, p < 0.0001; OR: 35.9) more frequently recognizing one or more peptides in the ELISA assay when compared to controls patients (Table 5). The pool of *T. vaginalis *positive patients was also significantly more frequently HIV positive compared to the pool of *T. vaginalis *negative patients (female and male combined: Pearson uncorrected χ^2 ^= 9.380, p = 0.002; OR: 2.6) (Table 5), as is now well recognised [2,3,6].

**Table 5:** Patients antisera response to peptides derived from TvBspA625.


	***T. vaginalis *positive patients**			***T. vaginalis *negative patients**		

	**Female**	**Male**	**Total^a^**	**Female**	**Male**	**Total^a^**

**Positive ≥ 1 peptide**	89	59	148* (88%)	3	7	10*(16%)

**Negative to all peptides**	15	6	21* (12%)	33	18	51* (84%)

Total	104	65	**169**	36	25	**61**

						

**HIV positive**	71	56	127** (75%)	15	18	33** (54%)

HIV positive & positive ≥ 1 peptide	62	53	115	0	4	4

HIV positive & negative all peptides	9	3	12	15	14	29

**HIV negative**	33	9	42** (25%)	21	7	28** (46%)

HIV negative & positive ≥ 1 peptide	27	6	33	3	3	6

HIV negative & negative all peptides	6	3	9	18	4	22

^a^A total of 169 patients with sera positive for *T. vaginalis *total protein extracts were contrasted to 61 patients with sera negative for *T. vaginalis *total protein extracts. Fractions of respective samples are indicated (in %). The proportion of HIV positive sera from the same set of patients was also compared. Sets of values used for statistical test and calculation of respective % are indicated by * or **.

## Discussion

Initial analyses of the draft genome sequence of *T. vaginalis *(isolate G3) identified a plethora of candidate surface/secreted proteins among which the largest family was made of the TvBspA proteins with over 650 entries that share a type of LRR [26,36]. Here we describe additional TvBspA candidate proteins further extending the size of this considerable protein family and the first detailed analyses of the sequence structural diversity of all 911 TvBspA candidate proteins. We also investigated selected TvBspA transcripts from parasites grown in different conditions and provide for one TvBspA protein the first cellular localisation data as well as identify sequence variations between clinical isolates and patients antibody responses during *T. vaginalis *infection.

### TvBspA gene family tremendous diversity and origins

More comprehensive protein searches of the annotated *T. vaginalis *draft genome, made more sensitive and specific by combining pattern and profile based searches for TpLRR containing proteins, extended the TvBspA gene family by over 250 to a total of 911 entries. Searching the RefSeq protein database for BspA-like proteins with the same search strategy identified a taxonomically divers set of organisms, including two additional eukaryotes (*E. dispar *and *E. histolytica*) and a great majority (82%) of species derived from two major bacterial lineages, Firmicutes (70%) and Bacteroidetes (12%). Interestingly, Firmicutes and Bacteroidetes are the most prevalent taxa identified by gene surveys in the gut of vertebrates, including humans [51]. Indeed, the great majority of taxa encoding BspA-like proteins, including the three eukaryotes, are known to share the capacity to thrive on mucosal surfaces either as pathogens, commensals or mutualists (79% of taxa in Table 1). These data clearly reinforced earlier observations, made on a much more restricted set of sequenced genomes [28], that BspA-like proteins are preferentially encoded by mucosal microbes.

This pattern strengthened the hypotheses that lateral gene transfers (LGT) of BspA-like genes between microorganisms thriving on mucosal surfaces took place [28] and that BspA-like proteins are involved in important aspects of microbes-mucosa interactions. Mucosal surfaces; which include the colon, the niche with the highest known density of microbes [52]; harbour a large diversity of cellular microbes (mainly Bacteria but also Archaea and various microbial eukaryotes) between which LGT could take place, a mechanism thought to contribute to adaptations to a mucosal life style [26,52,53]. As the taxonomic diversity of genomes encoding BspA-like proteins is higher among prokaryotes we suggest that the only three eukaryotes (*T. vaginalis *and two *Entamoeba *species) currently known to encode BspA-like proteins acquired the corresponding genes from prokaryotic donors, likely bacterial species. The three TvBspA proteins with the highest level of sequence identity in BlastP alignments with prokaryotic proteins are with the Firmicute *Eubacterium siraeum *(TVAG_495790, 57% identity and TVAG_057870, 56% identity) and the Bacteroidetes *Tannerella forsythia *(TVAG_225790, 52% identity) (additional file 4, Table S4). Interestingly a bias was observed among candidate LGT genes identified in *T. vaginalis *with Firmicutes and Bacteroridetes being the most common identifiable candidate donors as supported by detailed phylogenetics (with proteins that lead to reliable alignments, without repeats) [36,54]. The extensive eukaryotic BspA-like gene families (911 in *T. vaginalis*, 298 in *E. dipsar *and 124 in *E. histolytica*), compared with the much restricted gene families found in prokaryotes (1-19 entries per genome) could be explained by one or a few LGT acquisitions from prokaryotic donors followed by large numbers of gene duplication events within the eukaryotic genomes, so called "conservative" gene duplications that are thought (along with LGT) to contribute to an organism adaptations to its environment [55]. Alternatively, the larger gene families observed in eukaryotes could be explained by several LGTs followed by less dramatic sets of gene duplication events. We favour the former hypothesis as few eukaryotic BspA-like entries show higher scores with their prokaryotic counterparts (only 15 TvBspA had prokaryotic proteins as top hits from nine different Bacteria and one Archaea) with the great majority (93%) of individual TvBspA protein recovering as top BlastP hits other TvBspA (additional file 4, Table S4). *Trichomonas *sequences are also rather distinct from the *Entamoeba *sequences both in terms of their TpLRR and overall structural organisation, consistent with independent gene acquisition and amelioration (functional integration) programs - only 19 TvBspA proteins have *Entamoeba *entries (one EhBspA and 18 EdBspA) as top Blast hit (additional file 4, Table S4).

Contrasting TvBspA gene positions on scaffolds with a TvBspA global alignment and subfamily composition (as surrogate to phylogeny) of the corresponding proteins indicated that both tandem and ectopic gene duplications events took place, as discussed for the large disease resistance gene family encoding proteins with LRR in plants [39]. The largest gene cluster made of 17 TvBspA genes was generated by a combination of a few ectopic and several tandem duplication events. More detailed information on the genome distribution of the TvBspA genes, for instance their potential locations in subtelomeric regions as known for important surface variant proteins in other pathogenic microbial eukaryotes [56], will await more extensive clustering of the >17,000 scaffolds and their mapping onto chromosomes [36,57].

### TvBspA protein structural diversity

Following gene duplications, TvBspA paralogues differentiated dramatically. This diversity was identified in both the sequence and number of the TpLRR with extensive overall length variation between TvBspA proteins from less then 100 to over 1800 residues. The compression and/or extensions of the TpLRR segments contributed to most of the observed length diversity but variation of other type of sequences are also involved including non-LRR repeats, TMD and CT, indicating an evolutionary highly dynamic gene family and suggesting that these proteins play several distinct functions. As TvBspA proteins are characterised by TpLRR typically present on extracellular proteins in other taxa [41], and additional repeats are also present in several cases, they are potentially involved in various aspects of host-pathogen interactions as shown for many repeat containing proteins including LRR [43,44,53,58,59]. A total of 193 TvBspA were inferred to possess TMD and CCT, supporting the hypothesis that the N-terminal ends of the proteins, including the TpLRR, face the extracellular milieu if expressed on the cell surface. Potential SPs were also detected for TvBspA with and without TMD. For proteins without TMD and with SP this suggests that these could be secreted or bound to the cell surface with unknown anchors - as there are no GPI-anchors in *T. vaginalis *[36]. Notably the current complete absence of experimental data for *T. vaginalis *SP probably contributes to underestimating the number of SP positive TvBspA entries *in silico *as SignalP3.0 and PHOBIUS were trained with a restricted diversity of eukaryotes [60,61]. Entries genuinely without SP and TMD could be secreted or anchored to the cell surface through unknown mechanisms or could represent non-functional proteins, perhaps corresponding to pseudogenes, although EST suggest that all types of TvBspA proteins are transcribed and could be functional. Several TvBspA without TMD (with and without detected SP) were also characterised by conserved C-terminal ends, including motifs with conserved cysteines and other residues and in some cases were ending with hydrophobic residues. Such conserved C-terminal motifs could be implicated in anchoring TvBspA proteins via unknown lipids (as GPI-anchor do not exist in *T. vaginalis*). Such motifs included the sequences [TS]**C**K in members of subfamilies #20, 22, 24, 33, 34 and 35, S**C**HIA for some members of subfamilies #44 and 45 or T**C**Q**C**R in subfamily #26 (additional file 1, Table S1; additional file 6, Figure S1). These TvBspA C-termini could be modified by hypothetical and 'atypical' lipid anchors for a eukaryotic surface exposed protein, as shown, or hypothesized, for some surface proteins from *E. histolytica *[62] including EhBspA proteins [40]. In the case of several EhBspA, the cysteine of a C-terminal CAAX motif (cysteine followed by two aliphatic residues and any terminal residue [63]) could be implicated and one CAAX containing EhBspA protein was indeed demonstrated to be expressed on the cell surface [40]. None of the TvBspA possessed a C-terminal CAAX box.

The important sequence variation observed between the TvBspA paralogues contrast dramatically with the high level of sequence conservation observed among copies of the highly repetitive genes made of virus-like, transposable elements (TE) and unclassified gene families, with a 2.4% average pairwise difference for an average gene copy number of 660, identified in *T. vaginalis *G3 genome (listed in Table two in Carlton et al. [36]). This suggests that the TvBspA genes are under some level of positive selective pressure whereas the virus-like, TE and unclassified highly repetitive genes families are evolving neutrally, as would be expected for proteins involved in host-pathogen interactions (potentially TvBspA) and selfish genes, respectively. It will be of great interest to contrast the level of selection pressure (neutral, positive or negative selection using ω = dN/dS - nonsynonymous-synonymous substitution rate ratio tests) on TvBspA genes and contrast these with other genes across several *T. vaginalis *isolate and one or more closely related species - required to generate DNA alignments of ORF to calculate ω values, the more sequences the better for tests measuring selection - e.g. [64].

One TvBspA subfamily was characterised by a shared N-terminal GRD followed by the TpLRR. BlastP searches with the GRD from TvBspA-GRD identified hypothetical proteins encoded by a very restricted set of genomes including *T. vaginalis*, *Flavobacteria bacterium*, four strains of *Clostridium difficile*, two *C. difficile *phages and *Bacillus cereus*. These data defined a new protein domain of unknown function that is shared between proteins with distinct C-termini currently encoded by few and distantly related taxa, *T. vaginalis *(a eukaryote), *F. bacterium *(Bacteroidetes-Chlorobi), *C. difficile *and *B. cereus *(both Firmicute), and two *C. difficile *phages, with all cellular organisms sharing the capacity to be potentially pathogenic to human mucosa. *Clostridium difficile *is the most common source of nosocomial diarrhea [65] and *F. bacterium *and *B. cereus *are commonly found in soil and water systems with *B. cereus *being a common opportunistic pathogen also causing pathologies in the digestive tract [66]. Members of the Flavobacteria are often pathogens (e.g. *Flavobacteria psychrophilum*, a virulent fish pathogen listed in Table 1) or opportunistic pathogens, including in humans [67]. This highly restricted and biased taxonomic distribution among specialised mucosal pathogens and potential opportunist mucosal pathogens is intriguing. In addition many prophages encode toxins and other virulent factors in pathogenic bacteria [53] and the phage GRD containing protein (ORF 30 in phage PhiCD119 [68]) is located beside of the holin protein, which is part of the cell lysis casset, and that corresponds to one of the preferential locations of toxins encoded by lambda-like prophages as know for the shiga toxin in subsets of *E. coli *strains [69]. Indeed, the two GRD containg proteins in the complete genome of *C. difficile *630 are encoded by genes (CD0967 and CD2397) with the same location (as in phage PhiCD119) within the two highly conserved prophages identified during annotation[65]. Holin is also homologous to the TcdE gene of the *C. difficile *pathogenicity locus harbouring the five genes tcdABCDE that is known to regulate the expression of the *C. difficile *toxins tcdA and tcdB [65]. Taken together, these different considerations suggest that proteins containing the newly identified GRD could be involved in some aspects of *T. vaginalis*-host interactions possibly by contributing to damaging bacteria of the vaginal microflora or human cells. It will be of interest to directly test this hypothesis experimentally with both *T. vaginalis *and *C. difficile *GRD-containing proteins.

Among relatively recently duplicated TvBspA genes (paralogues encoding proteins with high level of protein sequence identity, >70%, 198 pairs of paralogues - additional file 4, Table S4) we identified cases where paralogues accumulated differences in both the number of TpLRR and/or other repetitive sequences as often observed for proteins known to be involved in host-pathogen interactions [43,44,53]. Such variations in repetitive segments were also identified between TvBspA625 from two clinical isolates, with isolate SS-22 possessing a shorter P/NRR in the TpLRR-TMD linker sequence when compared to TvBspA625 from isolate G3. The incapacity to PCR clone the entire TvBspA625 ORF from isolate SS-22 also suggested variations at the 5'end of the gene corresponding to the TpLRR domain. Western blot analyses did detect the same major band with the anti-CT-1 antisera but identified some differences with the other antisera (CT-2, EXT-1/2) and sequencing of the full TvBspA625 holomologue of isolate SS-22 will be required to try to rationalise these results. Taken together these data demonstrated that TvBspA paralogues within a given genome and orthologues between clinical isolates readily accumulate changes in repetitive sequences as would be expected for proteins involved in host-pathogen interactions and possibly under selection pressures such as in the case of host immune responses directed against them [43,44,53,56]. In addition variations in repetitive sequence of surface proteins can also lead to important quantitative alterations of their functions, such as variation of adhesion properties to substrates as in cell-cell adhesions during parasite swarming (possibly induced by TvBspA-TvBspA interactions) with dramatic cases described in yeast involving surface proteins with different type of tandem repeats [70,71]. Variants in TpLRR could also lead to different functions altogether (such as binding to different substrates), and possibly contribute to rapid adaptations to environmental changes (as for example between the male and female urogential tracts or between the various mucosal landmarks) as known, or suggested, for different parasitic and other microbial eukaryotes [43,44,71,72]. It will be particularly interesting to compare in the future the extent of global variations of TvBspA TpLRR and non-LRR repeats between various clinical isolates to gain a better insight into their potential roles in evading host immune response and possible link between *T. vaginalis *genetic diversity and virulence [26]. As such, TvBspA-like genes could represent valuable markers for epidemiological studies to type clinical isolates [26].

Another interesting aspect of the diversity among TvBspA proteins with TMD was identified in two subfamilies with conserved CT. Each subfamily was characterised with a classic sorting signals in their CT with one possessing a di-leucine-like signal and the other an NPXY-like signal, both known to mediate rapid endocytosis in other eukaryotes [46]. In both cases the sequence and their position in the cytoplasmic tails suggested that these are functional [46]. The NPXY containing sequences in particular were characterised by flanking sequences FDNP [LIF]F (similar to FDNPVY of the human LDL receptors where the important tyrosine can be replace by a phenylalanine) known to form potent signals for endocytosis further supporting their functionality [46]. Hence these TvBspA proteins could represent receptors mediating endocytosis of various host (or others) proteins with their TpLRR likely involved in binding to ligands. These TvBspA proteins represent to our knowledge the first candidate receptor potentially mediating endocytosis of various host or other proteins.

Some TvBspA genes are potential pseudogenes with 98 entries currently being annotated as such as they possess ORF disrupting sequence features. In addition, other TvBspA sequences have sequencing ambiguities or are obviously truncated due to sequencing problems or missing data. However, very little is currently known about the structure-function relationship for TvBspA proteins and some of these annotated pseudogenes could actually correspond to functional genes such as TVAG_191490, which correspond to a C-terminal truncated form compared to a longer TvBspA-GRD. Hence some 'pseudogenes' could actually encode functional proteins (a shorter one in the case of TVAG_191490 when compared to close paralogues) or correspond to a reservoir used to generate TvBspA diversity through recombination or other processes facilitating the creation of new functional genes distinct from existing ones [73].

### Evidence for expression

For a total of over 270 TvBspA entries (~30% of all 911 TvBspA genes) we obtained evidence for transcription through EST, RT-PCR and microarray analyses. Differential expression was suggested from EST surveys and demonstrated for a subset of genes by microarray and RT-PCR analyses for two conditions, change in iron concentration and binding of parasite to ECM proteins, consistent with different functions for the analysed TvBspA. Due to the large gene family size a global approach based on microarrays designed to cover all 911 TvBspA genes combined with testing key stages of *T. vaginalis *infections of the urogenital tract of both sexes will be required in the future to gain further insight into the functional relevance of the vast TvBspA gene family. The microarray data presented here indicate that this approach will be an important one to contrast transcripts abundance.

The use of antisera directed against peptide derived from the TvBspA625 sequence demonstrated by IFA the expression of the proteins and its cell surface location, consistent with the bioinformatics analyses, indicating that the TpLRR and the P/NRR of TvBspA625 are exposed to extracellular milieu where they could mediate binding to host or other proteins as demonstrated for the bacterial proteins BspA and LrrA [29,31,33].

The same peptides used to raise the mouse anti-TvBspA625 antisera were also used in ELISA assays to test the presence of antibodies recognizing TvBspA625 from clinical patients. Our data strongly suggest that this protein is indeed expressed and triggers antibody responses in both sexes during the majority of tested *T. vaginalis *infections. Variations of the length of the P/NRR of TvBspA625 also suggested that the protein is under selective pressure possibly due to the immune response it stimulates. Contrasting the TvBspA625 sequences and their expression (along with all other TvBspA) between additional *T. vaginalis *clinical isolates from patients with and without an antibody response to this protein would be particularly interesting to further test this possibility. The patients without detected antibodies directed against TvBspA625 (~12% of *T. vaginalis *investigated patients) could correspond to *T. vaginalis *isolates that don't express that protein at all or with differences in the epitopes tested here. From these different considerations, we predict that differential expression of TvBspA gene sets combined with differences in the TpLRR and other repeats between TvBspA proteins will be identified between different clinical isolates.

Finally TvBspA could play important roles in regulating the innate immune responses in the urogential tracts, as demonstrated *in vitro *for *T. vaginalis *total cell surface proteins [74], since the BspA protein from *T. forsythia *was shown to regulate in a CD4 and TLR2 dependent manner cytokine induction [75]. The TpLRR of some TvBspA could be directly involved in binding the LRR of TLRs and induce specific innate response signalling.

## Conclusions

Considering the phenotypes of organisms encoding BspA-like proteins, the majority being pathogens, commensal or mutualists thriving on vertebrate mucosal surfaces; the established function of BspA-like proteins in the pathogenicity of two mouth mucosal bacteria (*T. forsythia *and *T. denticola*); the extraordinarily large TvBspA protein family size with its vast structural diversity, the differential expression patterns demonstrated for some TvBspA genes and the cell surface expression and induction of an antibody response during infections for one TvBspA protein; together strongly suggest that the TvBspA proteins play various and important roles in *T. vaginalis*' pathobiology by contributing to the invasion and long term infections of the human urogenital tract. TvBspA-like proteins represent strong candidate surface proteins mediating interaction with various mucosal landmarks including the mucus; VEC, urethra epithelial cell and other host cells; ECM proteins and vaginal microflora or cell-cell adhesion during parasite swarming. TvBspA could also mediate endocytosis of various host proteins and viruses, as well as underpin phagocytosis of bacteria and various host cells. Finally TvBspA proteins could orchestrate the modulation of the innate immune system through TLRs signalling during infection and mediate immune evasion through differential expression.

## Methods

### Genome data mining and other bioinformatic analyses

A Wu-BlastP (expectation value ≤ 0.001) search at TrichDB [76] with the TpLRR of TvBspA625 as query (TVAG_073760, XP_001321233, positions 1-420, [28]) identified 885 TvBspA candidate proteins. A PHI-Blast [77] (NCBI Blast server, with same query as for the BlastP, expect threshold ≤ 0.001, with the TpLRR pattern [LIV]xx [LIV]x [LIV]xxx [LIV]xx [LIV]xxxAFxx [CNST]xx) [30] followed by two iterations of PSI-Blast searches [78] recovered 908 *T. vaginalis *annotated proteins in RefSeq (20 August 2008) [79], which included all but 13 entries of the Wu-BlastP list and 26 additional sequences, defining a total of 911 distinct TvBspA candidate proteins. We also performed BlastP searched with all putative TvBspA proteins and investigated their respective Blast hit lists using SPyPhy to annotate them and investigate their level of similarity with the proteins they hit [80].

Profile based searches [81] were also used to investigate the presence of TpLRR. All entries that hit the LRR of BspA-like proteins from other taxa in the BlastP searches or were positive for the TpLRR Profile search (TpLRR profile accession: PS50505 http://www.isrec.isb-sib.ch/cgi-bin/get_pstprf?PS50505) or the TpLRR pattern were all considered as BspA-like candidate proteins and were hence named TvBspA.

The first 150 bp of the 5' upstream regions for all TvBspA-like genes were extracted from TrichDB to allow their comparisons and identify potential Inr sequences (consensus: [ATC]CA_+1 _[ATGC] [AT]) or TATA-box (consensus: TATA [AT]A [AT]) typically located in *T. vaginalis *genes within 30 bp or 50 bp, respectively, upstream of the translation initiation codon [36,82].

In order to identify candidate surface and secreted proteins the potential presence of SP (SignalP3.0 [83]) and TMD (TMHMM2.0 [84]) was investigate using the annotation and retrieval tool at TrichDB. These data were complemented with TMD identified with SPLIT4 [85] and PHOBIUS [61,86], with the latter also investigating the presence of SP. Proteins positive for SP with either SignalP3.0 or PHOBIUS where considered to possess a SP. The position of TMD and protein topology in the membrane was established using the consensus between the three methods TMHMM2.0, SPLIT4 and PHOBIUS and entries were annotated as TMD-CCT when at least two methods overlapped with the TMD position and agreed with the protein topology, as such consensus approaches can provide better predictions [87]. In order to investigate the potential presence of CAAX motif at the C-terminus in TvBspA-like proteins without TMD domains we used PrePS [63].

SAPS [88,89], RepSeq [43] and REPTILE [44] analyses were performed to investigate the potential presence of repeats and their features (in addition to the TpLRR) and low complexity segments among TvBspA-like proteins.

Protein alignments were performed with ClustalW2.0.9 [90] for all or selected subsets of TvBspA sequences and took advantage of the iteration process (iteration on the final alignment for all 911 TvBspA or for each step for reduced set of sequences) that allow improvement of the alignment quality. Clustal was also used to generate table of protein pairwise sequence identity (%). SEAVIEW [91] was used to view, edit and manipulate the alignments for figure preparation.

To identify potential protein domains and functional sites the TvBspA proteins were analyses with InterProScan [92].

### Cell culturing

The *T. vaginalis *clinical isolate G3, for which a draft genome was published [36], and the clinical isolate SS-22 [45] were used for most of the molecular cell biology experimental work. In addition other isolates described below were used for EST libraries and microarray analyses. Cells were grown axenically at 37°C in Modified Diamond Medium [93] without agar, supplemented with 10%(v/v) heat-inactivated horse serum (Gibco, Invitrogen) and 50,000 units/l penicillin and 50 mg/l streptomycin (Penicillin-Streptomycin Solution, Sigma).

### EST libraries

Total RNAs were isolated from different culture conditions indicated below. cDNAs primed with oligo-dT were synthesized by using a ZAP-cDNA synthesis kit and directionally cloned into the EcoRI and XhoI sites of Uni-ZAP XR (Stratagene) vector. The quality of the unidirectional cDNA library was assessed by colony PCR of 96 randomly picked clones to determine the average insert size and percentage of clones without inserts. Then 384 randomly picked clones were sequenced to determine the percentage of vector contamination, valid average length and redundancy of the cDNA library. Plasmids were in-vivo excised from the cDNA library by using helper phage and transformed into *E. coli *DH10B (Invitrogen) as described by the manufacturer (Stratagene). Around 10,000 ESTs were randomly picked from each cDNA library which represents different stages of cell cycle, pathogenesis and specific nutrient requirements [94]. The five culture conditions used are described below:

Condition [1]: TvEST Library (10,749 ESTs), *T. vaginalis *isolate ATCC30236 (JH 31A#4).

Medium: YIS, pH 6.0 supplemented with 100 μM ferric ammonium citrate. Growth condition: unsynchronized culture harvested at mid-log phase, trophozoites forms.

Condition [2]: TvG Library (10,470 ESTs), *T. vaginalis *isolate: ATCC30001 (C-1:NIH). Medium: YIS, pH 6.0 supplemented with 100 μM ferric ammonium citrate. Growth condition: mid-log phase culture cold stressed for 4 hours at 4°C, then return to 37°C for 6 hours, synchronised G2 phase trophozoites forms.

Condition [3]: TvCS Library (9,865 ESTs), *T. vaginalis *isolate ATCC30001 (C-1:NIH).

Medium: YIS, pH 6.0 supplemented with 100 μM ferric ammonium citrate. Growth condition: Mid-log phase culture cold stressed for 4 hours at 4°C, pseudocyst forms.

Condition [4]: TvFN Library (9,955 ESTs), *T. vaginalis *isolate isolate TO16. Medium: DMEM:TYM. Growth condition: parasites were grown in fibronectin coated T-75 flask for 3 hours at 37°C, amoeboid forms.

Condition [5]: TvLI Library (10,505 ESTs), *T. vaginalis *isolate T1. Medium: TYM, pH 6.2. Growth condition: parasites were grown in culture medium supplemented with 80 μM Dipyridyl (DIP) for 10 passages at 37°C, trophozoite forms.

We also took advantage of the ~24,000 EST stored in GenBank dbEST that include those generated by TIGR [36] and our 4003 in house EST generated from *T. vaginalis *(G3) trophozoites (mid log-phase). The latter were deposited in dbEST with the accession numbers http://www.ncbi.nlm.nih.gov/nucest?term=Harriman_N%20Hirt_RP%20trichomonas%20vaginalis.

### Microarray and quantitate RT-PCR

Corning^® ^UltraGAPST coated slides were spotted with cDNA derived from the ~70,000 EST characterised from the various growth condition described above. A total of 7,680 cDNA (4,938 distinct entries) were successfully amplified by PCR from plasmids and spotted on the array once, twice or in triplicate. A total of 8 arrays were used to contrast expression levels - dye-swap experimental design for four independent experiments. *T. vaginalis *cells were grown in two conditions contrasting high iron (TYM medium supplemented with 150 μM iron nitrilotriacetate) and low iron (TYM medium with 80 μM DIP) and grown for 10 passages in the respective conditions. Total RNA was extracted using QuickPrep Total RNA Extraction Kit (Amersham Biosciences) and cleaned up using Rneasy CleanUp Kit (Quiagen). Total RNA concentration and purity was determined using a NanoDrop ND-1000 spectrophotometer (NanoDrop Technologies). The cDNA probes were synthesized from 2 μg of total RNA using primers contained in 3DNA Array 900 Expression Array Detection Kit (Genisphere). The hybridization was carried out following 3DNA Array 900 Expression Array Detection Kit (Genisphere) protocol. The signal was transformed with natural log (ln) and normalized by LOWESS normalization method in the TIGR microarray data analysis system (MIDAS) version 2.19 [95]. Entries with p values <1e-3 were considered to have significant differences in transcript concentration. Mean and standard deviation for the 13 TvBspA genes with significant difference in level of expression and appropriate control genes are listed in Table 4. qRT-PCR of selected genes was used to validate the microarray data and included three TvBspA genes and 14 control unrelated genes (unpublished data) two of which are listed in Table 4. All primers used for semi-quantitative RT-PCR and qRT-PCR are listed in additional file 19, Table S12. All tested genes by qRT-PCR were in qualitative agreement with the microarray data. The Microarray data were deposited into ArrayExpress with the accession number E-MTAB-126.

### Extracellular matrix binding assay and semi-quantitative RT-PCR

Culture cell flasks (25 cm^2^, Greiner Bio-One) were coated with a PBS solution containing 500 μg/ml Collagen I (rat tail, Marathon Lab), 40 μg/ml laminin (Engelbreth-Holm-Swarm murine sarcoma, basement membrane, Sigma), 50 μg/ml fibronectin (human plasma, Sigma) and 50 μg/ml phenol red (Sigma) as pH indicator. The solidification of the coating solution in a gel was obtained by incubation at 37°C for 10 min and coated flasks were stored at room temperature. The resulting gel matrix was an ultra-thin pink coloured layer of proteins homogenously covering the entire bottom surface of the flask. *Trichomonas vaginalis *cells used for binding assays were harvested from mid-log phase growth (~24 hrs) by centrifugation at 750 × g min^-1 ^and washed twice in minimal binding buffer (MBB) [96]. Parasites were counted using a Neubauer Haemocytometer and coated flasks were seeded with 5 × 10^6 ^cells in 5 ml of MBB and incubation carried out at 37°C for 60 min. Amoeboid shaped cells could be observed upon binding to the substrate after ~10 min. After 60 min, flasks were washed twice with warm MBB in order to remove unbound cells and the majority of cells were tightly bound to the substrate and showed a large proportion of amoeba forms or had pseudopodial-like cellular extensions. The population of cells were diverse in their morphology and movements with some cells actively roaming the substrate (including some with long pseudopodia up ~1/3 of the length of the cell), whereas others were static.

Expression of selected TvBspA-like protein coding genes was analysed by semi-quantitative RT-PCR contrasting ECM bound cells and trophozoites not exposed to the substrate. Total RNA extractions were performed in parallel on both ECM bound cells and trophozoites not exposed to the substrate, using SV Total RNA Isolation System (Promega) according to manufacturer specifications (that includes a DNAase treatment step to insure the absence of any genomic DNA) and total RNA were quantified by absorbance at 260 nm. The polyA+ mRNA were then purified using Dynabeads^® ^mRNA Purification Kit (Dynal Biotech) and The RT-PCR THERMOSCRIPT Kit (Invitrogen) was used for the RT-PCR reactions with specific primer pairs previously tested on genomic DNA - all producing the expected size amplicons without detectable background. The same amounts of cDNA from either ECM bound cells or trophozoites were used for each the PCR reactions. The amount of cDNA used for bound cells and trophozoites was normalized based on the total RNA concentrations since the smaller amount of cDNA obtained from the ECM proteins bound cells did not allow its quantification. Controls RT-PCRs consisted of actin (loading control) and alpha-actinin (up-regulated gene upon amoeba transformation) amplifications using specific primers previously described [97]. These procedures were carried out in five independent binding assays and independent PCR reactions (at least three per binding assays) and gave similar expression patterns for the tested genes. All primers are listed in additional file 19, Table S12. PCR reactions were run on 1% agarose gel for analysis.

### Peptide synthesis and mouse anti-peptide antisera

Four peptides were designed from the TvBspA625 sequence [28] and their sequence features to optimized peptide synthesis, solubility and antigenicity and to differentiate TvBspA625 from other *Trichomonas *proteins. Two peptides sequences were derived from the inferred extracellular domain and two peptides from the cytoplasmic tail (Figure 2). BlastP with all four peptides as query established that three peptides are likely to generate TvBspA625 specific antisera. One TvBspA625 peptide (EXT-1) could possibly generate antisera cross-reacting with TvBspA805 or be recognised by patients antibodies directed against TvBspA805 as a stretch of 10 identical contiguous residues was shared between TvBspA625 and TvBspA805. The four peptides were synthesised by adding a N-terminal cysteine, to allow cross-linking with the maleimide-activated carrier proteins. Peptides were coupled to both keyhole limpet hemocyanin (KLH) and bovine serum albumine (BSA) by using the Imject Maleimide Activated Immunogen kit (Pierce, Rockford, IL, USA), following the manufacturer instruction. Immunization of eight BALB/c mice (two mice/peptide) five weeks old was performed by both subcutaneous and intraperitoneal inoculation of 20 μg of KHL conjugated peptide proteins in complete Freund's adjuvant per inoculum. Mice were inoculated three times with an interval of 10 days, and sacrificed six days after the final intravenous boost with 10 μg of BSA-coupled peptides. Sera were collected and specific reactivity against each peptide was tested by ELISA using plates coated with KLH and BSA coupled peptides. The antisera titre ranged from 1:100,000 to 1:500,000. Presera and sera were collected and used for indirect immunofluorescence and Western blot analyses using indicated dilutions.

### Western blot analyses

Total cell extracts from *T. vaginalis *cell cutlures were submitted to SDS-PAGE and immunoblotting as previously described [98]. Briefly, 3 × 10^5 ^washed cells from exponentially growing cultures were resuspended in 100 μl of Laemmli lysis buffer and boiled for 3 min. 10 μl of each sample were then loaded in each well of a 7.5% SDS-PAGE gel, electrophoresed, blotted onto nitrocellulose, blocked, and separately incubated with the mouse anti-peptide sera at 1: 2000 dilution. After washing, membranes were incubated with a goat anti-mouse immunoglobulin sera, conjugated with alkaline phosphatase (Sigma, S. Louis, USA). Bound antibodies were detected by soaking the nitrocellulose membrane in AP buffer (0.1 M tris pH 9.5, 0.1 M NaCl, 0.005 M MgCl2) to which 0.33 mg/ml nitroblue tetrazolium (NBT), and 0.165 mg/ml 5-bromo-4-chloro-3-indolyl phosphate (BCIP) were added.

### Indirect immunofluorescence analyses (IFA)

Trophozoites of *T. vaginalis *grown *in vitro *were collected during the exponential growth phase at 350 × g for 5 min and washed twice with Ringer (NaCl 0.12 M, KCl 3.5 mM, CaCl2 2 mM, NaHCO3 2.5 mM, pH 7.2) and fixed with either 3% formaldehyde (Sigma) 10 min at room temperature or 70% ethanol at -20°C, 20 min. Fixed cells were pelleted by centrifugation at 250 × g for 10 min at 4°C, washed twice with PBS pH 7.2, and allowed to adhere to poly-L-lysine coated slides for 1 h. Formaldehyde fixed cells, once bound, were further incubated with 50 mM NH_4_Cl to quench protein side groups exposed by this fixation procedure and then washed twice in PBS. Slides were incubated with blocking buffer (3% bovine serum albumin in PBS) for 30 min before incubation with primary mouse anti-peptide antisera over night at 4°C. As control we used VG2, a mouse anti-*T. vaginalis*-α-tubulin monoclonal antibody [50] and a rabbit anti-*T. vaginalis*-hydrogenosomal malic enzyme antisera [49]. After three washes of 10 min each with PBS, the slides were incubated with both Alexa-Fluor-488 conjugated goat anti-mouse IgGs and Alexa-Fluor-594 conjugated goat anti-rabbit IgGs (both from Invitrogen, Molecular Probes) diluted 1:200 for 1 h at 37°C. After rinsing three times with PBS, the coverslips were mounted with VECTASHIELD^® ^Mounting Medium with DAPI (Vector Laboratories, UK) prior microscopic observation with a confocal laser scanning microscopy Leica TCS SP2UV. Images were captured and processed using Leica CS Lite program version 2.61.

### ELISA assays to measure human patients immune response against TvBspA625 peptides

Sera from a total of 591 humans with high risk of sexually transmitted diseases were selected and kept frozen at -20°C until enzyme-linked immunosorbent assay (ELISA) experiments; 356 sera were from female patients, while 235 from males. Consent was obtained from the patients and the material was databased and processed anonymously. ELISA were performed following a published method [99]. Cells from *T. vaginalis *isolate SS-22 were used as the source of total antigen since it is characterised by a low degree of phenotypic variation and is not infected by *Mycoplasma hominis*. Parasites (viability 99%) were resuspended at the density of 1 × 10^6^/ml in phosphate-buffered saline (PBS), and 50 μl of *T. vaginalis *suspension were seeded in each well of PVC microtiter-well plates (Becton Dickinson, Lincoln Park, NJ) and allowed to dry. 50 μl of ice-cold 95% ethanol were added to each well and allowed to dry, then washed in distilled water, and stored at 4°C until use. Prior to use, wells were pretreated for 2 hours with a PBS-0.05% Tween 20 (PBS-T) solution containing 5% nonfat dry milk. 100 μl of sera diluted 1:200 in the same solution were then added and incubated for two hours at room temperature. After extensive rinsing with PBS-T, 100 μl of goat anti-human IgG or IgM antibodies conjugated with alkaline phosphatase (Sigma, St. Louis) were added. After two hours, the color reaction was induced with specific substrates and absorbance measured. Cutoff was established as twice the mean value obtained with sera from 10 healthy male volunteers distinct from the 591 tested humans. Sera were classified as negative if the ELISA readings were lower than the cutoff value, or positive if at least twice above the cutoff value.

In order to evaluate the immunogenicity of TvBspA625 protein during *T. vaginalis *infections, the presence of specific antibodies against the four immunogenic peptides were tested by ELISA in 161 human sera positive for *T. vaginalis *total proteins, as defined above. In addition, 61 sera negative for *T. vaginalis *total proteins were used for comparisons. ELISA plates were coated over night separately with 1 μg of BSA or KLH-linked peptide in 50 μl carbonate buffer, pH 8,6. Plates were then washed with PBS 0.05% Tween 20 (PBS-T) and saturated with BPS-T containing 5% nonfat dry milk. All sera were diluted 1:200 and separately tested for reactivity against each peptide and immune complexes detected as described above. Statistical tests on the ELISA data (2-way contingency table, Pearson chi-square test) were performed at [100].

## Authors' contributions

CJN performed the ECM binding assays, RT-PCR, IFA, RT-PCR and sequencing on isolate SS-22 and analysed the data. ND performed the characterisation of anti-TvBspA625 peptide mouse sera, the testing of the immunoreactivity of human sera against *T. vaginalis *total extracts and TvBspA625 peptides, and RT-PCR on the SS-22 isolate. TSP performed the SPyPhy and InterProScan analyses, profile search and wrote specific scripts for additional bioinformatics analyses. LS performed the microarray experiments and qRT-PCR. PT designed and supervised the EST experiments and designed the microarrays and performed the analyses derived from them. JT and PT conceived and designed the microarray experiments and performed data analyses. PLF conceived and designed the production and characterisation of anti-BspA-625 peptide sera; immunoreactivity of human sera and data analyses and interpretation. RPH designed the overall project and supervised it on a day-to-day basis, performed several of the bioinformatics analyses and drafted the manuscript. All authors read and approved the final manuscript.

## Supplementary Material

Additional file 1 **Supplemental Table S1.** Annotation of all 911 TvBspA proteins. Table listing all 911 TvBspA-like entries (proteins and genes features are in distinct worksheets) with locus tags, accession numbers, CLUSS2 subfamilies and ClustalW alignment positions, genome position, identified sequence features (SP, TMD, TpLRR position), number of EST per entry, RT-PCR/microarray data, promoter features and other annotations. Click here for file

Additional file 2 **Supplemental Table S2. **PHI-BlastP taxonomic report for proteins with TpLRR. Full taxonomic report of PHI-Blast search on NCBI RefSeq protein database. In html format to be open in a web browser. Click here for file

Additional file 3 **Supplemental Table S3.** PHI-/PSI-Blast taxonomic report for proteins with TpLRR from *Trichomonas vaginalis *and *Entamoeba histolytica*. Full taxonomic report of PHI/PSI-BlastP searches on NCBI RefSeq protein database. In html format to be open in a web browser. Click here for file

Additional file 4 **Supplemental Table S4.** BlastP hit list for all 911 TvBspA. Table listing the top BlastP hit for each TvBspA (other TvBspA, other eukaryote, Bacteria and Archaea). Click here for file

Additional file 5 **Supplemental Table S5.** Example of *T. vaginalis *proteins with LRR distinct from TpLRR. Table listing the accession numbers of selected *T. vaginalis *proteins with LRR distinct from the TpLRR and taxa encoding related proteins. Click here for file

Additional file 6 **Supplemental Figure S1.ClustalW alignment of all 911 TvBspA proteins**. Protein alignment in clustal format to be open in an alignment editor such as SEAVIEW. Click here for file

Additional file 7 **Supplemental Figure S2.** ClustalW alignment of 193 TvBspA proteins with TMD-CCT. Protein alignment in clustal format to be open in an alignment editor such as SEAVIEW. Click here for file

Additional file 8 **Supplemental Figure S3. **Overview of scaffold with 18 TvBspA genes. Figure illustrating the gene content of contig DS113361 and highlighting the positions of 18 TvBspA genes. Click here for file

Additional file 9 **Supplemental Table S6.PHI-/PSI-Blast taxonomic report for Bacteria and Achaea**. Full taxonomic report of PHI/PSI-BlastP searches on NCBI RefSeq protein database. In html format to be open in a web browser. Click here for file

Additional file 10 **Supplemental Table S7.** The TvBspA with extra non-TpLRR repeats. Table listing the 35 TvBspA entries with additional non-TpLRR repeats identified by REPTILE and/or RepSeq. Click here for file

Additional file 11 **Supplemental Figure S4.** Alignment of TpLRR and other repeats for selected TvBspA proteins. Combination of manual and SAPS based alignment of repeats for TpLRR, and other repeats when present. Included selected TvBspA proteins with well conserved and less well conserved TpLRR illustrating their diversity. Click here for file

Additional file 12 **Supplemental Figure S5**. Alignment of the GRD from TvBspA-GRD and proteins with related GRD. Clustal alignments of the 12 TvBspA-GRD with nine proteins from RefSeq with related GRD - screen shots from SEAVIEW. Click here for file

Additional file 13 **Supplemental Table S8. **BlastP taxonomic report for proteins with GRD. Full taxonomic report of BlastP search on NCBI RefSeq protein database. In html format to be open in a web browser. Click here for file

Additional file 14 **Supplemental Table S9.** Summary of all data on transcribed *T. vaginalis *genes. Table listing EST, microarray and semi-quantitative RT-PCR data obtained for various *T. vaginalis *isolates. Click here for file

Additional file 15 **Supplemental Table S10. **TvBspA ESTs frequency table. Frequency table for TvBspA ESTs, including in relation to TvBspA structural organisation. Click here for file

Additional file 16 **Supplemental Table S11.** Summary annotation for the TvBspA analysed by semi-quantitative RT-PCR. Table listing locus tag, and selected protein features and annotations and RT-PCR results of the nine TvBspA analyses upon binding of the parasites to ECM proteins *in vitro*. Click here for file

Additional file 17 **Supplemental Figure S6.** Western blot analyses with mouse anti-TvBspA625 peptide antisera on *T. vaginalis *total proteins extracts. Click here for file

Additional file 18 **Supplemental Figure S7.** Indirect immunofluorescence analyses with anti-TvBspA625 peptide antisera on *T. vaginalis *fixed with formaldehyde. Click here for file

Additional file 19 **Supplemental Table S12.** List of primers used for RT-PCR and qRT-PCR. Click here for file
