# Supplementary material for: Trichomonas vaginalis vast BspA-like gene family: evidence for functional diversity from structural organisation and transcriptomics
Source: BMC Genomics. 2010 Feb 8;11:99. doi: 10.1186/1471-2164-11-99 (PMC2843621; doi:10.1186/1471-2164-11-99)
Supplement: Additional file 2 — Supplemental Table S2. PHI-BlastP taxonomic report for proteins with TpLRR. Full taxonomic report of PHI-Blast search on NCBI RefSeq protein database. In html format to be open in a web browser. [file 1471-2164-11-99-S2.HTML]

### Table S2. Taxonomic report for PHI-BLAST search on RefSeq

---


### Pattern: [LIV]xx[LIV]x[LIV]xxx[LIV]xx[LIV]xxxAFxx[CNST]xx

### e-value <= 0.001

---

**Index**

- Lineage Report
- Organism Report
- Taxonomy Report
- Help

**Lineage Report**  

```
cellular organisms
. Eukaryota          [eukaryotes]
. . Trichomonas vaginalis G3 --------------------------  774 1570 hits [trichomonads]        surface antigen BspA-like [Trichomonas vaginalis G3]
. . Entamoeba dispar SAW760 ...........................  205   92 hits [eukaryotes]          hypothetical protein, conserved [Entamoeba dispar SAW760]
. . Entamoeba histolytica HM-1:IMSS ...................  143   67 hits [eukaryotes]          leucine rich repeat protein, BspA family [Entamoeba histoly
. Methanosarcina barkeri str. Fusaro ------------------  251   28 hits [euryarchaeotes]      cell surface protein [Methanosarcina barkeri str. Fusaro]
. Eubacterium siraeum DSM 15702 .......................  249   54 hits [firmicutes]          hypothetical protein EUBSIR_02655 [Eubacterium siraeum DSM 
. Methanosarcina acetivorans C2A ......................  244   39 hits [euryarchaeotes]      cell surface protein [Methanosarcina acetivorans C2A]
. Flavobacterium psychrophilum JIP02/86 ...............  236   56 hits [CFB group bacteria]  cell surface leucine-rich repeat-containing protein [Flavob
. Clostridium leptum DSM 753 ..........................  229   14 hits [firmicutes]          hypothetical protein CLOLEP_03013 [Clostridium leptum DSM 7
. bacterium Ellin514 ..................................  199   16 hits [verrucomicrobia]     cell surface protein [bacterium Ellin514]
. Syntrophomonas wolfei subsp. wolfei str. Goettingen .  193    9 hits [firmicutes]          leucine-rich repeat-containing protein [Syntrophomonas wolf
. Clostridium spiroforme DSM 1552 .....................  178    4 hits [firmicutes]          hypothetical protein CLOSPI_02317 [Clostridium spiroforme D
. Clostridium beijerinckii NCIMB 8052 .................  174   15 hits [firmicutes]          cell wall binding repeat-containing protein [Clostridium be
. Epulopiscium sp. 'N.t. morphotype B' ................  174   73 hits [firmicutes]          cell surface protein [Epulopiscium sp. 'N.t. morphotype B']
. Victivallis vadensis ATCC BAA-548 ...................  167   12 hits [bacteria]            cell surface protein [Victivallis vadensis ATCC BAA-548]
. Shewanella pealeana ATCC 700345 .....................  155   26 hits [g-proteobacteria]    FNIP [Shewanella pealeana ATCC 700345]
. Anaerofustis stercorihominis DSM 17244 ..............  153   46 hits [firmicutes]          hypothetical protein ANASTE_00419 [Anaerofustis stercorihom
. Bacteroides fragilis NCTC 9343 ......................  148    4 hits [CFB group bacteria]  surface protein [Bacteroides fragilis NCTC 9343]
. Treponema denticola ATCC 35405 ......................  146   25 hits [spirochetes]         leucine rich repeat domain-containing protein [Treponema de
. Alistipes putredinis DSM 17216 ......................  146   19 hits [CFB group bacteria]  hypothetical protein ALIPUT_02273 [Alistipes putredinis DSM
. Synechococcus sp. WH 7805 ...........................  141   21 hits [cyanobacteria]       cell surface protein [Synechococcus sp. WH 7805]
. Ruminococcus torques ATCC 27756 .....................  138   18 hits [firmicutes]          hypothetical protein RUMTOR_02627 [Ruminococcus torques ATC
. Bacteroides ovatus ATCC 8483 ........................  134   12 hits [CFB group bacteria]  hypothetical protein BACOVA_04585 [Bacteroides ovatus ATCC 
. Methanococcus vannielii SB ..........................  126   12 hits [euryarchaeotes]      cell surface protein [Methanococcus vannielii SB]
. Clostridium sp. L2-50 ...............................  117   13 hits [firmicutes]          hypothetical protein CLOL250_02338 [Clostridium sp. L2-50]
. Clostridium butyricum 5521 ..........................  112    6 hits [firmicutes]          surface protein PspC [Clostridium butyricum 5521]
. Coprococcus eutactus ATCC 27759 .....................  107    6 hits [firmicutes]          hypothetical protein COPEUT_00473 [Coprococcus eutactus ATC
. Photobacterium sp. SKA34 ............................  104    7 hits [g-proteobacteria]    cell surface protein [Photobacterium sp. SKA34]
. Kordia algicida OT-1 ................................  103   10 hits [CFB group bacteria]  cell surface protein [Kordia algicida OT-1]
. Flavobacteriales bacterium ALC-1 ....................  101    9 hits [CFB group bacteria]  cell surface protein [Flavobacteriales bacterium ALC-1]
. Methanococcus maripaludis C7 ........................  100   16 hits [euryarchaeotes]      TPR repeat-containing protein [Methanococcus maripaludis C7]
. Bacteroides stercoris ATCC 43183 ....................   99    6 hits [CFB group bacteria]  hypothetical protein BACSTE_00622 [Bacteroides stercoris AT
. Clostridium phytofermentans ISDg ....................   99    9 hits [firmicutes]          hypothetical protein Cphy_3373 [Clostridium phytofermentans
. Clostridium bartlettii DSM 16795 ....................   96    6 hits [firmicutes]          hypothetical protein CLOBAR_00617 [Clostridium bartlettii D
. Desulfitobacterium hafniense Y51 ....................   91    9 hits [firmicutes]          hypothetical protein DSY3856 [Desulfitobacterium hafniense 
. Eubacterium ventriosum ATCC 27560 ...................   88   13 hits [firmicutes]          hypothetical protein EUBVEN_01340 [Eubacterium ventriosum A
. Shewanella halifaxensis HAW-EB4 .....................   83    5 hits [g-proteobacteria]    hypothetical protein Shal_3565 [Shewanella halifaxensis HAW
. Treponema pallidum subsp. pallidum str. Nichols .....   82    3 hits [spirochetes]         leucine-rich repeat-containing protein [Treponema pallidum 
. Treponema pallidum subsp. pallidum SS14 .............   82    3 hits [spirochetes]         leucine-rich repeat-containing protein [Treponema pallidum 
. Dorea formicigenerans ATCC 27755 ....................   80    4 hits [firmicutes]          hypothetical protein DORFOR_02330 [Dorea formicigenerans AT
. Clostridium scindens ATCC 35704 .....................   78   12 hits [firmicutes]          hypothetical protein CLOSCI_03447 [Clostridium scindens ATC
. Bacteroides fragilis YCH46 ..........................   77    1 hit  [CFB group bacteria]  putative cell surface antigen [Bacteroides fragilis YCH46]
. Bacteroides coprocola DSM 17136 .....................   73    3 hits [CFB group bacteria]  hypothetical protein BACCOP_00841 [Bacteroides coprocola DS
. Clostridium botulinum A3 str. Loch Maree ............   73   12 hits [firmicutes]          putative cell surface protein [Clostridium botulinum A3 str
. Clostridium sporogenes ATCC 15579 ...................   73   18 hits [firmicutes]          hypothetical protein CLOSPO_00560 [Clostridium sporogenes A
. Clostridium botulinum B1 str. Okra ..................   73   12 hits [firmicutes]          putative cell surface protein [Clostridium botulinum B1 str
. Clostridium botulinum Bf ............................   72   13 hits [firmicutes]          putative cell surface protein [Clostridium botulinum Bf]
. Methanococcus maripaludis C6 ........................   72    7 hits [euryarchaeotes]      hypothetical protein MmarC6_0234 [Methanococcus maripaludis
. Clostridium botulinum A str. ATCC 3502 ..............   71   13 hits [firmicutes]          cell surface protein [Clostridium botulinum A str. ATCC 350
. Clostridium botulinum NCTC 2916 .....................   71   13 hits [firmicutes]          putative cell surface protein [Clostridium botulinum NCTC 2
. Clostridium botulinum F str. Langeland ..............   71   18 hits [firmicutes]          putative cell surface protein [Clostridium botulinum F str.
. Bacteroides vulgatus ATCC 8482 ......................   68    2 hits [CFB group bacteria]  hypothetical protein BVU_3191 [Bacteroides vulgatus ATCC 84
. Ruminococcus obeum ATCC 29174 .......................   60    7 hits [firmicutes]          hypothetical protein RUMOBE_01418 [Ruminococcus obeum ATCC 
. Bacteroides thetaiotaomicron VPI-5482 ...............   59    4 hits [CFB group bacteria]  putative cell surface antigen [Bacteroides thetaiotaomicron
. Bacteroides caccae ATCC 43185 .......................   59    4 hits [CFB group bacteria]  hypothetical protein BACCAC_03700 [Bacteroides caccae ATCC 
. Streptococcus pneumoniae SP195 ......................   55    2 hits [firmicutes]          choline binding protein PcpA [Streptococcus pneumoniae SP19
. Streptococcus pneumoniae CGSP14 .....................   54    2 hits [firmicutes]          choline binding protein PcpA [Streptococcus pneumoniae CGSP
. Streptococcus pneumoniae CDC0288-04 .................   54    2 hits [firmicutes]          choline binding protein PcpA [Streptococcus pneumoniae CDC0
. Streptococcus pneumoniae CDC3059-06 .................   54    2 hits [firmicutes]          choline binding protein PcpA [Streptococcus pneumoniae CDC3
. Actinomyces odontolyticus ATCC 17982 ................   54    5 hits [high GC Gram+]       hypothetical protein ACTODO_00093 [Actinomyces odontolyticu
. Streptococcus pneumoniae SP23-BS72 ..................   54    2 hits [firmicutes]          choline binding protein PcpA [Streptococcus pneumoniae SP23
. Streptococcus pneumoniae SP6-BS73 ...................   54    2 hits [firmicutes]          choline binding protein PcpA [Streptococcus pneumoniae SP6-
. Streptococcus pneumoniae SP19-BS75 ..................   54    2 hits [firmicutes]          choline binding protein PcpA [Streptococcus pneumoniae SP19
. Streptococcus pneumoniae CDC1873-00 .................   54    2 hits [firmicutes]          choline binding protein PcpA [Streptococcus pneumoniae SP19
. Streptococcus pneumoniae CDC1087-00 .................   54    2 hits [firmicutes]          choline binding protein PcpA [Streptococcus pneumoniae SP19
. Streptococcus pneumoniae Hungary19A-6 ...............   54    2 hits [firmicutes]          choline binding protein PcpA [Streptococcus pneumoniae SP19
. Streptococcus pneumoniae SP18-BS74 ..................   54    2 hits [firmicutes]          ornithine carbamoyltransferase [Streptococcus pneumoniae SP
. Streptococcus pneumoniae SP11-BS70 ..................   54    2 hits [firmicutes]          ornithine carbamoyltransferase [Streptococcus pneumoniae SP
. Streptococcus pneumoniae MLV-016 ....................   54    2 hits [firmicutes]          ornithine carbamoyltransferase [Streptococcus pneumoniae SP
. Streptococcus pneumoniae TIGR4 ......................   54    4 hits [firmicutes]          hypothetical protein SpneT_02001904 [Streptococcus pneumoni
. Streptococcus pneumoniae G54 ........................   52    2 hits [firmicutes]          choline binding protein PcpA [Streptococcus pneumoniae G54]
. Acholeplasma laidlawii PG-8A ........................   52    2 hits [mycoplasmas]         putative surface-anchored antigen, BspA-like protein [Achol
. Lactococcus lactis subsp. lactis Il1403 .............   51    3 hits [firmicutes]          hypothetical protein L58460 [Lactococcus lactis subsp. lact
. Streptococcus pneumoniae D39 ........................   50    1 hit  [firmicutes]          choline binding protein PcpA [Streptococcus pneumoniae D39]
. Streptococcus pneumoniae R6 .........................   50    1 hit  [firmicutes]          choline binding protein PcpA [Streptococcus pneumoniae R6]
. Fusobacterium nucleatum subsp. nucleatum ATCC 25586 .   49    3 hits [fusobacteria]        surface antigen [Fusobacterium nucleatum subsp. nucleatum A
. Lactococcus lactis subsp. cremoris SK11 .............   48    2 hits [firmicutes]          subtilisin-like serine protease [Lactococcus lactis subsp. 
. Lactococcus lactis subsp. cremoris MG1363 ...........   47    2 hits [firmicutes]          putative secreted protein [Lactococcus lactis subsp. cremor
. Lactobacillus casei BL23 ............................   46    5 hits [firmicutes]          Putative uncharacterized protein [Lactobacillus casei]
. Clostridium sp. SS2/1 ...............................   43    4 hits [firmicutes]          hypothetical protein CLOSS21_02635 [Clostridium sp. SS2/1]
. Faecalibacterium prausnitzii M21/2 ..................   43    3 hits [firmicutes]          hypothetical protein FAEPRAM212_02550 [Faecalibacterium pra
. Lactobacillus casei ATCC 334 ........................   42    3 hits [firmicutes]          adhesion exoprotein [Lactobacillus casei ATCC 334]
. Mycoplasma agalactiae PG2 ...........................   39    1 hit  [mycoplasmas]         lipoprotein [Mycoplasma agalactiae PG2]
. Bacteroides capillosus ATCC 29799 ...................   38    2 hits [CFB group bacteria]  hypothetical protein BACCAP_00569 [Bacteroides capillosus A
. Streptococcus equi subsp. zooepidemicus MGCS10565 ...   37    3 hits [firmicutes]          cell surface protein, RBC-binding protein [Streptococcus eq
. candidate division TM7 single-cell isolate TM7b .....   33    1 hit  [bacteria]            Probable cell surface protein (Leucine-rich repeat protein)
. Streptococcus agalactiae CJB111 .....................   33    2 hits [firmicutes]          reticulocyte binding protein [Streptococcus agalactiae CJB1
. Streptococcus agalactiae A909 .......................   32    1 hit  [firmicutes]          hypothetical protein SAK_0502 [Streptococcus agalactiae A90
. Streptococcus agalactiae NEM316 .....................   32    1 hit  [firmicutes]          hypothetical protein gbs0456 [Streptococcus agalactiae NEM3
. Streptococcus agalactiae H36B .......................   32    1 hit  [firmicutes]          reticulocyte binding protein [Streptococcus agalactiae H36B]
. Streptococcus agalactiae 515 ........................   32    1 hit  [firmicutes]          reticulocyte binding protein [Streptococcus agalactiae 515]
. Streptococcus agalactiae COH1 .......................   32    1 hit  [firmicutes]          reticulocyte binding protein [Streptococcus agalactiae COH1]
. Streptococcus agalactiae 2603V/R ....................   32    1 hit  [firmicutes]          hypothetical protein SAG0421 [Streptococcus agalactiae 2603
. Streptococcus agalactiae 18RS21 .....................   32    1 hit  [firmicutes]          hypothetical protein SAG0421 [Streptococcus agalactiae 2603
. Streptococcus pneumoniae SP14-BS69 ..................   30    1 hit  [firmicutes]          choline binding protein PcpA [Streptococcus pneumoniae SP14
. Fusobacterium nucleatum subsp. vincentii ATCC 49256 .   30    1 hit  [fusobacteria]        Hypothetical Cytosolic Protein [Fusobacterium nucleatum sub
. Anaerotruncus colihominis DSM 17241 .................   29    1 hit  [firmicutes]          hypothetical protein ANACOL_02805 [Anaerotruncus colihomini
. Streptococcus pneumoniae SP9-BS68 ...................   28    2 hits [firmicutes]          choline binding protein PcpA [Streptococcus pneumoniae SP9-
. Finegoldia magna ATCC 29328 .........................   28    3 hits [firmicutes]          putative chimeric erythrocyte-binding protein [Finegoldia m
. Listeria monocytogenes FSL J2-064 ...................   28    1 hit  [firmicutes]          cell wall surface anchor family protein [Listeria monocytog
. Listeria monocytogenes FSL J1-175 ...................   28    1 hit  [firmicutes]          cell wall surface anchor family protein [Listeria monocytog
. Listeria monocytogenes FSL R2-503 ...................   28    1 hit  [firmicutes]          cell wall surface anchor family protein [Listeria monocytog
. Listeria monocytogenes FSL J1-194 ...................   28    1 hit  [firmicutes]          cell wall surface anchor family protein [Listeria monocytog
. Listeria monocytogenes HPB2262 ......................   28    1 hit  [firmicutes]          hypothetical protein LMSG_00080 [Listeria monocytogenes HPB
. Listeria monocytogenes FSL N1-017 ...................   28    1 hit  [firmicutes]          hypothetical protein LMHG_00875 [Listeria monocytogenes FSL
. Listeria monocytogenes str. 4b F2365 ................   28    1 hit  [firmicutes]          cell wall surface anchor family protein [Listeria monocytog
. Listeria monocytogenes str. 4b H7858 ................   28    1 hit  [firmicutes]          cell wall surface anchor family protein [Listeria monocytog
. Streptococcus pyogenes MGAS10750 ....................   27    3 hits [firmicutes]          Putative surface protein [Streptococcus pyogenes MGAS10750]
. Streptococcus pyogenes str. Manfredo ................   26    1 hit  [firmicutes]          putative surface-anchored protein [Streptococcus pyogenes s
. Streptococcus pyogenes MGAS10270 ....................   26    1 hit  [firmicutes]          cell surface protein [Streptococcus pyogenes MGAS10270]
. Streptococcus pyogenes MGAS9429 .....................   26    1 hit  [firmicutes]          cell surface protein [Streptococcus pyogenes MGAS9429] >gi|
. Streptococcus pyogenes MGAS2096 .....................   26    1 hit  [firmicutes]          cell surface protein [Streptococcus pyogenes MGAS9429] >gi|
. Streptococcus pyogenes MGAS10394 ....................   26    1 hit  [firmicutes]          cell surface protein [Streptococcus pyogenes MGAS10394]
. Streptococcus pyogenes MGAS6180 .....................   26    1 hit  [firmicutes]          cell surface protein [Streptococcus pyogenes MGAS6180]
. Streptococcus pyogenes MGAS315 ......................   26    1 hit  [firmicutes]          putative surface antigen [Streptococcus pyogenes MGAS315]
. Streptococcus pyogenes M1 GAS .......................   26    1 hit  [firmicutes]          hypothetical protein SPy_0843 [Streptococcus pyogenes M1 GA
. Streptococcus pyogenes MGAS5005 .....................   26    1 hit  [firmicutes]          hypothetical protein SPy_0843 [Streptococcus pyogenes M1 GA
. Streptococcus pyogenes MGAS8232 .....................   26    1 hit  [firmicutes]          hypothetical protein spyM18_0903 [Streptococcus pyogenes MG
. Streptococcus pyogenes SSI-1 ........................   26    1 hit  [firmicutes]          hypothetical protein SPs1285 [Streptococcus pyogenes SSI-1]
. Streptococcus pyogenes M49 591 ......................   26    1 hit  [firmicutes]          COG0556: Helicase subunit of the DNA excision repair comple
```

---

**Organism Report**

```
  Trichomonas vaginalis G3 [trichomonads] taxid 412133
 ref|XP_001321233.1| surface antigen BspA-like [Trichomonas...     774  0.0
 ref|XP_001313891.1| surface antigen BspA-like [Trichomonas...     520  4e-153
 ref|XP_001295773.1| surface antigen BspA-like [Trichomonas...     399  9e-117
 ref|XP_001327783.1| surface antigen BspA-like [Trichomonas...     352  2e-102
 ref|XP_001578939.1| surface antigen BspA-like [Trichomonas...     298  4e-86
 ref|XP_001300375.1| surface antigen BspA-like [Trichomonas...     277  6e-80
 ref|XP_001303246.1| surface antigen BspA-like [Trichomonas...     267  6e-77
 ref|XP_001325637.1| surface antigen BspA-like [Trichomonas...     266  1e-76
 ref|XP_001327995.1| surface antigen BspA-like [Trichomonas...     259  2e-74
 ref|XP_001310018.1| surface antigen BspA-like [Trichomonas...     247  5e-71
 ref|XP_001311472.1| surface antigen BspA-like [Trichomonas...     240  9e-69
 ref|XP_001313156.1| surface antigen BspA-like [Trichomonas...     232  2e-66
 ref|XP_001300837.1| surface antigen BspA-like [Trichomonas...     228  3e-65
 ref|XP_001300913.1| surface antigen BspA-like [Trichomonas...     221  3e-63
 ref|XP_001584042.1| surface antigen BspA-like [Trichomonas...     218  4e-62
 ref|XP_001317101.1| surface antigen BspA-like [Trichomonas...     218  5e-62
 ref|XP_001327997.1| surface antigen BspA-like [Trichomonas...     216  1e-61
 ref|XP_001318269.1| surface antigen BspA-like [Trichomonas...     211  5e-60
 ref|XP_001323112.1| surface antigen BspA-like [Trichomonas...     199  3e-56
 ref|XP_001327776.1| surface antigen BspA-like [Trichomonas...     188  5e-53
 ref|XP_001294553.1| surface antigen BspA-like [Trichomonas...     185  2e-52
 ref|XP_001302174.1| surface antigen BspA-like [Trichomonas...     182  3e-51
 ref|XP_001325828.1| surface antigen BspA-like [Trichomonas...     180  1e-50
 ref|XP_001316522.1| surface antigen BspA-like [Trichomonas...     175  3e-49
 ref|XP_001305369.1| surface antigen BspA-like [Trichomonas...     174  6e-49
 ref|XP_001583570.1| surface antigen BspA-like [Trichomonas...     173  2e-48
 ref|XP_001320775.1| surface antigen BspA-like [Trichomonas...     173  2e-48
 ref|XP_001311470.1| surface antigen BspA-like [Trichomonas...     171  6e-48
 ref|XP_001313426.1| surface antigen BspA-like [Trichomonas...     165  4e-46
 ref|XP_001326140.1| surface antigen BspA-like [Trichomonas...     162  2e-45
 ref|XP_001313272.1| surface antigen BspA-like [Trichomonas...     161  5e-45
 ref|XP_001578993.1| surface antigen BspA-like [Trichomonas...     161  7e-45
 ref|XP_001319549.1| surface antigen BspA-like [Trichomonas...     156  2e-43
 ref|XP_001315578.1| surface antigen BspA-like [Trichomonas...     155  4e-43
 ref|XP_001582116.1| surface antigen BspA-like [Trichomonas...     154  6e-43
 ref|XP_001322944.1| surface antigen BspA-like [Trichomonas...     153  1e-42
 ref|XP_001319437.1| surface antigen BspA-like [Trichomonas...     151  7e-42
 ref|XP_001583568.1| surface antigen BspA-like [Trichomonas...     150  1e-41
 ref|XP_001319438.1| surface antigen BspA-like [Trichomonas...     148  4e-41
 ref|XP_001318271.1| surface antigen BspA-like [Trichomonas...     148  4e-41
 ref|XP_001582486.1| surface antigen BspA-like [Trichomonas...     147  8e-41
 ref|XP_001315859.1| surface antigen BspA-like [Trichomonas...     146  2e-40
 ref|XP_001583025.1| surface antigen BspA-like [Trichomonas...     145  5e-40
 ref|XP_001328562.1| surface antigen BspA-like [Trichomonas...     145  5e-40
 ref|XP_001315000.1| surface antigen BspA-like [Trichomonas...     143  1e-39
 ref|XP_001315863.1| surface antigen BspA-like [Trichomonas...     142  3e-39
 ref|XP_001307704.1| surface antigen BspA-like [Trichomonas...     136  2e-37
 ref|XP_001583571.1| surface antigen BspA-like [Trichomonas...     135  3e-37
 ref|XP_001580208.1| surface antigen BspA-like [Trichomonas...     134  9e-37
 ref|XP_001326204.1| surface antigen BspA-like [Trichomonas...     134  9e-37
 ref|XP_001326586.1| surface antigen BspA-like [Trichomonas...     134  9e-37
 ref|XP_001313418.1| surface antigen BspA-like [Trichomonas...     134  9e-37
 ref|XP_001583764.1| surface antigen BspA-like [Trichomonas...     132  3e-36
 ref|XP_001328674.1| surface antigen BspA-like [Trichomonas...     132  3e-36
 ref|XP_001296463.1| surface antigen BspA-like [Trichomonas...     131  4e-36
 ref|XP_001310359.1| surface antigen BspA-like [Trichomonas...     130  1e-35
 ref|XP_001584044.1| surface antigen BspA-like [Trichomonas...     129  2e-35
 ref|XP_001316471.1| surface antigen BspA-like [Trichomonas...     128  5e-35
 ref|XP_001580185.1| surface antigen BspA-like [Trichomonas...     127  1e-34
 ref|XP_001326170.1| surface antigen BspA-like [Trichomonas...     127  1e-34
 ref|XP_001314904.1| Leucine Rich Repeat family protein [Tr...     127  1e-34
 ref|XP_001311382.1| surface antigen BspA-like [Trichomonas...     126  2e-34
 ref|XP_001328347.1| surface antigen BspA-like [Trichomonas...     125  4e-34
 ref|XP_001288325.1| surface antigen BspA-like [Trichomonas...     123  1e-33
 ref|XP_001582154.1| surface antigen BspA-like [Trichomonas...     123  2e-33
 ref|XP_001314765.1| surface antigen BspA-like [Trichomonas...     123  2e-33
 ref|XP_001305199.1| surface antigen BspA-like [Trichomonas...     123  2e-33
 ref|XP_001320680.1| surface antigen BspA-like [Trichomonas...     122  3e-33
 ref|XP_001584517.1| surface antigen BspA-like [Trichomonas...     122  3e-33
 ref|XP_001316735.1| surface antigen BspA-like [Trichomonas...     122  3e-33
 ref|XP_001314401.1| surface antigen BspA-like [Trichomonas...     121  5e-33
 ref|XP_001313953.1| surface antigen BspA-like [Trichomonas...     121  6e-33
 ref|XP_001582619.1| surface antigen BspA-like [Trichomonas...     121  8e-33
 ref|XP_001315147.1| surface antigen BspA-like [Trichomonas...     120  1e-32
 ref|XP_001584312.1| surface antigen BspA-like [Trichomonas...     120  1e-32
 ref|XP_001326610.1| surface antigen BspA-like [Trichomonas...     119  3e-32
 ref|XP_001311580.1| cell surface protein, putative [Tricho...     118  4e-32
 ref|XP_001316680.1| surface antigen BspA-like [Trichomonas...     117  9e-32
 ref|XP_001321334.1| surface antigen BspA-like [Trichomonas...     116  2e-31
 ref|XP_001329959.1| surface antigen BspA-like [Trichomonas...     115  3e-31
 ref|XP_001582153.1| surface antigen BspA-like [Trichomonas...     115  4e-31
 ref|XP_001316682.1| cell surface protein, putative [Tricho...     114  6e-31
 ref|XP_001583564.1| cell surface protein, putative [Tricho...     114  7e-31
 ref|XP_001321571.1| surface antigen BspA-like [Trichomonas...     114  7e-31
 ref|XP_001316457.1| surface antigen BspA-like [Trichomonas...     114  7e-31
 ref|XP_001317155.1| surface antigen BspA-like [Trichomonas...     113  1e-30
 ref|XP_001310344.1| surface antigen BspA-like [Trichomonas...     113  1e-30
 ref|XP_001327215.1| surface antigen BspA-like [Trichomonas...     112  4e-30
 ref|XP_001330077.1| surface antigen BspA-like [Trichomonas...     112  4e-30
 ref|XP_001320608.1| surface antigen BspA-like [Trichomonas...     111  5e-30
 ref|XP_001307862.1| surface antigen BspA-like [Trichomonas...     110  1e-29
 ref|XP_001303561.1| surface antigen BspA-like [Trichomonas...     110  1e-29
 ref|XP_001314845.1| surface antigen BspA-like [Trichomonas...     109  2e-29
 ref|XP_001278277.1| surface antigen BspA-like [Trichomonas...     109  2e-29
 ref|XP_001320160.1| surface antigen BspA-like [Trichomonas...     109  2e-29
 ref|XP_001584518.1| surface antigen BspA-like [Trichomonas...     109  3e-29
 ref|XP_001317132.1| surface antigen BspA-like [Trichomonas...     109  3e-29
 ref|XP_001318845.1| surface antigen BspA-like [Trichomonas...     108  4e-29
 ref|XP_001580137.1| surface antigen BspA-like [Trichomonas...     108  7e-29
 ref|XP_001583668.1| surface antigen BspA-like [Trichomonas...     107  1e-28
 ref|XP_001306128.1| surface antigen BspA-like [Trichomonas...     107  1e-28
 ref|XP_001297020.1| surface antigen BspA-like [Trichomonas...     107  1e-28
 ref|XP_001314499.1| surface antigen BspA-like [Trichomonas...     106  2e-28
 ref|XP_001580983.1| surface antigen BspA-like [Trichomonas...     106  2e-28
 ref|XP_001581243.1| surface antigen BspA-like [Trichomonas...     106  2e-28
 ref|XP_001319612.1| Leucine Rich Repeat family protein [Tr...     106  3e-28
 ref|XP_001307421.1| hypothetical protein TVAG_241760 [Tric...     106  3e-28
 ref|XP_001583892.1| surface antigen BspA-like [Trichomonas...     105  3e-28
 ref|XP_001316472.1| surface antigen BspA-like [Trichomonas...     105  3e-28
 ref|XP_001300468.1| Leucine Rich Repeat family protein [Tr...     105  3e-28
 ref|XP_001328355.1| surface antigen BspA-like [Trichomonas...     105  4e-28
 ref|XP_001306181.1| surface antigen BspA-like [Trichomonas...     105  4e-28
 ref|XP_001326595.1| surface antigen BspA-like [Trichomonas...     104  6e-28
 ref|XP_001312800.1| surface antigen BspA-like [Trichomonas...     104  6e-28
 ref|XP_001579760.1| surface antigen BspA-like [Trichomonas...     104  7e-28
 ref|XP_001580031.1| surface antigen BspA-like [Trichomonas...     104  7e-28
 ref|XP_001584521.1| surface antigen BspA-like [Trichomonas...     104  7e-28
 ref|XP_001317424.1| surface antigen BspA-like [Trichomonas...     104  7e-28
 ref|XP_001310243.1| surface antigen BspA-like [Trichomonas...     104  7e-28
 ref|XP_001307420.1| surface antigen BspA-like [Trichomonas...     104  7e-28
 ref|XP_001304480.1| surface antigen BspA-like [Trichomonas...     104  7e-28
 ref|XP_001584309.1| surface antigen BspA-like [Trichomonas...     103  1e-27
 ref|XP_001307417.1| surface antigen BspA-like [Trichomonas...     103  1e-27
 ref|XP_001578977.1| surface antigen BspA-like [Trichomonas...     103  2e-27
 ref|XP_001319243.1| surface antigen BspA-like [Trichomonas...     103  2e-27
 ref|XP_001304390.1| surface antigen BspA-like [Trichomonas...     103  2e-27
 ref|XP_001328364.1| hypothetical protein TVAG_465290 [Tric...     102  3e-27
 ref|XP_001309839.1| surface antigen BspA-like [Trichomonas...     102  3e-27
 ref|XP_001584519.1| surface antigen BspA-like [Trichomonas...     102  4e-27
 ref|XP_001580174.1| surface antigen BspA-like [Trichomonas...     102  4e-27
 ref|XP_001325465.1| Leucine Rich Repeat family protein [Tr...     102  4e-27
 ref|XP_001327996.1| surface antigen BspA-like [Trichomonas...     102  4e-27
 ref|XP_001328231.1| surface antigen BspA-like [Trichomonas...     102  4e-27
 ref|XP_001321229.1| surface antigen BspA-like [Trichomonas...     102  4e-27
 ref|XP_001308904.1| surface antigen BspA-like [Trichomonas...     102  4e-27
 ref|XP_001313121.1| surface antigen BspA-like [Trichomonas...     101  5e-27
 ref|XP_001584314.1| surface antigen BspA-like [Trichomonas...     101  6e-27
 ref|XP_001311047.1| surface antigen BspA-like [Trichomonas...     100  1e-26
 ref|XP_001325834.1| surface antigen BspA-like [Trichomonas...     100  1e-26
 ref|XP_001306722.1| surface antigen BspA-like [Trichomonas...     100  1e-26
 ref|XP_001301406.1| surface antigen BspA-like [Trichomonas...     100  1e-26
 ref|XP_001325301.1| cell surface protein, putative [Tricho...      99  2e-26
 ref|XP_001320123.1| surface antigen BspA-like [Trichomonas...      99  2e-26
 ref|XP_001309234.1| surface antigen BspA-like [Trichomonas...      99  2e-26
 ref|XP_001581880.1| surface antigen BspA-like [Trichomonas...      99  2e-26
 ref|XP_001322526.1| Leucine Rich Repeat family protein [Tr...      99  2e-26
 ref|XP_001328090.1| surface antigen BspA-like [Trichomonas...      99  2e-26
 ref|XP_001316694.1| surface antigen BspA-like [Trichomonas...      99  2e-26
 ref|XP_001316557.1| surface antigen BspA-like [Trichomonas...      99  2e-26
 ref|XP_001320607.1| surface antigen BspA-like [Trichomonas...      99  2e-26
 ref|XP_001324655.1| surface antigen BspA-like [Trichomonas...      98  4e-26
 ref|XP_001309881.1| surface antigen BspA-like [Trichomonas...      98  4e-26
 ref|XP_001312385.1| surface antigen BspA-like [Trichomonas...      98  4e-26
 ref|XP_001582172.1| surface antigen BspA-like [Trichomonas...      98  5e-26
 ref|XP_001308549.1| surface antigen BspA-like [Trichomonas...      98  5e-26
 ref|XP_001308356.1| surface antigen BspA-like [Trichomonas...      98  5e-26
 ref|XP_001303552.1| surface antigen BspA-like [Trichomonas...      98  5e-26
 ref|XP_001321875.1| surface antigen BspA-like [Trichomonas...      98  7e-26
 ref|XP_001583766.1| surface antigen BspA-like [Trichomonas...      97  9e-26
 ref|XP_001309390.1| surface antigen BspA-like [Trichomonas...      97  9e-26
 ref|XP_001302758.1| surface antigen BspA-like [Trichomonas...      97  9e-26
 ref|XP_001583967.1| surface antigen BspA-like [Trichomonas...      97  1e-25
 ref|XP_001309216.1| Leucine Rich Repeat family protein [Tr...      97  1e-25
 ref|XP_001276952.1| Leucine Rich Repeat family protein [Tr...      96  2e-25
 ref|XP_001329658.1| surface antigen BspA-like [Trichomonas...      96  2e-25
 ref|XP_001330142.1| surface antigen BspA-like [Trichomonas...      96  2e-25
 ref|XP_001309842.1| surface antigen BspA-like [Trichomonas...      96  2e-25
 ref|XP_001329761.1| surface antigen BspA-like [Trichomonas...      96  2e-25
 ref|XP_001316473.1| surface antigen BspA-like [Trichomonas...      96  2e-25
 ref|XP_001306978.1| surface antigen BspA-like [Trichomonas...      96  3e-25
 ref|XP_001311252.1| surface antigen BspA-like [Trichomonas...      95  3e-25
 ref|XP_001323136.1| surface antigen BspA-like [Trichomonas...      95  4e-25
 ref|XP_001323192.1| surface antigen BspA-like [Trichomonas...      95  4e-25
 ref|XP_001316739.1| surface antigen BspA-like [Trichomonas...      95  4e-25
 ref|XP_001308920.1| hypothetical protein TVAG_052480 [Tric...      95  4e-25
 ref|XP_001301434.1| surface antigen BspA-like [Trichomonas...      95  4e-25
 ref|XP_001584520.1| surface antigen BspA-like [Trichomonas...      94  6e-25
 ref|XP_001583613.1| surface antigen BspA-like [Trichomonas...      94  6e-25
 ref|XP_001304472.1| hypothetical protein TVAG_376540 [Tric...      94  6e-25
 ref|XP_001322549.1| surface antigen BspA-like [Trichomonas...      94  8e-25
 ref|XP_001322525.1| Leucine Rich Repeat family protein [Tr...      94  8e-25
 ref|XP_001319647.1| surface antigen BspA-like [Trichomonas...      94  8e-25
 ref|XP_001329219.1| surface antigen BspA-like [Trichomonas...      94  1e-24
 ref|XP_001316474.1| surface antigen BspA-like [Trichomonas...      94  1e-24
 ref|XP_001311149.1| surface antigen BspA-like [Trichomonas...      94  1e-24
 ref|XP_001310683.1| Leucine Rich Repeat family protein [Tr...      94  1e-24
 ref|XP_001307216.1| surface antigen BspA-like [Trichomonas...      94  1e-24
 ref|XP_001307744.1| surface antigen BspA-like [Trichomonas...      94  1e-24
 ref|XP_001301145.1| surface antigen BspA-like [Trichomonas...      94  1e-24
 ref|XP_001285293.1| surface antigen BspA-like [Trichomonas...      94  1e-24
 ref|XP_001322517.1| Leucine Rich Repeat family protein [Tr...      93  1e-24
 ref|XP_001327818.1| surface antigen BspA-like [Trichomonas...      93  1e-24
 ref|XP_001579564.1| surface antigen BspA-like [Trichomonas...      93  2e-24
 ref|XP_001583817.1| surface antigen BspA-like [Trichomonas...      93  2e-24
 ref|XP_001329823.1| surface antigen BspA-like [Trichomonas...      93  2e-24
 ref|XP_001316485.1| surface antigen BspA-like [Trichomonas...      93  2e-24
 ref|XP_001309884.1| surface antigen Bsp, putative [Trichom...      93  2e-24
 ref|XP_001312006.1| surface antigen BspA-like [Trichomonas...      93  2e-24
 ref|XP_001307497.1| surface antigen BspA-like [Trichomonas...      93  2e-24
 ref|XP_001298289.1| surface antigen BspA-like [Trichomonas...      92  3e-24
 ref|XP_001581326.1| surface antigen BspA-like [Trichomonas...      92  4e-24
 ref|XP_001313404.1| surface antigen BspA-like [Trichomonas...      92  4e-24
 ref|XP_001312134.1| surface antigen BspA-like [Trichomonas...      92  4e-24
 ref|XP_001297832.1| surface antigen BspA-like [Trichomonas...      92  4e-24
 ref|XP_001296128.1| surface antigen BspA-like [Trichomonas...      92  4e-24
 ref|XP_001580139.1| surface antigen BspA-like [Trichomonas...      91  5e-24
 ref|XP_001306833.1| surface antigen BspA-like [Trichomonas...      91  5e-24
 ref|XP_001579825.1| surface antigen BspA-like [Trichomonas...      91  8e-24
 ref|XP_001326822.1| surface antigen BspA-like [Trichomonas...      91  8e-24
 ref|XP_001328723.1| surface antigen BspA-like [Trichomonas...      91  8e-24
 ref|XP_001321471.1| surface antigen BspA-like [Trichomonas...      91  8e-24
 ref|XP_001310095.1| Leucine Rich Repeat family protein [Tr...      91  8e-24
 ref|XP_001296869.1| surface antigen BspA-like [Trichomonas...      91  8e-24
 ref|XP_001327196.1| Leucine Rich Repeat family protein [Tr...      90  1e-23
 ref|XP_001327190.1| surface antigen BspA-like [Trichomonas...      90  1e-23
 ref|XP_001277016.1| Leucine Rich Repeat family protein [Tr...      90  1e-23
 ref|XP_001328163.1| surface antigen BspA-like [Trichomonas...      90  1e-23
 ref|XP_001327834.1| Leucine Rich Repeat family protein [Tr...      90  1e-23
 ref|XP_001303554.1| surface antigen BspA-like [Trichomonas...      90  1e-23
 ref|XP_001300663.1| surface antigen BspA-like [Trichomonas...      90  1e-23
 ref|XP_001297026.1| surface antigen BspA-like [Trichomonas...      89  2e-23
 ref|XP_001277015.1| Leucine Rich Repeat family protein [Tr...      89  2e-23
 ref|XP_001311508.1| surface antigen BspA-like [Trichomonas...      89  2e-23
 ref|XP_001307861.1| surface antigen BspA-like [Trichomonas...      89  2e-23
 ref|XP_001302667.1| surface antigen BspA-like [Trichomonas...      89  2e-23
 ref|XP_001280250.1| surface antigen BspA-like [Trichomonas...      89  2e-23
 ref|XP_001311197.1| surface antigen BspA-like [Trichomonas...      89  3e-23
 ref|XP_001311308.1| surface antigen BspA-like [Trichomonas...      89  3e-23
 ref|XP_001580341.1| surface antigen BspA-like [Trichomonas...      88  4e-23
 ref|XP_001315574.1| Leucine Rich Repeat family protein [Tr...      88  4e-23
 ref|XP_001315820.1| surface antigen BspA-like [Trichomonas...      88  4e-23
 ref|XP_001312386.1| Leucine Rich Repeat family protein [Tr...      88  4e-23
 ref|XP_001309065.1| surface antigen BspA-like [Trichomonas...      88  4e-23
 ref|XP_001305184.1| Leucine Rich Repeat family protein [Tr...      88  4e-23
 ref|XP_001294025.1| surface antigen BspA-like [Trichomonas...      88  4e-23
 ref|XP_001584310.1| surface antigen BspA-like [Trichomonas...      88  5e-23
 ref|XP_001327177.1| hypothetical protein TVAG_396840 [Tric...      88  5e-23
 ref|XP_001321309.1| surface antigen BspA-like [Trichomonas...      88  5e-23
 ref|XP_001330279.1| Leucine Rich Repeat family protein [Tr...      88  5e-23
 ref|XP_001310600.1| surface antigen BspA-like [Trichomonas...      88  5e-23
 ref|XP_001308799.1| surface antigen BspA-like [Trichomonas...      88  5e-23
 ref|XP_001276867.1| surface antigen BspA-like [Trichomonas...      88  7e-23
 ref|XP_001322369.1| Leucine Rich Repeat family protein [Tr...      88  7e-23
 ref|XP_001316192.1| surface antigen BspA-like [Trichomonas...      88  7e-23
 ref|XP_001314937.1| surface antigen BspA-like [Trichomonas...      88  7e-23
 ref|XP_001580874.1| surface antigen BspA-like [Trichomonas...      87  9e-23
 ref|XP_001583256.1| surface antigen BspA-like [Trichomonas...      87  9e-23
 ref|XP_001322365.1| surface antigen Bsp, putative [Trichom...      87  9e-23
 ref|XP_001276979.1| Leucine Rich Repeat family protein [Tr...      87  1e-22
 ref|XP_001583765.1| surface antigen BspA-like [Trichomonas...      87  1e-22
 ref|XP_001584306.1| surface antigen BspA-like [Trichomonas...      87  1e-22
 ref|XP_001315236.1| surface antigen BspA-like [Trichomonas...      87  1e-22
 ref|XP_001312690.1| surface antigen BspA-like [Trichomonas...      87  1e-22
 ref|XP_001276937.1| surface antigen BspA-like [Trichomonas...      86  2e-22
 ref|XP_001317245.1| surface antigen BspA-like [Trichomonas...      86  2e-22
 ref|XP_001318545.1| surface antigen BspA-like [Trichomonas...      86  2e-22
 ref|XP_001306817.1| surface antigen BspA-like [Trichomonas...      86  2e-22
 ref|XP_001316797.1| surface antigen BspA-like [Trichomonas...      86  2e-22
 ref|XP_001311188.1| surface antigen BspA-like [Trichomonas...      86  2e-22
 ref|XP_001324335.1| surface antigen BspA-like [Trichomonas...      86  3e-22
 ref|XP_001322537.1| Leucine Rich Repeat family protein [Tr...      86  3e-22
 ref|XP_001317421.1| surface antigen BspA-like [Trichomonas...      86  3e-22
 ref|XP_001318754.1| Leucine Rich Repeat family protein [Tr...      86  3e-22
 ref|XP_001315235.1| surface antigen BspA-like [Trichomonas...      86  3e-22
 ref|XP_001329030.1| Leucine Rich Repeat family protein [Tr...      85  3e-22
 ref|XP_001316678.1| surface antigen BspA-like [Trichomonas...      85  3e-22
 ref|XP_001309820.1| surface antigen BspA-like [Trichomonas...      85  3e-22
 ref|XP_001323432.1| surface antigen BspA-like [Trichomonas...      85  4e-22
 ref|XP_001330231.1| surface antigen BspA-like [Trichomonas...      85  4e-22
 ref|XP_001309784.1| surface antigen BspA-like [Trichomonas...      85  4e-22
 ref|XP_001309347.1| surface antigen BspA-like [Trichomonas...      85  4e-22
 ref|XP_001330691.1| surface antigen BspA-like [Trichomonas...      84  6e-22
 ref|XP_001310694.1| surface antigen BspA-like [Trichomonas...      84  6e-22
 ref|XP_001307858.1| surface antigen BspA-like [Trichomonas...      84  6e-22
 ref|XP_001327832.1| surface antigen BspA-like [Trichomonas...      84  8e-22
 ref|XP_001308641.1| surface antigen BspA-like [Trichomonas...      84  8e-22
 ref|XP_001302316.1| Leucine Rich Repeat family protein [Tr...      84  8e-22
 ref|XP_001579739.1| surface antigen BspA-like [Trichomonas...      84  1e-21
 ref|XP_001326021.1| surface antigen BspA-like [Trichomonas...      84  1e-21
 ref|XP_001320904.1| surface antigen BspA-like [Trichomonas...      84  1e-21
 ref|XP_001317928.1| surface antigen BspA-like [Trichomonas...      84  1e-21
 ref|XP_001324897.1| Leucine Rich Repeat family protein [Tr...      83  1e-21
 ref|XP_001316127.1| surface antigen BspA-like [Trichomonas...      83  1e-21
 ref|XP_001327210.1| surface antigen BspA-like [Trichomonas...      83  2e-21
 ref|XP_001300591.1| surface antigen BspA-like [Trichomonas...      83  2e-21
 ref|XP_001324795.1| surface antigen BspA-like [Trichomonas...      83  2e-21
 ref|XP_001323660.1| Leucine Rich Repeat family protein [Tr...      83  2e-21
 ref|XP_001329934.1| surface antigen BspA-like [Trichomonas...      83  2e-21
 ref|XP_001317274.1| surface antigen BspA-like [Trichomonas...      83  2e-21
 ref|XP_001310695.1| Leucine Rich Repeat family protein [Tr...      83  2e-21
 ref|XP_001305729.1| surface antigen BspA-like [Trichomonas...      83  2e-21
 ref|XP_001302029.1| surface antigen BspA-like [Trichomonas...      82  3e-21
 ref|XP_001300473.1| surface antigen BspA-like [Trichomonas...      82  3e-21
 ref|XP_001298474.1| surface antigen BspA-like [Trichomonas...      82  3e-21
 ref|XP_001291104.1| surface antigen BspA-like [Trichomonas...      82  3e-21
 ref|XP_001329755.1| Leucine Rich Repeat family protein [Tr...      82  4e-21
 ref|XP_001310586.1| surface antigen BspA-like [Trichomonas...      82  4e-21
 ref|XP_001308539.1| Leucine Rich Repeat family protein [Tr...      82  4e-21
 ref|XP_001323401.1| Leucine Rich Repeat family protein [Tr...      81  5e-21
 ref|XP_001309450.1| surface antigen BspA-like [Trichomonas...      81  5e-21
 ref|XP_001580641.1| cell surface protein, putative [Tricho...      81  6e-21
 ref|XP_001313165.1| surface antigen BspA-like [Trichomonas...      81  6e-21
 ref|XP_001318748.1| surface antigen BspA-like [Trichomonas...      81  8e-21
 ref|XP_001303242.1| surface antigen BspA-like [Trichomonas...      81  8e-21
 ref|XP_001317386.1| hypothetical protein TVAG_058200 [Tric...      80  1e-20
 ref|XP_001312375.1| surface antigen BspA-like [Trichomonas...      80  1e-20
 ref|XP_001330718.1| surface antigen BspA-like [Trichomonas...      80  1e-20
 ref|XP_001322411.1| surface antigen BspA-like [Trichomonas...      80  1e-20
 ref|XP_001310673.1| surface antigen BspA-like [Trichomonas...      80  1e-20
 ref|XP_001312390.1| surface antigen BspA-like [Trichomonas...      80  1e-20
 ref|XP_001581729.1| surface antigen BspA-like [Trichomonas...      80  2e-20
 ref|XP_001329258.1| surface antigen BspA-like [Trichomonas...      80  2e-20
 ref|XP_001315559.1| surface antigen BspA-like [Trichomonas...      80  2e-20
 ref|XP_001309886.1| cell surface protein, putative [Tricho...      80  2e-20
 ref|XP_001305131.1| surface antigen BspA-like [Trichomonas...      80  2e-20
 ref|XP_001579116.1| surface antigen BspA-like [Trichomonas...      79  2e-20
 ref|XP_001327214.1| Leucine Rich Repeat family protein [Tr...      79  2e-20
 ref|XP_001322084.1| surface antigen BspA-like [Trichomonas...      79  2e-20
 ref|XP_001302598.1| surface antigen BspA-like [Trichomonas...      79  2e-20
 ref|XP_001325362.1| surface antigen BspA-like [Trichomonas...      79  3e-20
 ref|XP_001324305.1| surface antigen BspA-like [Trichomonas...      79  3e-20
 ref|XP_001303661.1| surface antigen BspA-like [Trichomonas...      79  3e-20
 ref|XP_001582264.1| surface antigen BspA-like [Trichomonas...      78  4e-20
 ref|XP_001323088.1| surface antigen BspA-like [Trichomonas...      78  4e-20
 ref|XP_001330675.1| cell surface protein, putative [Tricho...      78  4e-20
 ref|XP_001304295.1| surface antigen BspA-like [Trichomonas...      78  4e-20
 ref|XP_001304475.1| Leucine Rich Repeat family protein [Tr...      78  4e-20
 ref|XP_001328365.1| surface antigen BspA-like [Trichomonas...      78  5e-20
 ref|XP_001320323.1| Leucine Rich Repeat family protein [Tr...      78  5e-20
 ref|XP_001315653.1| Leucine Rich Repeat family protein [Tr...      78  5e-20
 ref|XP_001315446.1| surface antigen BspA-like [Trichomonas...      78  5e-20
 ref|XP_001312325.1| hypothetical protein TVAG_043030 [Tric...      78  5e-20
 ref|XP_001308031.1| surface antigen BspA-like [Trichomonas...      78  5e-20
 ref|XP_001579737.1| surface antigen BspA-like [Trichomonas...      77  9e-20
 ref|XP_001313390.1| surface antigen Bsp, putative [Trichom...      77  9e-20
 ref|XP_001307334.1| surface antigen BspA-like [Trichomonas...      77  9e-20
 ref|XP_001306375.1| surface antigen BspA-like [Trichomonas...      77  9e-20
 ref|XP_001325770.1| surface antigen BspA-like [Trichomonas...      77  1e-19
 ref|XP_001323277.1| surface antigen BspA-like [Trichomonas...      77  1e-19
 ref|XP_001327833.1| surface antigen BspA-like [Trichomonas...      77  1e-19
 ref|XP_001311500.1| surface antigen BspA-like [Trichomonas...      77  1e-19
 ref|XP_001309824.1| surface antigen BspA-like [Trichomonas...      77  1e-19
 ref|XP_001318044.1| surface antigen BspA-like [Trichomonas...      76  2e-19
 ref|XP_001309885.1| surface antigen BspA-like [Trichomonas...      76  2e-19
 ref|XP_001308805.1| surface antigen BspA-like [Trichomonas...      76  2e-19
 ref|XP_001309051.1| surface antigen BspA-like [Trichomonas...      76  2e-19
 ref|XP_001306350.1| surface antigen BspA-like [Trichomonas...      76  2e-19
 ref|XP_001322761.1| surface antigen BspA-like [Trichomonas...      75  3e-19
 ref|XP_001314105.1| surface antigen BspA-like [Trichomonas...      75  3e-19
 ref|XP_001583614.1| surface antigen BspA-like [Trichomonas...      75  4e-19
 ref|XP_001581093.1| surface antigen BspA-like [Trichomonas...      75  4e-19
 ref|XP_001308141.1| surface antigen BspA-like [Trichomonas...      75  4e-19
 ref|XP_001307423.1| surface antigen BspA-like [Trichomonas...      75  6e-19
 ref|XP_001327395.1| surface antigen BspA-like [Trichomonas...      74  8e-19
 ref|XP_001302033.1| surface antigen BspA-like [Trichomonas...      74  8e-19
 ref|XP_001295737.1| surface antigen BspA-like [Trichomonas...      74  8e-19
 ref|XP_001583078.1| surface antigen BspA-like [Trichomonas...      74  1e-18
 ref|XP_001307859.1| Leucine Rich Repeat family protein [Tr...      74  1e-18
 ref|XP_001323988.1| surface antigen BspA-like [Trichomonas...      73  1e-18
 ref|XP_001315701.1| surface antigen BspA-like [Trichomonas...      73  2e-18
 ref|XP_001303553.1| surface antigen BspA-like [Trichomonas...      73  2e-18
 ref|XP_001287142.1| surface antigen BspA-like [Trichomonas...      73  2e-18
 ref|XP_001582620.1| surface antigen BspA-like [Trichomonas...      73  2e-18
 ref|XP_001584303.1| surface antigen BspA-like [Trichomonas...      73  2e-18
 ref|XP_001313118.1| surface antigen BspA-like [Trichomonas...      73  2e-18
 ref|XP_001301673.1| cell surface protein, putative [Tricho...      72  3e-18
 ref|XP_001303105.1| surface antigen BspA-like [Trichomonas...      72  3e-18
 ref|XP_001313119.1| surface antigen Bsp, putative [Trichom...      72  4e-18
 ref|XP_001321287.1| surface antigen BspA-like [Trichomonas...      71  5e-18
 ref|XP_001316679.1| surface antigen BspA-like [Trichomonas...      71  5e-18
 ref|XP_001318842.1| surface antigen BspA-like [Trichomonas...      71  5e-18
 ref|XP_001317271.1| surface antigen BspA-like [Trichomonas...      71  6e-18
 ref|XP_001304114.1| surface antigen BspA-like [Trichomonas...      71  6e-18
 ref|XP_001301818.1| surface antigen BspA-like [Trichomonas...      71  6e-18
 ref|XP_001308231.1| surface antigen BspA-like [Trichomonas...      71  8e-18
 ref|XP_001584308.1| surface antigen BspA-like [Trichomonas...      70  2e-17
 ref|XP_001322571.1| hypothetical protein TVAG_109320 [Tric...      70  2e-17
 ref|XP_001328675.1| surface antigen BspA-like [Trichomonas...      70  2e-17
 ref|XP_001297500.1| surface antigen BspA-like [Trichomonas...      70  2e-17
 ref|XP_001583077.1| surface antigen BspA-like [Trichomonas...      69  2e-17
 ref|XP_001326103.1| hypothetical protein TVAG_028330 [Tric...      69  2e-17
 ref|XP_001297948.1| surface antigen BspA-like [Trichomonas...      69  2e-17
 ref|XP_001329850.1| surface antigen BspA-like [Trichomonas...      69  3e-17
 ref|XP_001320663.1| surface antigen BspA-like [Trichomonas...      69  3e-17
 ref|XP_001311048.1| surface antigen BspA-like [Trichomonas...      69  3e-17
 ref|XP_001322326.1| surface antigen BspA-like [Trichomonas...      68  4e-17
 ref|XP_001311619.1| surface antigen BspA-like [Trichomonas...      68  4e-17
 ref|XP_001311620.1| surface antigen BspA-like [Trichomonas...      68  4e-17
 ref|XP_001307331.1| surface antigen Bsp, putative [Trichom...      68  4e-17
 ref|XP_001323847.1| surface antigen BspA-like [Trichomonas...      68  5e-17
 ref|XP_001320681.1| surface antigen BspA-like [Trichomonas...      68  5e-17
 ref|XP_001309112.1| surface antigen BspA-like [Trichomonas...      68  5e-17
 ref|XP_001304113.1| surface antigen BspA-like [Trichomonas...      68  5e-17
 ref|XP_001579847.1| surface antigen BspA-like [Trichomonas...      68  7e-17
 ref|XP_001325166.1| surface antigen BspA-like [Trichomonas...      68  7e-17
 ref|XP_001317272.1| surface antigen BspA-like [Trichomonas...      68  7e-17
 ref|XP_001309486.1| surface antigen BspA-like [Trichomonas...      68  7e-17
 ref|XP_001302511.1| surface antigen BspA-like [Trichomonas...      68  7e-17
 ref|XP_001580822.1| Leucine Rich Repeat family protein [Tr...      67  9e-17
 ref|XP_001584307.1| surface antigen BspA-like [Trichomonas...      67  9e-17
 ref|XP_001329988.1| surface antigen BspA-like [Trichomonas...      67  9e-17
 ref|XP_001321572.1| surface antigen BspA-like [Trichomonas...      67  9e-17
 ref|XP_001297552.1| surface antigen BspA-like [Trichomonas...      67  9e-17
 ref|XP_001582100.1| surface antigen BspA-like [Trichomonas...      67  1e-16
 ref|XP_001330058.1| surface antigen BspA-like [Trichomonas...      67  1e-16
 ref|XP_001306837.1| surface antigen BspA-like [Trichomonas...      67  1e-16
 ref|XP_001329821.1| surface antigen BspA-like [Trichomonas...      67  2e-16
 ref|XP_001328354.1| surface antigen BspA-like [Trichomonas...      67  2e-16
 ref|XP_001306942.1| surface antigen BspA-like [Trichomonas...      67  2e-16
 ref|XP_001306394.1| surface antigen BspA-like [Trichomonas...      67  2e-16
 ref|XP_001327726.1| surface antigen BspA-like [Trichomonas...      66  2e-16
 ref|XP_001310837.1| surface antigen BspA-like [Trichomonas...      66  2e-16
 ref|XP_001307332.1| cell surface protein, putative [Tricho...      66  2e-16
 ref|XP_001298988.1| surface antigen BspA-like [Trichomonas...      66  2e-16
 ref|XP_001327026.1| surface antigen BspA-like [Trichomonas...      66  3e-16
 ref|XP_001583197.1| surface antigen BspA-like [Trichomonas...      65  3e-16
 ref|XP_001328349.1| surface antigen BspA-like [Trichomonas...      65  3e-16
 ref|XP_001317246.1| surface antigen BspA-like [Trichomonas...      65  3e-16
 ref|XP_001306351.1| surface antigen BspA-like [Trichomonas...      65  3e-16
 ref|XP_001294926.1| surface antigen BspA-like [Trichomonas...      65  4e-16
 ref|XP_001306877.1| surface antigen BspA-like [Trichomonas...      65  6e-16
 ref|XP_001581039.1| surface antigen BspA-like [Trichomonas...      64  7e-16
 ref|XP_001330510.1| surface antigen BspA-like [Trichomonas...      64  7e-16
 ref|XP_001307287.1| surface antigen BspA-like [Trichomonas...      64  7e-16
 ref|XP_001319242.1| surface antigen BspA-like [Trichomonas...      64  1e-15
 ref|XP_001311300.1| surface antigen BspA-like [Trichomonas...      64  1e-15
 ref|XP_001305200.1| surface antigen BspA-like [Trichomonas...      64  1e-15
 ref|XP_001318168.1| surface antigen BspA-like [Trichomonas...      63  1e-15
 ref|XP_001315237.1| surface antigen BspA-like [Trichomonas...      63  1e-15
 ref|XP_001315766.1| hypothetical protein TVAG_041950 [Tric...      63  2e-15
 ref|XP_001323205.1| surface antigen BspA-like [Trichomonas...      63  2e-15
 ref|XP_001307211.1| surface antigen BspA-like [Trichomonas...      63  2e-15
 ref|XP_001583510.1| cell surface protein, putative [Tricho...      62  3e-15
 ref|XP_001314744.1| surface antigen BspA-like [Trichomonas...      62  3e-15
 ref|XP_001302030.1| surface antigen BspA-like [Trichomonas...      62  3e-15
 ref|XP_001584304.1| surface antigen BspA-like [Trichomonas...      62  4e-15
 ref|XP_001579421.1| surface antigen BspA-like [Trichomonas...      62  5e-15
 ref|XP_001315598.1| surface antigen Bsp, putative [Trichom...      61  6e-15
 ref|XP_001328291.1| hypothetical protein TVAG_278340 [Tric...      60  1e-14
 ref|XP_001321548.1| surface antigen BspA-like [Trichomonas...      60  1e-14
 ref|XP_001298020.1| surface antigen BspA-like [Trichomonas...      60  1e-14
 ref|XP_001326539.1| surface antigen BspA-like [Trichomonas...      60  1e-14
 ref|XP_001319517.1| surface antigen BspA-like [Trichomonas...      60  1e-14
 ref|XP_001328361.1| surface antigen BspA-like [Trichomonas...      59  2e-14
 ref|XP_001314807.1| surface antigen BspA-like [Trichomonas...      59  2e-14
 ref|XP_001319162.1| surface antigen BspA-like [Trichomonas...      59  3e-14
 ref|XP_001309448.1| hypothetical protein TVAG_148700 [Tric...      59  4e-14
 ref|XP_001308918.1| hypothetical protein TVAG_052460 [Tric...      59  4e-14
 ref|XP_001306532.1| cell surface protein, putative [Tricho...      59  4e-14
 ref|XP_001327023.1| surface antigen BspA-like [Trichomonas...      58  5e-14
 ref|XP_001580028.1| surface antigen BspA-like [Trichomonas...      58  7e-14
 ref|XP_001299649.1| hypothetical protein TVAG_082800 [Tric...      58  7e-14
 ref|XP_001327024.1| surface antigen BspA-like [Trichomonas...      57  9e-14
 ref|XP_001329976.1| surface antigen BspA-like [Trichomonas...      57  9e-14
 ref|XP_001578976.1| conserved hypothetical protein [Tricho...      57  9e-14
 ref|XP_001310233.1| surface antigen BspA-like [Trichomonas...      57  9e-14
 ref|XP_001311984.1| surface antigen BspA-like [Trichomonas...      57  9e-14
 ref|XP_001326355.1| surface antigen BspA-like [Trichomonas...      57  1e-13
 ref|XP_001329820.1| surface antigen BspA-like [Trichomonas...      57  1e-13
 ref|XP_001583196.1| conserved hypothetical protein [Tricho...      57  1e-13
 ref|XP_001303399.1| surface antigen BspA-like [Trichomonas...      56  2e-13
 ref|XP_001305581.1| surface antigen Bsp, putative [Trichom...      55  3e-13
 ref|XP_001328760.1| surface antigen BspA-like [Trichomonas...      55  4e-13
 ref|XP_001328327.1| conserved hypothetical protein [Tricho...      55  5e-13
 ref|XP_001318284.1| surface antigen BspA-like [Trichomonas...      55  5e-13
 ref|XP_001316126.1| hypothetical protein TVAG_192020 [Tric...      55  5e-13
 ref|XP_001311049.1| surface antigen BspA-like [Trichomonas...      55  5e-13
 ref|XP_001305579.1| conserved hypothetical protein [Tricho...      55  5e-13
 ref|XP_001323870.1| surface antigen BspA-like [Trichomonas...      54  7e-13
 ref|XP_001583194.1| conserved hypothetical protein [Tricho...      54  9e-13
 ref|XP_001322763.1| surface antigen Bsp, putative [Trichom...      54  1e-12
 ref|XP_001304980.1| surface antigen BspA-like [Trichomonas...      54  1e-12
 ref|XP_001580665.1| surface antigen BspA-like [Trichomonas...      53  2e-12
 ref|XP_001317521.1| surface antigen BspA-like [Trichomonas...      53  2e-12
 ref|XP_001302536.1| surface antigen BspA-like [Trichomonas...      53  2e-12
 ref|XP_001327175.1| surface antigen BspA-like [Trichomonas...      53  2e-12
 ref|XP_001302594.1| surface antigen BspA-like [Trichomonas...      52  3e-12
 ref|XP_001583035.1| hypothetical protein TVAG_456840 [Tric...      52  4e-12
 ref|XP_001316575.1| hypothetical protein TVAG_070020 [Tric...      52  4e-12
 ref|XP_001317275.1| surface antigen BspA-like [Trichomonas...      52  4e-12
 ref|XP_001312366.1| surface antigen BspA-like [Trichomonas...      52  4e-12
 ref|XP_001306747.1| surface antigen BspA-like [Trichomonas...      52  4e-12
 ref|XP_001309841.1| surface antigen BspA-like [Trichomonas...      51  6e-12
 ref|XP_001306309.1| surface antigen BspA-like [Trichomonas...      51  6e-12
 ref|XP_001323659.1| surface antigen BspA-like [Trichomonas...      51  1e-11
 ref|XP_001322332.1| hypothetical protein TVAG_274720 [Tric...      51  1e-11
 ref|XP_001326264.1| surface antigen BspA-like [Trichomonas...      50  1e-11
 ref|XP_001309066.1| surface antigen BspA-like [Trichomonas...      50  1e-11
 ref|XP_001308225.1| surface antigen BspA-like [Trichomonas...      50  1e-11
 ref|XP_001286695.1| surface antigen BspA-like [Trichomonas...      50  1e-11
 ref|XP_001579044.1| Leucine Rich Repeat family protein [Tr...      50  2e-11
 ref|XP_001302270.1| hypothetical protein TVAG_574760 [Tric...      50  2e-11
 ref|XP_001584313.1| surface antigen BspA-like [Trichomonas...      49  2e-11
 ref|XP_001316621.1| surface antigen BspA-like [Trichomonas...      49  3e-11
 ref|XP_001306980.1| surface antigen BspA-like [Trichomonas...      48  5e-11
 ref|XP_001582516.1| hypothetical protein TVAG_012950 [Tric...      48  6e-11
 ref|XP_001329822.1| surface antigen BspA-like [Trichomonas...      48  6e-11
 ref|XP_001305201.1| surface antigen BspA-like [Trichomonas...      48  6e-11
 ref|XP_001311621.1| hypothetical protein TVAG_333550 [Tric...      47  8e-11
 ref|XP_001314764.1| surface antigen BspA-like [Trichomonas...      47  1e-10
 ref|XP_001308117.1| surface antigen BspA-like [Trichomonas...      47  1e-10
 ref|XP_001323860.1| hypothetical protein TVAG_158640 [Tric...      47  1e-10
 ref|XP_001297260.1| surface antigen BspA-like [Trichomonas...      47  1e-10
 ref|XP_001323863.1| surface antigen BspA-like [Trichomonas...      46  2e-10
 ref|XP_001312365.1| hypothetical protein TVAG_408450 [Tric...      46  2e-10
 ref|XP_001326468.1| surface antigen BspA-like [Trichomonas...      46  2e-10
 ref|XP_001309231.1| Leucine Rich Repeat family protein [Tr...      46  3e-10
 ref|XP_001581507.1| surface antigen BspA-like [Trichomonas...      45  5e-10
 ref|XP_001579305.1| surface antigen BspA-like [Trichomonas...      45  5e-10
 ref|XP_001583195.1| surface antigen BspA-like [Trichomonas...      44  7e-10
 ref|XP_001584394.1| cell surface protein, putative [Tricho...      44  7e-10
 ref|XP_001311339.1| hypothetical protein TVAG_125530 [Tric...      44  9e-10
 ref|XP_001309068.1| surface antigen BspA-like [Trichomonas...      44  9e-10
 ref|XP_001327006.1| surface antigen BspA-like [Trichomonas...      44  1e-09
 ref|XP_001306608.1| Leucine Rich Repeat family protein [Tr...      43  1e-09
 ref|XP_001330639.1| cell surface protein, putative [Tricho...      43  2e-09
 ref|XP_001313865.1| hypothetical protein TVAG_493090 [Tric...      43  2e-09
 ref|XP_001313120.1| hypothetical protein TVAG_007210 [Tric...      43  2e-09
 ref|XP_001301144.1| hypothetical protein TVAG_043300 [Tric...      43  2e-09
 ref|XP_001298588.1| hypothetical protein TVAG_145120 [Tric...      43  2e-09
 ref|XP_001321494.1| surface antigen BspA-like [Trichomonas...      42  4e-09
 ref|XP_001311473.1| surface antigen BspA-like [Trichomonas...      42  4e-09
 ref|XP_001321506.1| hypothetical protein TVAG_133410 [Tric...      41  5e-09
 ref|XP_001316692.1| surface antigen BspA-like [Trichomonas...      41  7e-09
 ref|XP_001330631.1| surface antigen Bsp, putative [Trichom...      41  7e-09
 ref|XP_001313444.1| hypothetical protein TVAG_350110 [Tric...      41  7e-09
 ref|XP_001303564.1| surface antigen BspA-like [Trichomonas...      41  7e-09
 ref|XP_001327007.1| Leucine Rich Repeat family protein [Tr...      40  1e-08
 ref|XP_001321501.1| hypothetical protein TVAG_133360 [Tric...      40  1e-08
 ref|XP_001321504.1| surface antigen BspA-like [Trichomonas...      40  2e-08
 ref|XP_001315805.1| hypothetical protein TVAG_042340 [Tric...      39  3e-08
 ref|XP_001298123.1| hypothetical protein TVAG_043250 [Tric...      39  3e-08
 ref|XP_001328766.1| cell surface protein, putative [Tricho...      38  4e-08
 ref|XP_001323868.1| surface antigen BspA-like [Trichomonas...      38  6e-08
 ref|XP_001328762.1| surface antigen BspA-like [Trichomonas...      38  6e-08
 ref|XP_001319648.1| hypothetical protein TVAG_419670 [Tric...      38  7e-08
 ref|XP_001318798.1| surface antigen BspA-like [Trichomonas...      37  1e-07
 ref|XP_001328755.1| surface antigen BspA-like [Trichomonas...      37  1e-07
 ref|XP_001312376.1| hypothetical protein TVAG_052890 [Tric...      37  1e-07
 ref|XP_001582616.1| hypothetical protein TVAG_013970 [Tric...      37  2e-07
 ref|XP_001322414.1| conserved hypothetical protein [Tricho...      36  3e-07
 ref|XP_001328763.1| surface antigen BspA-like [Trichomonas...      35  5e-07
 ref|XP_001322364.1| surface antigen BspA-like [Trichomonas...      35  6e-07
 ref|XP_001312048.1| surface antigen BspA-like [Trichomonas...      35  6e-07
 ref|XP_001306943.1| hypothetical protein TVAG_082200 [Tric...      34  8e-07
 ref|XP_001579824.1| surface antigen BspA-like [Trichomonas...      33  2e-06
 ref|XP_001582999.1| hypothetical protein TVAG_456480 [Tric...      33  2e-06
 ref|XP_001323987.1| surface antigen BspA-like [Trichomonas...      33  2e-06
 ref|XP_001325359.1| Leucine Rich Repeat family protein [Tr...      33  2e-06
 ref|XP_001582703.1| conserved hypothetical protein [Tricho...      32  3e-06
 ref|XP_001583566.1| surface antigen BspA-like [Trichomonas...      32  3e-06
 ref|XP_001319646.1| hypothetical protein TVAG_419650 [Tric...      32  3e-06
 ref|XP_001325360.1| surface antigen BspA-like [Trichomonas...      32  4e-06
 ref|XP_001318637.1| choline binding protein, putative [Tri...      32  4e-06
 ref|XP_001302666.1| surface antigen Bsp, putative [Trichom...      32  4e-06
 ref|XP_001324621.1| surface antigen BspA-like [Trichomonas...      32  5e-06
 ref|XP_001308226.1| surface antigen BspA-like [Trichomonas...      32  5e-06
 ref|XP_001312719.1| surface antigen BspA-like [Trichomonas...      31  6e-06
 ref|XP_001580218.1| surface antigen BspA-like [Trichomonas...      31  8e-06
 ref|XP_001322410.1| surface antigen BspA-like [Trichomonas...      31  8e-06
 ref|XP_001307708.1| surface antigen BspA-like [Trichomonas...      31  8e-06
 ref|XP_001316263.1| surface antigen BspA-like [Trichomonas...      31  1e-05
 ref|XP_001322572.1| hypothetical protein TVAG_109330 [Tric...      30  1e-05
 ref|XP_001321228.1| surface antigen BspA-like [Trichomonas...      30  1e-05
 ref|XP_001316381.1| conserved hypothetical protein [Tricho...      30  1e-05
 ref|XP_001579236.1| Leucine Rich Repeat family protein [Tr...      30  2e-05
 ref|XP_001330674.1| surface antigen BspA-like [Trichomonas...      30  2e-05
 ref|XP_001301143.1| surface antigen BspA-like [Trichomonas...      29  2e-05
 ref|XP_001328158.1| surface antigen BspA-like [Trichomonas...      29  4e-05
 ref|XP_001328162.1| hypothetical protein TVAG_165670 [Tric...      29  4e-05
 ref|XP_001305685.1| cell surface protein, putative [Tricho...      28  5e-05
 ref|XP_001579565.1| conserved hypothetical protein [Tricho...      28  8e-05
 ref|XP_001582896.1| surface antigen BspA-like [Trichomonas...      28  8e-05
 ref|XP_001307705.1| hypothetical protein TVAG_058650 [Tric...      27  1e-04
 ref|XP_001321488.1| cell surface protein, putative [Tricho...      27  1e-04
 ref|XP_001321492.1| cell surface protein, putative [Tricho...      27  1e-04
 ref|XP_001318169.1| conserved hypothetical protein [Tricho...      27  1e-04
 ref|XP_001323934.1| hypothetical protein TVAG_488130 [Tric...      26  2e-04
 ref|XP_001315613.1| surface antigen BspA-like [Trichomonas...      26  2e-04
 ref|XP_001312182.1| conserved hypothetical protein [Tricho...      26  2e-04
 ref|XP_001307333.1| hypothetical protein TVAG_194890 [Tric...      26  2e-04
 ref|XP_001328290.1| hypothetical protein TVAG_278330 [Tric...      26  2e-04
 ref|XP_001321496.1| surface antigen BspA-like [Trichomonas...      26  2e-04
 ref|XP_001308403.1| surface antigen BspA-like [Trichomonas...      26  2e-04
 ref|XP_001303020.1| surface antigen BspA-like [Trichomonas...      26  2e-04
 ref|XP_001581618.1| conserved hypothetical protein [Tricho...      26  3e-04
 ref|XP_001313013.1| surface antigen BspA-like [Trichomonas...      26  3e-04
 ref|XP_001326631.1| surface antigen Bsp, putative [Trichom...      25  4e-04
 ref|XP_001311694.1| surface antigen BspA-like [Trichomonas...      25  4e-04
 ref|XP_001308928.1| surface antigen BspA-like [Trichomonas...      25  4e-04
 ref|XP_001316253.1| surface antigen Bsp, putative [Trichom...      25  5e-04
 ref|XP_001307916.1| surface antigen BspA-like [Trichomonas...      25  5e-04
 ref|XP_001306188.1| surface antigen BspA-like [Trichomonas...      25  5e-04
 ref|XP_001308119.1| surface antigen BspA-like [Trichomonas...      25  6e-04
 ref|XP_001581225.1| surface antigen BspA-like [Trichomonas...      24  8e-04

  Methanosarcina barkeri str. Fusaro [euryarchaeotes] taxid 269797
 ref|YP_305509.1| cell surface protein [Methanosarcina bark...     251  4e-72
 ref|YP_305504.1| cell surface protein [Methanosarcina bark...     206  1e-58

  Eubacterium siraeum DSM 15702 [firmicutes] taxid 428128
 ref|ZP_02423777.1| hypothetical protein EUBSIR_02655 [Euba...     249  1e-71
 ref|ZP_02423818.1| hypothetical protein EUBSIR_02697 [Euba...     239  2e-68
 ref|ZP_02421573.1| hypothetical protein EUBSIR_00400 [Euba...     206  1e-58
 ref|ZP_02421667.1| hypothetical protein EUBSIR_00498 [Euba...     190  9e-54
 ref|ZP_02422346.1| hypothetical protein EUBSIR_01193 [Euba...     140  2e-38
 ref|ZP_02423885.1| hypothetical protein EUBSIR_02767 [Euba...     104  6e-28
 ref|ZP_02422266.1| hypothetical protein EUBSIR_01108 [Euba...      49  2e-11

  Methanosarcina acetivorans C2A [euryarchaeotes] taxid 188937
 ref|NP_619153.1| cell surface protein [Methanosarcina acet...     244  8e-70
 ref|NP_619149.1| cell surface protein [Methanosarcina acet...     209  1e-59
 ref|NP_619156.1| cell surface protein [Methanosarcina acet...     199  3e-56

  Flavobacterium psychrophilum JIP02/86 [CFB group bacteria] taxid 402612
 ref|YP_001295104.1| cell surface leucine-rich repeat-conta...     236  2e-67
 ref|YP_001295109.1| cell surface leucine-rich repeat-conta...     235  2e-67
 ref|YP_001295111.1| cell surface leucine-rich repeat-conta...     218  3e-62
 ref|YP_001295103.1| cell surface leucine-rich repeat-conta...     188  4e-53
 ref|YP_001295115.1| cell surface leucine-rich repeat-conta...     165  4e-46
 ref|YP_001295102.1| cell surface leucine-rich repeat-conta...     164  8e-46
 ref|YP_001295108.1| cell surface leucine-rich repeat-conta...     148  4e-41
 ref|YP_001295113.1| cell surface leucine-rich repeat-conta...     130  1e-35
 ref|YP_001295110.1| cell surface leucine-rich repeat-conta...     124  9e-34
 ref|YP_001295105.1| cell surface leucine-rich repeat-conta...     123  1e-33
 ref|YP_001295112.1| cell surface leucine-rich repeat-conta...     119  2e-32
 ref|YP_001295116.1| cell surface leucine-rich repeat-conta...     106  3e-28
 ref|YP_001295106.1| cell surface leucine-rich repeat-conta...      88  5e-23
 ref|YP_001295107.1| cell surface leucine-rich repeat-conta...      67  1e-16
 ref|YP_001295114.1| cell surface leucine-rich repeat-conta...      55  4e-13

  Clostridium leptum DSM 753 [firmicutes] taxid 428125
 ref|ZP_02081532.1| hypothetical protein CLOLEP_03013 [Clos...     229  2e-65

  Entamoeba dispar SAW760 [eukaryotes] taxid 370354
 ref|XP_001740560.1| hypothetical protein, conserved [Entam...     205  4e-58
 ref|XP_001734308.1| hypothetical protein, conserved [Entam...     189  2e-53
 ref|XP_001739293.1| hypothetical protein, conserved [Entam...     153  1e-42
 ref|XP_001735072.1| hypothetical protein EDI_342150 [Entam...     143  1e-39
 ref|XP_001741344.1| hypothetical protein, conserved [Entam...     141  7e-39
 ref|XP_001740398.1| hypothetical protein, conserved [Entam...     132  3e-36
 ref|XP_001733769.1| hypothetical protein, conserved [Entam...     126  2e-34
 ref|XP_001736642.1| hypothetical protein, conserved [Entam...     121  8e-33
 ref|XP_001734705.1| hypothetical protein, conserved [Entam...     120  1e-32
 ref|XP_001737239.1| hypothetical protein, conserved [Entam...     120  1e-32
 ref|XP_001733625.1| hypothetical protein, conserved [Entam...     119  2e-32
 ref|XP_001737480.1| hypothetical protein EDI_100620 [Entam...     116  2e-31
 ref|XP_001742001.1| hypothetical protein, conserved [Entam...     115  4e-31
 ref|XP_001737131.1| hypothetical protein EDI_231200 [Entam...     112  4e-30
 ref|XP_001738090.1| hypothetical protein, conserved [Entam...     109  2e-29
 ref|XP_001738868.1| hypothetical protein, conserved [Entam...     101  5e-27
 ref|XP_001739661.1| hypothetical protein, conserved [Entam...      98  5e-26
 ref|XP_001736492.1| hypothetical protein, conserved [Entam...      96  2e-25
 ref|XP_001735302.1| hypothetical protein, conserved [Entam...      96  3e-25
 ref|XP_001738317.1| hypothetical protein, conserved [Entam...      90  1e-23
 ref|XP_001733937.1| hypothetical protein EDI_233880 [Entam...      84  8e-22
 ref|XP_001734793.1| hypothetical protein, conserved [Entam...      84  1e-21
 ref|XP_001738044.1| hypothetical protein, conserved [Entam...      77  9e-20
 ref|XP_001738458.1| hypothetical protein, conserved [Entam...      77  9e-20
 ref|XP_001741630.1| hypothetical protein, conserved [Entam...      77  1e-19
 ref|XP_001740464.1| hypothetical protein, conserved [Entam...      65  6e-16
 ref|XP_001741957.1| hypothetical protein, conserved [Entam...      55  4e-13
 ref|XP_001733869.1| hypothetical protein, conserved [Entam...      54  9e-13
 ref|XP_001733629.1| hypothetical protein, conserved [Entam...      48  5e-11
 ref|XP_001737783.1| hypothetical protein, conserved [Entam...      45  4e-10
 ref|XP_001736600.1| hypothetical protein, conserved [Entam...      32  3e-06
 ref|XP_001733492.1| hypothetical protein EDI_323110 [Entam...      31  1e-05
 ref|XP_001734315.1| hypothetical protein, conserved [Entam...      28  8e-05
 ref|XP_001740706.1| hypothetical protein EDI_303900 [Entam...      27  1e-04

  bacterium Ellin514 [verrucomicrobia] taxid 320771
 ref|ZP_02967646.1| cell surface protein [bacterium Ellin514]      199  2e-56
 ref|ZP_02970487.1| hypothetical protein CflavDRAFT_5610 [b...     134  7e-37

  Syntrophomonas wolfei subsp. wolfei str. Goettingen [firmicutes] taxid 335541
 ref|YP_754057.1| leucine-rich repeat-containing protein [S...     193  1e-54

  Clostridium spiroforme DSM 1552 [firmicutes] taxid 428126
 ref|ZP_02868475.1| hypothetical protein CLOSPI_02317 [Clos...     178  4e-50

  Clostridium beijerinckii NCIMB 8052 [firmicutes] taxid 290402
 ref|YP_001310293.1| cell wall binding repeat-containing pr...     174  6e-49
 ref|YP_001310322.1| cell wall binding repeat-containing pr...     138  4e-38
 ref|YP_001309881.1| cell wall binding repeat-containing pr...     109  2e-29
 ref|YP_001308736.1| fibronectin, type III domain-containin...      81  6e-21
 ref|YP_001309903.1| cell wall binding repeat-containing pr...      32  4e-06

  Epulopiscium sp. 'N.t. morphotype B' [firmicutes] taxid 420336
 ref|ZP_02693515.1| cell surface protein [Epulopiscium sp. ...     174  7e-49
 ref|ZP_02692178.1| Probable cell surface protein (Leucine-...     172  4e-48
 ref|ZP_02693548.1| cell surface protein [Epulopiscium sp. ...     169  2e-47
 ref|ZP_02691993.1| Probable cell surface protein (Leucine-...     137  8e-38
 ref|ZP_02693554.1| Probable cell surface protein (Leucine-...     134  5e-37
 ref|ZP_02693390.1| cell surface protein [Epulopiscium sp. ...     124  9e-34
 ref|ZP_02691806.1| cell surface protein [Epulopiscium sp. ...     121  8e-33
 ref|ZP_02692842.1| Probable cell surface protein (Leucine-...     114  9e-31
 ref|ZP_02691805.1| cell surface protein [Epulopiscium sp. ...     113  1e-30
 ref|ZP_02693356.1| cell surface protein [Epulopiscium sp. ...     111  8e-30
 ref|ZP_02692410.1| cell surface protein [Epulopiscium sp. ...     108  7e-29
 ref|ZP_02693389.1| cell surface protein [Epulopiscium sp. ...      96  2e-25
 ref|ZP_02692841.1| cell surface protein [Epulopiscium sp. ...      95  3e-25
 ref|ZP_02693326.1| cell surface protein [Epulopiscium sp. ...      91  5e-24
 ref|ZP_02691807.1| Leucine-rich repeat (LRR) protein-like ...      86  3e-22
 ref|ZP_02693025.1| cell surface protein [Epulopiscium sp. ...      84  1e-21
 ref|ZP_02693306.1| Probable cell surface protein (Leucine-...      80  1e-20
 ref|ZP_02692843.1| Probable cell surface protein (Leucine-...      76  3e-19
 ref|ZP_02692800.1| cell surface protein [Epulopiscium sp. ...      35  6e-07

  Victivallis vadensis ATCC BAA-548 [bacteria] taxid 340101
 ref|ZP_01923169.1| cell surface protein [Victivallis vaden...     167  7e-47
 ref|ZP_01923170.1| hypothetical protein VvadDRAFT_1399 [Vi...      99  2e-26
 ref|ZP_01922308.1| hypothetical protein VvadDRAFT_0933 [Vi...      38  4e-08

  Shewanella pealeana ATCC 700345 [g-proteobacteria] taxid 398579
 ref|YP_001500360.1| FNIP [Shewanella pealeana ATCC 700345]        155  3e-43
 ref|YP_001502689.1| TPR repeat-containing protein [Shewane...     128  6e-35
 ref|YP_001503317.1| hypothetical protein Spea_3469 [Shewan...      77  1e-19

  Anaerofustis stercorihominis DSM 17244 [firmicutes] taxid 445971
 ref|ZP_02861219.1| hypothetical protein ANASTE_00419 [Anae...     153  2e-42
 ref|ZP_02861046.1| hypothetical protein ANASTE_00239 [Anae...     140  9e-39
 ref|ZP_02860961.1| hypothetical protein ANASTE_00152 [Anae...     137  8e-38
 ref|ZP_02860940.1| hypothetical protein ANASTE_00131 [Anae...     129  2e-35
 ref|ZP_02861139.1| hypothetical protein ANASTE_00332 [Anae...     105  3e-28
 ref|ZP_02861690.1| hypothetical protein ANASTE_00900 [Anae...     101  6e-27
 ref|ZP_02862016.1| hypothetical protein ANASTE_01229 [Anae...      94  1e-24
 ref|ZP_02862872.1| hypothetical protein ANASTE_02099 [Anae...      90  1e-23
 ref|ZP_02861865.1| hypothetical protein ANASTE_01075 [Anae...      78  5e-20
 ref|ZP_02861981.1| hypothetical protein ANASTE_01194 [Anae...      56  2e-13
 ref|ZP_02862435.1| hypothetical protein ANASTE_01650 [Anae...      29  3e-05
 ref|ZP_02861123.1| hypothetical protein ANASTE_00316 [Anae...      29  4e-05

  Bacteroides fragilis NCTC 9343 [CFB group bacteria] taxid 272559
 ref|YP_213662.1| surface protein [Bacteroides fragilis NCT...     148  3e-41
 ref|YP_211343.1| putative surface antigen [Bacteroides fra...      78  7e-20

  Treponema denticola ATCC 35405 [spirochetes] taxid 243275
 ref|NP_972265.1| leucine rich repeat domain-containing pro...     146  1e-40
 ref|NP_972858.1| surface antigen BspA, putative [Treponema...     135  3e-37
 ref|NP_972561.1| surface protein, putative [Treponema dent...     119  3e-32
 ref|NP_971188.1| surface protein, putative [Treponema dent...      84  1e-21
 ref|NP_973041.1| surface protein, putative [Treponema dent...      51  8e-12

  Alistipes putredinis DSM 17216 [CFB group bacteria] taxid 445970
 ref|ZP_02426114.1| hypothetical protein ALIPUT_02273 [Alis...     146  2e-40
 ref|ZP_02424091.1| hypothetical protein ALIPUT_00206 [Alis...     131  6e-36
 ref|ZP_02424866.1| hypothetical protein ALIPUT_00999 [Alis...      81  6e-21
 ref|ZP_02423935.1| hypothetical protein ALIPUT_00049 [Alis...      76  3e-19
 ref|ZP_02423940.1| hypothetical protein ALIPUT_00054 [Alis...      70  1e-17
 ref|ZP_02425898.1| hypothetical protein ALIPUT_02055 [Alis...      56  2e-13

  Entamoeba histolytica HM-1:IMSS [eukaryotes] taxid 294381
 ref|XP_656869.1| leucine rich repeat protein, BspA family ...     143  1e-39
 ref|XP_652287.1| leucine rich repeat protein, BspA family ...     139  3e-38
 ref|XP_655312.1| leucine rich repeat protein, BspA family ...     136  1e-37
 ref|XP_652730.1| leucine rich repeat protein, BspA family ...     134  9e-37
 ref|XP_657437.1| hypothetical protein EHI_151330 [Entamoeb...     129  2e-35
 ref|XP_650788.1| leucine rich repeat protein, BspA family ...     124  7e-34
 ref|XP_653754.1| leucine rich repeat protein, BspA family ...     118  4e-32
 ref|XP_653526.1| leucine rich repeat protein, BspA family ...     117  9e-32
 ref|XP_654709.1| leucine rich repeat protein, BspA family ...     117  1e-31
 ref|XP_651312.1| leucine rich repeat protein, BspA family ...     115  3e-31
 ref|XP_654062.1| leucine rich repeat protein, BspA family ...     111  6e-30
 ref|XP_657493.2| leucine rich repeat protein, BspA family ...     104  1e-27
 ref|XP_652255.1| leucine rich repeat protein, BspA family ...     103  1e-27
 ref|XP_652531.2| leucine rich repeat protein, BspA family ...     103  2e-27
 ref|XP_653459.2| leucine rich repeat protein, BspA family ...      99  2e-26
 ref|XP_649377.1| leucine rich repeat protein, BspA family ...      99  2e-26
 ref|XP_657020.2| leucine rich repeat protein, BspA family ...      95  3e-25
 ref|XP_001913739.1| hypothetical protein EHI_079970 [Entam...      92  3e-24
 ref|XP_654274.1| leucine rich repeat protein, BspA family ...      90  1e-23
 ref|XP_649473.2| leucine rich repeat protein, BspA family ...      87  1e-22
 ref|XP_652986.2| leucine rich repeat protein, BspA family ...      86  2e-22
 ref|XP_654256.1| leucine rich repeat protein, BspA family ...      86  2e-22
 ref|XP_648581.1| leucine rich repeat protein, BspA family ...      79  2e-20
 ref|XP_657078.1| leucine rich repeat protein, BspA family ...      77  1e-19
 ref|XP_649048.1| leucine rich repeat protein, BspA family ...      64  7e-16
 ref|XP_652183.1| leucine rich repeat protein, BspA family ...      56  2e-13
 ref|XP_651308.1| leucine rich repeat protein, BspA family ...      48  5e-11

  Synechococcus sp. WH 7805 [cyanobacteria] taxid 59931
 ref|ZP_01123009.1| cell surface protein [Synechococcus sp....     141  4e-39
 ref|ZP_01125124.1| cell surface protein [Synechococcus sp....     131  7e-36

  Ruminococcus torques ATCC 27756 [firmicutes] taxid 411460
 ref|ZP_01969043.1| hypothetical protein RUMTOR_02627 [Rumi...     138  4e-38
 ref|ZP_01969285.1| hypothetical protein RUMTOR_02870 [Rumi...     138  4e-38
 ref|ZP_01969039.1| hypothetical protein RUMTOR_02623 [Rumi...     113  1e-30
 ref|ZP_01969242.1| hypothetical protein RUMTOR_02827 [Rumi...      65  4e-16

  Bacteroides ovatus ATCC 8483 [CFB group bacteria] taxid 411476
 ref|ZP_02067577.1| hypothetical protein BACOVA_04585 [Bact...     134  5e-37
 ref|ZP_02063418.1| hypothetical protein BACOVA_00366 [Bact...     113  1e-30
 ref|ZP_02064596.1| hypothetical protein BACOVA_01565 [Bact...      59  3e-14
 ref|ZP_02063968.1| hypothetical protein BACOVA_00927 [Bact...      47  8e-11

  Methanococcus vannielii SB [euryarchaeotes] taxid 406327
 ref|YP_001322799.1| cell surface protein [Methanococcus va...     126  2e-34

  Clostridium sp. L2-50 [firmicutes] taxid 411489
 ref|ZP_02075562.1| hypothetical protein CLOL250_02338 [Clo...     117  9e-32
 ref|ZP_02075891.1| hypothetical protein CLOL250_02668 [Clo...     101  6e-27
 ref|ZP_02075561.1| hypothetical protein CLOL250_02337 [Clo...      99  2e-26
 ref|ZP_02073625.1| hypothetical protein CLOL250_00366 [Clo...      71  6e-18

  Clostridium butyricum 5521 [firmicutes] taxid 447214
 ref|ZP_02949217.1| surface protein PspC [Clostridium butyr...     112  3e-30
 ref|ZP_02950501.1| surface protein PspC [Clostridium butyr...      51  8e-12

  Coprococcus eutactus ATCC 27759 [firmicutes] taxid 411474
 ref|ZP_02205711.1| hypothetical protein COPEUT_00473 [Copr...     107  1e-28
 ref|ZP_02207819.1| hypothetical protein COPEUT_02644 [Copr...      64  7e-16
 ref|ZP_02206899.1| hypothetical protein COPEUT_01691 [Copr...      45  5e-10

  Photobacterium sp. SKA34 [g-proteobacteria] taxid 121723
 ref|ZP_01162683.1| cell surface protein [Photobacterium sp...     104  7e-28

  Kordia algicida OT-1 [CFB group bacteria] taxid 391587
 ref|ZP_02163681.1| cell surface protein [Kordia algicida O...     103  2e-27

  Flavobacteriales bacterium ALC-1 [CFB group bacteria] taxid 391603
 ref|ZP_02182632.1| cell surface protein [Flavobacteriales ...     101  8e-27

  Methanococcus maripaludis C7 [euryarchaeotes] taxid 426368
 ref|YP_001330886.1| TPR repeat-containing protein [Methano...     100  1e-26
 ref|YP_001329237.1| hypothetical protein MmarC7_0013 [Meth...      81  5e-21

  Bacteroides stercoris ATCC 43183 [CFB group bacteria] taxid 449673
 ref|ZP_02434396.1| hypothetical protein BACSTE_00622 [Bact...      99  2e-26
 ref|ZP_02435797.1| hypothetical protein BACSTE_02048 [Bact...      72  3e-18
 ref|ZP_02435130.1| hypothetical protein BACSTE_01368 [Bact...      52  4e-12
 ref|ZP_02434373.1| hypothetical protein BACSTE_00599 [Bact...      38  7e-08

  Clostridium phytofermentans ISDg [firmicutes] taxid 357809
 ref|YP_001560465.1| hypothetical protein Cphy_3373 [Clostr...      99  3e-26
 ref|YP_001559102.1| hypothetical protein Cphy_1995 [Clostr...      71  6e-18
 ref|YP_001559082.1| Ig domain-containing protein [Clostrid...      65  6e-16
 ref|YP_001558337.1| hypothetical protein Cphy_1220 [Clostr...      33  2e-06

  Clostridium bartlettii DSM 16795 [firmicutes] taxid 445973
 ref|ZP_02211019.1| hypothetical protein CLOBAR_00617 [Clos...      96  2e-25
 ref|ZP_02211373.1| hypothetical protein CLOBAR_00986 [Clos...      81  5e-21

  Desulfitobacterium hafniense Y51 [firmicutes] taxid 138119
 ref|YP_520089.1| hypothetical protein DSY3856 [Desulfitoba...      91  5e-24

  Eubacterium ventriosum ATCC 27560 [firmicutes] taxid 411463
 ref|ZP_02026084.1| hypothetical protein EUBVEN_01340 [Euba...      88  5e-23
 ref|ZP_02027186.1| hypothetical protein EUBVEN_02455 [Euba...      53  2e-12
 ref|ZP_02026466.1| hypothetical protein EUBVEN_01726 [Euba...      48  6e-11
 ref|ZP_02025833.1| hypothetical protein EUBVEN_01088 [Euba...      48  6e-11
 ref|ZP_02027368.1| hypothetical protein EUBVEN_02638 [Euba...      39  3e-08
 ref|ZP_02025927.1| hypothetical protein EUBVEN_01183 [Euba...      34  1e-06

  Shewanella halifaxensis HAW-EB4 [g-proteobacteria] taxid 458817
 ref|YP_001675765.1| hypothetical protein Shal_3565 [Shewan...      83  2e-21

  Treponema pallidum subsp. pallidum str. Nichols [spirochetes] taxid 243276
 ref|NP_218665.1| leucine-rich repeat-containing protein [T...      82  3e-21

  Treponema pallidum subsp. pallidum SS14 [spirochetes] taxid 455434
 ref|YP_001933230.1| leucine-rich repeat protein TpLRR [Tre...      82  3e-21

  Dorea formicigenerans ATCC 27755 [firmicutes] taxid 411461
 ref|ZP_02235444.1| hypothetical protein DORFOR_02330 [Dore...      80  1e-20

  Clostridium scindens ATCC 35704 [firmicutes] taxid 411468
 ref|ZP_02433176.1| hypothetical protein CLOSCI_03447 [Clos...      78  4e-20

  Bacteroides fragilis YCH46 [CFB group bacteria] taxid 295405
 ref|YP_098979.1| putative cell surface antigen [Bacteroide...      77  1e-19

  Bacteroides coprocola DSM 17136 [CFB group bacteria] taxid 470145
 ref|ZP_03008989.1| hypothetical protein BACCOP_00841 [Bact...      73  2e-18
 ref|ZP_03010628.1| hypothetical protein BACCOP_02509 [Bact...      38  7e-08

  Clostridium botulinum A3 str. Loch Maree [firmicutes] taxid 498214
 ref|YP_001785718.1| putative cell surface protein [Clostri...      73  2e-18

  Clostridium sporogenes ATCC 15579 [firmicutes] taxid 471871
 ref|ZP_02993488.1| hypothetical protein CLOSPO_00560 [Clos...      73  2e-18

  Clostridium botulinum B1 str. Okra [firmicutes] taxid 498213
 ref|YP_001780034.1| putative cell surface protein [Clostri...      73  2e-18

  Clostridium botulinum Bf [firmicutes] taxid 445336
 ref|ZP_02615908.1| putative cell surface protein [Clostrid...      72  3e-18

  Methanococcus maripaludis C6 [euryarchaeotes] taxid 444158
 ref|YP_001548287.1| hypothetical protein MmarC6_0234 [Meth...      72  3e-18
 ref|YP_001548288.1| hypothetical protein MmarC6_0235 [Meth...      36  2e-07

  Clostridium botulinum A str. ATCC 3502 [firmicutes] taxid 413999
 ref|YP_001252924.1| cell surface protein [Clostridium botu...      71  5e-18

  Clostridium botulinum NCTC 2916 [firmicutes] taxid 445335
 ref|ZP_02612389.1| putative cell surface protein [Clostrid...      71  8e-18

  Clostridium botulinum F str. Langeland [firmicutes] taxid 441772
 ref|YP_001389750.1| putative cell surface protein [Clostri...      71  8e-18

  Bacteroides vulgatus ATCC 8482 [CFB group bacteria] taxid 435590
 ref|YP_001300444.1| hypothetical protein BVU_3191 [Bactero...      68  5e-17

  Ruminococcus obeum ATCC 29174 [firmicutes] taxid 411459
 ref|ZP_01963695.1| hypothetical protein RUMOBE_01418 [Rumi...      60  1e-14
 ref|ZP_01964882.1| hypothetical protein RUMOBE_02612 [Rumi...      55  4e-13
 ref|ZP_01964239.1| hypothetical protein RUMOBE_01963 [Rumi...      26  3e-04

  Bacteroides thetaiotaomicron VPI-5482 [CFB group bacteria] taxid 226186
 ref|NP_810809.1| putative cell surface antigen [Bacteroide...      59  2e-14

  Bacteroides caccae ATCC 43185 [CFB group bacteria] taxid 411901
 ref|ZP_01962054.1| hypothetical protein BACCAC_03700 [Bact...      59  3e-14

  Streptococcus pneumoniae SP195 [firmicutes] taxid 453363
 ref|ZP_02713402.1| choline binding protein PcpA [Streptoco...      55  4e-13

  Streptococcus pneumoniae CGSP14 [firmicutes] taxid 516950
 ref|YP_001836822.1| choline binding protein PcpA [Streptoc...      54  9e-13

  Streptococcus pneumoniae CDC0288-04 [firmicutes] taxid 453364
 ref|ZP_02715811.1| choline binding protein PcpA [Streptoco...      54  9e-13

  Streptococcus pneumoniae CDC3059-06 [firmicutes] taxid 453365
 ref|ZP_02718249.1| choline binding protein PcpA [Streptoco...      54  1e-12

  Actinomyces odontolyticus ATCC 17982 [high GC Gram+] taxid 411466
 ref|ZP_02043255.1| hypothetical protein ACTODO_00093 [Acti...      54  1e-12
 ref|ZP_02043254.1| hypothetical protein ACTODO_00092 [Acti...      53  2e-12

  Streptococcus pneumoniae SP23-BS72 [firmicutes] taxid 406563
 ref|ZP_01835022.1| choline binding protein PcpA [Streptoco...      54  1e-12

  Streptococcus pneumoniae SP6-BS73 [firmicutes] taxid 406557
 ref|ZP_01820191.1| choline binding protein PcpA [Streptoco...      54  1e-12

  Streptococcus pneumoniae SP19-BS75 [firmicutes] taxid 406562
 ref|ZP_01833146.1| choline binding protein PcpA [Streptoco...      54  1e-12

  Streptococcus pneumoniae CDC1873-00 [firmicutes] taxid 453362
 ref|ZP_02708989.1| choline binding protein PcpA [Streptoco...      54  1e-12

  Streptococcus pneumoniae CDC1087-00 [firmicutes] taxid 453361
 ref|ZP_02710750.1| choline binding protein PcpA [Streptoco...      54  1e-12

  Streptococcus pneumoniae Hungary19A-6 [firmicutes] taxid 487214
 ref|YP_001695497.1| choline binding protein PcpA [Streptoc...      54  1e-12

  Streptococcus pneumoniae SP18-BS74 [firmicutes] taxid 406561
 ref|ZP_01830569.1| ornithine carbamoyltransferase [Strepto...      54  1e-12

  Streptococcus pneumoniae SP11-BS70 [firmicutes] taxid 406559
 ref|ZP_01825578.1| ornithine carbamoyltransferase [Strepto...      54  1e-12

  Streptococcus pneumoniae MLV-016 [firmicutes] taxid 453366
 ref|ZP_02721913.1| choline binding protein PcpA [Streptoco...      54  1e-12

  Streptococcus pneumoniae TIGR4 [firmicutes] taxid 170187
 ref|ZP_01407682.1| hypothetical protein SpneT_02001904 [St...      54  1e-12
 ref|NP_346554.1| choline binding protein PcpA [Streptococc...      54  1e-12

  Streptococcus pneumoniae G54 [firmicutes] taxid 512566
 ref|YP_002038725.1| choline binding protein PcpA [Streptoc...      52  3e-12

  Acholeplasma laidlawii PG-8A [mycoplasmas] taxid 441768
 ref|YP_001620762.1| putative surface-anchored antigen, Bsp...      52  4e-12

  Lactococcus lactis subsp. lactis Il1403 [firmicutes] taxid 272623
 ref|NP_267773.1| hypothetical protein L58460 [Lactococcus ...      51  8e-12

  Streptococcus pneumoniae D39 [firmicutes] taxid 373153
 ref|YP_817353.1| choline binding protein PcpA [Streptococc...      50  1e-11

  Streptococcus pneumoniae R6 [firmicutes] taxid 171101
 ref|NP_359536.1| choline binding protein PcpA [Streptococc...      50  1e-11

  Fusobacterium nucleatum subsp. nucleatum ATCC 25586 [fusobacteria] taxid 190304
 ref|NP_602643.1| surface antigen [Fusobacterium nucleatum ...      49  3e-11
 ref|NP_603116.1| putative cytoplasmic protein [Fusobacteri...      31  6e-06

  Lactococcus lactis subsp. cremoris SK11 [firmicutes] taxid 272622
 ref|YP_809644.1| subtilisin-like serine protease [Lactococ...      48  5e-11

  Lactococcus lactis subsp. cremoris MG1363 [firmicutes] taxid 416870
 ref|YP_001032204.1| putative secreted protein [Lactococcus...      47  1e-10

  Lactobacillus casei BL23 [firmicutes] taxid 543734
 ref|YP_001989023.1| Putative uncharacterized protein [Lact...      46  3e-10

  Clostridium sp. SS2/1 [firmicutes] taxid 411484
 ref|ZP_02440143.1| hypothetical protein CLOSS21_02635 [Clo...      43  2e-09
 ref|ZP_02437743.1| hypothetical protein CLOSS21_00178 [Clo...      33  2e-06

  Faecalibacterium prausnitzii M21/2 [firmicutes] taxid 411485
 ref|ZP_02092261.1| hypothetical protein FAEPRAM212_02550 [...      43  2e-09

  Lactobacillus casei ATCC 334 [firmicutes] taxid 321967
 ref|YP_808047.1| adhesion exoprotein [Lactobacillus casei ...      42  4e-09

  Mycoplasma agalactiae PG2 [mycoplasmas] taxid 347257
 ref|YP_001256394.1| lipoprotein [Mycoplasma agalactiae PG2]        39  3e-08

  Bacteroides capillosus ATCC 29799 [CFB group bacteria] taxid 411467
 ref|ZP_02034978.1| hypothetical protein BACCAP_00569 [Bact...      38  7e-08
 ref|ZP_02035394.1| hypothetical protein BACCAP_00990 [Bact...      28  8e-05

  Streptococcus equi subsp. zooepidemicus MGCS10565 [firmicutes] taxid 552526
 ref|YP_002123438.1| cell surface protein, RBC-binding prot...      37  1e-07

  candidate division TM7 single-cell isolate TM7b [bacteria] taxid 447455
 ref|ZP_02520161.1| Probable cell surface protein (Leucine-...      33  2e-06

  Streptococcus agalactiae CJB111 [firmicutes] taxid 342617
 ref|ZP_00787671.1| reticulocyte binding protein [Streptoco...      33  2e-06

  Streptococcus agalactiae A909 [firmicutes] taxid 205921
 ref|YP_329139.1| hypothetical protein SAK_0502 [Streptococ...      32  3e-06

  Streptococcus agalactiae NEM316 [firmicutes] taxid 211110
 ref|NP_734924.1| hypothetical protein gbs0456 [Streptococc...      32  3e-06

  Streptococcus agalactiae H36B [firmicutes] taxid 342615
 ref|ZP_00783119.1| reticulocyte binding protein [Streptoco...      32  3e-06

  Streptococcus agalactiae 515 [firmicutes] taxid 342614
 ref|ZP_00790303.1| reticulocyte binding protein [Streptoco...      32  3e-06

  Streptococcus agalactiae COH1 [firmicutes] taxid 342616
 ref|ZP_00784420.1| reticulocyte binding protein [Streptoco...      32  5e-06

  Streptococcus agalactiae 2603V/R [firmicutes] taxid 208435
 ref|NP_687455.1| hypothetical protein SAG0421 [Streptococc...      32  5e-06

  Streptococcus agalactiae 18RS21 [firmicutes] taxid 342613
 ref|ZP_00780863.1| cell wall surface anchor family protein...      32  5e-06

  Streptococcus pneumoniae SP14-BS69 [firmicutes] taxid 406560
 ref|ZP_01827888.1| choline binding protein PcpA [Streptoco...      30  1e-05

  Fusobacterium nucleatum subsp. vincentii ATCC 49256 [fusobacteria] taxid 209882
 ref|ZP_00144289.1| Hypothetical Cytosolic Protein [Fusobac...      30  2e-05

  Anaerotruncus colihominis DSM 17241 [firmicutes] taxid 445972
 ref|ZP_02443492.1| hypothetical protein ANACOL_02805 [Anae...      29  4e-05

  Streptococcus pneumoniae SP9-BS68 [firmicutes] taxid 406558
 ref|ZP_01821787.1| choline binding protein PcpA [Streptoco...      28  5e-05
 ref|ZP_01821788.1| ornithine carbamoyltransferase [Strepto...      26  3e-04

  Finegoldia magna ATCC 29328 [firmicutes] taxid 334413
 ref|YP_001692266.1| putative chimeric erythrocyte-binding ...      28  6e-05
 ref|YP_001692763.1| putative N-acetylmuramoyl-L-alanine am...      25  5e-04

  Listeria monocytogenes FSL J2-064 [firmicutes] taxid 393122
 ref|ZP_02321344.1| cell wall surface anchor family protein...      28  6e-05

  Listeria monocytogenes FSL J1-175 [firmicutes] taxid 393118
 ref|ZP_02288082.1| cell wall surface anchor family protein...      28  6e-05

  Listeria monocytogenes FSL R2-503 [firmicutes] taxid 393125
 ref|ZP_02285626.1| cell wall surface anchor family protein...      28  6e-05

  Listeria monocytogenes FSL J1-194 [firmicutes] taxid 393117
 ref|ZP_02278283.1| cell wall surface anchor family protein...      28  6e-05

  Listeria monocytogenes HPB2262 [firmicutes] taxid 401650
 ref|ZP_01945104.1| hypothetical protein LMSG_00080 [Lister...      28  6e-05

  Listeria monocytogenes FSL N1-017 [firmicutes] taxid 393123
 ref|ZP_01928583.1| hypothetical protein LMHG_00875 [Lister...      28  6e-05

  Listeria monocytogenes str. 4b F2365 [firmicutes] taxid 265669
 ref|YP_015226.1| cell wall surface anchor family protein [...      28  8e-05

  Listeria monocytogenes str. 4b H7858 [firmicutes] taxid 267410
 ref|ZP_00230071.1| cell wall surface anchor family protein...      28  8e-05

  Streptococcus pyogenes MGAS10750 [firmicutes] taxid 370554
 ref|YP_603188.1| Putative surface protein [Streptococcus p...      27  1e-04
 ref|YP_602236.1| Cell surface protein [Streptococcus pyoge...      26  2e-04

  Streptococcus pyogenes str. Manfredo [firmicutes] taxid 160491
 ref|YP_001128699.1| putative surface-anchored protein [Str...      26  2e-04

  Streptococcus pyogenes MGAS10270 [firmicutes] taxid 370552
 ref|YP_598319.1| cell surface protein [Streptococcus pyoge...      26  2e-04

  Streptococcus pyogenes MGAS9429 [firmicutes] taxid 370551
 ref|YP_596438.1| cell surface protein [Streptococcus pyoge...      26  2e-04

  Streptococcus pyogenes MGAS2096 [firmicutes] taxid 370553
 ref|YP_600315.1| cell surface protein [Streptococcus pyoge...      26  2e-04

  Streptococcus pyogenes MGAS10394 [firmicutes] taxid 286636
 ref|YP_059988.1| cell surface protein [Streptococcus pyoge...      26  2e-04

  Streptococcus pyogenes MGAS6180 [firmicutes] taxid 319701
 ref|YP_280101.1| cell surface protein [Streptococcus pyoge...      26  2e-04

  Streptococcus pyogenes MGAS315 [firmicutes] taxid 198466
 ref|NP_664373.1| putative surface antigen [Streptococcus p...      26  2e-04

  Streptococcus pyogenes M1 GAS [firmicutes] taxid 160490
 ref|NP_269051.1| hypothetical protein SPy_0843 [Streptococ...      26  2e-04

  Streptococcus pyogenes MGAS5005 [firmicutes] taxid 293653
 ref|YP_282014.1| cell surface protein [Streptococcus pyoge...      26  2e-04

  Streptococcus pyogenes MGAS8232 [firmicutes] taxid 186103
 ref|NP_607054.1| hypothetical protein spyM18_0903 [Strepto...      26  2e-04

  Streptococcus pyogenes SSI-1 [firmicutes] taxid 193567
 ref|NP_802547.1| hypothetical protein SPs1285 [Streptococc...      26  2e-04

  Streptococcus pyogenes M49 591 [firmicutes] taxid 294934
 ref|ZP_00366186.1| COG0556: Helicase subunit of the DNA ex...      26  2e-04
```

---

**Taxonomy Report**

```
cellular organisms ..............................................  2609 hits  119 orgs [root]
. Eukaryota .....................................................  1729 hits    3 orgs 
. . Trichomonas vaginalis G3 ....................................  1570 hits    1 orgs [Parabasalidea; Trichomonada; Trichomonadida; Trichomonadidae; Trichomonadinae; Trichomonas; Trichomonas vaginalis]
. . Entamoeba ...................................................   159 hits    2 orgs [Amoebozoa; Entamoebidae]
. . . Entamoeba dispar SAW760 ...................................    92 hits    1 orgs [Entamoeba dispar]
. . . Entamoeba histolytica HM-1:IMSS ...........................    67 hits    1 orgs [Entamoeba histolytica]
. Euryarchaeota .................................................   102 hits    5 orgs [Archaea]
. . Methanosarcina ..............................................    67 hits    2 orgs [Methanomicrobia; Methanosarcinales; Methanosarcinaceae]
. . . Methanosarcina barkeri str. Fusaro ........................    28 hits    1 orgs [Methanosarcina barkeri]
. . . Methanosarcina acetivorans C2A ............................    39 hits    1 orgs [Methanosarcina acetivorans]
. . Methanococcus ...............................................    35 hits    3 orgs [Methanococci; Methanococcales; Methanococcaceae]
. . . Methanococcus vannielii SB ................................    12 hits    1 orgs [Methanococcus vannielii]
. . . Methanococcus maripaludis .................................    23 hits    2 orgs 
. . . . Methanococcus maripaludis C7 ............................    16 hits    1 orgs 
. . . . Methanococcus maripaludis C6 ............................     7 hits    1 orgs 
. Bacteria ......................................................   778 hits  111 orgs 
. . Firmicutes ..................................................   515 hits   83 orgs 
. . . Clostridiales .............................................   424 hits   28 orgs [Clostridia]
. . . . Eubacteriaceae ..........................................   113 hits    3 orgs 
. . . . . Eubacterium ...........................................    67 hits    2 orgs 
. . . . . . Eubacterium siraeum DSM 15702 .......................    54 hits    1 orgs [Eubacterium siraeum]
. . . . . . Eubacterium ventriosum ATCC 27560 ...................    13 hits    1 orgs [Eubacterium ventriosum]
. . . . . Anaerofustis stercorihominis DSM 17244 ................    46 hits    1 orgs [Anaerofustis; Anaerofustis stercorihominis]
. . . . Clostridium .............................................   178 hits   15 orgs [Clostridiaceae]
. . . . . Clostridium leptum DSM 753 ............................    14 hits    1 orgs [Clostridium leptum]
. . . . . Clostridium beijerinckii NCIMB 8052 ...................    15 hits    1 orgs [Clostridium beijerinckii]
. . . . . Clostridium sp. L2-50 .................................    13 hits    1 orgs 
. . . . . Clostridium butyricum 5521 ............................     6 hits    1 orgs [Clostridium butyricum]
. . . . . Clostridium phytofermentans ISDg ......................     9 hits    1 orgs [Clostridium phytofermentans]
. . . . . Clostridium bartlettii DSM 16795 ......................     6 hits    1 orgs [Clostridium bartlettii]
. . . . . Clostridium scindens ATCC 35704 .......................    12 hits    1 orgs [Clostridium scindens]
. . . . . Clostridium botulinum .................................    81 hits    6 orgs 
. . . . . . Clostridium botulinum A .............................    25 hits    2 orgs 
. . . . . . . Clostridium botulinum A3 str. Loch Maree ..........    12 hits    1 orgs 
. . . . . . . Clostridium botulinum A str. ATCC 3502 ............    13 hits    1 orgs 
. . . . . . Clostridium botulinum B1 str. Okra ..................    12 hits    1 orgs [Clostridium botulinum B]
. . . . . . Clostridium botulinum Bf ............................    13 hits    1 orgs 
. . . . . . Clostridium botulinum NCTC 2916 .....................    13 hits    1 orgs 
. . . . . . Clostridium botulinum F str. Langeland ..............    18 hits    1 orgs [Clostridium botulinum F]
. . . . . Clostridium sporogenes ATCC 15579 .....................    18 hits    1 orgs [Clostridium sporogenes]
. . . . . Clostridium sp. SS2/1 .................................     4 hits    1 orgs 
. . . . Syntrophomonas wolfei subsp. wolfei str. Goettingen .....     9 hits    1 orgs [Syntrophomonadaceae; Syntrophomonas; Syntrophomonas wolfei; Syntrophomonas wolfei subsp. wolfei]
. . . . Epulopiscium sp. 'N.t. morphotype B' ....................    73 hits    1 orgs [unclassified Clostridiales; Epulopiscium]
. . . . Ruminococcaceae .........................................    29 hits    4 orgs 
. . . . . Ruminococcus ..........................................    25 hits    2 orgs 
. . . . . . Ruminococcus torques ATCC 27756 .....................    18 hits    1 orgs [Ruminococcus torques]
. . . . . . Ruminococcus obeum ATCC 29174 .......................     7 hits    1 orgs [Ruminococcus obeum]
. . . . . Faecalibacterium prausnitzii M21/2 ....................     3 hits    1 orgs [Faecalibacterium; Faecalibacterium prausnitzii]
. . . . . Anaerotruncus colihominis DSM 17241 ...................     1 hits    1 orgs [Anaerotruncus; Anaerotruncus colihominis]
. . . . Lachnospiraceae .........................................    10 hits    2 orgs 
. . . . . Coprococcus eutactus ATCC 27759 .......................     6 hits    1 orgs [Coprococcus; Coprococcus eutactus]
. . . . . Dorea formicigenerans ATCC 27755 ......................     4 hits    1 orgs [Dorea; Dorea formicigenerans]
. . . . Desulfitobacterium hafniense Y51 ........................     9 hits    1 orgs [Peptococcaceae; Desulfitobacterium; Desulfitobacterium hafniense]
. . . . Finegoldia magna ATCC 29328 .............................     3 hits    1 orgs [Clostridiales incertae sedis; Clostridiales Family XI. Incertae Sedis; Finegoldia; Finegoldia magna]
. . . Clostridium spiroforme DSM 1552 ...........................     4 hits    1 orgs [Erysipelotrichi; Erysipelotrichales; Erysipelotrichaceae; unclassified Erysipelotrichaceae; Clostridium spiroforme]
. . . Bacilli ...................................................    87 hits   54 orgs 
. . . . Lactobacillales .........................................    79 hits   46 orgs 
. . . . . Streptococcaceae ......................................    71 hits   44 orgs 
. . . . . . Streptococcus .......................................    64 hits   41 orgs 
. . . . . . . Streptococcus pneumoniae ..........................    37 hits   19 orgs 
. . . . . . . . Streptococcus pneumoniae SP195 ..................     2 hits    1 orgs 
. . . . . . . . Streptococcus pneumoniae CGSP14 .................     2 hits    1 orgs 
. . . . . . . . Streptococcus pneumoniae CDC0288-04 .............     2 hits    1 orgs 
. . . . . . . . Streptococcus pneumoniae CDC3059-06 .............     2 hits    1 orgs 
. . . . . . . . Streptococcus pneumoniae SP23-BS72 ..............     2 hits    1 orgs 
. . . . . . . . Streptococcus pneumoniae SP6-BS73 ...............     2 hits    1 orgs 
. . . . . . . . Streptococcus pneumoniae SP19-BS75 ..............     2 hits    1 orgs 
. . . . . . . . Streptococcus pneumoniae CDC1873-00 .............     2 hits    1 orgs 
. . . . . . . . Streptococcus pneumoniae CDC1087-00 .............     2 hits    1 orgs 
. . . . . . . . Streptococcus pneumoniae Hungary19A-6 ...........     2 hits    1 orgs 
. . . . . . . . Streptococcus pneumoniae SP18-BS74 ..............     2 hits    1 orgs 
. . . . . . . . Streptococcus pneumoniae SP11-BS70 ..............     2 hits    1 orgs 
. . . . . . . . Streptococcus pneumoniae MLV-016 ................     2 hits    1 orgs 
. . . . . . . . Streptococcus pneumoniae TIGR4 ..................     4 hits    1 orgs 
. . . . . . . . Streptococcus pneumoniae G54 ....................     2 hits    1 orgs 
. . . . . . . . Streptococcus pneumoniae D39 ....................     1 hits    1 orgs 
. . . . . . . . Streptococcus pneumoniae R6 .....................     1 hits    1 orgs 
. . . . . . . . Streptococcus pneumoniae SP14-BS69 ..............     1 hits    1 orgs 
. . . . . . . . Streptococcus pneumoniae SP9-BS68 ...............     2 hits    1 orgs 
. . . . . . . Streptococcus equi subsp. zooepidemicus MGCS10565 .     3 hits    1 orgs [Streptococcus dysgalactiae group; Streptococcus equi; Streptococcus equi subsp. zooepidemicus]
. . . . . . . Streptococcus agalactiae ..........................     9 hits    8 orgs 
. . . . . . . . Streptococcus agalactiae CJB111 .................     2 hits    1 orgs 
. . . . . . . . Streptococcus agalactiae A909 ...................     1 hits    1 orgs [Streptococcus agalactiae serogroup Ia]
. . . . . . . . Streptococcus agalactiae NEM316 .................     1 hits    1 orgs [Streptococcus agalactiae serogroup III]
. . . . . . . . Streptococcus agalactiae H36B ...................     1 hits    1 orgs 
. . . . . . . . Streptococcus agalactiae 515 ....................     1 hits    1 orgs 
. . . . . . . . Streptococcus agalactiae COH1 ...................     1 hits    1 orgs 
. . . . . . . . Streptococcus agalactiae 2603V/R ................     1 hits    1 orgs [Streptococcus agalactiae serogroup V]
. . . . . . . . Streptococcus agalactiae 18RS21 .................     1 hits    1 orgs 
. . . . . . . Streptococcus pyogenes ............................    15 hits   13 orgs 
. . . . . . . . Streptococcus pyogenes MGAS10750 ................     3 hits    1 orgs [Streptococcus pyogenes serotype M4]
. . . . . . . . Streptococcus pyogenes str. Manfredo ............     1 hits    1 orgs [Streptococcus pyogenes serotype M5]
. . . . . . . . Streptococcus pyogenes MGAS10270 ................     1 hits    1 orgs [Streptococcus pyogenes serotype M2]
. . . . . . . . Streptococcus pyogenes serotype M12 .............     2 hits    2 orgs 
. . . . . . . . . Streptococcus pyogenes MGAS9429 ...............     1 hits    1 orgs 
. . . . . . . . . Streptococcus pyogenes MGAS2096 ...............     1 hits    1 orgs 
. . . . . . . . Streptococcus pyogenes MGAS10394 ................     1 hits    1 orgs [Streptococcus pyogenes serotype M6]
. . . . . . . . Streptococcus pyogenes MGAS6180 .................     1 hits    1 orgs [Streptococcus pyogenes serotype M28]
. . . . . . . . Streptococcus pyogenes serotype M3 ..............     2 hits    2 orgs 
. . . . . . . . . Streptococcus pyogenes MGAS315 ................     1 hits    1 orgs 
. . . . . . . . . Streptococcus pyogenes SSI-1 ..................     1 hits    1 orgs 
. . . . . . . . Streptococcus pyogenes serotype M1 ..............     2 hits    2 orgs 
. . . . . . . . . Streptococcus pyogenes M1 GAS .................     1 hits    1 orgs 
. . . . . . . . . Streptococcus pyogenes MGAS5005 ...............     1 hits    1 orgs 
. . . . . . . . Streptococcus pyogenes MGAS8232 .................     1 hits    1 orgs [Streptococcus pyogenes serotype M18]
. . . . . . . . Streptococcus pyogenes M49 591 ..................     1 hits    1 orgs [Streptococcus pyogenes serotype M49]
. . . . . . Lactococcus lactis ..................................     7 hits    3 orgs [Lactococcus]
. . . . . . . Lactococcus lactis subsp. lactis Il1403 ...........     3 hits    1 orgs [Lactococcus lactis subsp. lactis]
. . . . . . . Lactococcus lactis subsp. cremoris ................     4 hits    2 orgs 
. . . . . . . . Lactococcus lactis subsp. cremoris SK11 .........     2 hits    1 orgs 
. . . . . . . . Lactococcus lactis subsp. cremoris MG1363 .......     2 hits    1 orgs 
. . . . . Lactobacillus casei ...................................     8 hits    2 orgs [Lactobacillaceae; Lactobacillus]
. . . . . . Lactobacillus casei BL23 ............................     5 hits    1 orgs 
. . . . . . Lactobacillus casei ATCC 334 ........................     3 hits    1 orgs 
. . . . Listeria monocytogenes ..................................     8 hits    8 orgs [Bacillales; Listeriaceae; Listeria]
. . . . . Listeria monocytogenes FSL J2-064 .....................     1 hits    1 orgs 
. . . . . Listeria monocytogenes FSL J1-175 .....................     1 hits    1 orgs 
. . . . . Listeria monocytogenes FSL R2-503 .....................     1 hits    1 orgs 
. . . . . Listeria monocytogenes FSL J1-194 .....................     1 hits    1 orgs 
. . . . . Listeria monocytogenes HPB2262 ........................     1 hits    1 orgs 
. . . . . Listeria monocytogenes FSL N1-017 .....................     1 hits    1 orgs 
. . . . . Listeria monocytogenes str. 4b F2365 ..................     1 hits    1 orgs 
. . . . . Listeria monocytogenes str. 4b H7858 ..................     1 hits    1 orgs 
. . Bacteroidetes ...............................................   132 hits   13 orgs [Bacteroidetes/Chlorobi group]
. . . Flavobacteriales ..........................................    75 hits    3 orgs [Flavobacteria]
. . . . Flavobacteriaceae .......................................    66 hits    2 orgs 
. . . . . Flavobacterium psychrophilum JIP02/86 .................    56 hits    1 orgs [Flavobacterium; Flavobacterium psychrophilum]
. . . . . Kordia algicida OT-1 ..................................    10 hits    1 orgs [Kordia; Kordia algicida]
. . . . Flavobacteriales bacterium ALC-1 ........................     9 hits    1 orgs [unclassified Flavobacteriales; unclassified Flavobacteriales (miscellaneous)]
. . . Bacteroidales .............................................    57 hits   10 orgs [Bacteroidetes (class)]
. . . . Bacteroides .............................................    38 hits    9 orgs [Bacteroidaceae]
. . . . . Bacteroides fragilis ..................................     5 hits    2 orgs 
. . . . . . Bacteroides fragilis NCTC 9343 ......................     4 hits    1 orgs 
. . . . . . Bacteroides fragilis YCH46 ..........................     1 hits    1 orgs 
. . . . . Bacteroides ovatus ATCC 8483 ..........................    12 hits    1 orgs [Bacteroides ovatus]
. . . . . Bacteroides stercoris ATCC 43183 ......................     6 hits    1 orgs [Bacteroides stercoris]
. . . . . Bacteroides coprocola DSM 17136 .......................     3 hits    1 orgs [Bacteroides coprocola]
. . . . . Bacteroides vulgatus ATCC 8482 ........................     2 hits    1 orgs [Bacteroides vulgatus]
. . . . . Bacteroides thetaiotaomicron VPI-5482 .................     4 hits    1 orgs [Bacteroides thetaiotaomicron]
. . . . . Bacteroides caccae ATCC 43185 .........................     4 hits    1 orgs [Bacteroides caccae]
. . . . . Bacteroides capillosus ATCC 29799 .....................     2 hits    1 orgs [Bacteroides capillosus]
. . . . Alistipes putredinis DSM 17216 ..........................    19 hits    1 orgs [Rikenellaceae; Alistipes; Alistipes putredinis]
. . Chlamydiae/Verrucomicrobia group ............................    28 hits    2 orgs 
. . . bacterium Ellin514 ........................................    16 hits    1 orgs [Verrucomicrobia; Verrucomicrobiae; Verrucomicrobiales; Verrucomicrobia subdivision 3]
. . . Victivallis vadensis ATCC BAA-548 .........................    12 hits    1 orgs [Lentisphaerae; Victivallales; Victivallaceae; Victivallis; Victivallis vadensis]
. . Gammaproteobacteria .........................................    38 hits    3 orgs [Proteobacteria]
. . . Shewanella ................................................    31 hits    2 orgs [Alteromonadales; Shewanellaceae]
. . . . Shewanella pealeana ATCC 700345 .........................    26 hits    1 orgs [Shewanella pealeana]
. . . . Shewanella halifaxensis HAW-EB4 .........................     5 hits    1 orgs [Shewanella halifaxensis]
. . . Photobacterium sp. SKA34 ..................................     7 hits    1 orgs [Vibrionales; Vibrionaceae; Photobacterium]
. . Treponema ...................................................    31 hits    3 orgs [Spirochaetes; Spirochaetes (class); Spirochaetales; Spirochaetaceae]
. . . Treponema denticola ATCC 35405 ............................    25 hits    1 orgs [Treponema denticola]
. . . Treponema pallidum subsp. pallidum ........................     6 hits    2 orgs [Treponema pallidum]
. . . . Treponema pallidum subsp. pallidum str. Nichols .........     3 hits    1 orgs 
. . . . Treponema pallidum subsp. pallidum SS14 .................     3 hits    1 orgs 
. . Synechococcus sp. WH 7805 ...................................    21 hits    1 orgs [Cyanobacteria; Chroococcales; Synechococcus]
. . Actinomyces odontolyticus ATCC 17982 ........................     5 hits    1 orgs [Actinobacteria; Actinobacteria (class); Actinobacteridae; Actinomycetales; Actinomycineae; Actinomycetaceae; Actinomyces; Actinomyces odontolyticus]
. . Mollicutes ..................................................     3 hits    2 orgs [Tenericutes]
. . . Acholeplasma laidlawii PG-8A ..............................     2 hits    1 orgs [Acholeplasmatales; Acholeplasmataceae; Acholeplasma; Acholeplasma laidlawii]
. . . Mycoplasma agalactiae PG2 .................................     1 hits    1 orgs [Mycoplasmatales; Mycoplasmataceae; Mycoplasma; Mycoplasma agalactiae]
. . Fusobacterium nucleatum .....................................     4 hits    2 orgs [Fusobacteria; Fusobacteria (class); Fusobacteriales; Fusobacteriaceae; Fusobacterium]
. . . Fusobacterium nucleatum subsp. nucleatum ATCC 25586 .......     3 hits    1 orgs [Fusobacterium nucleatum subsp. nucleatum]
. . . Fusobacterium nucleatum subsp. vincentii ATCC 49256 .......     1 hits    1 orgs [Fusobacterium nucleatum subsp. vincentii]
. . candidate division TM7 single-cell isolate TM7b .............     1 hits    1 orgs [unclassified Bacteria; candidate division TM7]
```
