# Supplementary material for: Trichomonas vaginalis vast BspA-like gene family: evidence for functional diversity from structural organisation and transcriptomics
Source: BMC Genomics. 2010 Feb 8;11:99. doi: 10.1186/1471-2164-11-99 (PMC2843621; doi:10.1186/1471-2164-11-99)
Supplement: Additional file 3 — Supplemental Table S3. PHI-/PSI-Blast taxonomic report for proteins with TpLRR from Trichomonas vaginalis and Entamoeba histolytica. Full taxonomic report of PHI/PSI-BlastP searches on NCBI RefSeq protein database. In html format to be open in a web browser. [file 1471-2164-11-99-S3.HTML]

### Table S3. Taxomic report for PSI-BLAST search on Trichomonas and Entamoeba at RefSeq

---


### Profile (PSSM): based on PHI-Blast (see Table 1 and Table S2), two iterations

### e-value <= 0.00001

---


### 1) Trichomonas

**Index**

- Lineage Report
- Organism Report
- Taxonomy Report
- Help

### 2) Entamoeba

**Index**

- Lineage Report
- Organism Report
- Taxonomy Report
- Help

---


### 1) Trichomonas

**Lineage Report**  

```
Trichomonas vaginalis G3 [trichomonads]
. Trichomonas vaginalis G3 -  430 5419 hits [trichomonads]  surface antigen BspA-like [Trichomonas vaginalis G3]
```

---

**Organism Report**

```
  Trichomonas vaginalis G3 [trichomonads] taxid 412133
 ref|XP_001313891.1| surface antigen BspA-like [Trichomonas...     430  1e-120
 ref|XP_001321233.1| surface antigen BspA-like [Trichomonas...     425  3e-119
 ref|XP_001310018.1| surface antigen BspA-like [Trichomonas...     398  3e-111
 ref|XP_001327783.1| surface antigen BspA-like [Trichomonas...     375  5e-104
 ref|XP_001584042.1| surface antigen BspA-like [Trichomonas...     370  1e-102
 ref|XP_001295773.1| surface antigen BspA-like [Trichomonas...     363  2e-100
 ref|XP_001303246.1| surface antigen BspA-like [Trichomonas...     342  2e-94
 ref|XP_001313156.1| surface antigen BspA-like [Trichomonas...     334  6e-92
 ref|XP_001323112.1| surface antigen BspA-like [Trichomonas...     328  4e-90
 ref|XP_001319549.1| surface antigen BspA-like [Trichomonas...     325  3e-89
 ref|XP_001313272.1| surface antigen BspA-like [Trichomonas...     320  2e-87
 ref|XP_001582492.1| surface antigen BspA-like [Trichomonas...     320  2e-87
 ref|XP_001320775.1| surface antigen BspA-like [Trichomonas...     319  2e-87
 ref|XP_001583668.1| surface antigen BspA-like [Trichomonas...     318  5e-87
 ref|XP_001317101.1| surface antigen BspA-like [Trichomonas...     316  3e-86
 ref|XP_001294553.1| surface antigen BspA-like [Trichomonas...     315  6e-86
 ref|XP_001300913.1| surface antigen BspA-like [Trichomonas...     314  8e-86
 ref|XP_001315000.1| surface antigen BspA-like [Trichomonas...     311  9e-85
 ref|XP_001300837.1| surface antigen BspA-like [Trichomonas...     310  2e-84
 ref|XP_001580185.1| surface antigen BspA-like [Trichomonas...     309  2e-84
 ref|XP_001328562.1| surface antigen BspA-like [Trichomonas...     309  3e-84
 ref|XP_001583566.1| surface antigen BspA-like [Trichomonas...     308  4e-84
 ref|XP_001580208.1| surface antigen BspA-like [Trichomonas...     308  4e-84
 ref|XP_001327997.1| surface antigen BspA-like [Trichomonas...     305  3e-83
 ref|XP_001318269.1| surface antigen BspA-like [Trichomonas...     305  5e-83
 ref|XP_001320680.1| surface antigen BspA-like [Trichomonas...     304  5e-83
 ref|XP_001326610.1| surface antigen BspA-like [Trichomonas...     304  9e-83
 ref|XP_001583570.1| surface antigen BspA-like [Trichomonas...     301  5e-82
 ref|XP_001316522.1| surface antigen BspA-like [Trichomonas...     300  1e-81
 ref|XP_001325637.1| surface antigen BspA-like [Trichomonas...     299  2e-81
 ref|XP_001584517.1| surface antigen BspA-like [Trichomonas...     298  4e-81
 ref|XP_001583571.1| surface antigen BspA-like [Trichomonas...     298  7e-81
 ref|XP_001322944.1| surface antigen BspA-like [Trichomonas...     296  1e-80
 ref|XP_001310359.1| surface antigen BspA-like [Trichomonas...     295  5e-80
 ref|XP_001582486.1| surface antigen BspA-like [Trichomonas...     294  6e-80
 ref|XP_001326467.1| surface antigen BspA-like [Trichomonas...     294  8e-80
 ref|XP_001311382.1| surface antigen BspA-like [Trichomonas...     294  1e-79
 ref|XP_001327776.1| surface antigen BspA-like [Trichomonas...     294  1e-79
 ref|XP_001311470.1| surface antigen BspA-like [Trichomonas...     293  1e-79
 ref|XP_001583564.1| cell surface protein, putative [Tricho...     293  1e-79
 ref|XP_001305369.1| surface antigen BspA-like [Trichomonas...     293  2e-79
 ref|XP_001313426.1| surface antigen BspA-like [Trichomonas...     291  6e-79
 ref|XP_001325828.1| surface antigen BspA-like [Trichomonas...     290  2e-78
 ref|XP_001321334.1| surface antigen BspA-like [Trichomonas...     289  3e-78
 ref|XP_001316797.1| surface antigen BspA-like [Trichomonas...     289  4e-78
 ref|XP_001583568.1| surface antigen BspA-like [Trichomonas...     288  4e-78
 ref|XP_001584519.1| surface antigen BspA-like [Trichomonas...     288  5e-78
 ref|XP_001318271.1| surface antigen BspA-like [Trichomonas...     288  7e-78
 ref|XP_001578993.1| surface antigen BspA-like [Trichomonas...     287  1e-77
 ref|XP_001315147.1| surface antigen BspA-like [Trichomonas...     286  2e-77
 ref|XP_001296463.1| surface antigen BspA-like [Trichomonas...     286  2e-77
 ref|XP_001308356.1| surface antigen BspA-like [Trichomonas...     284  7e-77
 ref|XP_001300375.1| surface antigen BspA-like [Trichomonas...     284  7e-77
 ref|XP_001579760.1| surface antigen BspA-like [Trichomonas...     282  4e-76
 ref|XP_001311580.1| cell surface protein, putative [Tricho...     281  8e-76
 ref|XP_001313418.1| surface antigen BspA-like [Trichomonas...     281  1e-75
 ref|XP_001325465.1| Leucine Rich Repeat family protein [Tr...     280  1e-75
 ref|XP_001580031.1| surface antigen BspA-like [Trichomonas...     279  3e-75
 ref|XP_001326204.1| surface antigen BspA-like [Trichomonas...     279  3e-75
 ref|XP_001316682.1| cell surface protein, putative [Tricho...     278  7e-75
 ref|XP_001316735.1| surface antigen BspA-like [Trichomonas...     276  2e-74
 ref|XP_001324655.1| surface antigen BspA-like [Trichomonas...     276  2e-74
 ref|XP_001314845.1| surface antigen BspA-like [Trichomonas...     275  4e-74
 ref|XP_001303399.1| surface antigen BspA-like [Trichomonas...     274  6e-74
 ref|XP_001313121.1| surface antigen BspA-like [Trichomonas...     274  1e-73
 ref|XP_001581243.1| surface antigen BspA-like [Trichomonas...     273  1e-73
 ref|XP_001327834.1| Leucine Rich Repeat family protein [Tr...     272  5e-73
 ref|XP_001578939.1| surface antigen BspA-like [Trichomonas...     272  5e-73
 ref|XP_001328347.1| surface antigen BspA-like [Trichomonas...     271  7e-73
 ref|XP_001312386.1| Leucine Rich Repeat family protein [Tr...     271  1e-72
 ref|XP_001316680.1| surface antigen BspA-like [Trichomonas...     269  2e-72
 ref|XP_001580641.1| cell surface protein, putative [Tricho...     268  4e-72
 ref|XP_001305199.1| surface antigen BspA-like [Trichomonas...     268  6e-72
 ref|XP_001583764.1| surface antigen BspA-like [Trichomonas...     268  7e-72
 ref|XP_001309885.1| surface antigen BspA-like [Trichomonas...     268  8e-72
 ref|XP_001327832.1| surface antigen BspA-like [Trichomonas...     268  8e-72
 ref|XP_001317132.1| surface antigen BspA-like [Trichomonas...     268  8e-72
 ref|XP_001320160.1| surface antigen BspA-like [Trichomonas...     268  8e-72
 ref|XP_001312385.1| surface antigen BspA-like [Trichomonas...     267  1e-71
 ref|XP_001307916.1| surface antigen BspA-like [Trichomonas...     267  1e-71
 ref|XP_001320690.1| surface antigen BspA-like [Trichomonas...     266  2e-71
 ref|XP_001301673.1| cell surface protein, putative [Tricho...     266  3e-71
 ref|XP_001325301.1| cell surface protein, putative [Tricho...     266  3e-71
 ref|XP_001327818.1| surface antigen BspA-like [Trichomonas...     265  4e-71
 ref|XP_001317386.1| hypothetical protein TVAG_058200 [Tric...     265  4e-71
 ref|XP_001579739.1| surface antigen BspA-like [Trichomonas...     264  6e-71
 ref|XP_001307744.1| surface antigen BspA-like [Trichomonas...     264  8e-71
 ref|XP_001277016.1| Leucine Rich Repeat family protein [Tr...     264  8e-71
 ref|XP_001580983.1| surface antigen BspA-like [Trichomonas...     264  9e-71
 ref|XP_001313390.1| surface antigen Bsp, putative [Trichom...     263  1e-70
 ref|XP_001322517.1| Leucine Rich Repeat family protein [Tr...     263  2e-70
 ref|XP_001318845.1| surface antigen BspA-like [Trichomonas...     263  3e-70
 ref|XP_001302174.1| surface antigen BspA-like [Trichomonas...     262  3e-70
 ref|XP_001319438.1| surface antigen BspA-like [Trichomonas...     262  3e-70
 ref|XP_001309886.1| cell surface protein, putative [Tricho...     262  4e-70
 ref|XP_001308549.1| surface antigen BspA-like [Trichomonas...     262  4e-70
 ref|XP_001301434.1| surface antigen BspA-like [Trichomonas...     262  4e-70
 ref|XP_001307417.1| surface antigen BspA-like [Trichomonas...     261  6e-70
 ref|XP_001330510.1| surface antigen BspA-like [Trichomonas...     261  6e-70
 ref|XP_001317928.1| surface antigen BspA-like [Trichomonas...     261  7e-70
 ref|XP_001330691.1| surface antigen BspA-like [Trichomonas...     261  7e-70
 ref|XP_001320608.1| surface antigen BspA-like [Trichomonas...     261  1e-69
 ref|XP_001580634.1| cell surface protein, putative [Tricho...     260  1e-69
 ref|XP_001580874.1| surface antigen BspA-like [Trichomonas...     260  1e-69
 ref|XP_001309881.1| surface antigen BspA-like [Trichomonas...     260  2e-69
 ref|XP_001276952.1| Leucine Rich Repeat family protein [Tr...     260  2e-69
 ref|XP_001321471.1| surface antigen BspA-like [Trichomonas...     259  2e-69
 ref|XP_001307861.1| surface antigen BspA-like [Trichomonas...     259  2e-69
 ref|XP_001322525.1| Leucine Rich Repeat family protein [Tr...     259  3e-69
 ref|XP_001303561.1| surface antigen BspA-like [Trichomonas...     258  4e-69
 ref|XP_001306181.1| surface antigen BspA-like [Trichomonas...     258  5e-69
 ref|XP_001584521.1| surface antigen BspA-like [Trichomonas...     258  6e-69
 ref|XP_001326140.1| surface antigen BspA-like [Trichomonas...     258  7e-69
 ref|XP_001584306.1| surface antigen BspA-like [Trichomonas...     257  1e-68
 ref|XP_001307862.1| surface antigen BspA-like [Trichomonas...     257  1e-68
 ref|XP_001312134.1| surface antigen BspA-like [Trichomonas...     257  1e-68
 ref|XP_001328231.1| surface antigen BspA-like [Trichomonas...     257  1e-68
 ref|XP_001319612.1| Leucine Rich Repeat family protein [Tr...     257  1e-68
 ref|XP_001584314.1| surface antigen BspA-like [Trichomonas...     257  1e-68
 ref|XP_001315701.1| surface antigen BspA-like [Trichomonas...     257  2e-68
 ref|XP_001584518.1| surface antigen BspA-like [Trichomonas...     256  2e-68
 ref|XP_001305729.1| surface antigen BspA-like [Trichomonas...     256  2e-68
 ref|XP_001315459.1| surface antigen BspA-like [Trichomonas...     256  2e-68
 ref|XP_001277015.1| Leucine Rich Repeat family protein [Tr...     256  3e-68
 ref|XP_001580139.1| surface antigen BspA-like [Trichomonas...     256  3e-68
 ref|XP_001326822.1| surface antigen BspA-like [Trichomonas...     256  3e-68
 ref|XP_001323277.1| surface antigen BspA-like [Trichomonas...     255  4e-68
 ref|XP_001307420.1| surface antigen BspA-like [Trichomonas...     255  4e-68
 ref|XP_001326586.1| surface antigen BspA-like [Trichomonas...     255  4e-68
 ref|XP_001315461.1| surface antigen BspA-like [Trichomonas...     255  5e-68
 ref|XP_001300468.1| Leucine Rich Repeat family protein [Tr...     255  5e-68
 ref|XP_001313404.1| surface antigen BspA-like [Trichomonas...     255  5e-68
 ref|XP_001314765.1| surface antigen BspA-like [Trichomonas...     254  6e-68
 ref|XP_001584520.1| surface antigen BspA-like [Trichomonas...     254  6e-68
 ref|XP_001311472.1| surface antigen BspA-like [Trichomonas...     254  8e-68
 ref|XP_001329755.1| Leucine Rich Repeat family protein [Tr...     254  9e-68
 ref|XP_001580174.1| surface antigen BspA-like [Trichomonas...     254  1e-67
 ref|XP_001583817.1| surface antigen BspA-like [Trichomonas...     252  3e-67
 ref|XP_001316471.1| surface antigen BspA-like [Trichomonas...     252  3e-67
 ref|XP_001309884.1| surface antigen Bsp, putative [Trichom...     252  4e-67
 ref|XP_001310586.1| surface antigen BspA-like [Trichomonas...     251  5e-67
 ref|XP_001276937.1| surface antigen BspA-like [Trichomonas...     251  7e-67
 ref|XP_001324656.1| surface antigen BspA-like [Trichomonas...     251  7e-67
 ref|XP_001309390.1| surface antigen BspA-like [Trichomonas...     251  1e-66
 ref|XP_001321229.1| surface antigen BspA-like [Trichomonas...     251  1e-66
 ref|XP_001301145.1| surface antigen BspA-like [Trichomonas...     250  1e-66
 ref|XP_001318168.1| surface antigen BspA-like [Trichomonas...     250  1e-66
 ref|XP_001314105.1| surface antigen BspA-like [Trichomonas...     250  2e-66
 ref|XP_001323983.1| surface antigen BspA-like [Trichomonas...     249  2e-66
 ref|XP_001580341.1| surface antigen BspA-like [Trichomonas...     249  2e-66
 ref|XP_001297020.1| surface antigen BspA-like [Trichomonas...     249  3e-66
 ref|XP_001306128.1| surface antigen BspA-like [Trichomonas...     248  5e-66
 ref|XP_001318284.1| surface antigen BspA-like [Trichomonas...     248  5e-66
 ref|XP_001315236.1| surface antigen BspA-like [Trichomonas...     248  6e-66
 ref|XP_001582172.1| surface antigen BspA-like [Trichomonas...     247  8e-66
 ref|XP_001316485.1| surface antigen BspA-like [Trichomonas...     247  9e-66
 ref|XP_001276979.1| Leucine Rich Repeat family protein [Tr...     247  1e-65
 ref|XP_001328163.1| surface antigen BspA-like [Trichomonas...     247  2e-65
 ref|XP_001310243.1| surface antigen BspA-like [Trichomonas...     247  2e-65
 ref|XP_001317424.1| surface antigen BspA-like [Trichomonas...     246  2e-65
 ref|XP_001314904.1| Leucine Rich Repeat family protein [Tr...     246  2e-65
 ref|XP_001307859.1| Leucine Rich Repeat family protein [Tr...     246  3e-65
 ref|XP_001325166.1| surface antigen BspA-like [Trichomonas...     246  3e-65
 ref|XP_001311308.1| surface antigen BspA-like [Trichomonas...     246  3e-65
 ref|XP_001584310.1| surface antigen BspA-like [Trichomonas...     246  3e-65
 ref|XP_001318754.1| Leucine Rich Repeat family protein [Tr...     245  4e-65
 ref|XP_001579236.1| Leucine Rich Repeat family protein [Tr...     245  4e-65
 ref|XP_001313165.1| surface antigen BspA-like [Trichomonas...     245  4e-65
 ref|XP_001315859.1| surface antigen BspA-like [Trichomonas...     245  5e-65
 ref|XP_001302758.1| surface antigen BspA-like [Trichomonas...     244  1e-64
 ref|XP_001316472.1| surface antigen BspA-like [Trichomonas...     244  1e-64
 ref|XP_001581880.1| surface antigen BspA-like [Trichomonas...     243  1e-64
 ref|XP_001330142.1| surface antigen BspA-like [Trichomonas...     243  1e-64
 ref|XP_001583614.1| surface antigen BspA-like [Trichomonas...     243  2e-64
 ref|XP_001307730.1| surface antigen BspA-like [Trichomonas...     243  2e-64
 ref|XP_001321125.1| surface antigen BspA-like [Trichomonas...     242  3e-64
 ref|XP_001329219.1| surface antigen BspA-like [Trichomonas...     242  3e-64
 ref|XP_001583613.1| surface antigen BspA-like [Trichomonas...     242  3e-64
 ref|XP_001320123.1| surface antigen BspA-like [Trichomonas...     242  4e-64
 ref|XP_001321309.1| surface antigen BspA-like [Trichomonas...     241  7e-64
 ref|XP_001330077.1| surface antigen BspA-like [Trichomonas...     241  8e-64
 ref|XP_001297552.1| surface antigen BspA-like [Trichomonas...     241  9e-64
 ref|XP_001319437.1| surface antigen BspA-like [Trichomonas...     241  1e-63
 ref|XP_001306350.1| surface antigen BspA-like [Trichomonas...     240  1e-63
 ref|XP_001302598.1| surface antigen BspA-like [Trichomonas...     240  1e-63
 ref|XP_001312006.1| surface antigen BspA-like [Trichomonas...     240  1e-63
 ref|XP_001307708.1| surface antigen BspA-like [Trichomonas...     240  1e-63
 ref|XP_001581729.1| surface antigen BspA-like [Trichomonas...     240  1e-63
 ref|XP_001307421.1| hypothetical protein TVAG_241760 [Tric...     240  2e-63
 ref|XP_001309820.1| surface antigen BspA-like [Trichomonas...     240  2e-63
 ref|XP_001314499.1| surface antigen BspA-like [Trichomonas...     240  2e-63
 ref|XP_001321228.1| surface antigen BspA-like [Trichomonas...     239  3e-63
 ref|XP_001276867.1| surface antigen BspA-like [Trichomonas...     239  3e-63
 ref|XP_001584044.1| surface antigen BspA-like [Trichomonas...     239  4e-63
 ref|XP_001321963.1| surface antigen Bsp, putative [Trichom...     239  4e-63
 ref|XP_001329658.1| surface antigen BspA-like [Trichomonas...     238  5e-63
 ref|XP_001316127.1| surface antigen BspA-like [Trichomonas...     238  6e-63
 ref|XP_001323192.1| surface antigen BspA-like [Trichomonas...     238  6e-63
 ref|XP_001308904.1| surface antigen BspA-like [Trichomonas...     237  8e-63
 ref|XP_001309234.1| surface antigen BspA-like [Trichomonas...     237  8e-63
 ref|XP_001315578.1| surface antigen BspA-like [Trichomonas...     237  1e-62
 ref|XP_001308031.1| surface antigen BspA-like [Trichomonas...     237  2e-62
 ref|XP_001329030.1| Leucine Rich Repeat family protein [Tr...     236  2e-62
 ref|XP_001316457.1| surface antigen BspA-like [Trichomonas...     236  2e-62
 ref|XP_001581326.1| surface antigen BspA-like [Trichomonas...     236  3e-62
 ref|XP_001315653.1| Leucine Rich Repeat family protein [Tr...     236  3e-62
 ref|XP_001584309.1| surface antigen BspA-like [Trichomonas...     236  3e-62
 ref|XP_001314401.1| surface antigen BspA-like [Trichomonas...     236  3e-62
 ref|XP_001310600.1| surface antigen BspA-like [Trichomonas...     236  4e-62
 ref|XP_001306394.1| surface antigen BspA-like [Trichomonas...     236  4e-62
 ref|XP_001304980.1| surface antigen BspA-like [Trichomonas...     235  4e-62
 ref|XP_001315820.1| surface antigen BspA-like [Trichomonas...     235  4e-62
 ref|XP_001298988.1| surface antigen BspA-like [Trichomonas...     235  5e-62
 ref|XP_001327995.1| surface antigen BspA-like [Trichomonas...     235  5e-62
 ref|XP_001298289.1| surface antigen BspA-like [Trichomonas...     235  6e-62
 ref|XP_001317274.1| surface antigen BspA-like [Trichomonas...     235  6e-62
 ref|XP_001310344.1| surface antigen BspA-like [Trichomonas...     235  6e-62
 ref|XP_001578977.1| surface antigen BspA-like [Trichomonas...     235  6e-62
 ref|XP_001329761.1| surface antigen BspA-like [Trichomonas...     234  8e-62
 ref|XP_001305581.1| surface antigen Bsp, putative [Trichom...     234  8e-62
 ref|XP_001582116.1| surface antigen BspA-like [Trichomonas...     234  8e-62
 ref|XP_001316739.1| surface antigen BspA-like [Trichomonas...     234  8e-62
 ref|XP_001313953.1| surface antigen BspA-like [Trichomonas...     234  1e-61
 ref|XP_001582619.1| surface antigen BspA-like [Trichomonas...     234  1e-61
 ref|XP_001307704.1| surface antigen BspA-like [Trichomonas...     234  1e-61
 ref|XP_001321571.1| surface antigen BspA-like [Trichomonas...     234  1e-61
 ref|XP_001316473.1| surface antigen BspA-like [Trichomonas...     234  1e-61
 ref|XP_001315460.1| surface antigen BspA-like [Trichomonas...     234  1e-61
 ref|XP_001308641.1| surface antigen BspA-like [Trichomonas...     233  2e-61
 ref|XP_001309839.1| surface antigen BspA-like [Trichomonas...     233  2e-61
 ref|XP_001312375.1| surface antigen BspA-like [Trichomonas...     233  2e-61
 ref|XP_001315559.1| surface antigen BspA-like [Trichomonas...     233  2e-61
 ref|XP_001308805.1| surface antigen BspA-like [Trichomonas...     233  2e-61
 ref|XP_001320607.1| surface antigen BspA-like [Trichomonas...     233  2e-61
 ref|XP_001296128.1| surface antigen BspA-like [Trichomonas...     232  3e-61
 ref|XP_001305184.1| Leucine Rich Repeat family protein [Tr...     232  3e-61
 ref|XP_001307497.1| surface antigen BspA-like [Trichomonas...     232  4e-61
 ref|XP_001330231.1| surface antigen BspA-like [Trichomonas...     231  5e-61
 ref|XP_001324795.1| surface antigen BspA-like [Trichomonas...     231  6e-61
 ref|XP_001306978.1| surface antigen BspA-like [Trichomonas...     231  6e-61
 ref|XP_001302536.1| surface antigen BspA-like [Trichomonas...     231  1e-60
 ref|XP_001296869.1| surface antigen BspA-like [Trichomonas...     231  1e-60
 ref|XP_001579825.1| surface antigen BspA-like [Trichomonas...     230  2e-60
 ref|XP_001316557.1| surface antigen BspA-like [Trichomonas...     230  2e-60
 ref|XP_001329823.1| surface antigen BspA-like [Trichomonas...     229  3e-60
 ref|XP_001329988.1| surface antigen BspA-like [Trichomonas...     229  3e-60
 ref|XP_001579564.1| surface antigen BspA-like [Trichomonas...     229  3e-60
 ref|XP_001584312.1| surface antigen BspA-like [Trichomonas...     229  4e-60
 ref|XP_001306817.1| surface antigen BspA-like [Trichomonas...     228  5e-60
 ref|XP_001326170.1| surface antigen BspA-like [Trichomonas...     228  5e-60
 ref|XP_001322326.1| surface antigen BspA-like [Trichomonas...     228  5e-60
 ref|XP_001305579.1| conserved hypothetical protein [Tricho...     228  5e-60
 ref|XP_001322365.1| surface antigen Bsp, putative [Trichom...     228  6e-60
 ref|XP_001315490.1| surface antigen BspA-like [Trichomonas...     228  6e-60
 ref|XP_001309112.1| surface antigen BspA-like [Trichomonas...     228  8e-60
 ref|XP_001312800.1| surface antigen BspA-like [Trichomonas...     228  8e-60
 ref|XP_001309842.1| surface antigen BspA-like [Trichomonas...     226  2e-59
 ref|XP_001318748.1| surface antigen BspA-like [Trichomonas...     226  2e-59
 ref|XP_001322413.1| surface antigen BspA-like [Trichomonas...     226  3e-59
 ref|XP_001319243.1| surface antigen BspA-like [Trichomonas...     226  3e-59
 ref|XP_001326468.1| surface antigen BspA-like [Trichomonas...     226  3e-59
 ref|XP_001304480.1| surface antigen BspA-like [Trichomonas...     226  3e-59
 ref|XP_001583025.1| surface antigen BspA-like [Trichomonas...     226  3e-59
 ref|XP_001327833.1| surface antigen BspA-like [Trichomonas...     225  4e-59
 ref|XP_001582153.1| surface antigen BspA-like [Trichomonas...     225  5e-59
 ref|XP_001308799.1| surface antigen BspA-like [Trichomonas...     225  5e-59
 ref|XP_001584303.1| surface antigen BspA-like [Trichomonas...     224  1e-58
 ref|XP_001315235.1| surface antigen BspA-like [Trichomonas...     224  1e-58
 ref|XP_001305131.1| surface antigen BspA-like [Trichomonas...     224  1e-58
 ref|XP_001312690.1| surface antigen BspA-like [Trichomonas...     224  1e-58
 ref|XP_001328157.1| surface antigen BspA-like [Trichomonas...     223  2e-58
 ref|XP_001319647.1| surface antigen BspA-like [Trichomonas...     222  3e-58
 ref|XP_001325362.1| surface antigen BspA-like [Trichomonas...     222  3e-58
 ref|XP_001302667.1| surface antigen BspA-like [Trichomonas...     222  5e-58
 ref|XP_001583256.1| surface antigen BspA-like [Trichomonas...     221  6e-58
 ref|XP_001311508.1| surface antigen BspA-like [Trichomonas...     221  1e-57
 ref|XP_001316474.1| surface antigen BspA-like [Trichomonas...     220  1e-57
 ref|XP_001307858.1| surface antigen BspA-like [Trichomonas...     219  2e-57
 ref|XP_001316694.1| surface antigen BspA-like [Trichomonas...     219  3e-57
 ref|XP_001295737.1| surface antigen BspA-like [Trichomonas...     219  3e-57
 ref|XP_001297026.1| surface antigen BspA-like [Trichomonas...     219  3e-57
 ref|XP_001322084.1| surface antigen BspA-like [Trichomonas...     219  4e-57
 ref|XP_001329850.1| surface antigen BspA-like [Trichomonas...     219  4e-57
 ref|XP_001323401.1| Leucine Rich Repeat family protein [Tr...     218  6e-57
 ref|XP_001325770.1| surface antigen BspA-like [Trichomonas...     218  6e-57
 ref|XP_001324897.1| Leucine Rich Repeat family protein [Tr...     217  9e-57
 ref|XP_001310525.1| Leucine Rich Repeat family protein [Tr...     217  1e-56
 ref|XP_001300663.1| surface antigen BspA-like [Trichomonas...     217  1e-56
 ref|XP_001326021.1| surface antigen BspA-like [Trichomonas...     217  2e-56
 ref|XP_001304295.1| surface antigen BspA-like [Trichomonas...     217  2e-56
 ref|XP_001328723.1| surface antigen BspA-like [Trichomonas...     216  2e-56
 ref|XP_001325446.1| surface antigen BspA-like [Trichomonas...     216  3e-56
 ref|XP_001581093.1| surface antigen BspA-like [Trichomonas...     216  4e-56
 ref|XP_001310818.1| surface antigen BspA-like [Trichomonas...     215  5e-56
 ref|XP_001308141.1| surface antigen BspA-like [Trichomonas...     214  7e-56
 ref|XP_001311832.1| surface antigen BspA-like [Trichomonas...     214  8e-56
 ref|XP_001323432.1| surface antigen BspA-like [Trichomonas...     214  8e-56
 ref|XP_001583892.1| surface antigen BspA-like [Trichomonas...     214  9e-56
 ref|XP_001583195.1| surface antigen BspA-like [Trichomonas...     214  1e-55
 ref|XP_001322411.1| surface antigen BspA-like [Trichomonas...     214  1e-55
 ref|XP_001313013.1| surface antigen BspA-like [Trichomonas...     214  1e-55
 ref|XP_001314807.1| surface antigen BspA-like [Trichomonas...     212  3e-55
 ref|XP_001302511.1| surface antigen BspA-like [Trichomonas...     212  3e-55
 ref|XP_001579116.1| surface antigen BspA-like [Trichomonas...     212  3e-55
 ref|XP_001315863.1| surface antigen BspA-like [Trichomonas...     212  4e-55
 ref|XP_001320904.1| surface antigen BspA-like [Trichomonas...     212  6e-55
 ref|XP_001308716.1| hypothetical protein TVAG_353980 [Tric...     211  7e-55
 ref|XP_001298020.1| surface antigen BspA-like [Trichomonas...     211  8e-55
 ref|XP_001330279.1| Leucine Rich Repeat family protein [Tr...     211  1e-54
 ref|XP_001309450.1| surface antigen BspA-like [Trichomonas...     210  1e-54
 ref|XP_001328674.1| surface antigen BspA-like [Trichomonas...     210  1e-54
 ref|XP_001328939.1| surface antigen BspA-like [Trichomonas...     210  2e-54
 ref|XP_001319517.1| surface antigen BspA-like [Trichomonas...     210  2e-54
 ref|XP_001322761.1| surface antigen BspA-like [Trichomonas...     210  2e-54
 ref|XP_001311620.1| surface antigen BspA-like [Trichomonas...     210  2e-54
 ref|XP_001329821.1| surface antigen BspA-like [Trichomonas...     209  3e-54
 ref|XP_001308539.1| Leucine Rich Repeat family protein [Tr...     209  3e-54
 ref|XP_001321572.1| surface antigen BspA-like [Trichomonas...     209  4e-54
 ref|XP_001318842.1| surface antigen BspA-like [Trichomonas...     209  4e-54
 ref|XP_001315016.1| surface antigen BspA-like [Trichomonas...     209  4e-54
 ref|XP_001327190.1| surface antigen BspA-like [Trichomonas...     209  5e-54
 ref|XP_001310694.1| surface antigen BspA-like [Trichomonas...     209  5e-54
 ref|XP_001317155.1| surface antigen BspA-like [Trichomonas...     208  5e-54
 ref|XP_001307334.1| surface antigen BspA-like [Trichomonas...     208  7e-54
 ref|XP_001328763.1| surface antigen BspA-like [Trichomonas...     208  7e-54
 ref|XP_001320663.1| surface antigen BspA-like [Trichomonas...     208  7e-54
 ref|XP_001315201.1| cell surface protein, putative [Tricho...     207  9e-54
 ref|XP_001582516.1| hypothetical protein TVAG_012950 [Tric...     207  1e-53
 ref|XP_001304390.1| surface antigen BspA-like [Trichomonas...     207  1e-53
 ref|XP_001318044.1| surface antigen BspA-like [Trichomonas...     207  1e-53
 ref|XP_001316263.1| surface antigen BspA-like [Trichomonas...     207  1e-53
 ref|XP_001306351.1| surface antigen BspA-like [Trichomonas...     207  2e-53
 ref|XP_001306722.1| surface antigen BspA-like [Trichomonas...     207  2e-53
 ref|XP_001303554.1| surface antigen BspA-like [Trichomonas...     206  2e-53
 ref|XP_001311011.1| surface antigen Bsp, putative [Trichom...     206  2e-53
 ref|XP_001321192.1| surface antigen BspA-like [Trichomonas...     206  3e-53
 ref|XP_001300591.1| surface antigen BspA-like [Trichomonas...     206  3e-53
 ref|XP_001584308.1| surface antigen BspA-like [Trichomonas...     206  3e-53
 ref|XP_001579181.1| surface antigen BspA-like [Trichomonas...     205  3e-53
 ref|XP_001321287.1| surface antigen BspA-like [Trichomonas...     204  1e-52
 ref|XP_001310695.1| Leucine Rich Repeat family protein [Tr...     204  1e-52
 ref|XP_001301143.1| surface antigen BspA-like [Trichomonas...     204  1e-52
 ref|XP_001583652.1| surface antigen BspA-like [Trichomonas...     203  2e-52
 ref|XP_001315237.1| surface antigen BspA-like [Trichomonas...     202  3e-52
 ref|XP_001316253.1| surface antigen Bsp, putative [Trichom...     202  3e-52
 ref|XP_001312325.1| hypothetical protein TVAG_043030 [Tric...     202  3e-52
 ref|XP_001301406.1| surface antigen BspA-like [Trichomonas...     202  3e-52
 ref|XP_001311579.1| surface antigen BspA-like [Trichomonas...     202  3e-52
 ref|XP_001581225.1| surface antigen BspA-like [Trichomonas...     202  4e-52
 ref|XP_001311984.1| surface antigen BspA-like [Trichomonas...     202  5e-52
 ref|XP_001317245.1| surface antigen BspA-like [Trichomonas...     202  5e-52
 ref|XP_001309051.1| surface antigen BspA-like [Trichomonas...     202  5e-52
 ref|XP_001302029.1| surface antigen BspA-like [Trichomonas...     202  6e-52
 ref|XP_001311694.1| surface antigen BspA-like [Trichomonas...     201  8e-52
 ref|XP_001579044.1| Leucine Rich Repeat family protein [Tr...     201  1e-51
 ref|XP_001578976.1| conserved hypothetical protein [Tricho...     201  1e-51
 ref|XP_001582895.1| surface antigen BspA-like [Trichomonas...     201  1e-51
 ref|XP_001323870.1| surface antigen BspA-like [Trichomonas...     200  1e-51
 ref|XP_001322369.1| Leucine Rich Repeat family protein [Tr...     200  2e-51
 ref|XP_001315014.1| surface antigen BspA-like [Trichomonas...     200  2e-51
 ref|XP_001320323.1| Leucine Rich Repeat family protein [Tr...     200  2e-51
 ref|XP_001321875.1| surface antigen BspA-like [Trichomonas...     200  2e-51
 ref|XP_001306833.1| surface antigen BspA-like [Trichomonas...     199  3e-51
 ref|XP_001580137.1| surface antigen BspA-like [Trichomonas...     199  3e-51
 ref|XP_001303552.1| surface antigen BspA-like [Trichomonas...     199  4e-51
 ref|XP_001307332.1| cell surface protein, putative [Tricho...     199  4e-51
 ref|XP_001323847.1| surface antigen BspA-like [Trichomonas...     198  6e-51
 ref|XP_001311047.1| surface antigen BspA-like [Trichomonas...     198  7e-51
 ref|XP_001328767.1| surface antigen BspA-like [Trichomonas...     198  8e-51
 ref|XP_001309486.1| surface antigen BspA-like [Trichomonas...     198  8e-51
 ref|XP_001317521.1| surface antigen BspA-like [Trichomonas...     197  1e-50
 ref|XP_001322549.1| surface antigen BspA-like [Trichomonas...     197  1e-50
 ref|XP_001583079.1| surface antigen BspA-like [Trichomonas...     197  1e-50
 ref|XP_001306837.1| surface antigen BspA-like [Trichomonas...     197  1e-50
 ref|XP_001322537.1| Leucine Rich Repeat family protein [Tr...     197  1e-50
 ref|XP_001311621.1| hypothetical protein TVAG_333550 [Tric...     197  2e-50
 ref|XP_001584304.1| surface antigen BspA-like [Trichomonas...     197  2e-50
 ref|XP_001582154.1| surface antigen BspA-like [Trichomonas...     197  2e-50
 ref|XP_001319162.1| surface antigen BspA-like [Trichomonas...     197  2e-50
 ref|XP_001306942.1| surface antigen BspA-like [Trichomonas...     197  2e-50
 ref|XP_001584208.1| leucine Rich Repeat domain protein, pu...     196  3e-50
 ref|XP_001303242.1| surface antigen BspA-like [Trichomonas...     195  4e-50
 ref|XP_001303105.1| surface antigen BspA-like [Trichomonas...     195  4e-50
 ref|XP_001297500.1| surface antigen BspA-like [Trichomonas...     195  4e-50
 ref|XP_001328349.1| surface antigen BspA-like [Trichomonas...     195  4e-50
 ref|XP_001310683.1| Leucine Rich Repeat family protein [Tr...     195  4e-50
 ref|XP_001329959.1| surface antigen BspA-like [Trichomonas...     195  4e-50
 ref|XP_001580218.1| surface antigen BspA-like [Trichomonas...     195  5e-50
 ref|XP_001316679.1| surface antigen BspA-like [Trichomonas...     195  5e-50
 ref|XP_001583765.1| surface antigen BspA-like [Trichomonas...     194  8e-50
 ref|XP_001310095.1| Leucine Rich Repeat family protein [Tr...     194  8e-50
 ref|XP_001317421.1| surface antigen BspA-like [Trichomonas...     194  9e-50
 ref|XP_001583967.1| surface antigen BspA-like [Trichomonas...     194  1e-49
 ref|XP_001315446.1| surface antigen BspA-like [Trichomonas...     194  1e-49
 ref|XP_001315574.1| Leucine Rich Repeat family protein [Tr...     194  1e-49
 ref|XP_001329258.1| surface antigen BspA-like [Trichomonas...     194  2e-49
 ref|XP_001306944.1| surface antigen BspA-like [Trichomonas...     193  2e-49
 ref|XP_001303661.1| surface antigen BspA-like [Trichomonas...     193  3e-49
 ref|XP_001329934.1| surface antigen BspA-like [Trichomonas...     192  5e-49
 ref|XP_001328355.1| surface antigen BspA-like [Trichomonas...     191  6e-49
 ref|XP_001584307.1| surface antigen BspA-like [Trichomonas...     191  7e-49
 ref|XP_001329976.1| surface antigen BspA-like [Trichomonas...     191  7e-49
 ref|XP_001579847.1| surface antigen BspA-like [Trichomonas...     191  9e-49
 ref|XP_001328760.1| surface antigen BspA-like [Trichomonas...     191  1e-48
 ref|XP_001321548.1| surface antigen BspA-like [Trichomonas...     190  1e-48
 ref|XP_001328090.1| surface antigen BspA-like [Trichomonas...     190  1e-48
 ref|XP_001301818.1| surface antigen BspA-like [Trichomonas...     190  2e-48
 ref|XP_001325834.1| surface antigen BspA-like [Trichomonas...     190  2e-48
 ref|XP_001310233.1| surface antigen BspA-like [Trichomonas...     189  3e-48
 ref|XP_001314744.1| surface antigen BspA-like [Trichomonas...     189  5e-48
 ref|XP_001581039.1| surface antigen BspA-like [Trichomonas...     187  1e-47
 ref|XP_001302316.1| Leucine Rich Repeat family protein [Tr...     187  1e-47
 ref|XP_001309216.1| Leucine Rich Repeat family protein [Tr...     187  1e-47
 ref|XP_001584393.1| surface antigen BspA-like [Trichomonas...     187  2e-47
 ref|XP_001288325.1| surface antigen BspA-like [Trichomonas...     186  3e-47
 ref|XP_001583078.1| surface antigen BspA-like [Trichomonas...     186  3e-47
 ref|XP_001298474.1| surface antigen BspA-like [Trichomonas...     186  3e-47
 ref|XP_001326103.1| hypothetical protein TVAG_028330 [Tric...     186  3e-47
 ref|XP_001323987.1| surface antigen BspA-like [Trichomonas...     186  3e-47
 ref|XP_001297948.1| surface antigen BspA-like [Trichomonas...     185  4e-47
 ref|XP_001327210.1| surface antigen BspA-like [Trichomonas...     185  5e-47
 ref|XP_001584305.1| surface antigen BspA-like [Trichomonas...     185  5e-47
 ref|XP_001324305.1| surface antigen BspA-like [Trichomonas...     185  6e-47
 ref|XP_001311300.1| surface antigen BspA-like [Trichomonas...     185  6e-47
 ref|XP_001330651.1| surface antigen Bsp, putative [Trichom...     185  7e-47
 ref|XP_001294025.1| surface antigen BspA-like [Trichomonas...     184  9e-47
 ref|XP_001311197.1| surface antigen BspA-like [Trichomonas...     184  9e-47
 ref|XP_001306716.1| surface antigen BspA-like [Trichomonas...     184  1e-46
 ref|XP_001322412.1| surface antigen BspA-like [Trichomonas...     184  1e-46
 ref|XP_001326264.1| surface antigen BspA-like [Trichomonas...     184  1e-46
 ref|XP_001327192.1| hypothetical protein TVAG_396990 [Tric...     184  1e-46
 ref|XP_001312629.1| surface antigen BspA-like [Trichomonas...     184  2e-46
 ref|XP_001300473.1| surface antigen BspA-like [Trichomonas...     184  2e-46
 ref|XP_001583077.1| surface antigen BspA-like [Trichomonas...     183  2e-46
 ref|XP_001320691.1| hypothetical protein TVAG_145480 [Tric...     183  2e-46
 ref|XP_001307423.1| surface antigen BspA-like [Trichomonas...     183  2e-46
 ref|XP_001321495.1| conserved hypothetical protein [Tricho...     182  4e-46
 ref|XP_001289543.1| hypothetical protein TVAG_461990 [Tric...     182  4e-46
 ref|XP_001302593.1| surface antigen BspA-like [Trichomonas...     182  5e-46
 ref|XP_001302626.1| surface antigen BspA-like [Trichomonas...     182  5e-46
 ref|XP_001307287.1| surface antigen BspA-like [Trichomonas...     182  6e-46
 ref|XP_001297832.1| surface antigen BspA-like [Trichomonas...     181  7e-46
 ref|XP_001322526.1| Leucine Rich Repeat family protein [Tr...     181  7e-46
 ref|XP_001582616.1| hypothetical protein TVAG_013970 [Tric...     181  8e-46
 ref|XP_001310673.1| surface antigen BspA-like [Trichomonas...     181  8e-46
 ref|XP_001309065.1| surface antigen BspA-like [Trichomonas...     181  8e-46
 ref|XP_001328675.1| surface antigen BspA-like [Trichomonas...     181  9e-46
 ref|XP_001580822.1| Leucine Rich Repeat family protein [Tr...     181  9e-46
 ref|XP_001580665.1| surface antigen BspA-like [Trichomonas...     180  1e-45
 ref|XP_001327215.1| surface antigen BspA-like [Trichomonas...     180  1e-45
 ref|XP_001316678.1| surface antigen BspA-like [Trichomonas...     180  1e-45
 ref|XP_001309784.1| surface antigen BspA-like [Trichomonas...     180  2e-45
 ref|XP_001311188.1| surface antigen BspA-like [Trichomonas...     180  2e-45
 ref|XP_001285293.1| surface antigen BspA-like [Trichomonas...     180  2e-45
 ref|XP_001318079.1| surface antigen BspA-like [Trichomonas...     180  2e-45
 ref|XP_001328365.1| surface antigen BspA-like [Trichomonas...     180  2e-45
 ref|XP_001314937.1| surface antigen BspA-like [Trichomonas...     179  3e-45
 ref|XP_001306979.1| surface antigen BspA-like [Trichomonas...     179  3e-45
 ref|XP_001307211.1| surface antigen BspA-like [Trichomonas...     179  4e-45
 ref|XP_001327026.1| surface antigen BspA-like [Trichomonas...     179  4e-45
 ref|XP_001328364.1| hypothetical protein TVAG_465290 [Tric...     179  5e-45
 ref|XP_001309231.1| Leucine Rich Repeat family protein [Tr...     178  6e-45
 ref|XP_001316692.1| surface antigen BspA-like [Trichomonas...     178  9e-45
 ref|XP_001309824.1| surface antigen BspA-like [Trichomonas...     177  9e-45
 ref|XP_001318958.1| surface antigen BspA-like [Trichomonas...     177  1e-44
 ref|XP_001580420.1| surface antigen BspA-like [Trichomonas...     177  1e-44
 ref|XP_001310837.1| surface antigen BspA-like [Trichomonas...     177  2e-44
 ref|XP_001278277.1| surface antigen BspA-like [Trichomonas...     177  2e-44
 ref|XP_001327196.1| Leucine Rich Repeat family protein [Tr...     176  3e-44
 ref|XP_001308117.1| surface antigen BspA-like [Trichomonas...     176  3e-44
 ref|XP_001579737.1| surface antigen BspA-like [Trichomonas...     176  3e-44
 ref|XP_001312366.1| surface antigen BspA-like [Trichomonas...     176  3e-44
 ref|XP_001304718.1| hypothetical protein TVAG_176470 [Tric...     175  4e-44
 ref|XP_001325284.1| hypothetical protein TVAG_212430 [Tric...     175  5e-44
 ref|XP_001312905.1| surface antigen BspA-like [Trichomonas...     175  5e-44
 ref|XP_001309823.1| hypothetical protein TVAG_133930 [Tric...     175  6e-44
 ref|XP_001317520.1| hypothetical protein TVAG_336890 [Tric...     175  7e-44
 ref|XP_001323127.1| surface antigen Bsp, putative [Trichom...     175  7e-44
 ref|XP_001304472.1| hypothetical protein TVAG_376540 [Tric...     175  7e-44
 ref|XP_001328755.1| surface antigen BspA-like [Trichomonas...     175  7e-44
 ref|XP_001308920.1| hypothetical protein TVAG_052480 [Tric...     175  7e-44
 ref|XP_001583766.1| surface antigen BspA-like [Trichomonas...     174  1e-43
 ref|XP_001327214.1| Leucine Rich Repeat family protein [Tr...     174  1e-43
 ref|XP_001580440.1| surface antigen BspA-like [Trichomonas...     173  2e-43
 ref|XP_001280250.1| surface antigen BspA-like [Trichomonas...     172  4e-43
 ref|XP_001321504.1| surface antigen BspA-like [Trichomonas...     172  4e-43
 ref|XP_001299679.1| surface antigen BspA-like [Trichomonas...     172  4e-43
 ref|XP_001295040.1| surface antigen BspA-like [Trichomonas...     172  5e-43
 ref|XP_001329145.1| conserved hypothetical protein [Tricho...     172  5e-43
 ref|XP_001311048.1| surface antigen BspA-like [Trichomonas...     172  5e-43
 ref|XP_001309841.1| surface antigen BspA-like [Trichomonas...     172  6e-43
 ref|XP_001324335.1| surface antigen BspA-like [Trichomonas...     172  6e-43
 ref|XP_001312628.1| surface antigen BspA-like [Trichomonas...     172  6e-43
 ref|XP_001318545.1| surface antigen BspA-like [Trichomonas...     172  6e-43
 ref|XP_001302033.1| surface antigen BspA-like [Trichomonas...     172  7e-43
 ref|XP_001322571.1| hypothetical protein TVAG_109320 [Tric...     172  7e-43
 ref|XP_001323868.1| surface antigen BspA-like [Trichomonas...     171  7e-43
 ref|XP_001321498.1| hypothetical protein TVAG_133330 [Tric...     171  7e-43
 ref|XP_001323988.1| surface antigen BspA-like [Trichomonas...     170  1e-42
 ref|XP_001302594.1| surface antigen BspA-like [Trichomonas...     170  2e-42
 ref|XP_001317517.1| hypothetical protein TVAG_336860 [Tric...     170  2e-42
 ref|XP_001324621.1| surface antigen BspA-like [Trichomonas...     169  3e-42
 ref|XP_001327177.1| hypothetical protein TVAG_396840 [Tric...     169  4e-42
 ref|XP_001326595.1| surface antigen BspA-like [Trichomonas...     169  4e-42
 ref|XP_001582264.1| surface antigen BspA-like [Trichomonas...     169  4e-42
 ref|XP_001308119.1| surface antigen BspA-like [Trichomonas...     169  5e-42
 ref|XP_001581831.1| surface antigen BspA-like [Trichomonas...     169  5e-42
 ref|XP_001316192.1| surface antigen BspA-like [Trichomonas...     168  8e-42
 ref|XP_001330675.1| cell surface protein, putative [Tricho...     167  2e-41
 ref|XP_001305685.1| cell surface protein, putative [Tricho...     167  2e-41
 ref|XP_001317272.1| surface antigen BspA-like [Trichomonas...     166  2e-41
 ref|XP_001313118.1| surface antigen BspA-like [Trichomonas...     166  2e-41
 ref|XP_001311252.1| surface antigen BspA-like [Trichomonas...     166  3e-41
 ref|XP_001276928.1| surface antigen BspA-like [Trichomonas...     166  3e-41
 ref|XP_001302853.1| surface antigen BspA-like [Trichomonas...     166  3e-41
 ref|XP_001329840.1| surface antigen BspA-like [Trichomonas...     166  3e-41
 ref|XP_001314392.1| hypothetical protein TVAG_003000 [Tric...     166  3e-41
 ref|XP_001298530.1| surface antigen BspA-like [Trichomonas...     165  6e-41
 ref|XP_001301531.1| surface antigen BspA-like [Trichomonas...     165  6e-41
 ref|XP_001312390.1| surface antigen BspA-like [Trichomonas...     165  7e-41
 ref|XP_001319015.1| hypothetical protein TVAG_103420 [Tric...     164  1e-40
 ref|XP_001298123.1| hypothetical protein TVAG_043250 [Tric...     163  2e-40
 ref|XP_001327175.1| surface antigen BspA-like [Trichomonas...     163  2e-40
 ref|XP_001311187.1| surface antigen BspA-like [Trichomonas...     163  3e-40
 ref|XP_001581323.1| surface antigen BspA-like [Trichomonas...     162  3e-40
 ref|XP_001313119.1| surface antigen Bsp, putative [Trichom...     162  4e-40
 ref|XP_001582615.1| surface antigen BspA-like [Trichomonas...     162  6e-40
 ref|XP_001321506.1| hypothetical protein TVAG_133410 [Tric...     161  8e-40
 ref|XP_001305200.1| surface antigen BspA-like [Trichomonas...     161  9e-40
 ref|XP_001329820.1| surface antigen BspA-like [Trichomonas...     161  1e-39
 ref|XP_001583194.1| conserved hypothetical protein [Tricho...     161  1e-39
 ref|XP_001306877.1| surface antigen BspA-like [Trichomonas...     160  2e-39
 ref|XP_001322763.1| surface antigen Bsp, putative [Trichom...     159  3e-39
 ref|XP_001309347.1| surface antigen BspA-like [Trichomonas...     159  4e-39
 ref|XP_001330718.1| surface antigen BspA-like [Trichomonas...     158  5e-39
 ref|XP_001308231.1| surface antigen BspA-like [Trichomonas...     158  6e-39
 ref|XP_001319242.1| surface antigen BspA-like [Trichomonas...     158  1e-38
 ref|XP_001326169.1| surface antigen BspA-like [Trichomonas...     157  1e-38
 ref|XP_001327023.1| surface antigen BspA-like [Trichomonas...     157  1e-38
 ref|XP_001316659.1| surface antigen BspA-like [Trichomonas...     157  1e-38
 ref|XP_001323660.1| Leucine Rich Repeat family protein [Tr...     157  2e-38
 ref|XP_001312716.1| surface antigen BspA-like [Trichomonas...     157  2e-38
 ref|XP_001583197.1| surface antigen BspA-like [Trichomonas...     157  2e-38
 ref|XP_001303020.1| surface antigen BspA-like [Trichomonas...     157  2e-38
 ref|XP_001327189.1| surface antigen BspA-like [Trichomonas...     156  2e-38
 ref|XP_001308763.1| surface antigen BspA-like [Trichomonas...     156  2e-38
 ref|XP_001304114.1| surface antigen BspA-like [Trichomonas...     155  5e-38
 ref|XP_001583510.1| cell surface protein, putative [Tricho...     155  5e-38
 ref|XP_001307331.1| surface antigen Bsp, putative [Trichom...     155  5e-38
 ref|XP_001317247.1| surface antigen BspA-like [Trichomonas...     155  6e-38
 ref|XP_001302270.1| hypothetical protein TVAG_574760 [Tric...     155  6e-38
 ref|XP_001327395.1| surface antigen BspA-like [Trichomonas...     155  7e-38
 ref|XP_001328314.1| surface antigen BspA-like [Trichomonas...     155  7e-38
 ref|XP_001579421.1| surface antigen BspA-like [Trichomonas...     155  8e-38
 ref|XP_001308032.1| surface antigen BspA-like [Trichomonas...     155  8e-38
 ref|XP_001583196.1| conserved hypothetical protein [Tricho...     154  1e-37
 ref|XP_001328761.1| surface antigen BspA-like [Trichomonas...     154  1e-37
 ref|XP_001584396.1| surface antigen BspA-like [Trichomonas...     154  1e-37
 ref|XP_001579423.1| surface antigen BspA-like [Trichomonas...     154  1e-37
 ref|XP_001329822.1| surface antigen BspA-like [Trichomonas...     154  1e-37
 ref|XP_001327198.1| leucine Rich Repeat domain protein, pu...     153  2e-37
 ref|XP_001306309.1| surface antigen BspA-like [Trichomonas...     153  3e-37
 ref|XP_001302666.1| surface antigen Bsp, putative [Trichom...     153  3e-37
 ref|XP_001311500.1| surface antigen BspA-like [Trichomonas...     152  4e-37
 ref|XP_001323136.1| surface antigen BspA-like [Trichomonas...     152  5e-37
 ref|XP_001298588.1| hypothetical protein TVAG_145120 [Tric...     152  5e-37
 ref|XP_001308916.1| surface antigen Bsp, putative [Trichom...     152  6e-37
 ref|XP_001308917.1| surface antigen BspA-like [Trichomonas...     152  7e-37
 ref|XP_001287142.1| surface antigen BspA-like [Trichomonas...     151  8e-37
 ref|XP_001320681.1| surface antigen BspA-like [Trichomonas...     151  9e-37
 ref|XP_001296127.1| surface antigen BspA-like [Trichomonas...     151  9e-37
 ref|XP_001308225.1| surface antigen BspA-like [Trichomonas...     151  1e-36
 ref|XP_001312364.1| surface antigen BspA-like [Trichomonas...     151  1e-36
 ref|XP_001300665.1| surface antigen BspA-like [Trichomonas...     150  1e-36
 ref|XP_001308939.1| surface antigen BspA-like [Trichomonas...     150  2e-36
 ref|XP_001318798.1| surface antigen BspA-like [Trichomonas...     149  3e-36
 ref|XP_001323863.1| surface antigen BspA-like [Trichomonas...     149  3e-36
 ref|XP_001308403.1| surface antigen BspA-like [Trichomonas...     149  3e-36
 ref|XP_001328762.1| surface antigen BspA-like [Trichomonas...     149  3e-36
 ref|XP_001322573.1| surface antigen BspA-like [Trichomonas...     149  3e-36
 ref|XP_001311149.1| surface antigen BspA-like [Trichomonas...     149  4e-36
 ref|XP_001327213.1| surface antigen BspA-like [Trichomonas...     149  4e-36
 ref|XP_001323126.1| hypothetical protein TVAG_259880 [Tric...     149  4e-36
 ref|XP_001327726.1| surface antigen BspA-like [Trichomonas...     149  4e-36
 ref|XP_001326623.1| hypothetical protein TVAG_255920 [Tric...     148  5e-36
 ref|XP_001302030.1| surface antigen BspA-like [Trichomonas...     148  5e-36
 ref|XP_001317271.1| surface antigen BspA-like [Trichomonas...     148  5e-36
 ref|XP_001306375.1| surface antigen BspA-like [Trichomonas...     148  6e-36
 ref|XP_001315805.1| hypothetical protein TVAG_042340 [Tric...     147  1e-35
 ref|XP_001312181.1| surface antigen BspA-like [Trichomonas...     147  1e-35
 ref|XP_001321508.1| conserved hypothetical protein [Tricho...     147  2e-35
 ref|XP_001304113.1| surface antigen BspA-like [Trichomonas...     146  3e-35
 ref|XP_001581685.1| surface antigen BspA-like [Trichomonas...     146  3e-35
 ref|XP_001299680.1| hypothetical protein TVAG_236800 [Tric...     146  3e-35
 ref|XP_001303835.1| surface antigen BspA-like [Trichomonas...     145  5e-35
 ref|XP_001327211.1| Leucine Rich Repeat family protein [Tr...     145  6e-35
 ref|XP_001580439.1| surface antigen BspA-like [Trichomonas...     145  6e-35
 ref|XP_001328361.1| surface antigen BspA-like [Trichomonas...     144  1e-34
 ref|XP_001291104.1| surface antigen BspA-like [Trichomonas...     143  2e-34
 ref|XP_001316621.1| surface antigen BspA-like [Trichomonas...     143  3e-34
 ref|XP_001317519.1| surface antigen BspA-like [Trichomonas...     143  3e-34
 ref|XP_001292036.1| surface antigen Bsp, putative [Trichom...     142  4e-34
 ref|XP_001327024.1| surface antigen BspA-like [Trichomonas...     142  4e-34
 ref|XP_001323860.1| hypothetical protein TVAG_158640 [Tric...     142  4e-34
 ref|XP_001581244.1| Leucine Rich Repeat family protein [Tr...     142  5e-34
 ref|XP_001303564.1| surface antigen BspA-like [Trichomonas...     142  7e-34
 ref|XP_001313250.1| surface antigen BspA-like [Trichomonas...     141  8e-34
 ref|XP_001321494.1| surface antigen BspA-like [Trichomonas...     141  8e-34
 ref|XP_001326262.1| surface antigen BspA-like [Trichomonas...     141  9e-34
 ref|XP_001318789.1| surface antigen BspA-like [Trichomonas...     140  1e-33
 ref|XP_001315766.1| hypothetical protein TVAG_041950 [Tric...     140  2e-33
 ref|XP_001313444.1| hypothetical protein TVAG_350110 [Tric...     139  4e-33
 ref|XP_001580028.1| surface antigen BspA-like [Trichomonas...     139  4e-33
 ref|XP_001583035.1| hypothetical protein TVAG_456840 [Tric...     139  4e-33
 ref|XP_001309449.1| hypothetical protein TVAG_148710 [Tric...     138  5e-33
 ref|XP_001299649.1| hypothetical protein TVAG_082800 [Tric...     138  1e-32
 ref|XP_001330631.1| surface antigen Bsp, putative [Trichom...     137  1e-32
 ref|XP_001581245.1| Leucine Rich Repeat family protein [Tr...     137  1e-32
 ref|XP_001583549.1| Leucine Rich Repeat family protein [Tr...     137  1e-32
 ref|XP_001583618.1| cell surface protein, putative [Tricho...     137  2e-32
 ref|XP_001310732.1| conserved hypothetical protein [Tricho...     137  2e-32
 ref|XP_001318637.1| choline binding protein, putative [Tri...     137  2e-32
 ref|XP_001579305.1| surface antigen BspA-like [Trichomonas...     137  2e-32
 ref|XP_001303553.1| surface antigen BspA-like [Trichomonas...     137  2e-32
 ref|XP_001302271.1| surface antigen BspA-like [Trichomonas...     137  2e-32
 ref|XP_001304475.1| Leucine Rich Repeat family protein [Tr...     137  2e-32
 ref|XP_001302341.1| Leucine Rich Repeat family protein [Tr...     136  3e-32
 ref|XP_001329016.1| surface antigen BspA-like [Trichomonas...     136  3e-32
 ref|XP_001326355.1| surface antigen BspA-like [Trichomonas...     135  4e-32
 ref|XP_001578887.1| surface antigen BspA-like [Trichomonas...     135  4e-32
 ref|XP_001584246.1| hypothetical protein TVAG_185670 [Tric...     135  6e-32
 ref|XP_001325056.1| surface antigen BspA-like [Trichomonas...     135  8e-32
 ref|XP_001310106.1| hypothetical protein TVAG_355370 [Tric...     133  2e-31
 ref|XP_001328354.1| surface antigen BspA-like [Trichomonas...     133  2e-31
 ref|XP_001311339.1| hypothetical protein TVAG_125530 [Tric...     133  2e-31
 ref|XP_001321487.1| hypothetical protein TVAG_133220 [Tric...     133  2e-31
 ref|XP_001312719.1| surface antigen BspA-like [Trichomonas...     133  2e-31
 ref|XP_001322414.1| conserved hypothetical protein [Tricho...     133  3e-31
 ref|XP_001311619.1| surface antigen BspA-like [Trichomonas...     132  5e-31
 ref|XP_001327996.1| surface antigen BspA-like [Trichomonas...     131  8e-31
 ref|XP_001319016.1| surface antigen BspA-like [Trichomonas...     131  1e-30
 ref|XP_001582620.1| surface antigen BspA-like [Trichomonas...     131  1e-30
 ref|XP_001327711.1| surface antigen BspA-like [Trichomonas...     131  1e-30
 ref|XP_001317246.1| surface antigen BspA-like [Trichomonas...     130  2e-30
 ref|XP_001327025.1| surface antigen BspA-like [Trichomonas...     130  2e-30
 ref|XP_001313865.1| hypothetical protein TVAG_493090 [Tric...     130  2e-30
 ref|XP_001316693.1| hypothetical protein TVAG_194350 [Tric...     129  3e-30
 ref|XP_001321496.1| surface antigen BspA-like [Trichomonas...     129  4e-30
 ref|XP_001329710.1| surface antigen BspA-like [Trichomonas...     128  6e-30
 ref|XP_001582100.1| surface antigen BspA-like [Trichomonas...     128  6e-30
 ref|XP_001330058.1| surface antigen BspA-like [Trichomonas...     128  7e-30
 ref|XP_001310120.1| surface antigen BspA-like [Trichomonas...     128  7e-30
 ref|XP_001328291.1| hypothetical protein TVAG_278340 [Tric...     127  1e-29
 ref|XP_001316690.1| surface antigen BspA-like [Trichomonas...     127  1e-29
 ref|XP_001321962.1| hypothetical protein TVAG_413890 [Tric...     127  2e-29
 ref|XP_001323205.1| surface antigen BspA-like [Trichomonas...     127  2e-29
 ref|XP_001327006.1| surface antigen BspA-like [Trichomonas...     127  2e-29
 ref|XP_001316574.1| surface antigen BspA-like [Trichomonas...     127  2e-29
 ref|XP_001324036.1| hypothetical protein TVAG_458850 [Tric...     126  3e-29
 ref|XP_001321507.1| cell surface protein, putative [Tricho...     126  3e-29
 ref|XP_001303837.1| surface antigen BspA-like [Trichomonas...     125  4e-29
 ref|XP_001330674.1| surface antigen BspA-like [Trichomonas...     125  7e-29
 ref|XP_001313120.1| hypothetical protein TVAG_007210 [Tric...     124  1e-28
 ref|XP_001322415.1| surface antigen BspA-like [Trichomonas...     124  1e-28
 ref|XP_001326539.1| surface antigen BspA-like [Trichomonas...     123  2e-28
 ref|XP_001583565.1| surface antigen BspA-like [Trichomonas...     123  2e-28
 ref|XP_001307216.1| surface antigen BspA-like [Trichomonas...     123  3e-28
 ref|XP_001308918.1| hypothetical protein TVAG_052460 [Tric...     122  4e-28
 ref|XP_001317275.1| surface antigen BspA-like [Trichomonas...     122  6e-28
 ref|XP_001325055.1| hypothetical protein TVAG_221850 [Tric...     122  6e-28
 ref|XP_001303506.1| leucine-rich repeat protein, putative ...     122  8e-28
 ref|XP_001323088.1| surface antigen BspA-like [Trichomonas...     121  8e-28
 ref|XP_001328327.1| conserved hypothetical protein [Tricho...     121  9e-28
 ref|XP_001328647.1| surface antigen BspA-like [Trichomonas...     121  1e-27
 ref|XP_001327178.1| conserved hypothetical protein [Tricho...     121  1e-27
 ref|XP_001297260.1| surface antigen BspA-like [Trichomonas...     121  1e-27
 ref|XP_001319221.1| hypothetical protein TVAG_174670 [Tric...     120  2e-27
 ref|XP_001316126.1| hypothetical protein TVAG_192020 [Tric...     120  2e-27
 ref|XP_001579824.1| surface antigen BspA-like [Trichomonas...     120  3e-27
 ref|XP_001297833.1| surface antigen Bsp, putative [Trichom...     119  4e-27
 ref|XP_001583617.1| cell surface protein, putative [Tricho...     118  8e-27
 ref|XP_001309171.1| hypothetical protein TVAG_205270 [Tric...     118  1e-26
 ref|XP_001320609.1| surface antigen Bsp, putative [Trichom...     117  2e-26
 ref|XP_001294926.1| surface antigen BspA-like [Trichomonas...     117  2e-26
 ref|XP_001326631.1| surface antigen Bsp, putative [Trichom...     116  3e-26
 ref|XP_001321632.1| surface antigen BspA-like [Trichomonas...     116  3e-26
 ref|XP_001321489.1| surface antigen BspA-like [Trichomonas...     116  3e-26
 ref|XP_001580846.1| surface antigen Bsp, putative [Trichom...     116  4e-26
 ref|XP_001321501.1| hypothetical protein TVAG_133360 [Tric...     115  5e-26
 ref|XP_001321493.1| surface antigen BspA-like [Trichomonas...     115  7e-26
 ref|XP_001304391.1| surface antigen BspA-like [Trichomonas...     115  8e-26
 ref|XP_001321488.1| cell surface protein, putative [Tricho...     115  9e-26
 ref|XP_001321492.1| cell surface protein, putative [Tricho...     115  9e-26
 ref|XP_001321488.1| cell surface protein, putative [Tricho...     104  1e-22
 ref|XP_001321492.1| cell surface protein, putative [Tricho...     104  1e-22
 ref|XP_001321488.1| cell surface protein, putative [Tricho...      97  2e-20
 ref|XP_001321492.1| cell surface protein, putative [Tricho...      97  2e-20
 ref|XP_001321488.1| cell surface protein, putative [Tricho...      88  8e-18
 ref|XP_001321492.1| cell surface protein, putative [Tricho...      88  8e-18
 ref|XP_001321488.1| cell surface protein, putative [Tricho...      84  1e-16
 ref|XP_001321492.1| cell surface protein, putative [Tricho...      84  1e-16
 ref|XP_001321488.1| cell surface protein, putative [Tricho...      70  3e-12
 ref|XP_001321492.1| cell surface protein, putative [Tricho...      70  3e-12
 ref|XP_001582999.1| hypothetical protein TVAG_456480 [Tric...     115  9e-26
 ref|XP_001322332.1| hypothetical protein TVAG_274720 [Tric...     115  9e-26
 ref|XP_001322364.1| surface antigen BspA-like [Trichomonas...     114  1e-25
 ref|XP_001315545.1| surface antigen BspA-like [Trichomonas...     114  1e-25
 ref|XP_001312182.1| conserved hypothetical protein [Tricho...     114  1e-25
 ref|XP_001323593.1| hypothetical protein TVAG_379520 [Tric...     114  2e-25
 ref|XP_001301412.1| surface antigen BspA-like [Trichomonas...     113  2e-25
 ref|XP_001329975.1| hypothetical protein TVAG_010640 [Tric...     113  2e-25
 ref|XP_001303834.1| hypothetical protein TVAG_228050 [Tric...     113  2e-25
 ref|XP_001301144.1| hypothetical protein TVAG_043300 [Tric...     113  2e-25
 ref|XP_001311207.1| cell surface protein, putative [Tricho...     113  3e-25
 ref|XP_001316575.1| hypothetical protein TVAG_070020 [Tric...     112  4e-25
 ref|XP_001318908.1| hypothetical protein TVAG_211370 [Tric...     112  4e-25
 ref|XP_001308538.1| hypothetical protein TVAG_459570 [Tric...     112  7e-25
 ref|XP_001304034.1| hypothetical protein TVAG_308640 [Tric...     112  8e-25
 ref|XP_001325359.1| Leucine Rich Repeat family protein [Tr...     111  1e-24
 ref|XP_001306608.1| Leucine Rich Repeat family protein [Tr...     111  1e-24
 ref|XP_001327007.1| Leucine Rich Repeat family protein [Tr...     111  1e-24
 ref|XP_001580219.1| surface antigen BspA-like [Trichomonas...     110  2e-24
 ref|XP_001306747.1| surface antigen BspA-like [Trichomonas...     109  4e-24
 ref|XP_001582896.1| surface antigen BspA-like [Trichomonas...     109  4e-24
 ref|XP_001330639.1| cell surface protein, putative [Tricho...     109  4e-24
 ref|XP_001321191.1| surface antigen BspA-like [Trichomonas...     109  4e-24
 ref|XP_001582998.1| surface antigen Bsp, putative [Trichom...     109  5e-24
 ref|XP_001326263.1| surface antigen BspA-like [Trichomonas...     109  5e-24
 ref|XP_001303019.1| hypothetical protein TVAG_396460 [Tric...     108  6e-24
 ref|XP_001309448.1| hypothetical protein TVAG_148700 [Tric...     108  6e-24
 ref|XP_001330717.1| hypothetical protein TVAG_319430 [Tric...     108  8e-24
 ref|XP_001307288.1| surface antigen BspA-like [Trichomonas...     108  1e-23
 ref|XP_001309066.1| surface antigen BspA-like [Trichomonas...     107  1e-23
 ref|XP_001581067.1| hypothetical protein TVAG_365890 [Tric...     107  2e-23
 ref|XP_001315613.1| surface antigen BspA-like [Trichomonas...     106  2e-23
 ref|XP_001322410.1| surface antigen BspA-like [Trichomonas...     106  3e-23
 ref|XP_001311473.1| surface antigen BspA-like [Trichomonas...     105  5e-23
 ref|XP_001317012.1| surface antigen BspA-like [Trichomonas...     105  5e-23
 ref|XP_001322572.1| hypothetical protein TVAG_109330 [Tric...     105  7e-23
 ref|XP_001581507.1| surface antigen BspA-like [Trichomonas...     105  1e-22
 ref|XP_001314764.1| surface antigen BspA-like [Trichomonas...     105  1e-22
 ref|XP_001326630.1| surface antigen BspA-like [Trichomonas...     104  1e-22
 ref|XP_001309358.1| surface antigen BspA-like [Trichomonas...     103  2e-22
 ref|XP_001325236.1| Leucine Rich Repeat family protein [Tr...     103  2e-22
 ref|XP_001310696.1| surface antigen BspA-like [Trichomonas...     103  3e-22
 ref|XP_001321545.1| hypothetical protein TVAG_394940 [Tric...     102  5e-22
 ref|XP_001306943.1| hypothetical protein TVAG_082200 [Tric...     102  5e-22
 ref|XP_001297423.1| surface antigen Bsp, putative [Trichom...     102  5e-22
 ref|XP_001303551.1| hypothetical protein TVAG_437650 [Tric...     101  1e-21
 ref|XP_001315598.1| surface antigen Bsp, putative [Trichom...     100  2e-21
 ref|XP_001295073.1| surface antigen BspA-like [Trichomonas...     100  2e-21
 ref|XP_001310672.1| hypothetical protein TVAG_080020 [Tric...     100  3e-21
 ref|XP_001321491.1| cell surface protein, putative [Tricho...     100  3e-21
 ref|XP_001306532.1| cell surface protein, putative [Tricho...      99  4e-21
 ref|XP_001580823.1| surface antigen BspA-like [Trichomonas...      99  4e-21
 ref|XP_001306980.1| surface antigen BspA-like [Trichomonas...      98  8e-21
 ref|XP_001583567.1| surface antigen BspA-like [Trichomonas...      98  1e-20
 ref|XP_001305201.1| surface antigen BspA-like [Trichomonas...      97  1e-20
 ref|XP_001316191.1| hypothetical protein TVAG_314980 [Tric...      97  2e-20
 ref|XP_001584394.1| cell surface protein, putative [Tricho...      96  2e-20
 ref|XP_001323659.1| surface antigen BspA-like [Trichomonas...      96  3e-20
 ref|XP_001300472.1| hypothetical protein TVAG_329770 [Tric...      96  3e-20
 ref|XP_001329423.1| hypothetical protein TVAG_291520 [Tric...      96  4e-20
 ref|XP_001311049.1| surface antigen BspA-like [Trichomonas...      95  6e-20
 ref|XP_001312048.1| surface antigen BspA-like [Trichomonas...      95  7e-20
 ref|XP_001328672.1| cell surface protein, putative [Tricho...      95  1e-19
 ref|XP_001328290.1| hypothetical protein TVAG_278330 [Tric...      94  1e-19
 ref|XP_001309840.1| surface antigen Bsp, putative [Trichom...      93  2e-19
 ref|XP_001319648.1| hypothetical protein TVAG_419670 [Tric...      93  2e-19
 ref|XP_001583511.1| hypothetical protein TVAG_035480 [Tric...      93  2e-19
 ref|XP_001308928.1| surface antigen BspA-like [Trichomonas...      93  3e-19
 ref|XP_001583462.1| hypothetical protein TVAG_034990 [Tric...      92  4e-19
 ref|XP_001286695.1| surface antigen BspA-like [Trichomonas...      92  4e-19
 ref|XP_001314378.1| Leucine Rich Repeat family protein [Tr...      91  9e-19
 ref|XP_001318169.1| conserved hypothetical protein [Tricho...      91  1e-18
 ref|XP_001325360.1| surface antigen BspA-like [Trichomonas...      90  2e-18
 ref|XP_001327176.1| hypothetical protein TVAG_396830 [Tric...      90  2e-18
 ref|XP_001328759.1| conserved hypothetical protein [Tricho...      90  2e-18
 ref|XP_001308086.1| surface antigen BspA-like [Trichomonas...      90  2e-18
 ref|XP_001309068.1| surface antigen BspA-like [Trichomonas...      90  2e-18
 ref|XP_001316381.1| conserved hypothetical protein [Tricho...      90  3e-18
 ref|XP_001319646.1| hypothetical protein TVAG_419650 [Tric...      88  9e-18
 ref|XP_001319490.1| surface antigen BspA-like [Trichomonas...      87  2e-17
 ref|XP_001321499.1| surface antigen BspA-like [Trichomonas...      86  3e-17
 ref|XP_001311301.1| Leucine Rich Repeat family protein [Tr...      86  4e-17
 ref|XP_001308723.1| hypothetical protein TVAG_354050 [Tric...      86  5e-17
 ref|XP_001323135.1| hypothetical protein TVAG_259970 [Tric...      85  5e-17
 ref|XP_001307206.1| hypothetical protein TVAG_057770 [Tric...      85  1e-16
 ref|XP_001582703.1| conserved hypothetical protein [Tricho...      84  1e-16
 ref|XP_001584313.1| surface antigen BspA-like [Trichomonas...      84  1e-16
 ref|XP_001307333.1| hypothetical protein TVAG_194890 [Tric...      84  1e-16
 ref|XP_001317516.1| hypothetical protein TVAG_336850 [Tric...      84  2e-16
 ref|XP_001315612.1| surface antigen Bsp, putative [Trichom...      83  3e-16
 ref|XP_001583619.1| hypothetical protein TVAG_475610 [Tric...      83  3e-16
 ref|XP_001318272.1| conserved hypothetical protein [Tricho...      83  3e-16
 ref|XP_001313249.1| surface antigen BspA-like [Trichomonas...      82  4e-16
 ref|XP_001327805.1| surface antigen BspA-like [Trichomonas...      82  7e-16
 ref|XP_001321503.1| cell surface protein, putative [Tricho...      81  9e-16
 ref|XP_001328766.1| cell surface protein, putative [Tricho...      80  2e-15
 ref|XP_001308179.1| hypothetical protein TVAG_149670 [Tric...      80  2e-15
 ref|XP_001321874.1| surface antigen BspA-like [Trichomonas...      80  2e-15
 ref|XP_001317644.1| surface antigen BspA-like [Trichomonas...      79  5e-15
 ref|XP_001312365.1| hypothetical protein TVAG_408450 [Tric...      79  6e-15
 ref|XP_001310877.1| hypothetical protein TVAG_316120 [Tric...      78  6e-15
 ref|XP_001579565.1| conserved hypothetical protein [Tricho...      78  1e-14
 ref|XP_001322368.1| surface antigen BspA-like [Trichomonas...      77  1e-14
 ref|XP_001580297.1| hypothetical protein TVAG_452250 [Tric...      77  2e-14
 ref|XP_001315245.1| conserved hypothetical protein [Tricho...      76  2e-14
 ref|XP_001311507.1| hypothetical protein TVAG_429310 [Tric...      76  3e-14
 ref|XP_001317491.1| surface antigen Bsp, putative [Trichom...      76  4e-14
 ref|XP_001313368.1| hypothetical protein TVAG_084760 [Tric...      76  5e-14
 ref|XP_001306510.1| surface antigen BspA-like [Trichomonas...      75  6e-14
 ref|XP_001581618.1| conserved hypothetical protein [Tricho...      75  6e-14
 ref|XP_001315682.1| conserved hypothetical protein [Tricho...      75  7e-14
 ref|XP_001304473.1| hypothetical protein TVAG_376550 [Tric...      75  8e-14
 ref|XP_001308226.1| surface antigen BspA-like [Trichomonas...      75  8e-14
 ref|XP_001308905.1| hypothetical protein TVAG_114860 [Tric...      74  1e-13
 ref|XP_001584263.1| surface antigen BspA-like [Trichomonas...      73  2e-13
 ref|XP_001325235.1| Leucine Rich Repeat family protein [Tr...      73  2e-13
 ref|XP_001312720.1| Leucine Rich Repeat family protein [Tr...      73  3e-13
 ref|XP_001325237.1| hypothetical protein TVAG_077520 [Tric...      73  4e-13
 ref|XP_001306723.1| surface antigen BspA-like [Trichomonas...      73  4e-13
 ref|XP_001584125.1| hypothetical protein TVAG_183760 [Tric...      73  4e-13
 ref|XP_001309583.1| surface antigen Bsp, putative [Trichom...      72  6e-13
 ref|XP_001311740.1| hypothetical protein TVAG_479820 [Tric...      72  6e-13
 ref|XP_001320873.1| conserved hypothetical protein [Tricho...      72  6e-13
 ref|XP_001584441.1| hypothetical protein TVAG_071190 [Tric...      71  1e-12
 ref|XP_001318270.1| serine-rich protein, putative [Trichom...      71  1e-12
 ref|XP_001309584.1| surface antigen Bsp, putative [Trichom...      71  1e-12
 ref|XP_001279743.1| Leucine Rich Repeat family protein [Tr...      71  1e-12
 ref|XP_001315611.1| Leucine Rich Repeat family protein [Tr...      71  1e-12
 ref|XP_001312324.1| hypothetical protein TVAG_043020 [Tric...      70  2e-12
 ref|XP_001324976.1| cell surface protein, putative [Tricho...      70  3e-12
 ref|XP_001312376.1| hypothetical protein TVAG_052890 [Tric...      70  3e-12
 ref|XP_001292683.1| hypothetical protein TVAG_042450 [Tric...      69  5e-12
 ref|XP_001306511.1| hypothetical protein TVAG_382260 [Tric...      69  6e-12
 ref|XP_001318045.1| hypothetical protein TVAG_202800 [Tric...      68  6e-12
 ref|XP_001328162.1| hypothetical protein TVAG_165670 [Tric...      68  9e-12
 ref|XP_001328158.1| surface antigen BspA-like [Trichomonas...      68  1e-11
 ref|XP_001285581.1| hypothetical protein TVAG_032340 [Tric...      68  1e-11
 ref|XP_001302036.1| surface protein, putative [Trichomonas...      68  1e-11
 ref|XP_001296750.1| hypothetical protein TVAG_419330 [Tric...      68  1e-11
 ref|XP_001306308.1| hypothetical protein TVAG_406420 [Tric...      67  2e-11
 ref|XP_001582094.1| surface antigen BspA-like [Trichomonas...      67  2e-11
 ref|XP_001328765.1| hypothetical protein TVAG_348520 [Tric...      66  2e-11
 ref|XP_001307419.1| surface antigen BspA-like [Trichomonas...      66  3e-11
 ref|XP_001324304.1| hypothetical protein TVAG_039290 [Tric...      66  3e-11
 ref|XP_001306188.1| surface antigen BspA-like [Trichomonas...      66  3e-11
 ref|XP_001582338.1| cell surface protein, putative [Tricho...      66  4e-11
 ref|XP_001327760.1| hypothetical protein TVAG_495520 [Tric...      66  5e-11
 ref|XP_001311506.1| hypothetical protein TVAG_429300 [Tric...      66  5e-11
 ref|XP_001584190.1| hypothetical protein TVAG_185100 [Tric...      65  6e-11
 ref|XP_001327121.1| hypothetical protein TVAG_380080 [Tric...      65  8e-11
 ref|XP_001317511.1| surface antigen BspA-like [Trichomonas...      65  1e-10
 ref|XP_001308118.1| surface protein, putative [Trichomonas...      64  1e-10
 ref|XP_001323934.1| hypothetical protein TVAG_488130 [Tric...      64  2e-10
 ref|XP_001307705.1| hypothetical protein TVAG_058650 [Tric...      64  2e-10
 ref|XP_001305041.1| conserved hypothetical protein [Tricho...      63  2e-10
 ref|XP_001299246.1| hypothetical protein TVAG_450350 [Tric...      63  3e-10
 ref|XP_001302448.1| hypothetical protein TVAG_372300 [Tric...      62  5e-10
 ref|XP_001583512.1| hypothetical protein TVAG_035490 [Tric...      62  6e-10
 ref|XP_001298562.1| hypothetical protein TVAG_602250 [Tric...      62  7e-10
 ref|XP_001302449.1| surface protein, putative [Trichomonas...      62  7e-10
 ref|XP_001305336.1| surface antigen BspA-like [Trichomonas...      61  1e-09
 ref|XP_001323867.1| surface antigen BspA-like [Trichomonas...      61  1e-09
 ref|XP_001312261.1| hypothetical protein TVAG_181880 [Tric...      61  1e-09
 ref|XP_001582517.1| surface antigen BspA-like [Trichomonas...      61  2e-09
 ref|XP_001582454.1| hypothetical protein TVAG_199070 [Tric...      60  2e-09
 ref|XP_001310343.1| hypothetical protein TVAG_437910 [Tric...      60  2e-09
 ref|XP_001580175.1| surface antigen BspA-like [Trichomonas...      60  2e-09
 ref|XP_001325481.1| hypothetical protein TVAG_463920 [Tric...      60  2e-09
 ref|XP_001323869.1| hypothetical protein TVAG_158730 [Tric...      60  3e-09
 ref|XP_001583863.1| surface antigen BspA-like [Trichomonas...      60  4e-09
 ref|XP_001327488.1| leucine Rich Repeat domain protein, pu...      60  4e-09
 ref|XP_001317518.1| hypothetical protein TVAG_336870 [Tric...      58  1e-08
 ref|XP_001310680.1| hypothetical protein TVAG_080100 [Tric...      58  1e-08
 ref|XP_001315557.1| cell surface protein, putative [Tricho...      58  1e-08
 ref|XP_001583864.1| surface antigen BspA-like [Trichomonas...      57  2e-08
 ref|XP_001304001.1| surface antigen Bsp, putative [Trichom...      57  2e-08
 ref|XP_001305684.1| cell surface protein, putative [Tricho...      56  3e-08
 ref|XP_001582994.1| hypothetical protein TVAG_456430 [Tric...      56  3e-08
 ref|XP_001328648.1| hypothetical protein TVAG_004560 [Tric...      56  4e-08
 ref|XP_001308540.1| surface antigen BspA-like [Trichomonas...      56  4e-08
 ref|XP_001308762.1| surface antigen BspA-like [Trichomonas...      55  7e-08
 ref|XP_001311503.1| hypothetical protein TVAG_429260 [Tric...      55  7e-08
 ref|XP_001324214.1| cell surface protein, putative [Tricho...      55  9e-08
 ref|XP_001304357.1| hypothetical protein TVAG_484410 [Tric...      54  1e-07
 ref|XP_001310182.1| hypothetical protein TVAG_037590 [Tric...      54  1e-07
 ref|XP_001317632.1| hypothetical protein TVAG_197100 [Tric...      54  1e-07
 ref|XP_001327804.1| choline binding protein, putative [Tri...      54  1e-07
 ref|XP_001323864.1| hypothetical protein TVAG_158680 [Tric...      54  2e-07
 ref|XP_001323634.1| hypothetical protein TVAG_418480 [Tric...      53  3e-07
 ref|XP_001302034.1| hypothetical protein TVAG_442610 [Tric...      53  3e-07
 ref|XP_001323504.1| hypothetical protein TVAG_061850 [Tric...      53  3e-07
 ref|XP_001327114.1| cell surface protein, putative [Tricho...      53  4e-07
 ref|XP_001302451.1| hypothetical protein TVAG_372330 [Tric...      52  6e-07
 ref|XP_001320964.1| hypothetical protein TVAG_251670 [Tric...      52  6e-07
 ref|XP_001328756.1| hypothetical protein TVAG_348430 [Tric...      51  1e-06
 ref|XP_001308230.1| leucine-rich repeat protein, putative ...      51  1e-06
 ref|XP_001321631.1| hypothetical protein TVAG_420300 [Tric...      51  2e-06
 ref|XP_001581659.1| hypothetical protein TVAG_163690 [Tric...      50  2e-06
 ref|XP_001300177.1| hypothetical protein TVAG_116420 [Tric...      49  4e-06
 ref|XP_001326624.1| hypothetical protein TVAG_255930 [Tric...      49  4e-06
 ref|XP_001310681.1| hypothetical protein TVAG_080110 [Tric...      49  4e-06
 ref|XP_001311834.1| hypothetical protein TVAG_210990 [Tric...      49  5e-06
 ref|XP_001579422.1| hypothetical protein TVAG_046400 [Tric...      48  7e-06
 ref|XP_001309232.1| hypothetical protein TVAG_264260 [Tric...      48  8e-06
 ref|XP_001302450.1| hypothetical protein TVAG_372320 [Tric...      48  1e-05
```

---

**Taxonomy Report**

```
Trichomonas vaginalis G3 .  5419 hits    1 orgs [root; cellular organisms; Eukaryota; Parabasalidea; Trichomonada; Trichomonadida; Trichomonadidae; Trichomonadinae; Trichomonas; Trichomonas vaginalis]
```

---


### 2) Entamoeba

**Lineage Report**  

```
Entamoeba [eukaryotes]
. Entamoeba dispar SAW760 ---------  439 2213 hits [eukaryotes]  hypothetical protein, conserved [Entamoeba dispar SAW760]
. Entamoeba histolytica HM-1:IMSS .  361  930 hits [eukaryotes]  leucine rich repeat protein, BspA family [Entamoeba histoly
```

---

**Organism Report**

```
  Entamoeba dispar SAW760 [eukaryotes] taxid 370354
 ref|XP_001741794.1| hypothetical protein, conserved [Entam...     439  6e-124
 ref|XP_001735764.1| hypothetical protein, conserved [Entam...     405  2e-113
 ref|XP_001741679.1| hypothetical protein, conserved [Entam...     400  4e-112
 ref|XP_001740405.1| hypothetical protein, conserved [Entam...     394  3e-110
 ref|XP_001733882.1| hypothetical protein, conserved [Entam...     378  2e-105
 ref|XP_001734626.1| hypothetical protein, conserved [Entam...     376  9e-105
 ref|XP_001734805.1| hypothetical protein, conserved [Entam...     372  1e-103
 ref|XP_001733663.1| hypothetical protein, conserved [Entam...     372  1e-103
 ref|XP_001736002.1| hypothetical protein EDI_001030 [Entam...     368  2e-102
 ref|XP_001741795.1| hypothetical protein, conserved [Entam...     367  5e-102
 ref|XP_001738834.1| hypothetical protein, conserved [Entam...     363  6e-101
 ref|XP_001741567.1| hypothetical protein, conserved [Entam...     363  9e-101
 ref|XP_001741028.1| hypothetical protein, conserved [Entam...     362  1e-100
 ref|XP_001738617.1| hypothetical protein, conserved [Entam...     357  5e-99
 ref|XP_001735765.1| hypothetical protein, conserved [Entam...     354  3e-98
 ref|XP_001741825.1| hypothetical protein, conserved [Entam...     354  3e-98
 ref|XP_001735361.1| hypothetical protein, conserved [Entam...     354  4e-98
 ref|XP_001734451.1| hypothetical protein, conserved [Entam...     353  5e-98
 ref|XP_001733318.1| hypothetical protein, conserved [Entam...     350  8e-97
 ref|XP_001733887.1| hypothetical protein, conserved [Entam...     346  1e-95
 ref|XP_001741894.1| hypothetical protein, conserved [Entam...     346  1e-95
 ref|XP_001739223.1| hypothetical protein, conserved [Entam...     343  8e-95
 ref|XP_001734935.1| hypothetical protein, conserved [Entam...     339  1e-93
 ref|XP_001733746.1| hypothetical protein, conserved [Entam...     339  1e-93
 ref|XP_001740612.1| hypothetical protein, conserved [Entam...     338  2e-93
 ref|XP_001735475.1| hypothetical protein, conserved [Entam...     338  2e-93
 ref|XP_001734609.1| hypothetical protein, conserved [Entam...     336  8e-93
 ref|XP_001739690.1| hypothetical protein, conserved [Entam...     328  2e-90
 ref|XP_001735447.1| hypothetical protein, conserved [Entam...     327  4e-90
 ref|XP_001735958.1| hypothetical protein, conserved [Entam...     327  4e-90
 ref|XP_001733282.1| hypothetical protein, conserved [Entam...     326  1e-89
 ref|XP_001740867.1| hypothetical protein, conserved [Entam...     323  8e-89
 ref|XP_001737584.1| hypothetical protein, conserved [Entam...     318  3e-87
 ref|XP_001734308.1| hypothetical protein, conserved [Entam...     317  6e-87
 ref|XP_001735676.1| hypothetical protein, conserved [Entam...     316  9e-87
 ref|XP_001733551.1| hypothetical protein, conserved [Entam...     315  1e-86
 ref|XP_001737125.1| hypothetical protein EDI_212630 [Entam...     311  4e-85
 ref|XP_001740492.1| hypothetical protein, conserved [Entam...     309  8e-85
 ref|XP_001735195.1| hypothetical protein, conserved [Entam...     309  9e-85
 ref|XP_001740134.1| hypothetical protein, conserved [Entam...     306  8e-84
 ref|XP_001735921.1| hypothetical protein, conserved [Entam...     304  3e-83
 ref|XP_001733769.1| hypothetical protein, conserved [Entam...     303  6e-83
 ref|XP_001736642.1| hypothetical protein, conserved [Entam...     303  8e-83
 ref|XP_001738029.1| hypothetical protein, conserved [Entam...     303  8e-83
 ref|XP_001741179.1| hypothetical protein, conserved [Entam...     301  3e-82
 ref|XP_001741100.1| hypothetical protein, conserved [Entam...     298  2e-81
 ref|XP_001736971.1| hypothetical protein, conserved [Entam...     295  2e-80
 ref|XP_001737342.1| hypothetical protein, conserved [Entam...     293  1e-79
 ref|XP_001735627.1| hypothetical protein EDI_097000 [Entam...     293  1e-79
 ref|XP_001733937.1| hypothetical protein EDI_233880 [Entam...     293  1e-79
 ref|XP_001733760.1| hypothetical protein, conserved [Entam...     291  2e-79
 ref|XP_001734402.1| hypothetical protein, conserved [Entam...     291  3e-79
 ref|XP_001737531.1| hypothetical protein, conserved [Entam...     291  3e-79
 ref|XP_001738656.1| hypothetical protein, conserved [Entam...     287  4e-78
 ref|XP_001739665.1| hypothetical protein, conserved [Entam...     287  6e-78
 ref|XP_001737687.1| hypothetical protein, conserved [Entam...     286  1e-77
 ref|XP_001738179.1| hypothetical protein, conserved [Entam...     284  4e-77
 ref|XP_001739586.1| hypothetical protein, conserved [Entam...     283  1e-76
 ref|XP_001741619.1| hypothetical protein EDI_266780 [Entam...     282  1e-76
 ref|XP_001734453.1| hypothetical protein, conserved [Entam...     281  4e-76
 ref|XP_001740287.1| hypothetical protein, conserved [Entam...     279  9e-76
 ref|XP_001740560.1| hypothetical protein, conserved [Entam...     279  1e-75
 ref|XP_001740344.1| hypothetical protein, conserved [Entam...     279  1e-75
 ref|XP_001736447.1| hypothetical protein, conserved [Entam...     277  4e-75
 ref|XP_001735512.1| hypothetical protein, conserved [Entam...     277  5e-75
 ref|XP_001738659.1| hypothetical protein, conserved [Entam...     276  1e-74
 ref|XP_001741776.1| hypothetical protein EDI_255420 [Entam...     274  3e-74
 ref|XP_001739293.1| hypothetical protein, conserved [Entam...     272  2e-73
 ref|XP_001738090.1| hypothetical protein, conserved [Entam...     270  7e-73
 ref|XP_001733625.1| hypothetical protein, conserved [Entam...     268  2e-72
 ref|XP_001740398.1| hypothetical protein, conserved [Entam...     268  3e-72
 ref|XP_001734836.1| hypothetical protein EDI_122800 [Entam...     263  6e-71
 ref|XP_001741176.1| hypothetical protein EDI_242480 [Entam...     263  1e-70
 ref|XP_001737131.1| hypothetical protein EDI_231200 [Entam...     262  1e-70
 ref|XP_001740471.1| hypothetical protein, conserved [Entam...     261  5e-70
 ref|XP_001735296.1| hypothetical protein EDI_193970 [Entam...     260  5e-70
 ref|XP_001734405.1| hypothetical protein EDI_205930 [Entam...     260  8e-70
 ref|XP_001737480.1| hypothetical protein EDI_100620 [Entam...     257  3e-69
 ref|XP_001741680.1| hypothetical protein, conserved [Entam...     257  4e-69
 ref|XP_001735663.1| hypothetical protein, conserved [Entam...     256  1e-68
 ref|XP_001739955.1| hypothetical protein, conserved [Entam...     256  1e-68
 ref|XP_001736102.1| hypothetical protein, conserved [Entam...     252  2e-67
 ref|XP_001738450.1| hypothetical protein, conserved [Entam...     251  4e-67
 ref|XP_001735202.1| hypothetical protein EDI_098120 [Entam...     249  2e-66
 ref|XP_001740357.1| hypothetical protein, conserved [Entam...     247  4e-66
 ref|XP_001734692.1| hypothetical protein EDI_127240 [Entam...     246  2e-65
 ref|XP_001734950.1| hypothetical protein EDI_038580 [Entam...     245  2e-65
 ref|XP_001735640.1| hypothetical protein EDI_259030 [Entam...     244  3e-65
 ref|XP_001738078.1| hypothetical protein, conserved [Entam...     244  5e-65
 ref|XP_001734574.1| hypothetical protein, conserved [Entam...     244  6e-65
 ref|XP_001741934.1| hypothetical protein EDI_084580 [Entam...     243  6e-65
 ref|XP_001737864.1| hypothetical protein, conserved [Entam...     243  6e-65
 ref|XP_001741219.1| hypothetical protein EDI_304860 [Entam...     243  7e-65
 ref|XP_001741226.1| hypothetical protein, conserved [Entam...     241  3e-64
 ref|XP_001737874.1| hypothetical protein, conserved [Entam...     241  3e-64
 ref|XP_001735201.1| hypothetical protein, conserved [Entam...     240  8e-64
 ref|XP_001740148.1| hypothetical protein EDI_026470 [Entam...     239  1e-63
 ref|XP_001737569.1| hypothetical protein EDI_314150 [Entam...     236  9e-63
 ref|XP_001740402.1| hypothetical protein, conserved [Entam...     236  1e-62
 ref|XP_001737387.1| hypothetical protein, conserved [Entam...     235  2e-62
 ref|XP_001733810.1| hypothetical protein, conserved [Entam...     235  3e-62
 ref|XP_001735072.1| hypothetical protein EDI_342150 [Entam...     235  3e-62
 ref|XP_001741007.1| hypothetical protein EDI_090620 [Entam...     234  4e-62
 ref|XP_001734705.1| hypothetical protein, conserved [Entam...     232  2e-61
 ref|XP_001740980.1| hypothetical protein, conserved [Entam...     231  3e-61
 ref|XP_001741344.1| hypothetical protein, conserved [Entam...     231  4e-61
 ref|XP_001737239.1| hypothetical protein, conserved [Entam...     231  5e-61
 ref|XP_001738653.1| hypothetical protein, conserved [Entam...     227  6e-60
 ref|XP_001735524.1| hypothetical protein EDI_079470 [Entam...     227  7e-60
 ref|XP_001735836.1| hypothetical protein EDI_215510 [Entam...     225  2e-59
 ref|XP_001736644.1| hypothetical protein, conserved [Entam...     225  2e-59
 ref|XP_001733869.1| hypothetical protein, conserved [Entam...     224  4e-59
 ref|XP_001740408.1| hypothetical protein, conserved [Entam...     224  5e-59
 ref|XP_001737629.1| hypothetical protein EDI_109220 [Entam...     222  1e-58
 ref|XP_001738629.1| hypothetical protein, conserved [Entam...     219  2e-57
 ref|XP_001734793.1| hypothetical protein, conserved [Entam...     218  3e-57
 ref|XP_001741326.1| hypothetical protein EDI_312110 [Entam...     217  4e-57
 ref|XP_001738868.1| hypothetical protein, conserved [Entam...     217  7e-57
 ref|XP_001741938.1| hypothetical protein, conserved [Entam...     215  2e-56
 ref|XP_001740932.1| hypothetical protein, conserved [Entam...     212  3e-55
 ref|XP_001741570.1| hypothetical protein EDI_272700 [Entam...     211  3e-55
 ref|XP_001740298.1| hypothetical protein EDI_227850 [Entam...     211  5e-55
 ref|XP_001735910.1| hypothetical protein, conserved [Entam...     210  8e-55
 ref|XP_001736006.1| hypothetical protein, conserved [Entam...     210  9e-55
 ref|XP_001738462.1| hypothetical protein EDI_294620 [Entam...     209  2e-54
 ref|XP_001737106.1| hypothetical protein EDI_295610 [Entam...     208  3e-54
 ref|XP_001741436.1| hypothetical protein, conserved [Entam...     207  5e-54
 ref|XP_001735781.1| hypothetical protein EDI_304210 [Entam...     207  7e-54
 ref|XP_001735360.1| hypothetical protein, conserved [Entam...     205  2e-53
 ref|XP_001734173.1| hypothetical protein, conserved [Entam...     205  3e-53
 ref|XP_001738875.1| hypothetical protein, conserved [Entam...     205  3e-53
 ref|XP_001740387.1| hypothetical protein, conserved [Entam...     204  6e-53
 ref|XP_001734401.1| hypothetical protein, conserved [Entam...     204  7e-53
 ref|XP_001733494.1| hypothetical protein EDI_006530 [Entam...     203  8e-53
 ref|XP_001734399.1| hypothetical protein EDI_014330 [Entam...     203  9e-53
 ref|XP_001737692.1| hypothetical protein, conserved [Entam...     203  1e-52
 ref|XP_001735811.1| hypothetical protein, conserved [Entam...     202  3e-52
 ref|XP_001738451.1| hypothetical protein EDI_148970 [Entam...     199  2e-51
 ref|XP_001741861.1| hypothetical protein, conserved [Entam...     199  2e-51
 ref|XP_001737985.1| hypothetical protein, conserved [Entam...     198  3e-51
 ref|XP_001734298.1| hypothetical protein EDI_098430 [Entam...     192  3e-49
 ref|XP_001741615.1| hypothetical protein, conserved [Entam...     192  3e-49
 ref|XP_001740782.1| hypothetical protein, conserved [Entam...     190  5e-49
 ref|XP_001738317.1| hypothetical protein, conserved [Entam...     190  1e-48
 ref|XP_001735207.1| hypothetical protein, conserved [Entam...     189  1e-48
 ref|XP_001734310.1| hypothetical protein EDI_124440 [Entam...     189  1e-48
 ref|XP_001740667.1| hypothetical protein, conserved [Entam...     189  2e-48
 ref|XP_001734334.1| hypothetical protein EDI_142180 [Entam...     189  2e-48
 ref|XP_001735157.1| hypothetical protein, conserved [Entam...     185  3e-47
 ref|XP_001737118.1| hypothetical protein EDI_285200 [Entam...     182  2e-46
 ref|XP_001737686.1| hypothetical protein, conserved [Entam...     181  3e-46
 ref|XP_001740736.1| hypothetical protein, conserved [Entam...     181  3e-46
 ref|XP_001737275.1| hypothetical protein, conserved [Entam...     180  5e-46
 ref|XP_001736492.1| hypothetical protein, conserved [Entam...     180  6e-46
 ref|XP_001741327.1| hypothetical protein EDI_312120 [Entam...     179  1e-45
 ref|XP_001738968.1| hypothetical protein, conserved [Entam...     179  2e-45
 ref|XP_001738044.1| hypothetical protein, conserved [Entam...     179  2e-45
 ref|XP_001735912.1| hypothetical protein, conserved [Entam...     178  3e-45
 ref|XP_001734808.1| hypothetical protein, conserved [Entam...     178  3e-45
 ref|XP_001735896.1| hypothetical protein, conserved [Entam...     177  5e-45
 ref|XP_001741961.1| hypothetical protein, conserved [Entam...     177  6e-45
 ref|XP_001737719.1| hypothetical protein, conserved [Entam...     177  7e-45
 ref|XP_001738657.1| hypothetical protein EDI_329100 [Entam...     177  8e-45
 ref|XP_001733649.1| hypothetical protein, conserved [Entam...     175  2e-44
 ref|XP_001736612.1| hypothetical protein, conserved [Entam...     174  4e-44
 ref|XP_001735994.1| hypothetical protein, conserved [Entam...     174  5e-44
 ref|XP_001734591.1| hypothetical protein, conserved [Entam...     174  5e-44
 ref|XP_001735845.1| hypothetical protein, conserved [Entam...     174  5e-44
 ref|XP_001734375.1| hypothetical protein, conserved [Entam...     173  1e-43
 ref|XP_001736600.1| hypothetical protein, conserved [Entam...     170  6e-43
 ref|XP_001737142.1| hypothetical protein, conserved [Entam...     170  8e-43
 ref|XP_001736556.1| hypothetical protein, conserved [Entam...     169  1e-42
 ref|XP_001739242.1| hypothetical protein EDI_250790 [Entam...     169  2e-42
 ref|XP_001733888.1| hypothetical protein, conserved [Entam...     169  2e-42
 ref|XP_001741277.1| hypothetical protein, conserved [Entam...     169  2e-42
 ref|XP_001733629.1| hypothetical protein, conserved [Entam...     168  3e-42
 ref|XP_001734275.1| hypothetical protein EDI_234080 [Entam...     167  7e-42
 ref|XP_001739661.1| hypothetical protein, conserved [Entam...     167  7e-42
 ref|XP_001734009.1| hypothetical protein, conserved [Entam...     165  3e-41
 ref|XP_001737385.1| hypothetical protein, conserved [Entam...     165  3e-41
 ref|XP_001737214.1| hypothetical protein, conserved [Entam...     165  3e-41
 ref|XP_001738641.1| hypothetical protein, conserved [Entam...     164  5e-41
 ref|XP_001733579.1| hypothetical protein, conserved [Entam...     164  6e-41
 ref|XP_001740868.1| hypothetical protein, conserved [Entam...     164  7e-41
 ref|XP_001733599.1| hypothetical protein, conserved [Entam...     164  7e-41
 ref|XP_001739680.1| hypothetical protein EDI_039980 [Entam...     162  2e-40
 ref|XP_001738391.1| hypothetical protein, conserved [Entam...     161  5e-40
 ref|XP_001736975.1| hypothetical protein, conserved [Entam...     158  4e-39
 ref|XP_001742001.1| hypothetical protein, conserved [Entam...     157  7e-39
 ref|XP_001740309.1| hypothetical protein, conserved [Entam...     157  8e-39
 ref|XP_001741878.1| hypothetical protein EDI_181820 [Entam...     156  1e-38
 ref|XP_001734804.1| hypothetical protein, conserved [Entam...     155  3e-38
 ref|XP_001736822.1| hypothetical protein, conserved [Entam...     153  7e-38
 ref|XP_001740566.1| hypothetical protein, conserved [Entam...     153  9e-38
 ref|XP_001740940.1| hypothetical protein, conserved [Entam...     152  2e-37
 ref|XP_001738338.1| hypothetical protein, conserved [Entam...     152  2e-37
 ref|XP_001733693.1| hypothetical protein, conserved [Entam...     151  4e-37
 ref|XP_001735772.1| hypothetical protein, conserved [Entam...     151  5e-37
 ref|XP_001734852.1| hypothetical protein, conserved [Entam...     147  5e-36
 ref|XP_001740200.1| hypothetical protein EDI_139870 [Entam...     143  8e-35
 ref|XP_001739017.1| hypothetical protein, conserved [Entam...     143  1e-34
 ref|XP_001738072.1| hypothetical protein, conserved [Entam...     141  5e-34
 ref|XP_001739121.1| hypothetical protein, conserved [Entam...     139  2e-33
 ref|XP_001738461.1| hypothetical protein, conserved [Entam...     138  2e-33
 ref|XP_001739251.1| hypothetical protein EDI_043720 [Entam...     138  3e-33
 ref|XP_001735410.1| hypothetical protein, conserved [Entam...     138  4e-33
 ref|XP_001735302.1| hypothetical protein, conserved [Entam...     137  1e-32
 ref|XP_001738456.1| hypothetical protein, conserved [Entam...     136  1e-32
 ref|XP_001736823.1| hypothetical protein, conserved [Entam...     136  1e-32
 ref|XP_001738460.1| hypothetical protein EDI_104120 [Entam...     136  2e-32
 ref|XP_001737783.1| hypothetical protein, conserved [Entam...     135  3e-32
 ref|XP_001738214.1| hypothetical protein EDI_126740 [Entam...     135  3e-32
 ref|XP_001734812.1| hypothetical protein, conserved [Entam...     135  4e-32
 ref|XP_001739733.1| hypothetical protein, conserved [Entam...     133  1e-31
 ref|XP_001734593.1| hypothetical protein, conserved [Entam...     133  1e-31
 ref|XP_001739241.1| hypothetical protein EDI_257860 [Entam...     131  4e-31
 ref|XP_001740464.1| hypothetical protein, conserved [Entam...     130  8e-31
 ref|XP_001739734.1| hypothetical protein, conserved [Entam...     130  1e-30
 ref|XP_001740979.1| hypothetical protein, conserved [Entam...     129  2e-30
 ref|XP_001738392.1| hypothetical protein, conserved [Entam...     129  2e-30
 ref|XP_001734509.1| hypothetical protein, conserved [Entam...     127  8e-30
 ref|XP_001738264.1| hypothetical protein, conserved [Entam...     126  1e-29
 ref|XP_001741718.1| hypothetical protein EDI_098930 [Entam...     123  1e-28
 ref|XP_001741630.1| hypothetical protein, conserved [Entam...     123  1e-28
 ref|XP_001738458.1| hypothetical protein, conserved [Entam...     121  6e-28
 ref|XP_001735427.1| hypothetical protein EDI_267850 [Entam...     117  6e-27
 ref|XP_001741957.1| hypothetical protein, conserved [Entam...     117  1e-26
 ref|XP_001734980.1| hypothetical protein, conserved [Entam...     115  3e-26
 ref|XP_001741330.1| hypothetical protein, conserved [Entam...     115  3e-26
 ref|XP_001738549.1| hypothetical protein, conserved [Entam...     114  6e-26
 ref|XP_001737116.1| hypothetical protein, conserved [Entam...     112  3e-25
 ref|XP_001740092.1| hypothetical protein, conserved [Entam...     112  3e-25
 ref|XP_001735712.1| hypothetical protein, conserved [Entam...     109  2e-24
 ref|XP_001740087.1| hypothetical protein, conserved [Entam...     108  2e-24
 ref|XP_001737308.1| hypothetical protein EDI_226110 [Entam...     108  3e-24
 ref|XP_001740203.1| hypothetical protein EDI_289220 [Entam...     107  7e-24
 ref|XP_001737311.1| hypothetical protein, conserved [Entam...     105  2e-23
 ref|XP_001734315.1| hypothetical protein, conserved [Entam...     105  3e-23
 ref|XP_001740590.1| hypothetical protein, conserved [Entam...     105  3e-23
 ref|XP_001737685.1| hypothetical protein EDI_331560 [Entam...     105  3e-23
 ref|XP_001740017.1| hypothetical protein, conserved [Entam...     105  3e-23
 ref|XP_001741027.1| hypothetical protein, conserved [Entam...     100  1e-21
 ref|XP_001736777.1| hypothetical protein EDI_194810 [Entam...      99  2e-21
 ref|XP_001740956.1| hypothetical protein EDI_274790 [Entam...      97  7e-21
 ref|XP_001739803.1| hypothetical protein EDI_160990 [Entam...      96  2e-20
 ref|XP_001740508.1| hypothetical protein EDI_024880 [Entam...      94  5e-20
 ref|XP_001733909.1| hypothetical protein, conserved [Entam...      94  6e-20
 ref|XP_001734314.1| hypothetical protein EDI_269140 [Entam...      93  1e-19
 ref|XP_001738511.1| hypothetical protein, conserved [Entam...      91  3e-19
 ref|XP_001738455.1| hypothetical protein EDI_027280 [Entam...      90  8e-19
 ref|XP_001739243.1| hypothetical protein EDI_250800 [Entam...      90  8e-19
 ref|XP_001738455.1| hypothetical protein EDI_027280 [Entam...      85  5e-17
 ref|XP_001739243.1| hypothetical protein EDI_250800 [Entam...      85  5e-17
 ref|XP_001738455.1| hypothetical protein EDI_027280 [Entam...      83  1e-16
 ref|XP_001739243.1| hypothetical protein EDI_250800 [Entam...      83  1e-16
 ref|XP_001738455.1| hypothetical protein EDI_027280 [Entam...      81  4e-16
 ref|XP_001739243.1| hypothetical protein EDI_250800 [Entam...      81  4e-16
 ref|XP_001738455.1| hypothetical protein EDI_027280 [Entam...      80  8e-16
 ref|XP_001739243.1| hypothetical protein EDI_250800 [Entam...      80  8e-16
 ref|XP_001738455.1| hypothetical protein EDI_027280 [Entam...      78  6e-15
 ref|XP_001739243.1| hypothetical protein EDI_250800 [Entam...      78  6e-15
 ref|XP_001738455.1| hypothetical protein EDI_027280 [Entam...      71  6e-13
 ref|XP_001739243.1| hypothetical protein EDI_250800 [Entam...      71  6e-13
 ref|XP_001738455.1| hypothetical protein EDI_027280 [Entam...      67  7e-12
 ref|XP_001739243.1| hypothetical protein EDI_250800 [Entam...      67  7e-12
 ref|XP_001738455.1| hypothetical protein EDI_027280 [Entam...      65  4e-11
 ref|XP_001739243.1| hypothetical protein EDI_250800 [Entam...      65  4e-11
 ref|XP_001738455.1| hypothetical protein EDI_027280 [Entam...      55  4e-08
 ref|XP_001739243.1| hypothetical protein EDI_250800 [Entam...      55  4e-08
 ref|XP_001738455.1| hypothetical protein EDI_027280 [Entam...      54  6e-08
 ref|XP_001739243.1| hypothetical protein EDI_250800 [Entam...      54  6e-08
 ref|XP_001739691.1| hypothetical protein EDI_159440 [Entam...      90  1e-18
 ref|XP_001735523.1| hypothetical protein EDI_079460 [Entam...      89  2e-18
 ref|XP_001739250.1| hypothetical protein, conserved [Entam...      89  2e-18
 ref|XP_001740706.1| hypothetical protein EDI_303900 [Entam...      83  1e-16
 ref|XP_001741467.1| hypothetical protein EDI_146410 [Entam...      83  1e-16
 ref|XP_001739419.1| hypothetical protein EDI_287860 [Entam...      82  2e-16
 ref|XP_001737119.1| hypothetical protein EDI_341480 [Entam...      80  2e-15
 ref|XP_001739075.1| hypothetical protein EDI_313320 [Entam...      78  3e-15
 ref|XP_001736172.1| hypothetical protein EDI_178160 [Entam...      77  8e-15
 ref|XP_001739074.1| hypothetical protein EDI_313310 [Entam...      76  1e-14
 ref|XP_001733492.1| hypothetical protein EDI_323110 [Entam...      76  2e-14
 ref|XP_001740728.1| hypothetical protein, conserved [Entam...      75  3e-14
 ref|XP_001734256.1| hypothetical protein, conserved [Entam...      75  3e-14
 ref|XP_001738096.1| hypothetical protein, conserved [Entam...      73  1e-13
 ref|XP_001741429.1| hypothetical protein EDI_280280 [Entam...      71  5e-13
 ref|XP_001737524.1| hypothetical protein EDI_311230 [Entam...      68  3e-12
 ref|XP_001741787.1| hypothetical protein EDI_127450 [Entam...      68  4e-12
 ref|XP_001737041.1| hypothetical protein, conserved [Entam...      67  9e-12
 ref|XP_001741022.1| hypothetical protein EDI_277450 [Entam...      66  2e-11
 ref|XP_001738470.1| hypothetical protein EDI_309650 [Entam...      62  3e-10
 ref|XP_001734257.1| hypothetical protein EDI_331760 [Entam...      60  1e-09
 ref|XP_001739939.1| hypothetical protein EDI_089870 [Entam...      57  8e-09
 ref|XP_001737107.1| hypothetical protein EDI_248650 [Entam...      55  4e-08
 ref|XP_001740266.1| hypothetical protein EDI_177500 [Entam...      54  5e-08
 ref|XP_001740090.1| hypothetical protein EDI_006520 [Entam...      54  5e-08
 ref|XP_001734794.1| hypothetical protein EDI_054010 [Entam...      53  1e-07
 ref|XP_001740988.1| hypothetical protein EDI_043320 [Entam...      47  7e-06

  Entamoeba histolytica HM-1:IMSS [eukaryotes] taxid 294381
 ref|XP_648136.1| leucine rich repeat protein, BspA family ...     361  2e-100
 ref|XP_001913792.1| leucine rich repeat protein 1 [Entamoe...     361  2e-100
 ref|XP_001914244.1| leucine rich repeat protein 1 [Entamoe...     361  2e-100
 ref|XP_001913792.1| leucine rich repeat protein 1 [Entamoe...     359  9e-100
 ref|XP_001914244.1| leucine rich repeat protein 1 [Entamoe...     359  9e-100
 ref|XP_001913792.1| leucine rich repeat protein 1 [Entamoe...     325  3e-89
 ref|XP_001914244.1| leucine rich repeat protein 1 [Entamoe...     325  3e-89
 ref|XP_001913792.1| leucine rich repeat protein 1 [Entamoe...     320  9e-88
 ref|XP_001914244.1| leucine rich repeat protein 1 [Entamoe...     320  9e-88
 ref|XP_654945.2| leucine rich repeat protein, BspA family ...     361  3e-100
 ref|XP_001914389.1| leucine rich repeat protein, BspA fami...     348  2e-96
 ref|XP_647950.1| leucine rich repeat protein, BspA family ...     339  1e-93
 ref|XP_648615.1| leucine rich repeat protein, BspA family ...     336  6e-93
 ref|XP_651468.1| leucine rich repeat protein, BspA family ...     323  6e-89
 ref|XP_001914509.1| leucine rich repeat protein, BspA fami...     312  2e-85
 ref|XP_651527.1| leucine rich repeat protein, BspA family ...     310  4e-85
 ref|XP_648067.2| leucine rich repeat protein, BspA family ...     307  4e-84
 ref|XP_648727.1| leucine rich repeat protein, BspA family ...     307  5e-84
 ref|XP_649640.1| leucine rich repeat protein, BspA family ...     304  2e-83
 ref|XP_652255.1| leucine rich repeat protein, BspA family ...     302  1e-82
 ref|XP_653526.1| leucine rich repeat protein, BspA family ...     302  1e-82
 ref|XP_657553.1| leucine rich repeat protein, BspA family ...     293  6e-80
 ref|XP_657437.1| hypothetical protein EHI_151330 [Entamoeb...     291  2e-79
 ref|XP_652986.2| leucine rich repeat protein, BspA family ...     281  3e-76
 ref|XP_656869.1| leucine rich repeat protein, BspA family ...     277  6e-75
 ref|XP_657020.2| leucine rich repeat protein, BspA family ...     277  7e-75
 ref|XP_647955.1| leucine rich repeat protein, BspA family ...     273  9e-74
 ref|XP_652730.1| leucine rich repeat protein, BspA family ...     267  5e-72
 ref|XP_649402.2| leucine rich repeat protein, BspA family ...     265  2e-71
 ref|XP_653909.2| leucine rich repeat protein, BspA family ...     262  1e-70
 ref|XP_651218.1| leucine rich repeat protein, BspA family ...     259  9e-70
 ref|XP_648118.2| leucine rich repeat protein, BspA family ...     259  1e-69
 ref|XP_651312.1| leucine rich repeat protein, BspA family ...     259  1e-69
 ref|XP_652531.2| leucine rich repeat protein, BspA family ...     259  2e-69
 ref|XP_654256.1| leucine rich repeat protein, BspA family ...     257  3e-69
 ref|XP_650302.1| leucine rich repeat protein, BspA family ...     255  2e-68
 ref|XP_651425.1| leucine rich repeat protein, BspA family ...     252  1e-67
 ref|XP_651979.1| leucine rich repeat protein, BspA family ...     249  2e-66
 ref|XP_650788.1| leucine rich repeat protein, BspA family ...     244  4e-65
 ref|XP_649473.2| leucine rich repeat protein, BspA family ...     243  7e-65
 ref|XP_653176.1| leucine rich repeat protein, BspA family ...     237  6e-63
 ref|XP_652287.1| leucine rich repeat protein, BspA family ...     236  1e-62
 ref|XP_651302.1| leucine rich repeat protein, BspA family ...     233  1e-61
 ref|XP_655312.1| leucine rich repeat protein, BspA family ...     229  1e-60
 ref|XP_653754.1| leucine rich repeat protein, BspA family ...     228  3e-60
 ref|XP_648877.1| leucine rich repeat protein, BspA family ...     227  4e-60
 ref|XP_652183.1| leucine rich repeat protein, BspA family ...     221  3e-58
 ref|XP_648144.1| leucine rich repeat protein, BspA family ...     221  3e-58
 ref|XP_650599.2| leucine rich repeat protein, BspA family ...     221  5e-58
 ref|XP_001914387.1| leucine rich repeat protein, BspA fami...     219  2e-57
 ref|XP_649307.2| leucine rich repeat protein, BspA family ...     217  6e-57
 ref|XP_657493.2| leucine rich repeat protein, BspA family ...     214  6e-56
 ref|XP_648456.2| hypothetical protein EHI_098720 [Entamoeb...     206  1e-53
 ref|XP_651604.1| leucine rich repeat protein, BspA family ...     204  6e-53
 ref|XP_650208.1| leucine rich repeat protein, BspA family ...     198  3e-51
 ref|XP_657486.2| leucine rich repeat protein, BspA family ...     196  1e-50
 ref|XP_653459.2| leucine rich repeat protein, BspA family ...     196  1e-50
 ref|XP_654274.1| leucine rich repeat protein, BspA family ...     195  2e-50
 ref|XP_654709.1| leucine rich repeat protein, BspA family ...     192  2e-49
 ref|XP_648169.1| leucine rich repeat protein, BspA family ...     187  8e-48
 ref|XP_655176.2| hypothetical protein EHI_198630 [Entamoeb...     184  7e-47
 ref|XP_001913739.1| hypothetical protein EHI_079970 [Entam...     183  1e-46
 ref|XP_648581.1| leucine rich repeat protein, BspA family ...     179  2e-45
 ref|XP_001913709.1| leucine rich repeat protein [Entamoeba...     174  6e-44
 ref|XP_649324.1| leucine rich repeat protein, BspA family ...     174  7e-44
 ref|XP_652773.1| leucine rich repeat protein, BspA family ...     172  1e-43
 ref|XP_657078.1| leucine rich repeat protein, BspA family ...     172  2e-43
 ref|XP_648009.1| leucine rich repeat protein, BspA family ...     171  4e-43
 ref|XP_648467.2| leucine rich repeat protein, BspA family ...     169  1e-42
 ref|XP_651410.1| leucine rich repeat protein, BspA family ...     167  5e-42
 ref|XP_001914495.1| leucine rich repeat protein, BspA fami...     167  7e-42
 ref|XP_648354.1| leucine rich repeat protein, BspA family ...     166  1e-41
 ref|XP_001914364.1| leucine rich repeat protein, BspA fami...     166  2e-41
 ref|XP_001914312.1| leucine rich repeat protein, BspA fami...     165  2e-41
 ref|XP_654033.1| leucine rich repeat protein, BspA family ...     165  3e-41
 ref|XP_648029.2| leucine rich repeat protein, BspA family ...     162  2e-40
 ref|XP_651308.1| leucine rich repeat protein, BspA family ...     161  5e-40
 ref|XP_649377.1| leucine rich repeat protein, BspA family ...     159  1e-39
 ref|XP_001914377.1| leucine rich repeat protein, BspA fami...     156  1e-38
 ref|XP_654062.1| leucine rich repeat protein, BspA family ...     153  7e-38
 ref|XP_655795.2| hypothetical protein EHI_151590 [Entamoeb...     151  4e-37
 ref|XP_651509.1| leucine rich repeat protein, BspA family ...     150  7e-37
 ref|XP_650237.1| leucine rich repeat protein, BspA family ...     149  2e-36
 ref|XP_001914533.1| leucine rich repeat protein, BspA fami...     140  1e-33
 ref|XP_651133.2| leucine rich repeat protein, BspA family ...     139  2e-33
 ref|XP_651914.1| leucine rich repeat protein, BspA family ...     139  2e-33
 ref|XP_655705.1| leucine rich repeat protein, BspA family ...     138  3e-33
 ref|XP_001914612.1| leucine rich repeat protein, BspA fami...     137  6e-33
 ref|XP_650551.2| leucine rich repeat protein, BspA family ...     137  6e-33
 ref|XP_648832.1| leucine rich repeat protein, BspA family ...     137  7e-33
 ref|XP_651813.1| leucine rich repeat protein, BspA family ...     135  2e-32
 ref|XP_001914433.1| leucine rich repeat protein [Entamoeba...     133  1e-31
 ref|XP_651898.1| leucine rich repeat protein, BspA family ...     130  1e-30
 ref|XP_001914401.1| leucine rich repeat protein, BspA fami...     128  3e-30
 ref|XP_648823.1| leucine rich repeat protein, BspA family ...     115  2e-26
 ref|XP_648451.1| hypothetical protein EHI_111960 [Entamoeb...     111  5e-25
 ref|XP_649048.1| leucine rich repeat protein, BspA family ...     111  5e-25
 ref|XP_650847.2| leucine rich repeat protein, BspA family ...     110  1e-24
 ref|XP_001914146.1| leucine rich repeat protein, BspA fami...     108  3e-24
 ref|XP_650434.2| hypothetical protein EHI_154170 [Entamoeb...     108  4e-24
 ref|XP_001914421.1| leucine rich repeat protein, BspA fami...     106  2e-23
 ref|XP_001914462.1| leucine rich repeat protein, BspA fami...     105  3e-23
 ref|XP_649281.2| leucine rich repeat protein, BspA family ...     105  4e-23
 ref|XP_649001.1| leucine rich repeat protein, BspA family ...     103  1e-22
 ref|XP_656199.1| hypothetical protein EHI_012090 [Entamoeb...     101  3e-22
 ref|XP_653894.2| leucine rich repeat protein, BspA family ...      95  3e-20
 ref|XP_001913872.1| leucine rich repeat protein, BspA fami...      91  3e-19
 ref|XP_655019.1| hypothetical protein EHI_084160 [Entamoeb...      83  1e-16
 ref|XP_651875.1| leucine rich repeat protein, BspA family ...      78  3e-15
 ref|XP_001914505.1| hypothetical protein EHI_102700 [Entam...      76  1e-14
 ref|XP_001914228.1| hypothetical protein EHI_154590 [Entam...      76  1e-14
 ref|XP_649553.1| leucine rich repeat protein, BspA family ...      73  1e-13
 ref|XP_001914430.1| leucine rich repeat protein, BspA fami...      72  3e-13
 ref|XP_657303.1| leucine-rich repeat containing protein [E...      71  6e-13
 ref|XP_001914519.1| hypothetical protein EHI_161300 [Entam...      70  1e-12
 ref|XP_647912.1| hypothetical protein EHI_113310 [Entamoeb...      67  7e-12
 ref|XP_001914342.1| leucine rich repeat protein, BspA fami...      65  3e-11
 ref|XP_649499.1| leucine-rich repeat containing protein [E...      65  4e-11
 ref|XP_001914356.1| hypothetical protein EHI_046800 [Entam...      63  1e-10
 ref|XP_654924.1| leucine-rich repeat containing protein [E...      60  1e-09
 ref|XP_655005.1| hypothetical protein, conserved [Entamoeb...      58  3e-09
 ref|XP_649029.1| hypothetical protein EHI_186220 [Entamoeb...      56  1e-08
 ref|XP_651994.2| protein kinase domain containing protein ...      53  9e-08
 ref|XP_001914178.1| hypothetical protein EHI_028330 [Entam...      47  9e-06
```

---

**Taxonomy Report**

```
Entamoeba .........................  3143 hits    2 orgs [root; cellular organisms; Eukaryota; Amoebozoa; Archamoebae; Entamoebidae]
. Entamoeba dispar SAW760 .........  2213 hits    1 orgs [Entamoeba dispar]
. Entamoeba histolytica HM-1:IMSS .   930 hits    1 orgs [Entamoeba histolytica]
```
