# Supplementary material for: Trichomonas vaginalis vast BspA-like gene family: evidence for functional diversity from structural organisation and transcriptomics
Source: BMC Genomics. 2010 Feb 8;11:99. doi: 10.1186/1471-2164-11-99 (PMC2843621; doi:10.1186/1471-2164-11-99)
Supplement: Additional file 5 — Supplemental Table S5. Example of T. vaginalis proteins with LRR distinct from TpLRR. Table listing the accession numbers of selected T. vaginalis proteins with LRR distinct from the TpLRR and taxa encoding related proteins. [file 1471-2164-11-99-S5.PDF]

Table S5. Example of *T. vaginalis* proteins with LRR distinct from TvBspA TpLRR.

RefSeq accession	*T. vaginalis* related proteins	BlastP taxa hits (top hits only)[a]	Feature of *T. vaginalis* protein(s) including RPS-Blast top hit
XP_001306799	23	*Paramecium tetraurelia*<br>*Tetrahymena thermophila*<br>*Leishmania* sp (3)<br>and other eukaryotes	Positive for the profile cd00116: LRR_RI, ribonuclease inhibitor-like subfamily, potentially intracellular
XP_001310250	14	*Leishmania* sp. (3)<br>*Trypanosoma* sp. (2)	Potentially intracellular, no identified profile or domain
XP_001313858	---	*Dictyostelium discoideum*<br>*Clostridium botulinum*<br>other eukaryotes	Candidate membrane protein with 9 TMDs positive for profile COG4886: Leucine-rich repeat protein
XP_001318595<br>XP_001582816	2 (entry below)	*Trichomonas* specific	Candidate membrane proteins with 7 TMDs<br>Candidate membrane proteins with 8 TMDs Positive for profile cl02423: Leucine-rich repeats, ribonuclease inhibitor-like subfamily

[a]BlastP search e-value ≤1e^{-5} against RefSeq and not *T. vaginalis*
