# Supplementary material for: Trichomonas vaginalis vast BspA-like gene family: evidence for functional diversity from structural organisation and transcriptomics
Source: BMC Genomics. 2010 Feb 8;11:99. doi: 10.1186/1471-2164-11-99 (PMC2843621; doi:10.1186/1471-2164-11-99)
Supplement: Additional file 8 — Supplemental Figure S3. Overview of scaffold with 18 TvBspA genes. Figure illustrating the gene content of contig DS113361 and highlighting the positions of 18 TvBspA genes. [file 1471-2164-11-99-S8.PDF]

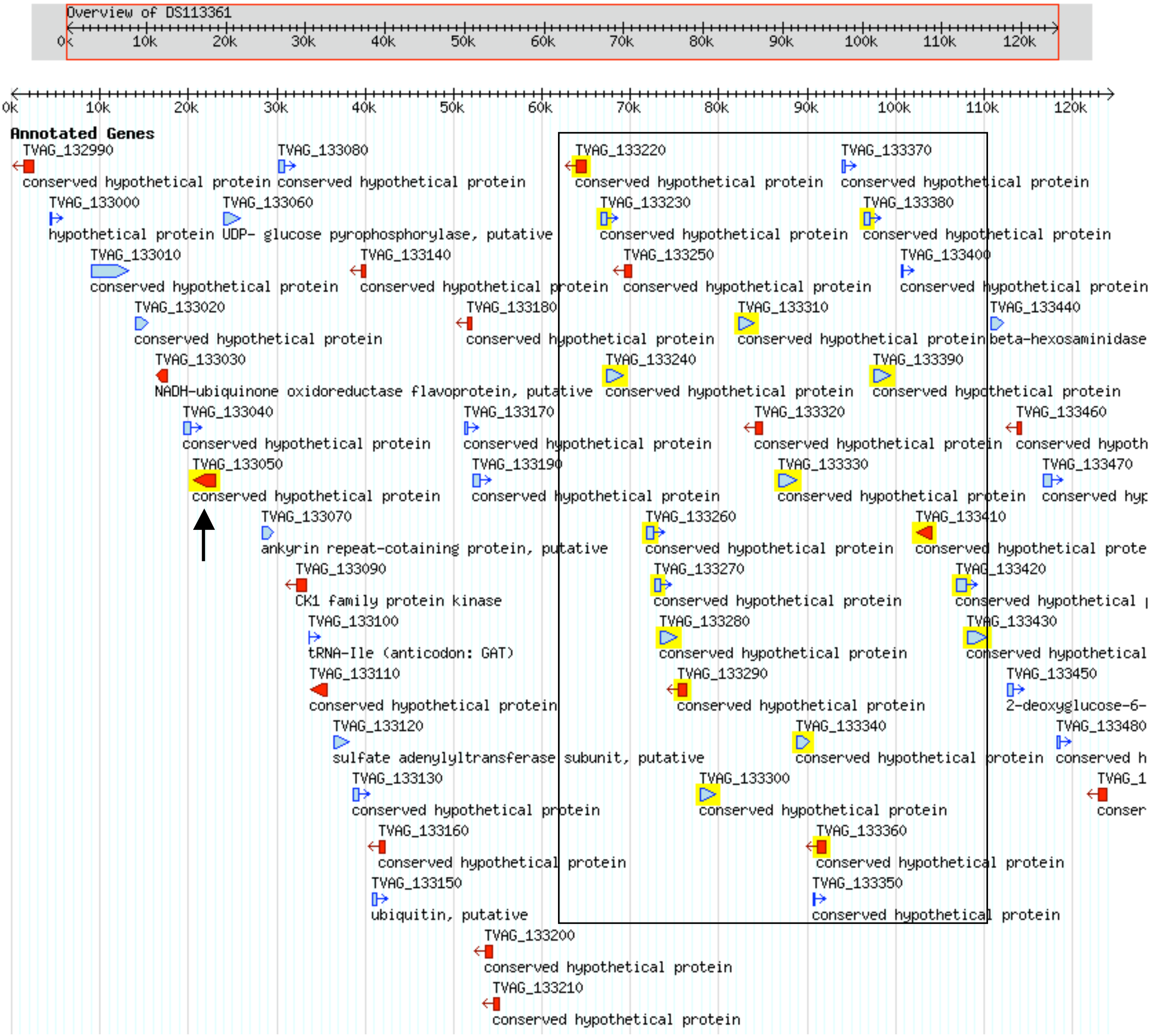

Figure S3. Scaffold encoding 18 TvBspA candidate proteins.

The scaffold DS113361 is 124.7 kbp long and encodes a total of 18 candidate TvBspA proteins (yellow highlight). The entire length of the scaffold is shown and the graphical representation was obtained from TrichDB. 17 TvBspA are clustered (boxed) towards the 3’end of the scaffold and are intermingled with five non-BspA encoding genes. One TvBspA ORF is located within the first 25 kbp of the scaffold (vertical arrow - TVAG_133050). CLUSS2 recovered the 17 clustered protein sequences (boxed) into three subfamilies (#200, #228 and #275), and the global Clustal alignment (with all 911 TvBspA) recovered the 17 proteins close to each other (Table 2; additional file 1, Table S1 and text) indicating that they are more closely related to each other within each subfamily then to other TvBspA. The TVAG_133050 entry is part of subfamily #22 made of a total of 14 TvBspA entries encoded by different scaffolds.
