# Supplementary material for: Trichomonas vaginalis vast BspA-like gene family: evidence for functional diversity from structural organisation and transcriptomics
Source: BMC Genomics. 2010 Feb 8;11:99. doi: 10.1186/1471-2164-11-99 (PMC2843621; doi:10.1186/1471-2164-11-99)
Supplement: Additional file 9 — Supplemental Table S6.PHI-/PSI-Blast taxonomic report for Bacteria and Achaea. Full taxonomic report of PHI/PSI-BlastP searches on NCBI RefSeq protein database. In html format to be open in a web browser. [file 1471-2164-11-99-S9.HTML]

### Table S6. Taxonomic report for PSI-BLAST search on Archaea and Bacteria at RefSeq

---


### Profile (PSSM): based on PHI-Blast (see Table 1 and Table S2), two iterations

### e-value <= 0.00001

---


### 1) Bacteria

**Index**

- Lineage Report
- Organism Report
- Taxonomy Report
- Help

### 2) Archaea

**Index**

- Lineage Report
- Organism Report
- Taxonomy Report
- Help

---


### 1) Bacteria

**Lineage Report**  

```
Bacteria       [bacteria]
. Firmicutes     [firmicutes]
. . Clostridiales  [firmicutes]
. . . Clostridiaceae [firmicutes]
. . . . Clostridium    [firmicutes]
. . . . . Clostridium leptum DSM 753 -------------------------  472   5 hits [firmicutes]          hypothetical protein CLOLEP_03013 [Clostridium leptum DSM 7
. . . . . Clostridium sp. L2-50 ..............................  264  77 hits [firmicutes]          hypothetical protein CLOL250_02668 [Clostridium sp. L2-50]
. . . . . Clostridium phytofermentans ISDg ...................  253  41 hits [firmicutes]          Ig domain-containing protein [Clostridium phytofermentans I
. . . . . Clostridium beijerinckii NCIMB 8052 ................  249  55 hits [firmicutes]          cell wall binding repeat-containing protein [Clostridium be
. . . . . Clostridium scindens ATCC 35704 ....................  231  26 hits [firmicutes]          hypothetical protein CLOSCI_03447 [Clostridium scindens ATC
. . . . . Clostridium sporogenes ATCC 15579 ..................  230  15 hits [firmicutes]          hypothetical protein CLOSPO_00560 [Clostridium sporogenes A
. . . . . Clostridium botulinum F str. Langeland .............  223  18 hits [firmicutes]          putative cell surface protein [Clostridium botulinum F str.
. . . . . Clostridium butyricum 5521 .........................  220  14 hits [firmicutes]          surface protein PspC [Clostridium butyricum 5521]
. . . . . Clostridium bartlettii DSM 16795 ...................  202  28 hits [firmicutes]          hypothetical protein CLOBAR_00617 [Clostridium bartlettii D
. . . . . Clostridium botulinum A3 str. Loch Maree ...........  177  16 hits [firmicutes]          putative cell surface protein [Clostridium botulinum A3 str
. . . . . Clostridium botulinum A str. ATCC 3502 .............  174  18 hits [firmicutes]          cell surface protein [Clostridium botulinum A str. ATCC 350
. . . . . Clostridium botulinum NCTC 2916 ....................  172  17 hits [firmicutes]          putative cell surface protein [Clostridium botulinum NCTC 2
. . . . . Clostridium botulinum B1 str. Okra .................  170  16 hits [firmicutes]          putative cell surface protein [Clostridium botulinum B1 str
. . . . . Clostridium botulinum Bf ...........................  169  18 hits [firmicutes]          putative cell surface protein [Clostridium botulinum Bf]
. . . . . Clostridium sp. SS2/1 ..............................  134  52 hits [firmicutes]          hypothetical protein CLOSS21_02635 [Clostridium sp. SS2/1]
. . . . . Clostridium thermocellum ATCC 27405 ................  126   3 hits [firmicutes]          cellulosome enzyme, dockerin type I [Clostridium thermocell
. . . . . Clostridium thermocellum DSM 4150 ..................  118   3 hits [firmicutes]          cellulosome protein dockerin type I [Clostridium thermocell
. . . . . Clostridium novyi NT ...............................   71   3 hits [firmicutes]          hypothetical protein NT01CX_1459 [Clostridium novyi NT]
. . . . . Clostridium bolteae ATCC BAA-613 ...................   56   2 hits [firmicutes]          hypothetical protein CLOBOL_00037 [Clostridium bolteae ATCC
. . . . Alkaliphilus metalliredigens QYMF --------------------   81   8 hits [firmicutes]          cell wall/surface repeat-containing protein [Alkaliphilus m
. . . Eubacterium siraeum DSM 15702 --------------------------  426  48 hits [firmicutes]          hypothetical protein EUBSIR_02697 [Eubacterium siraeum DSM 
. . . Epulopiscium sp. 'N.t. morphotype B' ...................  355 189 hits [firmicutes]          cell surface protein [Epulopiscium sp. 'N.t. morphotype B']
. . . Anaerofustis stercorihominis DSM 17244 .................  343  91 hits [firmicutes]          hypothetical protein ANASTE_00419 [Anaerofustis stercorihom
. . . Syntrophomonas wolfei subsp. wolfei str. Goettingen ....  321   4 hits [firmicutes]          leucine-rich repeat-containing protein [Syntrophomonas wolf
. . . Ruminococcus torques ATCC 27756 ........................  310  43 hits [firmicutes]          hypothetical protein RUMTOR_02627 [Ruminococcus torques ATC
. . . Coprococcus eutactus ATCC 27759 ........................  305  36 hits [firmicutes]          hypothetical protein COPEUT_00473 [Coprococcus eutactus ATC
. . . Desulfitobacterium hafniense Y51 .......................  256  12 hits [firmicutes]          hypothetical protein DSY3856 [Desulfitobacterium hafniense 
. . . Eubacterium ventriosum ATCC 27560 ......................  228  75 hits [firmicutes]          hypothetical protein EUBVEN_02455 [Eubacterium ventriosum A
. . . Dorea formicigenerans ATCC 27755 .......................  224   8 hits [firmicutes]          hypothetical protein DORFOR_02330 [Dorea formicigenerans AT
. . . Ruminococcus obeum ATCC 29174 ..........................  182  35 hits [firmicutes]          hypothetical protein RUMOBE_01418 [Ruminococcus obeum ATCC 
. . . Finegoldia magna ATCC 29328 ............................  180  12 hits [firmicutes]          putative N-acetylmuramoyl-L-alanine amidase [Finegoldia mag
. . . Ruminococcus gnavus ATCC 29149 .........................  111  23 hits [firmicutes]          hypothetical protein RUMGNA_01106 [Ruminococcus gnavus ATCC
. . . Anaerostipes caccae DSM 14662 ..........................  102  29 hits [firmicutes]          hypothetical protein ANACAC_01224 [Anaerostipes caccae DSM 
. . . Faecalibacterium prausnitzii M21/2 .....................   90  12 hits [firmicutes]          hypothetical protein FAEPRAM212_02550 [Faecalibacterium pra
. . . Anaerotruncus colihominis DSM 17241 ....................   84  11 hits [firmicutes]          hypothetical protein ANACOL_02805 [Anaerotruncus colihomini
. . . Desulfotomaculum reducens MI-1 .........................   62   3 hits [firmicutes]          hypothetical protein Dred_1389 [Desulfotomaculum reducens M
. . . Dorea longicatena DSM 13814 ............................   58   4 hits [firmicutes]          hypothetical protein DORLON_01692 [Dorea longicatena DSM 13
. . Clostridium spiroforme DSM 1552 --------------------------  379   5 hits [firmicutes]          hypothetical protein CLOSPI_02317 [Clostridium spiroforme D
. . Lactobacillus casei BL23 .................................  177   2 hits [firmicutes]          Putative uncharacterized protein [Lactobacillus casei]
. . Streptococcus pyogenes MGAS6180 ..........................  169   8 hits [firmicutes]          cell surface protein [Streptococcus pyogenes MGAS6180]
. . Streptococcus pyogenes SSI-1 .............................  169   8 hits [firmicutes]          hypothetical protein SPs1285 [Streptococcus pyogenes SSI-1]
. . Streptococcus agalactiae COH1 ............................  169   8 hits [firmicutes]          reticulocyte binding protein [Streptococcus agalactiae COH1]
. . Streptococcus pyogenes MGAS10750 .........................  169  16 hits [firmicutes]          Cell surface protein [Streptococcus pyogenes MGAS10750]
. . Streptococcus agalactiae 2603V/R .........................  169   8 hits [firmicutes]          hypothetical protein SAG0421 [Streptococcus agalactiae 2603
. . Streptococcus agalactiae 18RS21 ..........................  169   8 hits [firmicutes]          hypothetical protein SAG0421 [Streptococcus agalactiae 2603
. . Streptococcus agalactiae H36B ............................  169   8 hits [firmicutes]          reticulocyte binding protein [Streptococcus agalactiae H36B]
. . Streptococcus pyogenes MGAS315 ...........................  169   8 hits [firmicutes]          putative surface antigen [Streptococcus pyogenes MGAS315]
. . Streptococcus pyogenes MGAS10270 .........................  167   8 hits [firmicutes]          cell surface protein [Streptococcus pyogenes MGAS10270]
. . Streptococcus pyogenes MGAS9429 ..........................  167   8 hits [firmicutes]          cell surface protein [Streptococcus pyogenes MGAS9429] >gi|
. . Streptococcus pyogenes MGAS2096 ..........................  167   8 hits [firmicutes]          cell surface protein [Streptococcus pyogenes MGAS9429] >gi|
. . Streptococcus agalactiae NEM316 ..........................  167   8 hits [firmicutes]          hypothetical protein gbs0456 [Streptococcus agalactiae NEM3
. . Streptococcus pyogenes M1 GAS ............................  167   7 hits [firmicutes]          hypothetical protein SPy_0843 [Streptococcus pyogenes M1 GA
. . Streptococcus pyogenes MGAS5005 ..........................  167   7 hits [firmicutes]          hypothetical protein SPy_0843 [Streptococcus pyogenes M1 GA
. . Streptococcus agalactiae A909 ............................  167   8 hits [firmicutes]          hypothetical protein SAK_0502 [Streptococcus agalactiae A90
. . Streptococcus pneumoniae SP6-BS73 ........................  167   4 hits [firmicutes]          choline binding protein PcpA [Streptococcus pneumoniae SP6-
. . Streptococcus pyogenes str. Manfredo .....................  166   8 hits [firmicutes]          putative surface-anchored protein [Streptococcus pyogenes s
. . Streptococcus pyogenes MGAS8232 ..........................  166   8 hits [firmicutes]          hypothetical protein spyM18_0903 [Streptococcus pyogenes MG
. . Streptococcus pyogenes M49 591 ...........................  166   8 hits [firmicutes]          COG0556: Helicase subunit of the DNA excision repair comple
. . Streptococcus pyogenes MGAS10394 .........................  166   8 hits [firmicutes]          cell surface protein [Streptococcus pyogenes MGAS10394]
. . Streptococcus pneumoniae CDC0288-04 ......................  165   4 hits [firmicutes]          choline binding protein PcpA [Streptococcus pneumoniae CDC0
. . Streptococcus pneumoniae CDC3059-06 ......................  165   4 hits [firmicutes]          choline binding protein PcpA [Streptococcus pneumoniae CDC3
. . Streptococcus pneumoniae CGSP14 ..........................  165   4 hits [firmicutes]          choline binding protein PcpA [Streptococcus pneumoniae CGSP
. . Streptococcus agalactiae 515 .............................  164   9 hits [firmicutes]          reticulocyte binding protein [Streptococcus agalactiae 515]
. . Streptococcus pneumoniae SP195 ...........................  164   4 hits [firmicutes]          choline binding protein PcpA [Streptococcus pneumoniae SP19
. . Streptococcus pneumoniae SP11-BS70 .......................  164   4 hits [firmicutes]          ornithine carbamoyltransferase [Streptococcus pneumoniae SP
. . Streptococcus pneumoniae MLV-016 .........................  164   4 hits [firmicutes]          ornithine carbamoyltransferase [Streptococcus pneumoniae SP
. . Streptococcus pneumoniae SP19-BS75 .......................  163   4 hits [firmicutes]          choline binding protein PcpA [Streptococcus pneumoniae SP19
. . Streptococcus pneumoniae CDC1873-00 ......................  163   4 hits [firmicutes]          choline binding protein PcpA [Streptococcus pneumoniae SP19
. . Streptococcus pneumoniae CDC1087-00 ......................  163   4 hits [firmicutes]          choline binding protein PcpA [Streptococcus pneumoniae SP19
. . Streptococcus pneumoniae Hungary19A-6 ....................  163   4 hits [firmicutes]          choline binding protein PcpA [Streptococcus pneumoniae SP19
. . Streptococcus pneumoniae TIGR4 ...........................  162   8 hits [firmicutes]          choline binding protein PcpA [Streptococcus pneumoniae TIGR
. . Streptococcus pneumoniae SP18-BS74 .......................  158   4 hits [firmicutes]          ornithine carbamoyltransferase [Streptococcus pneumoniae SP
. . Lactobacillus casei ATCC 334 .............................  156   2 hits [firmicutes]          adhesion exoprotein [Lactobacillus casei ATCC 334]
. . Streptococcus pneumoniae G54 .............................  155   5 hits [firmicutes]          choline binding protein PcpA [Streptococcus pneumoniae G54]
. . Streptococcus pneumoniae R6 ..............................  154   5 hits [firmicutes]          choline binding protein PcpA [Streptococcus pneumoniae R6]
. . Streptococcus pneumoniae D39 .............................  153   4 hits [firmicutes]          choline binding protein PcpA [Streptococcus pneumoniae D39]
. . Streptococcus pneumoniae SP23-BS72 .......................  145   3 hits [firmicutes]          choline binding protein PcpA [Streptococcus pneumoniae SP23
. . Streptococcus agalactiae CJB111 ..........................  141  12 hits [firmicutes]          reticulocyte binding protein [Streptococcus agalactiae CJB1
. . Leuconostoc mesenteroides subsp. mesenteroides ATCC 8293 .  132   4 hits [firmicutes]          subtilisin-like serine protease [Leuconostoc mesenteroides 
. . Streptococcus pneumoniae SP14-BS69 .......................  126   7 hits [firmicutes]          choline binding protein PcpA [Streptococcus pneumoniae SP14
. . Streptococcus equi subsp. zooepidemicus MGCS10565 ........  123  17 hits [firmicutes]          cell surface protein, RBC-binding protein [Streptococcus eq
. . Lactobacillus plantarum WCFS1 ............................  101   7 hits [firmicutes]          cell surface protein (putative) [Lactobacillus plantarum WC
. . Streptococcus pneumoniae SP9-BS68 ........................   91  14 hits [firmicutes]          choline binding protein PcpA [Streptococcus pneumoniae SP9-
. . Listeria monocytogenes FSL J1-175 ........................   90  10 hits [firmicutes]          cell wall surface anchor family protein [Listeria monocytog
. . Listeria monocytogenes FSL J1-194 ........................   90  15 hits [firmicutes]          cell wall surface anchor family protein [Listeria monocytog
. . Listeria monocytogenes FSL N1-017 ........................   90   6 hits [firmicutes]          hypothetical protein LMHG_00875 [Listeria monocytogenes FSL
. . Listeria monocytogenes str. 4b F2365 .....................   89  13 hits [firmicutes]          cell wall surface anchor family protein [Listeria monocytog
. . Listeria monocytogenes str. 4b H7858 .....................   89  14 hits [firmicutes]          cell wall surface anchor family protein [Listeria monocytog
. . Listeria monocytogenes HPB2262 ...........................   89  12 hits [firmicutes]          hypothetical protein LMSG_00080 [Listeria monocytogenes HPB
. . Listeria monocytogenes FSL J2-064 ........................   89   8 hits [firmicutes]          cell wall surface anchor family protein [Listeria monocytog
. . Listeria monocytogenes FSL N3-165 ........................   86   5 hits [firmicutes]          hypothetical protein LMIG_02847 [Listeria monocytogenes FSL
. . Listeria monocytogenes str. 1/2a F6854 ...................   86   5 hits [firmicutes]          conserved domain protein [Listeria monocytogenes str. 1/2a 
. . Listeria monocytogenes F6900 .............................   86   5 hits [firmicutes]          conserved domain protein [Listeria monocytogenes str. 1/2a 
. . Listeria monocytogenes J0161 .............................   86   5 hits [firmicutes]          conserved domain protein [Listeria monocytogenes str. 1/2a 
. . Listeria monocytogenes J2818 .............................   86   5 hits [firmicutes]          conserved domain protein [Listeria monocytogenes str. 1/2a 
. . Lactococcus lactis subsp. cremoris SK11 ..................   85   6 hits [firmicutes]          subtilisin-like serine protease [Lactococcus lactis subsp. 
. . Lactococcus lactis subsp. cremoris MG1363 ................   85   6 hits [firmicutes]          putative secreted protein [Lactococcus lactis subsp. cremor
. . Listeria monocytogenes FSL R2-503 ........................   85   5 hits [firmicutes]          cell wall surface anchor family protein [Listeria monocytog
. . Enterococcus faecalis V583 ...............................   80   3 hits [firmicutes]          hypothetical protein EF2248 [Enterococcus faecalis V583]
. . Lactobacillus acidophilus NCFM ...........................   71   1 hit  [firmicutes]          hypothetical protein LBA1740 [Lactobacillus acidophilus NCF
. . Lactococcus lactis subsp. lactis Il1403 ..................   70   3 hits [firmicutes]          hypothetical protein L58460 [Lactococcus lactis subsp. lact
. . Listeria monocytogenes EGD-e .............................   68   2 hits [firmicutes]          putative membrane associated lipoprotein [Listeria monocyto
. bacterium Ellin514 -----------------------------------------  378  11 hits [verrucomicrobia]     cell surface protein [bacterium Ellin514]
. Flavobacterium psychrophilum JIP02/86 ......................  336  85 hits [CFB group bacteria]  cell surface leucine-rich repeat-containing protein [Flavob
. Treponema denticola ATCC 35405 .............................  315  65 hits [spirochetes]         leucine rich repeat domain-containing protein [Treponema de
. Alistipes putredinis DSM 17216 .............................  311  61 hits [CFB group bacteria]  hypothetical protein ALIPUT_02273 [Alistipes putredinis DSM
. Bacteroides fragilis NCTC 9343 .............................  303  20 hits [CFB group bacteria]  surface protein [Bacteroides fragilis NCTC 9343]
. Synechococcus sp. WH 7805 ..................................  294  17 hits [cyanobacteria]       cell surface protein [Synechococcus sp. WH 7805]
. Shewanella pealeana ATCC 700345 ............................  293  17 hits [g-proteobacteria]    FNIP [Shewanella pealeana ATCC 700345]
. Bacteroides ovatus ATCC 8483 ...............................  293  38 hits [CFB group bacteria]  hypothetical protein BACOVA_04585 [Bacteroides ovatus ATCC 
. Victivallis vadensis ATCC BAA-548 ..........................  243  31 hits [bacteria]            hypothetical protein VvadDRAFT_0933 [Victivallis vadensis A
. Kordia algicida OT-1 .......................................  236  11 hits [CFB group bacteria]  cell surface protein [Kordia algicida OT-1]
. Actinomyces odontolyticus ATCC 17982 .......................  204  11 hits [high GC Gram+]       hypothetical protein ACTODO_00092 [Actinomyces odontolyticu
. Photobacterium sp. SKA34 ...................................  194   8 hits [g-proteobacteria]    cell surface protein [Photobacterium sp. SKA34]
. Flavobacteriales bacterium ALC-1 ...........................  191   8 hits [CFB group bacteria]  cell surface protein [Flavobacteriales bacterium ALC-1]
. Bacteroides stercoris ATCC 43183 ...........................  184  42 hits [CFB group bacteria]  hypothetical protein BACSTE_00622 [Bacteroides stercoris AT
. Mycoplasma agalactiae PG2 ..................................  177  11 hits [mycoplasmas]         lipoprotein [Mycoplasma agalactiae PG2]
. Bacteroides thetaiotaomicron VPI-5482 ......................  169  30 hits [CFB group bacteria]  putative cell surface antigen [Bacteroides thetaiotaomicron
. Bacteroides fragilis YCH46 .................................  169  14 hits [CFB group bacteria]  putative cell surface antigen [Bacteroides fragilis YCH46]
. Shewanella halifaxensis HAW-EB4 ............................  169   9 hits [g-proteobacteria]    hypothetical protein Shal_3565 [Shewanella halifaxensis HAW
. Treponema pallidum subsp. pallidum str. Nichols ............  167   9 hits [spirochetes]         leucine-rich repeat-containing protein [Treponema pallidum 
. Treponema pallidum subsp. pallidum SS14 ....................  167   9 hits [spirochetes]         leucine-rich repeat-containing protein [Treponema pallidum 
. Bacteroides caccae ATCC 43185 ..............................  165  12 hits [CFB group bacteria]  hypothetical protein BACCAC_03700 [Bacteroides caccae ATCC 
. Bacteroides vulgatus ATCC 8482 .............................  158  13 hits [CFB group bacteria]  hypothetical protein BVU_3191 [Bacteroides vulgatus ATCC 84
. Acholeplasma laidlawii PG-8A ...............................  156  11 hits [mycoplasmas]         putative surface-anchored antigen, BspA-like protein [Achol
. Bacteroides coprocola DSM 17136 ............................  151  11 hits [CFB group bacteria]  hypothetical protein BACCOP_00841 [Bacteroides coprocola DS
. Escherichia coli E24377A ...................................  133   4 hits [enterobacteria]      hypothetical protein EcE24377A_1450 [Escherichia coli E2437
. candidate division TM7 single-cell isolate TM7b ............  121  11 hits [bacteria]            Probable cell surface protein (Leucine-rich repeat protein)
. Fusobacterium nucleatum subsp. nucleatum ATCC 25586 ........  116   9 hits [fusobacteria]        putative cytoplasmic protein [Fusobacterium nucleatum subsp
. Bacteroides intestinalis DSM 17393 .........................  115  14 hits [CFB group bacteria]  hypothetical protein BACINT_00102 [Bacteroides intestinalis
. Fusobacterium nucleatum subsp. vincentii ATCC 49256 ........  113   4 hits [fusobacteria]        Hypothetical Cytosolic Protein [Fusobacterium nucleatum sub
. Flavobacterium johnsoniae UW101 ............................   93   4 hits [CFB group bacteria]  hypothetical protein Fjoh_3983 [Flavobacterium johnsoniae U
. Bacteroides capillosus ATCC 29799 ..........................   93  27 hits [CFB group bacteria]  hypothetical protein BACCAP_00990 [Bacteroides capillosus A
. Parabacteroides distasonis ATCC 8503 .......................   93  10 hits [CFB group bacteria]  putative surface antigen BspA [Parabacteroides distasonis A
. Bacteroides uniformis ATCC 8492 ............................   83   6 hits [CFB group bacteria]  hypothetical protein BACUNI_02088 [Bacteroides uniformis AT
. Anabaena variabilis ATCC 29413 .............................   75   1 hit  [cyanobacteria]       small GTP-binding protein domain-containing protein [Anabae
. Blastopirellula marina DSM 3645 ............................   74   2 hits [planctomycetes]      serine/threonine protein kinase [Blastopirellula marina DSM
. Mycoplasma arthritidis 158L3-1 .............................   67   4 hits [mycoplasmas]         hypothetical lipoprotein [Mycoplasma arthritidis 158L3-1]
. Nostoc punctiforme PCC 73102 ...............................   67   2 hits [cyanobacteria]       small GTP-binding protein [Nostoc punctiforme PCC 73102]
. Mycoplasma hyopneumoniae J .................................   65   9 hits [mycoplasmas]         hypothetical protein MHJ_0123 [Mycoplasma hyopneumoniae J]
. Mycoplasma hyopneumoniae 7448 ..............................   65   9 hits [mycoplasmas]         hypothetical protein MHP7448_0127 [Mycoplasma hyopneumoniae
. Mycoplasma mobile 163K .....................................   64   4 hits [mycoplasmas]         hypothetical protein MMOB1360 [Mycoplasma mobile 163K]
. Mycoplasma hyopneumoniae 232 ...............................   64   8 hits [mycoplasmas]         hypothetical protein mhp254 [Mycoplasma hyopneumoniae 232]
. Microscilla marina ATCC 23134 ..............................   63   1 hit  [CFB group bacteria]  leucine-rich repeat containing protein [Microscilla marina 
. Mycoplasma pulmonis UAB CTIP ...............................   59   7 hits [mycoplasmas]         hypothetical protein MYPU_2110 [Mycoplasma pulmonis UAB CTI
. Vibrio cholerae V52 ........................................   56   2 hits [g-proteobacteria]    bifunctional GlmU protein [Vibrio cholerae V52]
. Yersinia pseudotuberculosis IP 31758 .......................   55   1 hit  [enterobacteria]      hypothetical protein YpsIP31758_2931 [Yersinia pseudotuberc
```

---

**Organism Report**

```
  Clostridium leptum DSM 753 [firmicutes] taxid 428125
 ref|ZP_02081532.1| hypothetical protein CLOLEP_03013 [Clos...     472  8e-132

  Eubacterium siraeum DSM 15702 [firmicutes] taxid 428128
 ref|ZP_02423818.1| hypothetical protein EUBSIR_02697 [Euba...     426  7e-118
 ref|ZP_02423777.1| hypothetical protein EUBSIR_02655 [Euba...     377  4e-103
 ref|ZP_02421667.1| hypothetical protein EUBSIR_00498 [Euba...     363  5e-99
 ref|ZP_02421573.1| hypothetical protein EUBSIR_00400 [Euba...     351  2e-95
 ref|ZP_02422346.1| hypothetical protein EUBSIR_01193 [Euba...     237  4e-61
 ref|ZP_02423885.1| hypothetical protein EUBSIR_02767 [Euba...     233  9e-60
 ref|ZP_02422266.1| hypothetical protein EUBSIR_01108 [Euba...      98  3e-19

  Clostridium spiroforme DSM 1552 [firmicutes] taxid 428126
 ref|ZP_02868475.1| hypothetical protein CLOSPI_02317 [Clos...     379  1e-103

  bacterium Ellin514 [verrucomicrobia] taxid 320771
 ref|ZP_02967646.1| cell surface protein [bacterium Ellin514]      378  2e-103
 ref|ZP_02970487.1| hypothetical protein CflavDRAFT_5610 [b...     278  3e-73

  Epulopiscium sp. 'N.t. morphotype B' [firmicutes] taxid 420336
 ref|ZP_02693515.1| cell surface protein [Epulopiscium sp. ...     355  2e-96
 ref|ZP_02693548.1| cell surface protein [Epulopiscium sp. ...     335  2e-90
 ref|ZP_02693390.1| cell surface protein [Epulopiscium sp. ...     291  3e-77
 ref|ZP_02692178.1| Probable cell surface protein (Leucine-...     290  5e-77
 ref|ZP_02693554.1| Probable cell surface protein (Leucine-...     248  2e-64
 ref|ZP_02691993.1| Probable cell surface protein (Leucine-...     240  6e-62
 ref|ZP_02693356.1| cell surface protein [Epulopiscium sp. ...     234  3e-60
 ref|ZP_02691805.1| cell surface protein [Epulopiscium sp. ...     217  8e-55
 ref|ZP_02692842.1| Probable cell surface protein (Leucine-...     212  2e-53
 ref|ZP_02692410.1| cell surface protein [Epulopiscium sp. ...     209  1e-52
 ref|ZP_02693389.1| cell surface protein [Epulopiscium sp. ...     208  3e-52
 ref|ZP_02691806.1| cell surface protein [Epulopiscium sp. ...     206  1e-51
 ref|ZP_02693306.1| Probable cell surface protein (Leucine-...     182  1e-44
 ref|ZP_02691807.1| Leucine-rich repeat (LRR) protein-like ...     180  7e-44
 ref|ZP_02692841.1| cell surface protein [Epulopiscium sp. ...     165  2e-39
 ref|ZP_02693326.1| cell surface protein [Epulopiscium sp. ...     162  2e-38
 ref|ZP_02693025.1| cell surface protein [Epulopiscium sp. ...     153  1e-35
 ref|ZP_02692843.1| Probable cell surface protein (Leucine-...     145  3e-33
 ref|ZP_02692800.1| cell surface protein [Epulopiscium sp. ...     114  5e-24

  Anaerofustis stercorihominis DSM 17244 [firmicutes] taxid 445971
 ref|ZP_02861219.1| hypothetical protein ANASTE_00419 [Anae...     343  6e-93
 ref|ZP_02860961.1| hypothetical protein ANASTE_00152 [Anae...     296  1e-78
 ref|ZP_02861046.1| hypothetical protein ANASTE_00239 [Anae...     284  3e-75
 ref|ZP_02860940.1| hypothetical protein ANASTE_00131 [Anae...     284  5e-75
 ref|ZP_02861139.1| hypothetical protein ANASTE_00332 [Anae...     246  1e-63
 ref|ZP_02861690.1| hypothetical protein ANASTE_00900 [Anae...     237  4e-61
 ref|ZP_02862016.1| hypothetical protein ANASTE_01229 [Anae...     208  4e-52
 ref|ZP_02861865.1| hypothetical protein ANASTE_01075 [Anae...     187  7e-46
 ref|ZP_02862872.1| hypothetical protein ANASTE_02099 [Anae...     169  2e-40
 ref|ZP_02861981.1| hypothetical protein ANASTE_01194 [Anae...     164  7e-39
 ref|ZP_02861254.1| hypothetical protein ANASTE_00454 [Anae...     141  4e-32
 ref|ZP_02863047.1| hypothetical protein ANASTE_02280 [Anae...     118  2e-25
 ref|ZP_02862435.1| hypothetical protein ANASTE_01650 [Anae...     107  6e-22
 ref|ZP_02861123.1| hypothetical protein ANASTE_00316 [Anae...      78  6e-13
 ref|ZP_02863091.1| hypothetical protein ANASTE_02333 [Anae...      56  2e-06

  Flavobacterium psychrophilum JIP02/86 [CFB group bacteria] taxid 402612
 ref|YP_001295104.1| cell surface leucine-rich repeat-conta...     336  6e-91
 ref|YP_001295109.1| cell surface leucine-rich repeat-conta...     328  3e-88
 ref|YP_001295111.1| cell surface leucine-rich repeat-conta...     299  1e-79
 ref|YP_001295103.1| cell surface leucine-rich repeat-conta...     259  1e-67
 ref|YP_001295102.1| cell surface leucine-rich repeat-conta...     242  2e-62
 ref|YP_001295115.1| cell surface leucine-rich repeat-conta...     228  2e-58
 ref|YP_001295108.1| cell surface leucine-rich repeat-conta...     212  2e-53
 ref|YP_001295113.1| cell surface leucine-rich repeat-conta...     187  5e-46
 ref|YP_001295112.1| cell surface leucine-rich repeat-conta...     174  7e-42
 ref|YP_001295110.1| cell surface leucine-rich repeat-conta...     174  8e-42
 ref|YP_001295105.1| cell surface leucine-rich repeat-conta...     165  2e-39
 ref|YP_001295116.1| cell surface leucine-rich repeat-conta...     165  3e-39
 ref|YP_001295106.1| cell surface leucine-rich repeat-conta...     140  9e-32
 ref|YP_001295107.1| cell surface leucine-rich repeat-conta...     138  3e-31
 ref|YP_001295114.1| cell surface leucine-rich repeat-conta...     116  1e-24

  Syntrophomonas wolfei subsp. wolfei str. Goettingen [firmicutes] taxid 335541
 ref|YP_754057.1| leucine-rich repeat-containing protein [S...     321  3e-86

  Treponema denticola ATCC 35405 [spirochetes] taxid 243275
 ref|NP_972265.1| leucine rich repeat domain-containing pro...     315  3e-84
 ref|NP_972858.1| surface antigen BspA, putative [Treponema...     263  7e-69
 ref|NP_972561.1| surface protein, putative [Treponema dent...     224  6e-57
 ref|NP_971188.1| surface protein, putative [Treponema dent...     175  2e-42
 ref|NP_973333.1| surface antigen, putative [Treponema dent...     145  2e-33
 ref|NP_973041.1| surface protein, putative [Treponema dent...     116  1e-24
 ref|NP_972586.1| hypothetical protein TDE1983 [Treponema d...      96  1e-18
 ref|NP_971352.1| hypothetical protein TDE0741 [Treponema d...      94  7e-18
 ref|NP_971588.1| hypothetical protein TDE0978 [Treponema d...      88  3e-16
 ref|NP_973055.1| hypothetical protein TDE2457 [Treponema d...      64  6e-09
 ref|NP_973335.1| hypothetical protein TDE2737 [Treponema d...      62  3e-08

  Alistipes putredinis DSM 17216 [CFB group bacteria] taxid 445970
 ref|ZP_02426114.1| hypothetical protein ALIPUT_02273 [Alis...     311  3e-83
 ref|ZP_02424091.1| hypothetical protein ALIPUT_00206 [Alis...     299  9e-80
 ref|ZP_02424866.1| hypothetical protein ALIPUT_00999 [Alis...     180  6e-44
 ref|ZP_02423940.1| hypothetical protein ALIPUT_00054 [Alis...     179  2e-43
 ref|ZP_02423935.1| hypothetical protein ALIPUT_00049 [Alis...     160  1e-37
 ref|ZP_02425898.1| hypothetical protein ALIPUT_02055 [Alis...     130  6e-29
 ref|ZP_02424814.1| hypothetical protein ALIPUT_00944 [Alis...      61  5e-08

  Ruminococcus torques ATCC 27756 [firmicutes] taxid 411460
 ref|ZP_01969043.1| hypothetical protein RUMTOR_02627 [Rumi...     310  4e-83
 ref|ZP_01969039.1| hypothetical protein RUMTOR_02623 [Rumi...     298  2e-79
 ref|ZP_01969285.1| hypothetical protein RUMTOR_02870 [Rumi...     278  3e-73
 ref|ZP_01969242.1| hypothetical protein RUMTOR_02827 [Rumi...     151  4e-35
 ref|ZP_01969140.1| hypothetical protein RUMTOR_02725 [Rumi...      66  1e-09
 ref|ZP_01969326.1| hypothetical protein RUMTOR_02914 [Rumi...      66  1e-09

  Coprococcus eutactus ATCC 27759 [firmicutes] taxid 411474
 ref|ZP_02205711.1| hypothetical protein COPEUT_00473 [Copr...     305  2e-81
 ref|ZP_02207819.1| hypothetical protein COPEUT_02644 [Copr...     160  7e-38
 ref|ZP_02206899.1| hypothetical protein COPEUT_01691 [Copr...      97  8e-19
 ref|ZP_02205710.1| hypothetical protein COPEUT_00472 [Copr...      88  5e-16
 ref|ZP_02205610.1| hypothetical protein COPEUT_00372 [Copr...      66  1e-09
 ref|ZP_02205380.1| hypothetical protein COPEUT_00139 [Copr...      58  4e-07

  Bacteroides fragilis NCTC 9343 [CFB group bacteria] taxid 272559
 ref|YP_213662.1| surface protein [Bacteroides fragilis NCT...     303  9e-81
 ref|YP_211343.1| putative surface antigen [Bacteroides fra...     168  3e-40
 ref|YP_211460.1| hypothetical protein BF1824 [Bacteroides ...      79  2e-13
 ref|YP_211145.1| hypothetical protein BF1499 [Bacteroides ...      57  8e-07

  Synechococcus sp. WH 7805 [cyanobacteria] taxid 59931
 ref|ZP_01123009.1| cell surface protein [Synechococcus sp....     294  3e-78
 ref|ZP_01125124.1| cell surface protein [Synechococcus sp....     235  2e-60
 ref|ZP_01123034.1| calcium-binding protein, hemolysin-type...      57  8e-07

  Shewanella pealeana ATCC 700345 [g-proteobacteria] taxid 398579
 ref|YP_001500360.1| FNIP [Shewanella pealeana ATCC 700345]        293  8e-78
 ref|YP_001502689.1| TPR repeat-containing protein [Shewane...     216  1e-54
 ref|YP_001503317.1| hypothetical protein Spea_3469 [Shewan...     162  2e-38

  Bacteroides ovatus ATCC 8483 [CFB group bacteria] taxid 411476
 ref|ZP_02067577.1| hypothetical protein BACOVA_04585 [Bact...     293  1e-77
 ref|ZP_02063418.1| hypothetical protein BACOVA_00366 [Bact...     197  5e-49
 ref|ZP_02064596.1| hypothetical protein BACOVA_01565 [Bact...     160  6e-38
 ref|ZP_02063968.1| hypothetical protein BACOVA_00927 [Bact...     120  1e-25
 ref|ZP_02063583.1| hypothetical protein BACOVA_00533 [Bact...      70  2e-10

  Clostridium sp. L2-50 [firmicutes] taxid 411489
 ref|ZP_02075891.1| hypothetical protein CLOL250_02668 [Clo...     264  4e-69
 ref|ZP_02073995.1| hypothetical protein CLOL250_00753 [Clo...     237  4e-61
 ref|ZP_02073625.1| hypothetical protein CLOL250_00366 [Clo...     226  1e-57
 ref|ZP_02075562.1| hypothetical protein CLOL250_02338 [Clo...     223  1e-56
 ref|ZP_02075561.1| hypothetical protein CLOL250_02337 [Clo...     170  8e-41
 ref|ZP_02075272.1| hypothetical protein CLOL250_02048 [Clo...     125  2e-27
 ref|ZP_02075887.1| hypothetical protein CLOL250_02664 [Clo...      92  3e-17
 ref|ZP_02074853.1| hypothetical protein CLOL250_01629 [Clo...      67  1e-09
 ref|ZP_02074719.1| hypothetical protein CLOL250_01495 [Clo...      62  2e-08
 ref|ZP_02075407.1| hypothetical protein CLOL250_02183 [Clo...      61  4e-08

  Desulfitobacterium hafniense Y51 [firmicutes] taxid 138119
 ref|YP_520089.1| hypothetical protein DSY3856 [Desulfitoba...     256  1e-66

  Clostridium phytofermentans ISDg [firmicutes] taxid 357809
 ref|YP_001559082.1| Ig domain-containing protein [Clostrid...     253  9e-66
 ref|YP_001560465.1| hypothetical protein Cphy_3373 [Clostr...     225  3e-57
 ref|YP_001559102.1| hypothetical protein Cphy_1995 [Clostr...     124  5e-27
 ref|YP_001558337.1| hypothetical protein Cphy_1220 [Clostr...      93  1e-17
 ref|YP_001558066.1| hypothetical protein Cphy_0945 [Clostr...      57  9e-07
 ref|YP_001558062.1| hypothetical protein Cphy_0939 [Clostr...      56  2e-06

  Clostridium beijerinckii NCIMB 8052 [firmicutes] taxid 290402
 ref|YP_001310293.1| cell wall binding repeat-containing pr...     249  2e-64
 ref|YP_001309881.1| cell wall binding repeat-containing pr...     207  6e-52
 ref|YP_001310322.1| cell wall binding repeat-containing pr...     204  4e-51
 ref|YP_001308736.1| fibronectin, type III domain-containin...     139  1e-31
 ref|YP_001309903.1| cell wall binding repeat-containing pr...      84  6e-15

  Victivallis vadensis ATCC BAA-548 [bacteria] taxid 340101
 ref|ZP_01922308.1| hypothetical protein VvadDRAFT_0933 [Vi...     243  6e-63
 ref|ZP_01923169.1| cell surface protein [Victivallis vaden...     218  2e-55
 ref|ZP_01923170.1| hypothetical protein VvadDRAFT_1399 [Vi...     162  2e-38

  Kordia algicida OT-1 [CFB group bacteria] taxid 391587
 ref|ZP_02163681.1| cell surface protein [Kordia algicida O...     236  1e-60

  Clostridium scindens ATCC 35704 [firmicutes] taxid 411468
 ref|ZP_02433176.1| hypothetical protein CLOSCI_03447 [Clos...     231  4e-59
 ref|ZP_02429939.1| hypothetical protein CLOSCI_00143 [Clos...      65  5e-09

  Clostridium sporogenes ATCC 15579 [firmicutes] taxid 471871
 ref|ZP_02993488.1| hypothetical protein CLOSPO_00560 [Clos...     230  7e-59

  Eubacterium ventriosum ATCC 27560 [firmicutes] taxid 411463
 ref|ZP_02027186.1| hypothetical protein EUBVEN_02455 [Euba...     228  3e-58
 ref|ZP_02025833.1| hypothetical protein EUBVEN_01088 [Euba...     205  3e-51
 ref|ZP_02026466.1| hypothetical protein EUBVEN_01726 [Euba...     174  5e-42
 ref|ZP_02026087.1| hypothetical protein EUBVEN_01343 [Euba...     172  3e-41
 ref|ZP_02026084.1| hypothetical protein EUBVEN_01340 [Euba...     158  4e-37
 ref|ZP_02026088.1| hypothetical protein EUBVEN_01344 [Euba...     152  3e-35
 ref|ZP_02026085.1| hypothetical protein EUBVEN_01341 [Euba...     151  4e-35
 ref|ZP_02027368.1| hypothetical protein EUBVEN_02638 [Euba...     138  2e-31
 ref|ZP_02026535.1| hypothetical protein EUBVEN_01798 [Euba...      98  4e-19
 ref|ZP_02026664.1| hypothetical protein EUBVEN_01927 [Euba...      85  4e-15
 ref|ZP_02025927.1| hypothetical protein EUBVEN_01183 [Euba...      84  8e-15
 ref|ZP_02027384.1| hypothetical protein EUBVEN_02654 [Euba...      76  2e-12

  Dorea formicigenerans ATCC 27755 [firmicutes] taxid 411461
 ref|ZP_02235444.1| hypothetical protein DORFOR_02330 [Dore...     224  7e-57

  Clostridium botulinum F str. Langeland [firmicutes] taxid 441772
 ref|YP_001389750.1| putative cell surface protein [Clostri...     223  9e-57

  Clostridium butyricum 5521 [firmicutes] taxid 447214
 ref|ZP_02949217.1| surface protein PspC [Clostridium butyr...     220  8e-56
 ref|ZP_02950501.1| surface protein PspC [Clostridium butyr...     214  4e-54

  Actinomyces odontolyticus ATCC 17982 [high GC Gram+] taxid 411466
 ref|ZP_02043254.1| hypothetical protein ACTODO_00092 [Acti...     204  3e-51
 ref|ZP_02043255.1| hypothetical protein ACTODO_00093 [Acti...     196  1e-48

  Clostridium bartlettii DSM 16795 [firmicutes] taxid 445973
 ref|ZP_02211019.1| hypothetical protein CLOBAR_00617 [Clos...     202  1e-50
 ref|ZP_02211373.1| hypothetical protein CLOBAR_00986 [Clos...     184  5e-45
 ref|ZP_02212394.1| hypothetical protein CLOBAR_02011 [Clos...      63  1e-08

  Photobacterium sp. SKA34 [g-proteobacteria] taxid 121723
 ref|ZP_01162683.1| cell surface protein [Photobacterium sp...     194  4e-48

  Flavobacteriales bacterium ALC-1 [CFB group bacteria] taxid 391603
 ref|ZP_02182632.1| cell surface protein [Flavobacteriales ...     191  5e-47

  Bacteroides stercoris ATCC 43183 [CFB group bacteria] taxid 449673
 ref|ZP_02434396.1| hypothetical protein BACSTE_00622 [Bact...     184  6e-45
 ref|ZP_02435797.1| hypothetical protein BACSTE_02048 [Bact...     171  3e-41
 ref|ZP_02435130.1| hypothetical protein BACSTE_01368 [Bact...     120  1e-25
 ref|ZP_02434373.1| hypothetical protein BACSTE_00599 [Bact...      92  3e-17
 ref|ZP_02435588.1| hypothetical protein BACSTE_01835 [Bact...      85  4e-15
 ref|ZP_02435741.1| hypothetical protein BACSTE_01989 [Bact...      75  2e-12

  Ruminococcus obeum ATCC 29174 [firmicutes] taxid 411459
 ref|ZP_01963695.1| hypothetical protein RUMOBE_01418 [Rumi...     182  2e-44
 ref|ZP_01964882.1| hypothetical protein RUMOBE_02612 [Rumi...     131  5e-29
 ref|ZP_01962356.1| hypothetical protein RUMOBE_00069 [Rumi...      66  2e-09
 ref|ZP_01964702.1| hypothetical protein RUMOBE_02427 [Rumi...      56  2e-06

  Finegoldia magna ATCC 29328 [firmicutes] taxid 334413
 ref|YP_001692763.1| putative N-acetylmuramoyl-L-alanine am...     180  5e-44
 ref|YP_001692266.1| putative chimeric erythrocyte-binding ...      91  4e-17

  Mycoplasma agalactiae PG2 [mycoplasmas] taxid 347257
 ref|YP_001256394.1| lipoprotein [Mycoplasma agalactiae PG2]       177  6e-43
 ref|YP_001256759.1| lipoprotein [Mycoplasma agalactiae PG2]        67  9e-10
 ref|YP_001256393.1| lipoprotein [Mycoplasma agalactiae PG2]        58  6e-07

  Lactobacillus casei BL23 [firmicutes] taxid 543734
 ref|YP_001989023.1| Putative uncharacterized protein [Lact...     177  6e-43

  Clostridium botulinum A3 str. Loch Maree [firmicutes] taxid 498214
 ref|YP_001785718.1| putative cell surface protein [Clostri...     177  9e-43

  Clostridium botulinum A str. ATCC 3502 [firmicutes] taxid 413999
 ref|YP_001252924.1| cell surface protein [Clostridium botu...     174  4e-42

  Clostridium botulinum NCTC 2916 [firmicutes] taxid 445335
 ref|ZP_02612389.1| putative cell surface protein [Clostrid...     172  2e-41

  Clostridium botulinum B1 str. Okra [firmicutes] taxid 498213
 ref|YP_001780034.1| putative cell surface protein [Clostri...     170  7e-41

  Bacteroides thetaiotaomicron VPI-5482 [CFB group bacteria] taxid 226186
 ref|NP_810809.1| putative cell surface antigen [Bacteroide...     169  1e-40
 ref|NP_813351.1| putative cell surface protein [Bacteroide...     115  4e-24
 ref|NP_810153.1| hypothetical protein BT_1240 [Bacteroides...      79  2e-13
 ref|NP_810684.1| putative cell surface protein [Bacteroide...      67  1e-09

  Streptococcus pyogenes MGAS6180 [firmicutes] taxid 319701
 ref|YP_280101.1| cell surface protein [Streptococcus pyoge...     169  1e-40
 ref|YP_280985.1| putative Fe3+-siderophore transport prote...      73  9e-12

  Streptococcus pyogenes SSI-1 [firmicutes] taxid 193567
 ref|NP_802547.1| hypothetical protein SPs1285 [Streptococc...     169  1e-40
 ref|NP_801568.1| hypothetical protein SPs0306 [Streptococc...      73  1e-11

  Streptococcus agalactiae COH1 [firmicutes] taxid 342616
 ref|ZP_00784420.1| reticulocyte binding protein [Streptoco...     169  1e-40

  Bacteroides fragilis YCH46 [CFB group bacteria] taxid 295405
 ref|YP_098979.1| putative cell surface antigen [Bacteroide...     169  1e-40
 ref|YP_099029.1| hypothetical protein BF1748 [Bacteroides ...      79  2e-13

  Shewanella halifaxensis HAW-EB4 [g-proteobacteria] taxid 458817
 ref|YP_001675765.1| hypothetical protein Shal_3565 [Shewan...     169  2e-40

  Streptococcus pyogenes MGAS10750 [firmicutes] taxid 370554
 ref|YP_602236.1| Cell surface protein [Streptococcus pyoge...     169  2e-40
 ref|YP_603188.1| Putative surface protein [Streptococcus p...      89  2e-16
 ref|YP_603083.1| Putative Fe3+-siderophore transport prote...      73  1e-11

  Streptococcus agalactiae 2603V/R [firmicutes] taxid 208435
 ref|NP_687455.1| hypothetical protein SAG0421 [Streptococc...     169  2e-40

  Streptococcus agalactiae 18RS21 [firmicutes] taxid 342613
 ref|ZP_00780863.1| cell wall surface anchor family protein...     169  2e-40

  Streptococcus agalactiae H36B [firmicutes] taxid 342615
 ref|ZP_00783119.1| reticulocyte binding protein [Streptoco...     169  2e-40

  Streptococcus pyogenes MGAS315 [firmicutes] taxid 198466
 ref|NP_664373.1| putative surface antigen [Streptococcus p...     169  2e-40
 ref|NP_665365.1| hypothetical protein SpyM3_1561 [Streptoc...      73  1e-11

  Clostridium botulinum Bf [firmicutes] taxid 445336
 ref|ZP_02615908.1| putative cell surface protein [Clostrid...     169  2e-40

  Streptococcus pyogenes MGAS10270 [firmicutes] taxid 370552
 ref|YP_598319.1| cell surface protein [Streptococcus pyoge...     167  4e-40
 ref|YP_599207.1| iron ABC transporter permease [Streptococ...      73  1e-11

  Treponema pallidum subsp. pallidum str. Nichols [spirochetes] taxid 243276
 ref|NP_218665.1| leucine-rich repeat-containing protein [T...     167  5e-40

  Treponema pallidum subsp. pallidum SS14 [spirochetes] taxid 455434
 ref|YP_001933230.1| leucine-rich repeat protein TpLRR [Tre...     167  5e-40

  Streptococcus pyogenes MGAS9429 [firmicutes] taxid 370551
 ref|YP_596438.1| cell surface protein [Streptococcus pyoge...     167  5e-40
 ref|YP_597265.1| putative Fe3+-siderophore transport prote...      73  1e-11

  Streptococcus pyogenes MGAS2096 [firmicutes] taxid 370553
 ref|YP_600315.1| cell surface protein [Streptococcus pyoge...     167  5e-40
 ref|YP_601153.1| iron ABC transporter permease [Streptococ...      73  1e-11

  Streptococcus agalactiae NEM316 [firmicutes] taxid 211110
 ref|NP_734924.1| hypothetical protein gbs0456 [Streptococc...     167  7e-40

  Streptococcus pyogenes M1 GAS [firmicutes] taxid 160490
 ref|NP_269051.1| hypothetical protein SPy_0843 [Streptococ...     167  8e-40
 ref|NP_269809.1| hypothetical protein SPy_1798 [Streptococ...      73  9e-12

  Streptococcus pyogenes MGAS5005 [firmicutes] taxid 293653
 ref|YP_282014.1| cell surface protein [Streptococcus pyoge...     167  8e-40
 ref|YP_282893.1| putative Fe3+-siderophore transport prote...      73  9e-12

  Streptococcus agalactiae A909 [firmicutes] taxid 205921
 ref|YP_329139.1| hypothetical protein SAK_0502 [Streptococ...     167  9e-40

  Streptococcus pneumoniae SP6-BS73 [firmicutes] taxid 406557
 ref|ZP_01820191.1| choline binding protein PcpA [Streptoco...     167  9e-40

  Streptococcus pyogenes str. Manfredo [firmicutes] taxid 160491
 ref|YP_001128699.1| putative surface-anchored protein [Str...     166  1e-39
 ref|YP_001127907.1| putative iron transport-associated pro...      73  1e-11

  Streptococcus pyogenes MGAS8232 [firmicutes] taxid 186103
 ref|NP_607054.1| hypothetical protein spyM18_0903 [Strepto...     166  1e-39
 ref|NP_607878.1| hypothetical protein spyM18_1868 [Strepto...      73  1e-11

  Streptococcus pyogenes M49 591 [firmicutes] taxid 294934
 ref|ZP_00366186.1| COG0556: Helicase subunit of the DNA ex...     166  1e-39
 ref|ZP_00366197.1| COG4886: Leucine-rich repeat (LRR) prot...      73  1e-11

  Streptococcus pyogenes MGAS10394 [firmicutes] taxid 286636
 ref|YP_059988.1| cell surface protein [Streptococcus pyoge...     166  2e-39
 ref|YP_060840.1| iron ABC transporter permease [Streptococ...      73  1e-11

  Bacteroides caccae ATCC 43185 [CFB group bacteria] taxid 411901
 ref|ZP_01962054.1| hypothetical protein BACCAC_03700 [Bact...     165  2e-39
 ref|ZP_01959472.1| hypothetical protein BACCAC_01078 [Bact...      75  3e-12

  Streptococcus pneumoniae CDC0288-04 [firmicutes] taxid 453364
 ref|ZP_02715811.1| choline binding protein PcpA [Streptoco...     165  2e-39

  Streptococcus pneumoniae CDC3059-06 [firmicutes] taxid 453365
 ref|ZP_02718249.1| choline binding protein PcpA [Streptoco...     165  2e-39

  Streptococcus pneumoniae CGSP14 [firmicutes] taxid 516950
 ref|YP_001836822.1| choline binding protein PcpA [Streptoc...     165  4e-39

  Streptococcus agalactiae 515 [firmicutes] taxid 342614
 ref|ZP_00790303.1| reticulocyte binding protein [Streptoco...     164  5e-39

  Streptococcus pneumoniae SP195 [firmicutes] taxid 453363
 ref|ZP_02713402.1| choline binding protein PcpA [Streptoco...     164  8e-39

  Streptococcus pneumoniae SP11-BS70 [firmicutes] taxid 406559
 ref|ZP_01825578.1| ornithine carbamoyltransferase [Strepto...     164  8e-39

  Streptococcus pneumoniae MLV-016 [firmicutes] taxid 453366
 ref|ZP_02721913.1| choline binding protein PcpA [Streptoco...     164  8e-39

  Streptococcus pneumoniae SP19-BS75 [firmicutes] taxid 406562
 ref|ZP_01833146.1| choline binding protein PcpA [Streptoco...     163  9e-39

  Streptococcus pneumoniae CDC1873-00 [firmicutes] taxid 453362
 ref|ZP_02708989.1| choline binding protein PcpA [Streptoco...     163  9e-39

  Streptococcus pneumoniae CDC1087-00 [firmicutes] taxid 453361
 ref|ZP_02710750.1| choline binding protein PcpA [Streptoco...     163  9e-39

  Streptococcus pneumoniae Hungary19A-6 [firmicutes] taxid 487214
 ref|YP_001695497.1| choline binding protein PcpA [Streptoc...     163  9e-39

  Streptococcus pneumoniae TIGR4 [firmicutes] taxid 170187
 ref|NP_346554.1| choline binding protein PcpA [Streptococc...     162  2e-38
 ref|ZP_01407682.1| hypothetical protein SpneT_02001904 [St...     151  4e-35

  Bacteroides vulgatus ATCC 8482 [CFB group bacteria] taxid 435590
 ref|YP_001300444.1| hypothetical protein BVU_3191 [Bactero...     158  2e-37
 ref|YP_001299036.1| putative cell surface protein, putativ...      70  1e-10

  Streptococcus pneumoniae SP18-BS74 [firmicutes] taxid 406561
 ref|ZP_01830569.1| ornithine carbamoyltransferase [Strepto...     158  4e-37

  Acholeplasma laidlawii PG-8A [mycoplasmas] taxid 441768
 ref|YP_001620762.1| putative surface-anchored antigen, Bsp...     156  1e-36
 ref|YP_001621304.1| hypothetical protein ACL_1334 [Acholep...      59  3e-07

  Lactobacillus casei ATCC 334 [firmicutes] taxid 321967
 ref|YP_808047.1| adhesion exoprotein [Lactobacillus casei ...     156  1e-36

  Streptococcus pneumoniae G54 [firmicutes] taxid 512566
 ref|YP_002038725.1| choline binding protein PcpA [Streptoc...     155  3e-36

  Streptococcus pneumoniae R6 [firmicutes] taxid 171101
 ref|NP_359536.1| choline binding protein PcpA [Streptococc...     154  7e-36

  Streptococcus pneumoniae D39 [firmicutes] taxid 373153
 ref|YP_817353.1| choline binding protein PcpA [Streptococc...     153  9e-36

  Bacteroides coprocola DSM 17136 [CFB group bacteria] taxid 470145
 ref|ZP_03008989.1| hypothetical protein BACCOP_00841 [Bact...     151  5e-35
 ref|ZP_03010628.1| hypothetical protein BACCOP_02509 [Bact...     146  1e-33

  Streptococcus pneumoniae SP23-BS72 [firmicutes] taxid 406563
 ref|ZP_01835022.1| choline binding protein PcpA [Streptoco...     145  2e-33

  Streptococcus agalactiae CJB111 [firmicutes] taxid 342617
 ref|ZP_00787671.1| reticulocyte binding protein [Streptoco...     141  5e-32

  Clostridium sp. SS2/1 [firmicutes] taxid 411484
 ref|ZP_02440143.1| hypothetical protein CLOSS21_02635 [Clo...     134  5e-30
 ref|ZP_02437743.1| hypothetical protein CLOSS21_00178 [Clo...      98  4e-19
 ref|ZP_02439407.1| hypothetical protein CLOSS21_01873 [Clo...      94  6e-18
 ref|ZP_02439405.1| hypothetical protein CLOSS21_01871 [Clo...      88  4e-16
 ref|ZP_02440445.1| hypothetical protein CLOSS21_02949 [Clo...      76  1e-12
 ref|ZP_02440444.1| hypothetical protein CLOSS21_02948 [Clo...      64  6e-09
 ref|ZP_02439803.1| hypothetical protein CLOSS21_02285 [Clo...      64  8e-09
 ref|ZP_02438145.1| hypothetical protein CLOSS21_00585 [Clo...      60  1e-07
 ref|ZP_02439815.1| hypothetical protein CLOSS21_02297 [Clo...      58  6e-07

  Escherichia coli E24377A [enterobacteria] taxid 331111
 ref|YP_001462548.1| hypothetical protein EcE24377A_1450 [E...     133  9e-30

  Leuconostoc mesenteroides subsp. mesenteroides ATCC 8293 [firmicutes] taxid 203120
 ref|YP_819342.1| subtilisin-like serine protease [Leuconos...     132  2e-29

  Streptococcus pneumoniae SP14-BS69 [firmicutes] taxid 406560
 ref|ZP_01827888.1| choline binding protein PcpA [Streptoco...     126  1e-27

  Clostridium thermocellum ATCC 27405 [firmicutes] taxid 203119
 ref|YP_001038304.1| cellulosome enzyme, dockerin type I [C...     126  2e-27

  Streptococcus equi subsp. zooepidemicus MGCS10565 [firmicutes] taxid 552526
 ref|YP_002123438.1| cell surface protein, RBC-binding prot...     123  9e-27
 ref|YP_002122760.1| putative Fe3+-siderophore transport pr...      78  4e-13

  candidate division TM7 single-cell isolate TM7b [bacteria] taxid 447455
 ref|ZP_02520161.1| Probable cell surface protein (Leucine-...     121  4e-26

  Clostridium thermocellum DSM 4150 [firmicutes] taxid 492476
 ref|ZP_03151475.1| cellulosome protein dockerin type I [Cl...     118  3e-25

  Fusobacterium nucleatum subsp. nucleatum ATCC 25586 [fusobacteria] taxid 190304
 ref|NP_603116.1| putative cytoplasmic protein [Fusobacteri...     116  2e-24
 ref|NP_602643.1| surface antigen [Fusobacterium nucleatum ...     112  2e-23

  Bacteroides intestinalis DSM 17393 [CFB group bacteria] taxid 471870
 ref|ZP_03012554.1| hypothetical protein BACINT_00102 [Bact...     115  4e-24
 ref|ZP_03015102.1| hypothetical protein BACINT_02691 [Bact...      69  2e-10

  Fusobacterium nucleatum subsp. vincentii ATCC 49256 [fusobacteria] taxid 209882
 ref|ZP_00144289.1| Hypothetical Cytosolic Protein [Fusobac...     113  1e-23

  Ruminococcus gnavus ATCC 29149 [firmicutes] taxid 411470
 ref|ZP_02040342.1| hypothetical protein RUMGNA_01106 [Rumi...     111  5e-23
 ref|ZP_02040507.1| hypothetical protein RUMGNA_01271 [Rumi...      60  1e-07

  Anaerostipes caccae DSM 14662 [firmicutes] taxid 411490
 ref|ZP_02418641.1| hypothetical protein ANACAC_01224 [Anae...     102  3e-20
 ref|ZP_02420370.1| hypothetical protein ANACAC_02987 [Anae...      96  1e-18
 ref|ZP_02420369.1| hypothetical protein ANACAC_02986 [Anae...      83  2e-14
 ref|ZP_02419441.1| hypothetical protein ANACAC_02030 [Anae...      81  5e-14
 ref|ZP_02418024.1| hypothetical protein ANACAC_00591 [Anae...      68  3e-10
 ref|ZP_02418387.1| hypothetical protein ANACAC_00965 [Anae...      57  7e-07

  Lactobacillus plantarum WCFS1 [firmicutes] taxid 220668
 ref|NP_786420.1| cell surface protein (putative) [Lactobac...     101  3e-20
 ref|NP_786384.1| cell surface protein (putative) [Lactobac...      67  9e-10
 ref|NP_786170.1| cell surface protein precursor [Lactobaci...      57  9e-07

  Flavobacterium johnsoniae UW101 [CFB group bacteria] taxid 376686
 ref|YP_001196312.1| hypothetical protein Fjoh_3983 [Flavob...      93  1e-17

  Bacteroides capillosus ATCC 29799 [CFB group bacteria] taxid 411467
 ref|ZP_02035394.1| hypothetical protein BACCAP_00990 [Bact...      93  1e-17
 ref|ZP_02034978.1| hypothetical protein BACCAP_00569 [Bact...      91  3e-17

  Parabacteroides distasonis ATCC 8503 [CFB group bacteria] taxid 435591
 ref|YP_001303778.1| putative surface antigen BspA [Parabac...      93  2e-17

  Streptococcus pneumoniae SP9-BS68 [firmicutes] taxid 406558
 ref|ZP_01821787.1| choline binding protein PcpA [Streptoco...      91  6e-17
 ref|ZP_01821788.1| ornithine carbamoyltransferase [Strepto...      83  1e-14

  Faecalibacterium prausnitzii M21/2 [firmicutes] taxid 411485
 ref|ZP_02092261.1| hypothetical protein FAEPRAM212_02550 [...      90  1e-16

  Listeria monocytogenes FSL J1-175 [firmicutes] taxid 393118
 ref|ZP_02288082.1| cell wall surface anchor family protein...      90  1e-16
 ref|ZP_02286345.1| hypothetical protein LmonocFSL_03699 [L...      69  2e-10
 ref|ZP_02286349.1| hypothetical protein LmonocFSL_03759 [L...      58  3e-07
 ref|ZP_02286347.1| hypothetical protein LmonocFSL_03719 [L...      56  1e-06

  Listeria monocytogenes FSL J1-194 [firmicutes] taxid 393117
 ref|ZP_02278283.1| cell wall surface anchor family protein...      90  1e-16
 ref|ZP_02277696.1| hypothetical protein LmonFS_12524 [List...      75  4e-12
 ref|ZP_02277695.1| hypothetical protein LmonFS_12519 [List...      70  9e-11
 ref|ZP_02276434.1| lipoprotein, putative [Listeria monocyt...      68  4e-10
 ref|ZP_02276432.1| hypothetical protein LmonFS_02529 [List...      56  1e-06
 ref|ZP_02276428.1| hypothetical protein LmonFS_02509 [List...      55  3e-06

  Listeria monocytogenes FSL N1-017 [firmicutes] taxid 393123
 ref|ZP_01928583.1| hypothetical protein LMHG_00875 [Lister...      90  1e-16
 ref|ZP_01926433.1| conserved domain protein [Listeria mono...      62  3e-08
 ref|ZP_01926434.1| hypothetical protein LMHG_03012 [Lister...      60  1e-07

  Listeria monocytogenes str. 4b F2365 [firmicutes] taxid 265669
 ref|YP_015226.1| cell wall surface anchor family protein [...      89  2e-16
 ref|YP_013101.1| putative lipoprotein [Listeria monocytoge...      74  7e-12
 ref|YP_013100.1| hypothetical protein LMOf2365_0494 [Liste...      72  2e-11
 ref|YP_014648.1| hypothetical protein LMOf2365_2055 [Liste...      70  9e-11
 ref|YP_014649.1| hypothetical protein LMOf2365_2056 [Liste...      64  7e-09

  Listeria monocytogenes str. 4b H7858 [firmicutes] taxid 267410
 ref|ZP_00230071.1| cell wall surface anchor family protein...      89  2e-16
 ref|ZP_00231738.1| conserved domain protein [Listeria mono...      80  1e-13
 ref|ZP_00230316.1| probable membrane associated lipoprotei...      74  8e-12
 ref|ZP_00230315.1| conserved domain protein [Listeria mono...      71  3e-11
 ref|ZP_00230317.1| lipoprotein, putative [Listeria monocyt...      63  1e-08

  Listeria monocytogenes HPB2262 [firmicutes] taxid 401650
 ref|ZP_01945104.1| hypothetical protein LMSG_00080 [Lister...      89  2e-16
 ref|ZP_01943010.1| hypothetical protein LMSG_02923 [Lister...      75  3e-12
 ref|ZP_01943013.1| hypothetical protein LMSG_02913 [Lister...      67  9e-10
 ref|ZP_01943009.1| hypothetical protein LMSG_02922 [Lister...      65  4e-09

  Listeria monocytogenes FSL J2-064 [firmicutes] taxid 393122
 ref|ZP_02321344.1| cell wall surface anchor family protein...      89  2e-16
 ref|ZP_02319401.1| lipoprotein, putative [Listeria monocyt...      70  8e-11

  Listeria monocytogenes FSL N3-165 [firmicutes] taxid 393124
 ref|ZP_01929099.1| hypothetical protein LMIG_02847 [Lister...      86  1e-15
 ref|ZP_01929101.1| hypothetical protein LMIG_02839 [Lister...      70  1e-10

  Listeria monocytogenes str. 1/2a F6854 [firmicutes] taxid 267409
 ref|ZP_00232304.1| conserved domain protein [Listeria mono...      86  2e-15
 ref|ZP_00232305.1| conserved domain protein [Listeria mono...      68  5e-10

  Listeria monocytogenes F6900 [firmicutes] taxid 393128
 ref|ZP_01931881.1| conserved domain protein [Listeria mono...      86  2e-15
 ref|ZP_01931882.1| conserved domain protein [Listeria mono...      68  5e-10

  Listeria monocytogenes J0161 [firmicutes] taxid 393130
 ref|ZP_01935906.1| conserved domain protein [Listeria mono...      86  2e-15
 ref|ZP_01935907.1| conserved domain protein [Listeria mono...      68  5e-10

  Listeria monocytogenes J2818 [firmicutes] taxid 393131
 ref|ZP_01937302.1| hypothetical protein LMPG_02776 [Lister...      86  2e-15
 ref|ZP_01937303.1| hypothetical protein LMPG_02777 [Lister...      68  5e-10

  Lactococcus lactis subsp. cremoris SK11 [firmicutes] taxid 272622
 ref|YP_809644.1| subtilisin-like serine protease [Lactococ...      85  2e-15

  Lactococcus lactis subsp. cremoris MG1363 [firmicutes] taxid 416870
 ref|YP_001032204.1| putative secreted protein [Lactococcus...      85  4e-15

  Listeria monocytogenes FSL R2-503 [firmicutes] taxid 393125
 ref|ZP_02285626.1| cell wall surface anchor family protein...      85  4e-15

  Anaerotruncus colihominis DSM 17241 [firmicutes] taxid 445972
 ref|ZP_02443492.1| hypothetical protein ANACOL_02805 [Anae...      84  8e-15

  Bacteroides uniformis ATCC 8492 [CFB group bacteria] taxid 411479
 ref|ZP_02070664.1| hypothetical protein BACUNI_02088 [Bact...      83  9e-15

  Alkaliphilus metalliredigens QYMF [firmicutes] taxid 293826
 ref|YP_001319604.1| cell wall/surface repeat-containing pr...      81  4e-14

  Enterococcus faecalis V583 [firmicutes] taxid 226185
 ref|NP_815907.1| hypothetical protein EF2248 [Enterococcus...      80  1e-13

  Anabaena variabilis ATCC 29413 [cyanobacteria] taxid 240292
 ref|YP_322011.1| small GTP-binding protein domain-containi...      75  2e-12

  Blastopirellula marina DSM 3645 [planctomycetes] taxid 314230
 ref|ZP_01089912.1| serine/threonine protein kinase [Blasto...      74  6e-12

  Lactobacillus acidophilus NCFM [firmicutes] taxid 272621
 ref|YP_194583.1| hypothetical protein LBA1740 [Lactobacill...      71  5e-11

  Clostridium novyi NT [firmicutes] taxid 386415
 ref|YP_877540.1| hypothetical protein NT01CX_1459 [Clostri...      71  7e-11

  Lactococcus lactis subsp. lactis Il1403 [firmicutes] taxid 272623
 ref|NP_267773.1| hypothetical protein L58460 [Lactococcus ...      70  1e-10

  Listeria monocytogenes EGD-e [firmicutes] taxid 169963
 ref|NP_463989.1| putative membrane associated lipoprotein ...      68  4e-10

  Mycoplasma arthritidis 158L3-1 [mycoplasmas] taxid 243272
 ref|YP_001999737.1| hypothetical lipoprotein [Mycoplasma a...      67  7e-10

  Nostoc punctiforme PCC 73102 [cyanobacteria] taxid 63737
 ref|YP_001864880.1| small GTP-binding protein [Nostoc punc...      67  1e-09

  Mycoplasma hyopneumoniae J [mycoplasmas] taxid 262719
 ref|YP_278926.1| hypothetical protein MHJ_0123 [Mycoplasma...      65  3e-09
 ref|YP_279337.1| hypothetical protein MHJ_0540 [Mycoplasma...      63  2e-08

  Mycoplasma hyopneumoniae 7448 [mycoplasmas] taxid 262722
 ref|YP_287525.1| hypothetical protein MHP7448_0127 [Mycopl...      65  3e-09
 ref|YP_287927.1| hypothetical protein MHP7448_0538 [Mycopl...      63  2e-08

  Mycoplasma mobile 163K [mycoplasmas] taxid 267748
 ref|YP_015833.1| hypothetical protein MMOB1360 [Mycoplasma...      64  6e-09

  Mycoplasma hyopneumoniae 232 [mycoplasmas] taxid 295358
 ref|YP_115767.1| hypothetical protein mhp254 [Mycoplasma h...      64  7e-09
 ref|YP_116064.1| hypothetical protein mhp555 [Mycoplasma h...      60  1e-07

  Microscilla marina ATCC 23134 [CFB group bacteria] taxid 313606
 ref|ZP_01688907.1| leucine-rich repeat containing protein ...      63  2e-08

  Desulfotomaculum reducens MI-1 [firmicutes] taxid 349161
 ref|YP_001112744.1| hypothetical protein Dred_1389 [Desulf...      62  3e-08

  Mycoplasma pulmonis UAB CTIP [mycoplasmas] taxid 272635
 ref|NP_326042.1| hypothetical protein MYPU_2110 [Mycoplasm...      59  3e-07

  Dorea longicatena DSM 13814 [firmicutes] taxid 411462
 ref|ZP_01995697.1| hypothetical protein DORLON_01692 [Dore...      58  4e-07

  Vibrio cholerae V52 [g-proteobacteria] taxid 345076
 ref|ZP_01682698.1| bifunctional GlmU protein [Vibrio chole...      56  1e-06

  Clostridium bolteae ATCC BAA-613 [firmicutes] taxid 411902
 ref|ZP_02082525.1| hypothetical protein CLOBOL_00037 [Clos...      56  2e-06

  Yersinia pseudotuberculosis IP 31758 [enterobacteria] taxid 349747
 ref|YP_001401893.1| hypothetical protein YpsIP31758_2931 [...      55  4e-06
```

---

**Taxonomy Report**

```
Bacteria .........................................................  2202 hits  147 orgs [root; cellular organisms]
. Firmicutes .....................................................  1509 hits  102 orgs 
. . Clostridiales ................................................  1070 hits   37 orgs [Clostridia]
. . . Clostridiaceae .............................................   435 hits   20 orgs 
. . . . Clostridium ..............................................   427 hits   19 orgs 
. . . . . Clostridium leptum DSM 753 .............................     5 hits    1 orgs [Clostridium leptum]
. . . . . Clostridium sp. L2-50 ..................................    77 hits    1 orgs 
. . . . . Clostridium phytofermentans ISDg .......................    41 hits    1 orgs [Clostridium phytofermentans]
. . . . . Clostridium beijerinckii NCIMB 8052 ....................    55 hits    1 orgs [Clostridium beijerinckii]
. . . . . Clostridium scindens ATCC 35704 ........................    26 hits    1 orgs [Clostridium scindens]
. . . . . Clostridium sporogenes ATCC 15579 ......................    15 hits    1 orgs [Clostridium sporogenes]
. . . . . Clostridium botulinum ..................................   103 hits    6 orgs 
. . . . . . Clostridium botulinum F str. Langeland ...............    18 hits    1 orgs [Clostridium botulinum F]
. . . . . . Clostridium botulinum A ..............................    34 hits    2 orgs 
. . . . . . . Clostridium botulinum A3 str. Loch Maree ...........    16 hits    1 orgs 
. . . . . . . Clostridium botulinum A str. ATCC 3502 .............    18 hits    1 orgs 
. . . . . . Clostridium botulinum NCTC 2916 ......................    17 hits    1 orgs 
. . . . . . Clostridium botulinum B1 str. Okra ...................    16 hits    1 orgs [Clostridium botulinum B]
. . . . . . Clostridium botulinum Bf .............................    18 hits    1 orgs 
. . . . . Clostridium butyricum 5521 .............................    14 hits    1 orgs [Clostridium butyricum]
. . . . . Clostridium bartlettii DSM 16795 .......................    28 hits    1 orgs [Clostridium bartlettii]
. . . . . Clostridium sp. SS2/1 ..................................    52 hits    1 orgs 
. . . . . Clostridium thermocellum ...............................     6 hits    2 orgs 
. . . . . . Clostridium thermocellum ATCC 27405 ..................     3 hits    1 orgs 
. . . . . . Clostridium thermocellum DSM 4150 ....................     3 hits    1 orgs 
. . . . . Clostridium novyi NT ...................................     3 hits    1 orgs [Clostridium novyi]
. . . . . Clostridium bolteae ATCC BAA-613 .......................     2 hits    1 orgs [Clostridium bolteae]
. . . . Alkaliphilus metalliredigens QYMF ........................     8 hits    1 orgs [Alkaliphilus; Alkaliphilus metalliredigens]
. . . Eubacteriaceae .............................................   214 hits    3 orgs 
. . . . Eubacterium ..............................................   123 hits    2 orgs 
. . . . . Eubacterium siraeum DSM 15702 ..........................    48 hits    1 orgs [Eubacterium siraeum]
. . . . . Eubacterium ventriosum ATCC 27560 ......................    75 hits    1 orgs [Eubacterium ventriosum]
. . . . Anaerofustis stercorihominis DSM 17244 ...................    91 hits    1 orgs [Anaerofustis; Anaerofustis stercorihominis]
. . . Epulopiscium sp. 'N.t. morphotype B' .......................   189 hits    1 orgs [unclassified Clostridiales; Epulopiscium]
. . . Syntrophomonas wolfei subsp. wolfei str. Goettingen ........     4 hits    1 orgs [Syntrophomonadaceae; Syntrophomonas; Syntrophomonas wolfei; Syntrophomonas wolfei subsp. wolfei]
. . . Ruminococcaceae ............................................   124 hits    5 orgs 
. . . . Ruminococcus .............................................   101 hits    3 orgs 
. . . . . Ruminococcus torques ATCC 27756 ........................    43 hits    1 orgs [Ruminococcus torques]
. . . . . Ruminococcus obeum ATCC 29174 ..........................    35 hits    1 orgs [Ruminococcus obeum]
. . . . . Ruminococcus gnavus ATCC 29149 .........................    23 hits    1 orgs [Ruminococcus gnavus]
. . . . Faecalibacterium prausnitzii M21/2 .......................    12 hits    1 orgs [Faecalibacterium; Faecalibacterium prausnitzii]
. . . . Anaerotruncus colihominis DSM 17241 ......................    11 hits    1 orgs [Anaerotruncus; Anaerotruncus colihominis]
. . . Lachnospiraceae ............................................    77 hits    4 orgs 
. . . . Coprococcus eutactus ATCC 27759 ..........................    36 hits    1 orgs [Coprococcus; Coprococcus eutactus]
. . . . Dorea ....................................................    12 hits    2 orgs 
. . . . . Dorea formicigenerans ATCC 27755 .......................     8 hits    1 orgs [Dorea formicigenerans]
. . . . . Dorea longicatena DSM 13814 ............................     4 hits    1 orgs [Dorea longicatena]
. . . . Anaerostipes caccae DSM 14662 ............................    29 hits    1 orgs [Anaerostipes; Anaerostipes caccae]
. . . Peptococcaceae .............................................    15 hits    2 orgs 
. . . . Desulfitobacterium hafniense Y51 .........................    12 hits    1 orgs [Desulfitobacterium; Desulfitobacterium hafniense]
. . . . Desulfotomaculum reducens MI-1 ...........................     3 hits    1 orgs [Desulfotomaculum; Desulfotomaculum reducens]
. . . Finegoldia magna ATCC 29328 ................................    12 hits    1 orgs [Clostridiales incertae sedis; Clostridiales Family XI. Incertae Sedis; Finegoldia; Finegoldia magna]
. . Clostridium spiroforme DSM 1552 ..............................     5 hits    1 orgs [Erysipelotrichi; Erysipelotrichales; Erysipelotrichaceae; unclassified Erysipelotrichaceae; Clostridium spiroforme]
. . Bacilli ......................................................   434 hits   64 orgs 
. . . Lactobacillales ............................................   324 hits   50 orgs 
. . . . Lactobacillus ............................................    12 hits    4 orgs [Lactobacillaceae]
. . . . . Lactobacillus casei ....................................     4 hits    2 orgs 
. . . . . . Lactobacillus casei BL23 .............................     2 hits    1 orgs 
. . . . . . Lactobacillus casei ATCC 334 .........................     2 hits    1 orgs 
. . . . . Lactobacillus plantarum WCFS1 ..........................     7 hits    1 orgs [Lactobacillus plantarum]
. . . . . Lactobacillus acidophilus NCFM .........................     1 hits    1 orgs [Lactobacillus acidophilus]
. . . . Streptococcaceae .........................................   305 hits   44 orgs 
. . . . . Streptococcus ..........................................   290 hits   41 orgs 
. . . . . . Streptococcus pyogenes ...............................   110 hits   13 orgs 
. . . . . . . Streptococcus pyogenes MGAS6180 ....................     8 hits    1 orgs [Streptococcus pyogenes serotype M28]
. . . . . . . Streptococcus pyogenes serotype M3 .................    16 hits    2 orgs 
. . . . . . . . Streptococcus pyogenes SSI-1 .....................     8 hits    1 orgs 
. . . . . . . . Streptococcus pyogenes MGAS315 ...................     8 hits    1 orgs 
. . . . . . . Streptococcus pyogenes MGAS10750 ...................    16 hits    1 orgs [Streptococcus pyogenes serotype M4]
. . . . . . . Streptococcus pyogenes MGAS10270 ...................     8 hits    1 orgs [Streptococcus pyogenes serotype M2]
. . . . . . . Streptococcus pyogenes serotype M12 ................    16 hits    2 orgs 
. . . . . . . . Streptococcus pyogenes MGAS9429 ..................     8 hits    1 orgs 
. . . . . . . . Streptococcus pyogenes MGAS2096 ..................     8 hits    1 orgs 
. . . . . . . Streptococcus pyogenes serotype M1 .................    14 hits    2 orgs 
. . . . . . . . Streptococcus pyogenes M1 GAS ....................     7 hits    1 orgs 
. . . . . . . . Streptococcus pyogenes MGAS5005 ..................     7 hits    1 orgs 
. . . . . . . Streptococcus pyogenes str. Manfredo ...............     8 hits    1 orgs [Streptococcus pyogenes serotype M5]
. . . . . . . Streptococcus pyogenes MGAS8232 ....................     8 hits    1 orgs [Streptococcus pyogenes serotype M18]
. . . . . . . Streptococcus pyogenes M49 591 .....................     8 hits    1 orgs [Streptococcus pyogenes serotype M49]
. . . . . . . Streptococcus pyogenes MGAS10394 ...................     8 hits    1 orgs [Streptococcus pyogenes serotype M6]
. . . . . . Streptococcus agalactiae .............................    69 hits    8 orgs 
. . . . . . . Streptococcus agalactiae COH1 ......................     8 hits    1 orgs 
. . . . . . . Streptococcus agalactiae 2603V/R ...................     8 hits    1 orgs [Streptococcus agalactiae serogroup V]
. . . . . . . Streptococcus agalactiae 18RS21 ....................     8 hits    1 orgs 
. . . . . . . Streptococcus agalactiae H36B ......................     8 hits    1 orgs 
. . . . . . . Streptococcus agalactiae NEM316 ....................     8 hits    1 orgs [Streptococcus agalactiae serogroup III]
. . . . . . . Streptococcus agalactiae A909 ......................     8 hits    1 orgs [Streptococcus agalactiae serogroup Ia]
. . . . . . . Streptococcus agalactiae 515 .......................     9 hits    1 orgs 
. . . . . . . Streptococcus agalactiae CJB111 ....................    12 hits    1 orgs 
. . . . . . Streptococcus pneumoniae .............................    94 hits   19 orgs 
. . . . . . . Streptococcus pneumoniae SP6-BS73 ..................     4 hits    1 orgs 
. . . . . . . Streptococcus pneumoniae CDC0288-04 ................     4 hits    1 orgs 
. . . . . . . Streptococcus pneumoniae CDC3059-06 ................     4 hits    1 orgs 
. . . . . . . Streptococcus pneumoniae CGSP14 ....................     4 hits    1 orgs 
. . . . . . . Streptococcus pneumoniae SP195 .....................     4 hits    1 orgs 
. . . . . . . Streptococcus pneumoniae SP11-BS70 .................     4 hits    1 orgs 
. . . . . . . Streptococcus pneumoniae MLV-016 ...................     4 hits    1 orgs 
. . . . . . . Streptococcus pneumoniae SP19-BS75 .................     4 hits    1 orgs 
. . . . . . . Streptococcus pneumoniae CDC1873-00 ................     4 hits    1 orgs 
. . . . . . . Streptococcus pneumoniae CDC1087-00 ................     4 hits    1 orgs 
. . . . . . . Streptococcus pneumoniae Hungary19A-6 ..............     4 hits    1 orgs 
. . . . . . . Streptococcus pneumoniae TIGR4 .....................     8 hits    1 orgs 
. . . . . . . Streptococcus pneumoniae SP18-BS74 .................     4 hits    1 orgs 
. . . . . . . Streptococcus pneumoniae G54 .......................     5 hits    1 orgs 
. . . . . . . Streptococcus pneumoniae R6 ........................     5 hits    1 orgs 
. . . . . . . Streptococcus pneumoniae D39 .......................     4 hits    1 orgs 
. . . . . . . Streptococcus pneumoniae SP23-BS72 .................     3 hits    1 orgs 
. . . . . . . Streptococcus pneumoniae SP14-BS69 .................     7 hits    1 orgs 
. . . . . . . Streptococcus pneumoniae SP9-BS68 ..................    14 hits    1 orgs 
. . . . . . Streptococcus equi subsp. zooepidemicus MGCS10565 ....    17 hits    1 orgs [Streptococcus dysgalactiae group; Streptococcus equi; Streptococcus equi subsp. zooepidemicus]
. . . . . Lactococcus lactis .....................................    15 hits    3 orgs [Lactococcus]
. . . . . . Lactococcus lactis subsp. cremoris ...................    12 hits    2 orgs 
. . . . . . . Lactococcus lactis subsp. cremoris SK11 ............     6 hits    1 orgs 
. . . . . . . Lactococcus lactis subsp. cremoris MG1363 ..........     6 hits    1 orgs 
. . . . . . Lactococcus lactis subsp. lactis Il1403 ..............     3 hits    1 orgs [Lactococcus lactis subsp. lactis]
. . . . Leuconostoc mesenteroides subsp. mesenteroides ATCC 8293 .     4 hits    1 orgs [Leuconostocaceae; Leuconostoc; Leuconostoc mesenteroides; Leuconostoc mesenteroides subsp. mesenteroides]
. . . . Enterococcus faecalis V583 ...............................     3 hits    1 orgs [Enterococcaceae; Enterococcus; Enterococcus faecalis]
. . . Listeria monocytogenes .....................................   110 hits   14 orgs [Bacillales; Listeriaceae; Listeria]
. . . . Listeria monocytogenes FSL J1-175 ........................    10 hits    1 orgs 
. . . . Listeria monocytogenes FSL J1-194 ........................    15 hits    1 orgs 
. . . . Listeria monocytogenes FSL N1-017 ........................     6 hits    1 orgs 
. . . . Listeria monocytogenes str. 4b F2365 .....................    13 hits    1 orgs 
. . . . Listeria monocytogenes str. 4b H7858 .....................    14 hits    1 orgs 
. . . . Listeria monocytogenes HPB2262 ...........................    12 hits    1 orgs 
. . . . Listeria monocytogenes FSL J2-064 ........................     8 hits    1 orgs 
. . . . Listeria monocytogenes FSL N3-165 ........................     5 hits    1 orgs 
. . . . Listeria monocytogenes str. 1/2a F6854 ...................     5 hits    1 orgs 
. . . . Listeria monocytogenes F6900 .............................     5 hits    1 orgs 
. . . . Listeria monocytogenes J0161 .............................     5 hits    1 orgs 
. . . . Listeria monocytogenes J2818 .............................     5 hits    1 orgs 
. . . . Listeria monocytogenes FSL R2-503 ........................     5 hits    1 orgs 
. . . . Listeria monocytogenes EGD-e .............................     2 hits    1 orgs 
. Chlamydiae/Verrucomicrobia group ...............................    42 hits    2 orgs 
. . bacterium Ellin514 ...........................................    11 hits    1 orgs [Verrucomicrobia; Verrucomicrobiae; Verrucomicrobiales; Verrucomicrobia subdivision 3]
. . Victivallis vadensis ATCC BAA-548 ............................    31 hits    1 orgs [Lentisphaerae; Victivallales; Victivallaceae; Victivallis; Victivallis vadensis]
. Bacteroidetes ..................................................   407 hits   18 orgs [Bacteroidetes/Chlorobi group]
. . Flavobacteriales .............................................   108 hits    4 orgs [Flavobacteria]
. . . Flavobacteriaceae ..........................................   100 hits    3 orgs 
. . . . Flavobacterium ...........................................    89 hits    2 orgs 
. . . . . Flavobacterium psychrophilum JIP02/86 ..................    85 hits    1 orgs [Flavobacterium psychrophilum]
. . . . . Flavobacterium johnsoniae UW101 ........................     4 hits    1 orgs [Flavobacterium johnsoniae]
. . . . Kordia algicida OT-1 .....................................    11 hits    1 orgs [Kordia; Kordia algicida]
. . . Flavobacteriales bacterium ALC-1 ...........................     8 hits    1 orgs [unclassified Flavobacteriales; unclassified Flavobacteriales (miscellaneous)]
. . Bacteroidales ................................................   298 hits   13 orgs [Bacteroidetes (class)]
. . . Alistipes putredinis DSM 17216 .............................    61 hits    1 orgs [Rikenellaceae; Alistipes; Alistipes putredinis]
. . . Bacteroides ................................................   227 hits   11 orgs [Bacteroidaceae]
. . . . Bacteroides fragilis .....................................    34 hits    2 orgs 
. . . . . Bacteroides fragilis NCTC 9343 .........................    20 hits    1 orgs 
. . . . . Bacteroides fragilis YCH46 .............................    14 hits    1 orgs 
. . . . Bacteroides ovatus ATCC 8483 .............................    38 hits    1 orgs [Bacteroides ovatus]
. . . . Bacteroides stercoris ATCC 43183 .........................    42 hits    1 orgs [Bacteroides stercoris]
. . . . Bacteroides thetaiotaomicron VPI-5482 ....................    30 hits    1 orgs [Bacteroides thetaiotaomicron]
. . . . Bacteroides caccae ATCC 43185 ............................    12 hits    1 orgs [Bacteroides caccae]
. . . . Bacteroides vulgatus ATCC 8482 ...........................    13 hits    1 orgs [Bacteroides vulgatus]
. . . . Bacteroides coprocola DSM 17136 ..........................    11 hits    1 orgs [Bacteroides coprocola]
. . . . Bacteroides intestinalis DSM 17393 .......................    14 hits    1 orgs [Bacteroides intestinalis]
. . . . Bacteroides capillosus ATCC 29799 ........................    27 hits    1 orgs [Bacteroides capillosus]
. . . . Bacteroides uniformis ATCC 8492 ..........................     6 hits    1 orgs [Bacteroides uniformis]
. . . Parabacteroides distasonis ATCC 8503 .......................    10 hits    1 orgs [Porphyromonadaceae; Parabacteroides; Parabacteroides distasonis]
. . Microscilla marina ATCC 23134 ................................     1 hits    1 orgs [Sphingobacteria; Sphingobacteriales; Flexibacteraceae; Microscilla; Microscilla marina]
. Treponema ......................................................    83 hits    3 orgs [Spirochaetes; Spirochaetes (class); Spirochaetales; Spirochaetaceae]
. . Treponema denticola ATCC 35405 ...............................    65 hits    1 orgs [Treponema denticola]
. . Treponema pallidum subsp. pallidum ...........................    18 hits    2 orgs [Treponema pallidum]
. . . Treponema pallidum subsp. pallidum str. Nichols ............     9 hits    1 orgs 
. . . Treponema pallidum subsp. pallidum SS14 ....................     9 hits    1 orgs 
. Cyanobacteria ..................................................    20 hits    3 orgs 
. . Synechococcus sp. WH 7805 ....................................    17 hits    1 orgs [Chroococcales; Synechococcus]
. . Nostocaceae ..................................................     3 hits    2 orgs [Nostocales]
. . . Anabaena variabilis ATCC 29413 .............................     1 hits    1 orgs [Anabaena; Anabaena variabilis]
. . . Nostoc punctiforme PCC 73102 ...............................     2 hits    1 orgs [Nostoc; Nostoc punctiforme]
. Gammaproteobacteria ............................................    41 hits    6 orgs [Proteobacteria]
. . Shewanella ...................................................    26 hits    2 orgs [Alteromonadales; Shewanellaceae]
. . . Shewanella pealeana ATCC 700345 ............................    17 hits    1 orgs [Shewanella pealeana]
. . . Shewanella halifaxensis HAW-EB4 ............................     9 hits    1 orgs [Shewanella halifaxensis]
. . Vibrionaceae .................................................    10 hits    2 orgs [Vibrionales]
. . . Photobacterium sp. SKA34 ...................................     8 hits    1 orgs [Photobacterium]
. . . Vibrio cholerae V52 ........................................     2 hits    1 orgs [Vibrio; Vibrio cholerae]
. . Enterobacteriaceae ...........................................     5 hits    2 orgs [Enterobacteriales]
. . . Escherichia coli E24377A ...................................     4 hits    1 orgs [Escherichia; Escherichia coli]
. . . Yersinia pseudotuberculosis IP 31758 .......................     1 hits    1 orgs [Yersinia; Yersinia pseudotuberculosis]
. Actinomyces odontolyticus ATCC 17982 ...........................    11 hits    1 orgs [Actinobacteria; Actinobacteria (class); Actinobacteridae; Actinomycetales; Actinomycineae; Actinomycetaceae; Actinomyces; Actinomyces odontolyticus]
. Mollicutes .....................................................    63 hits    8 orgs [Tenericutes]
. . Mycoplasma ...................................................    52 hits    7 orgs [Mycoplasmatales; Mycoplasmataceae]
. . . Mycoplasma agalactiae PG2 ..................................    11 hits    1 orgs [Mycoplasma agalactiae]
. . . Mycoplasma arthritidis 158L3-1 .............................     4 hits    1 orgs [Mycoplasma arthritidis]
. . . Mycoplasma hyopneumoniae ...................................    26 hits    3 orgs 
. . . . Mycoplasma hyopneumoniae J ...............................     9 hits    1 orgs 
. . . . Mycoplasma hyopneumoniae 7448 ............................     9 hits    1 orgs 
. . . . Mycoplasma hyopneumoniae 232 .............................     8 hits    1 orgs 
. . . Mycoplasma mobile 163K .....................................     4 hits    1 orgs [Mycoplasma mobile]
. . . Mycoplasma pulmonis UAB CTIP ...............................     7 hits    1 orgs [Mycoplasma pulmonis]
. . Acholeplasma laidlawii PG-8A .................................    11 hits    1 orgs [Acholeplasmatales; Acholeplasmataceae; Acholeplasma; Acholeplasma laidlawii]
. candidate division TM7 single-cell isolate TM7b ................    11 hits    1 orgs [unclassified Bacteria; candidate division TM7]
. Fusobacterium nucleatum ........................................    13 hits    2 orgs [Fusobacteria; Fusobacteria (class); Fusobacteriales; Fusobacteriaceae; Fusobacterium]
. . Fusobacterium nucleatum subsp. nucleatum ATCC 25586 ..........     9 hits    1 orgs [Fusobacterium nucleatum subsp. nucleatum]
. . Fusobacterium nucleatum subsp. vincentii ATCC 49256 ..........     4 hits    1 orgs [Fusobacterium nucleatum subsp. vincentii]
. Blastopirellula marina DSM 3645 ................................     2 hits    1 orgs [Planctomycetes; Planctomycetacia; Planctomycetales; Planctomycetaceae; Blastopirellula; Blastopirellula marina]
```

---


### 2) Archaea

**Lineage Report**  

```
Euryarchaeota  [euryarchaeotes]
. Methanosarcina [euryarchaeotes]
. . Methanosarcina acetivorans C2A -----  436 38 hits [euryarchaeotes]  cell surface protein [Methanosarcina acetivorans C2A]
. . Methanosarcina barkeri str. Fusaro .  429 27 hits [euryarchaeotes]  cell surface protein [Methanosarcina barkeri str. Fusaro]
. Methanococcus vannielii SB -----------  251  8 hits [euryarchaeotes]  cell surface protein [Methanococcus vannielii SB]
. Methanococcus maripaludis C7 .........  250 14 hits [euryarchaeotes]  TPR repeat-containing protein [Methanococcus maripaludis C7]
. Methanococcus maripaludis C6 .........  135 16 hits [euryarchaeotes]  hypothetical protein MmarC6_0234 [Methanococcus maripaludis
```

---

**Organism Report**

```
  Methanosarcina acetivorans C2A [euryarchaeotes] taxid 188937
 ref|NP_619153.1| cell surface protein [Methanosarcina acet...     436  3e-122
 ref|NP_619156.1| cell surface protein [Methanosarcina acet...     392  7e-109
 ref|NP_619149.1| cell surface protein [Methanosarcina acet...     303  2e-82

  Methanosarcina barkeri str. Fusaro [euryarchaeotes] taxid 269797
 ref|YP_305509.1| cell surface protein [Methanosarcina bark...     429  4e-120
 ref|YP_305504.1| cell surface protein [Methanosarcina bark...     299  5e-81

  Methanococcus vannielii SB [euryarchaeotes] taxid 406327
 ref|YP_001322799.1| cell surface protein [Methanococcus va...     251  1e-66
 ref|YP_001322745.1| hypothetical protein Mevan_0223 [Metha...      49  8e-06

  Methanococcus maripaludis C7 [euryarchaeotes] taxid 426368
 ref|YP_001330886.1| TPR repeat-containing protein [Methano...     250  3e-66
 ref|YP_001329237.1| hypothetical protein MmarC7_0013 [Meth...     145  1e-34

  Methanococcus maripaludis C6 [euryarchaeotes] taxid 444158
 ref|YP_001548287.1| hypothetical protein MmarC6_0234 [Meth...     135  1e-31
 ref|YP_001548288.1| hypothetical protein MmarC6_0235 [Meth...     115  2e-25
```

---

**Taxonomy Report**

```
Euryarchaeota ..........................   103 hits    5 orgs [root; cellular organisms; Archaea]
. Methanosarcina .......................    65 hits    2 orgs [Methanomicrobia; Methanosarcinales; Methanosarcinaceae]
. . Methanosarcina acetivorans C2A .....    38 hits    1 orgs [Methanosarcina acetivorans]
. . Methanosarcina barkeri str. Fusaro .    27 hits    1 orgs [Methanosarcina barkeri]
. Methanococcus ........................    38 hits    3 orgs [Methanococci; Methanococcales; Methanococcaceae]
. . Methanococcus vannielii SB .........     8 hits    1 orgs [Methanococcus vannielii]
. . Methanococcus maripaludis ..........    30 hits    2 orgs 
. . . Methanococcus maripaludis C7 .....    14 hits    1 orgs 
. . . Methanococcus maripaludis C6 .....    16 hits    1 orgs
```
