# Supplementary material for: Trichomonas vaginalis vast BspA-like gene family: evidence for functional diversity from structural organisation and transcriptomics
Source: BMC Genomics. 2010 Feb 8;11:99. doi: 10.1186/1471-2164-11-99 (PMC2843621; doi:10.1186/1471-2164-11-99)
Supplement: Additional file 11 — Supplemental Figure S4. Alignment of TpLRR and other repeats for selected TvBspA proteins. Combination of manual and SAPS based alignment of repeats for TpLRR, and other repeats when present. Included selected TvBspA proteins with well conserved and less well conserved TpLRR illustrating their diversity. [file 1471-2164-11-99-S11.PDF]

>TvBspA515 --- TVAG_244780 (not TpLRR positive: TpLRR 1-177 in BlastP)

```
MGTSVFIHCNNIESITLPRSLTSSDGRTFNGCSIKKVIVQLPEIFPYYISLFAESSIDLLIFQTPISDVVFGDRE
SMKNAAKTIIFDLPKPDSSTSELKAFEELIDLTFIHIKNINNHPIPYNFVIADNVPIYVERNGININETAFSKSN
IFNFTYLGKDPIPGDFLVNARSCEHVYVTRFYTSDKLGGLYVTVLDDLEPTPEP
EETSSSSSTSS
EETTSSSSSSSS
EETSSSSSTSS
EETSSSSSSSSS
EETSSSSSSSS
EETSSSSSSSSS
EETTSSSSSSS
EETTSSSSSSS
EETTSSSSSSSE
EETTSSSSSSSSPS
EETTSSSSTSSS
TEKEETPIIDPKTPIPPPVNSSAASQNNTVEESSTNSSSGNSDPNNSNNGGSSNSAGNTAGIVCGVVLIVAMIVV
VIFVVYKMKCAKDGKPDVEVGDQNTILNQKVDLMVKQKLDQLVKQKVDKIVSEKVDEIVTSRLEDVESDNYDYES
HDLNYSDEEGHTNEDFILPDKNNDSDNIIQL
```

Note: This sequence has one tandem repeat rich in serine – alignment highlighted in purple - in addition to a divergent TpLRR

>TvBspA575 --- TVAG_244800 (not TpLRR positive: TpLRR 1-177 in BlastP)

```
LxxIxLxxxVxxIgxxAFxxCxx
MGIGVFKHCNNIESITLPRSLTSSDAFVFYECSNIKQVIVQVPENFVSQMSVFVGSSIDLLIFQTPISDIVFGDR
ESMKNAAKTIIFDLPKPDSSTSELKAFKDLINLTFIHIKNINNHPIPYNFVTADNVSILVEQNGISINETAFSEA
NIFNFTYLGKDPIPGDFLVNARSCEHVYVTRSYTSDKLGGLYVTVLDDLEPTPEP
EETSSSSSSSS
EETTSSSSSSS
EETTSSSSSSS
EETTSSSSSSS
EETTSSSSSSS
EETTSSSSSSS
EETTSSSSSSS
EETTSSSSSSS
EETTSSSSSSSS
EETTSYSSSPS
EETTSSSSSSPS
EETTSSSSSSSSPS
EETTSSSSSPS
EETTSSSSSSSSPS
EETTSSSSSSSSPS
EETTSSSSTSSST
EKEETPIIDPKTPIPPPVNSSAASQNNTVEESSTNSSSGNSDPNNSNNGGSSNSAGNTAGIVCGVVLIVAMIVVV
IFVVYKMKCAKDGKPDVEVGDQNTILNQKVDLMVKQKLDQLVKQKVDKIVSEKVDEIVTSRLEDVESDNYDYESH
DLNYSDEEGHTNEDFILPDKNNDSDNIIQL
```

Note: This sequence has one tandem repeat rich in serine – alignment highlighted in purple - in addition to a divergent TpLRR

>TvBspA605 --- TVAG_244930 (TpLRR positive)

```
LxxIxLxxxVxxIgxxAFxxCxx
MISLLFLFISSKDSYKIDVVNNVAQLSASKGAVYTGEITIPSKVDHNSKSYVVGILRKSLFENST
ITSITLPNSITEIKQNCFANCKK
LKTVNLKA---------------
-------LKLNSIRARTFCSCDS
LVAVILPTTVKTIEDYSFYNCYN
LEAID------------------
----TENIPIVSIGNYSFTNCRK
LTEIVVNNK--------------
--------KFSYLGDYCFANTSL
TSFNF-QDNLTHLGIGAFSHTK-
```

LRNFEISDTL-------------
---------LTEIADHAFEYCQ-
ITELLLPPSINSIGESAFEGNSG
IKNVDISYT--------------
--------CVYTIKSAAFKNCHS
LSSFNPSILTQYIGDFVFEATN-
LSEFTVSESYRHFGSYCFKSCQN
LIKANFY---------------
------EMMFDNIPEGLFYNCTK
FQVIQFARRYFEIKAHAFEF-TA
IEHIRFPNSIIGIGDYAFAHCHS
MLTVDISN---------------
-------IDYSLTGTHIFYDCPK
VNLVLFSEDDIVLPPYLLAGTS-
LEIFNPSRNILGIGEGCFMNCKK
LKEVNLLRTVMT-----------
-----------NISDYAFFGCNI
SKILLPPSISLIGYMALGNGTVNSVVYYGQNMPFFGYSTKFGSVTVNFGYQSDVFCGDKVRYEHLNQVGENGEKM
PEDQSLVGFFLKFIALVMLIIFVTYMVNKHGLEYYLGFFEHNNRRLIIG

Note: This protein has two putative TM domains.

>TvBspA625 --- TVAG_073760 (TpLRR positive, ~14 x LRR)

LxxIxLxxxVxxIgxxAFxxCxx
MMTPGKSSKTPIVGNLT
TLGITIPDTVTSIGFKTFYGCSS
FTSIIIPNSVTSIGTKAFTGCSS
LTSITIGNSVTSFGQEAFSECSS
ITSITIPNSVTTIRDFAFSGCSK
LTSITIPNSVTSLGSHAFRGCSG
LTSIIIPDSVTLIRGSIFYGCSS
LTSITIPNSVTSIYSSAFYGCSS
LTSITIPDSVLDFGSAAFQECSK
LTNIKIGNNVDSIGSLAFKRCSS
LTNITIPDSVTTIANSAFYECSK
LTSITIGKSVTRIEGNAFSKCYS
LTSITIK----------------
----TTNDITSSITTDVFLNCP-
ITELIYETTGLTFLTYEYFKDKVT
LIKFNIPKSDSNSMIR
LQEITSLPTLTHFTNLNKVTIEN
INELTIPESFIEGDN
FEIL-ITNNIKSIDPNAFKDCS-
INKFTYLGTDKLENDFLKNAKSCEEVITSTKYSDNEIGGMTITHKQSE
ENPNQPG
ENPNQPG
ENPNQPG
ENPNQPG
ENPSQPG
ENPSQPG
ENPNQPG
ENPSQPG
ENPNQPG
ENPNQPG
ENPNQPG
ENPSQPG
EN
TDDPSKSKENKAIKGGEIAGIIIGSLIGICLVVAICFGVYYYFMRIKPKNKNDDNEGNQEDTIANGTNEVTNENV
LATFDEQPNNESDSNGLDSAEV

Note: This sequence has one additional tandem repeat rich – alignment highlighted in purple

\>TvBspA681 --- TVAG_163950 (TpLRR positive)

LxxIxLxxxVxxIgxxAFxxCxx
MLFSLISLALSSCSD
SKTFIFISPYKGVTY-ANCTCM
LTANTRNSLPSDWTVPSTVNYNGD
--------AYTVIAIKDFSSYQ
FTSITFPDSIQRIDSKAFLANT-
IKRVTFGTGLQYLGSGAFKNAY-
LGTVILPNTFKLLYLSAFARATE
--------IIIPYGIQAFGDEPF
NNSNTLINIKGNYPSDS
----IQILNADKIYYYGTDDCLTITGT
VRKIYVTANYKTSTLGGKPATVCLQGDF
RFEAKTASPLTYAAAEYFGADRT
PS
VPATFNEGKVTQIGQNLFKDNTI
ISSFVIGKIILKIGDYAFSGCTN
LRRITFEES--------------
-------CKLIEIASYAFSGCAS
SS
FTSIVLPKTVNKIGANAFLGCIK
LTTVTLPSLITSIDPSCFSNCPL
-ETVNIIDSGDGYI-STYEGCST
LTGKVIIGDGITEIDSFGFKGTR
LxxIxLxxxVxxIgxxAFxxCxx
IKNVQFNS-VTTIKEGAFEQSSL
QCILILPATVTTVEVDAFSKCAD
-LSRVYYCGTSKFDQKAFNENSF
TKIYVLSTYPESNIFGLTSNKVL
NEQCQDPESADQCPSHEKPIIIEPDDETKSG
GGSTS
GGSTS
GGSTS
GGSTS
GGSTS
GGSTS
GGSSS
GGSTS
GGSTS
GGSTS
GGSSS
GGSTS
GGSTS
GGSTS
GGSTS
GGSSS
G
DEVSKDNKDGSLVKDAAESLNKLPVWAIVLICLGVVGVIVAVIIMALRSQITQFCGECNIIKGQAEV

Note: This sequence has one additional tandem repeat rich in glycine and serine (purple highlight) 16x GGS[TS]S

>TvBspA724 --- TVAG_158720 (TpLRR positive)

```
LxxIxLxxxVxxIgxxAFxxCxx
MNNFEVATFILGKLVFYGCTSLDIQKIGTNFNLISTDSQPFIRTG
ITTLTINTGVQYLFCNSPS----
LTTVVFSETS-------------
--------GLTFVPSHMFENCTS
LQSVTFNSGMTTIKRFAFANTA-
LTKLDLKN-VFILEPLSFYKSG-
VQEITFNKNVEVKNIWDTEYKDESDVKGEYDD
VYTIEQMNKGILSYLTQFMDVE-
ESDFRLIEKYSTHRFCVFLNESR
LTKITLGSSVTEFKSSMFANCS-
LTTFDVTGNSNFVFESGILYSKSK
IELICIAT-LA------------
ATEYTVPSSISIISPFAFTFVPT
ITKITVPSSVAKFNY-AFANMNR
LRNITFQGSVSFIPDGAFFCCHS
LKNIEIPSS--------------
-------STVTSAGDLSFAFCYS
LVNL-MTNSFEKIGRGCFICCQK
LSSVDC-SKLTILNEFTFYHCIG
NTTFTFGDNIKIISKNAFFM-SS
IESFTVPANVISIEQQAFSMCKE
LTTVTLNSKLVRIGKEAFAGSG-
VKNIQIPNSVRFIEQSAFDNCKN
VT-FSFETG--------------
------GHPLFDVDNNCFIHKAT
KNLLFTFGDTYHKFTVSEKVKEVLPNSLRFTTVQRERNEGGFTLFSGVNTLVIPEVVYDVQDNLTNNPLIDSVCL
DGISTFNLFFPIRNDMTVFITDDFVYGTYYNTIHVIHDMCKDTVDSRYDPRRYDRLDGIYEGNCSTSINDTYTSE
FSDPIYGGNFPFINQKQAKAVFEYAGVSPLTAFISIYVILITLALIFSALYIARIN
```

>TvBspA733 --- TVAG_301290 (TpLRR positive)

```
LxxIxLxxxVxxIgxxAFxxCxx
MHEASINSILSCSSDLTIKGS
VIFDTAVMKITKINKNAFGQCKP
TNVKLPDTIV-SIGDSLFKNCES
LISINIPLLIKTIPESCFENCYN
LRTVKLSSLTQEIQRNAFKHCYS
LNNIDLSS-ISYIGPYAFSFCSN
LTSISIMKPDSSIFERAFYNCTS
LNKVTCIDYSIFKSPEIFSGCKN
LTGFSSSINISGYIYDIYNTKYAS
LTDIPDFDQKIKYPNNTVI----
ISYLFDKLPQKGLTISQFVSYVNPE
LFRKIDSNYKIKIDKNNPKYKIKAS
IITYNDRIIKVMNST--------
FNSMKLSSSIRVICENSFSY-SN
LSFLITSKNLHRIEQYSFYR-SS
ISSIQLSEKLQIIDSYAFYQ-SN
LSSIYLPRSVLFVGHHSFAGCPN
LNSIQVNCSNSIVENSLFDSCRS
LKVVYYDCSVVPC--SCFLNCER
LSFVYLDNNVKLVERDSFRSCTS
LETVRIGNSLSEIKENAFYSCSS
LISMEIPQSIRKIGENAFYGTK-
LTLRFIVTKKYKFRYICKNFIE-
-----LVRLENATAIESFDVQESIIGFGNRSISNNFY
LTEANIPIGVKYLGDECFANCSS
LKTVLLPHTLKIIGKNCFLHCNN
LKEISIPESIYEIGEFAFRNCSL
LSRI
FYCSDFDVEISHPLVFNTSTLIHVSKNYQGTTLCGYKVIQEKNMRCKFTKAAVNVWKKRFMLLLYVIIVVSAAIA
FGAIFQNKIPNNQNTVGNTNNDTQFNDQILNLNDIKDVLD
```

>TvBspA788 --- TVAG_268070 (TpLRR positive)

```
LxxIxLxxxVxxIgxxAFxxCxx
MLSLLLSASICKADIQYDGATGTVTVTANEEWTADEWYSIFSDIKN
FTKAVLKGTYTAIPREAFSGAIN
MTEIDIQAPIVNISAYAFAQCNS
LKTFTLPDSVQIVGYAAFSSCEN
LESFYWKNTNRYFGEYCFYNCPK
LATFDHAEGDQTQP---------
-----IEGKKFVYSNFTFQSCIA
LKTCNLHPSVRFIAPHLFDGCSS
LATFTLPENVTIINDFSFSACG-
LTTFDASQ-IATINQFGFAGCLK
LHTIKLSDKLRYIGPHAFETCIE
LQTVEGITNNNDFTIDDSVFEGA
LSLVSFHVPECMTKIPDYVFHNAQK
LASVNIPIGVTYIGKYAFLNTS-
ITEIYLQGNVTTIREGAFGALRN
LKKITV-----------------
TETNFNFKSVNNLLMTKDGKIIVAYPQDLH
ETKITIPDGAEYIGQYCYSYASS
ATEVVFPKSVIDIGLHAFAGCKS
IQSITLPEHVETISDYAFQGC--
TGLIEINIQAKSICLGAFENCKS
LKRVTFPAQLELIGNEAFRNCAS
LNEVNLQNCVNLTYLGNAT----
-----------------FIFCSG
LKKVTLPDKIRVIPNQCFFYCSN
LQSFTFPPNVRRIRDHAFSYCKN
LKSITLPEGLTNLGTKAFHN-TA
LTHITVPANVSFIPNGVFSDIPT
LETAEFKGVLRSFSANVFANDAR
LTKIQFWDDVKNFDKLALEGTRI
LNSILYCGQVAVEGTFVEDHTR-
IGSRITVQVGSDYPIETFGGVKVEKTVGICIPHRTPQPTPLASATPKPDNSKNKKLYIAIGVSAGVIILAVVAAA
FIVKCCMVKRGWVKKETLTESLLTQTV
```

>TvBspA805 --- TVAG_154640 (TpLRR positive, ~ 26 x LRR)

```
LxxIxLxxxVxxIgxxAFxxCxx
    MLFCLFSSAYCYCPEGSYA
-----IPSGKPSIEDSAFYECTS
LTSITIPNSVTSIGNSAFSGCSK
LTSITIPNSVTSIGIYAFYECTS
LTSITIPNSVTSIGNGAFDSCSS
LTSITIPNSVTSIGYNAFSGCSS
LTSITIPNSAKSIESSTFYECSS
LTSITIPNSVISIEDSAFCKCTS
LTSITIGNSVTSIGIYAFSECSS
LTGITIPNSVTSIGNSAFSECSK
LTSITIPNNVTSIGIYAFDSCSS
LTSITIGNSVTSIEDSAFSGCSK
LTSITIPNSVTSIGNSAFSECSS
LTSITIPNSVTSIGNSAFSGCSK
LTSITIPNNVTSIGIYAFYECTS
LTSITIPNSVTSIGNEAFYECSS
LTSITIPNSVTSIGYTAFYECTS
LTSITIPNSVISIEDSAFCKCTS
LTSITIGNSVTSIGNTAFYECSS
LTSITIPDGVKSIGKNTFYNCSK
LTSITIPNSVISIEDSAFYNCSS
LTSITIGNSVTSIGNGAFDSCSS
LTSITIKTTEDI-----------
---------TNSVKTDAFVNCP-
ITELIYETTGFSILTFDYFKDT–
V-KLVKFNIPKSNSNS-------
--MIRLQEIKSLPTLTHFTK---
LDKVTIENINELTIPESFVKGD-
KFEIFISNNIKFIDPNAFKDCSIN
KFTYLGTDKLEGDFLKNAKSCEE
VIISVKYSNNEIGGKTINYKYNEETGEYEN
PNQPGGNPGD
PNQPGGNPGD
PSQPGGNPDD
PNQPGGNPGD
PSKSKENKANKGGKTAGIIIGSLIGIWLVAAICFGVYYYFVHFKPKNKKDDNEYNDEETIAIGTNELTNENVLAT
IDELPNNDYASAEV
```

>TvBspA923 --- TVAG_355160 (TpLRR positive, 35 x LRR)

LxxIxLxxxVxxIgxxAFxxCxx
MIFSWIFLIHTASNLQDTDTLIIPEDTKEIREYQYANLNN
FTKILFPKSLEIIHEHAFSCCKN
LESIELPPNLSIIEDNSFEYCKR
MKTVVFHSENTIISQYSFQGCYA
LEIIQLPSNLKIISSHLFTNCSN
LQTIQLPTSLQSIQNNAFYGCES
LQHIEIPSNVTYIGKGCFYHCTN
LQTIQLPASLESIQNTTFSGCTS
LQHIEIPSNVTSIGNGCFRYCSK
LQTIQLPASLKSIEGYSFYRCRS
LQHIDLPQYAM-IGQYCFCNCTN
LQSIKLSYGLE-IQAGTFKNCIN
LGKIDISD-VKSIDDYAFYNCTK
LTFDSFSSSLSFIGKFAFSYC--
LSIKNINIPTDNIREGVFSNCFG
LETIQFNFWLFTIEDKASENCIN
LGEIKFSLAIISIGKYAFYNCTK
LTFDSL--PVDNISDYAFAYCNN
IKRIVFLI----------------
-------TRILETGTDVFANCAN
LESVEFQDKLTTINNNLFRNCYS
LKSIQIPDSLKFIGNSSFQNCIS
LESFTFTENVEKIGEFAFEGCIN
IKSITFLNSSRQVNIEKYCFKDLTS
LENITFPSSMNSIPIGLFCNCTS
IVRIEIPNSITQIDPRAFSHCNK
LKELTLGTSIITLFDECFCECSS
LEIVNIKSEKLTSIGIKSFFNCY
LKSISFPVSLQKIELHAFANCAN
LTELTFSIALLELYEGCFINCTS
LKKINLPNGLKIIGKNSFSNCYE
LHEIIIESNCS-IKGDVFENCSK
LENIIINSQNYDVFQIESFHNTV
-KSITFNFSVIQYPSVSSFHH--
LENIKIFSYENDSFINEYFITSS
NVSISIIGNIKQI-SDKSFSNSY
---INTFLYCGDRSVEGKFLSKD
RVKIVNISEYYPHISIGGVHAHKTSECPNFPQKKYFKLTTLHIVLITVSIVILSSIGITIIIKFQRYLKSQKKIE
NKIMLERLVDKDFG

>TvBspA927 --- TVAG_383590 (TpLRR positive)

LxxIxLxxxVxxIgxxAFxxCxx
MIHSLFIFRRFLRDDVPTVNFDAETSTMTISGAGTLTQELILSKDPNN
ATKTLTIEGGLTISSNCFSSHPN
LQKLTINSNNMNIQSLAFEGCPQ
LSTLELSGIFGDLGDHIFAHCQS
LTQITLPETLTVIPDSMFSNCHN
ITKATISSQTTTISHGAFADNEK
LAEINFPDTLTVIGEEAFKDTG-
LTSVTLNK-VN-LSARSFAECDA
LTTVTIAE-IAEIPSNCFFKCIS
LTTVTITSGLTKVNSQSFLDCKN
LTTFTYPDTLATLGDASFQGCQS
-LNIEIPKILTVIPDSCFETVPF
VN-LTIPDTVQTISGLAFSHNDQ
LRVVILPKSLKSIDSSAFSYCNN
VDSYTIAENANFFVTDGILFQKSEFGNTLVLYPPTNEQ
DVLMLDPYNVANIADCAFLGAK-
VKSLVATSLDSIGKFSFFSSQ--
IKTFNVREGLKEYPEYAFAQTP-
IESFSVPSGVTKIGRYCFNDCRN
LSDITFPDTLSEIGDYSFQGTN-
VQAI-VAPMVKVIGNYAFANSY-
ILGIDLTENLTYIGNNAFEGAFR
LTHITLSSSIKEILPCTFRSCRS
LKEV-VCGDIISIGDQAFEYCHA
LVNVTFKDSLQRIDFNAFHECYK
LQISEFPAAMNHIGFFGFSTCYS
LTNLTFYGDV-YINHDAFYNCIN
LQTVTFAVKARLLGSMCFSSCIK
LTTLNFLSSIIEFQKTPFYNTT-
VAEVVYCVDRP-VEGPMFPNTPT
IKVSNYYPKDKFADITPQVSE
LPQCPPLQTPLPTPESSESSSEIYTPTPIPSYTPEPPTSSSSESSSTETETPK
PTHIPTSK
PTETPSPD
PTETPSPD
PTETPSPD
PTETPSPD
PTQTPTPE
P-DTPSPS
RS
PTKEPNTPKPTQSSSSTAPGPSSSPTPDPAAQKRKRTIIYATTGSILAIIIIVVVVILVVTALSRRKRAQSEILT
DSILTQIL

Note: This sequence has two low complexity stretches (blue highlight) in addition to the shown LRR and P/TRR (purple) 4x PTETPSPD – with capital letters indicating fully conserved residues in the alignment.

```
>TvBspA950 --- TVAG_139560 (TpLRR positive)

LxxIxLxxxVxxIgxxAFxxCxx
MLFFFHFFIAQTLSANEISFALDKDTLTITGNGDLTQELLNEKVKSKFNKLIINANSIKLSDSLFEGNTD
IESVTISSGISEISNSVFSGCSN
LKTIDISCSINTIGELSFADCTS
LSEIKLPSTVSSIGKEAFKKCTS
IKTHTLPLHVLKIEEGVFSYNYA
LLSIKLHDSLTYIGRRAFYWCKN
LKSIEIPESCTVVSDYAFEFCVD
MTNLTLPNSLQLLGNSSFMQCHS
LKTITISQNL-------------
------PSTDCKFGVSAFEGCES
LEKLTFPESISSLGMRCFLDCSS
LESITIPETITQISFKCFEKCKS
LKYVTLHSKIKYFANNVFFDCHA
LISIDIPQQVTRISDLCFTYCRN
LKTVNLSPNLKTIGTSSFSGCDN
LETITFPATLEVVGKNCFQNCKL
LNNIELPSNLSDIGDSCFYGCES
LSKISLPNSIKFVKSATFEDSTS
LSEIVLSQYIEEIRDSAFCNCKS
LKEINLPSTLKSIGNYVFFGCKS
LEKVK------------------
CDPRNRYFISENNILFSYSKAE-
LILVP---ACYNSDKFSFPST--
VSTLHHSSFAYNKY---------
ISEIIIPDHVETIGFASFYECTN
LKSIKLSNKLKEIDGKLFYGCSS
LSSIEIPNSVTCIRLGTFTSCSS
LSSVKLSENLETIEGFVFQNCIK
LESIKIPNGIQEIRQYCFGN-SG
LKSIELPENVSMLERYCFSNCRS
LKTIKLSDKIHTFGDSCFENCSS
LETLVFPENLGVLGQKSLSMCTS
LVNLTLSDKLRKISRDTFYGCSK
LENIKVSN-VSEIESSAFRDCTS
LKEMRLPPSIVDIPFNLFWNCVN
LRKVIIASELNYIDNNAFCYCNN
LKVIIFLQNIFGISPYAFAYCEK
LSDIYFCGNQDVLGEISFSNTTI
HVSNSSVTNFATNNVTYDGQKVCADADIDIDVKKSKNNVLLIVMIVVTSLLLVSFIILIAFYIRSRKMVKSDFTE
SLLSNTLV
```

```
>TvBspA1047 --- TVAG_158740 (TpLRR positive)

LxxIxLxxxVxxIgxxAFxxCxx
MLNSLTLTRLLTLNVYLDTAALAGPTVEISDSSDFTNDET
LSLIIKNG---------------
AVNLRLTGSLTKMV--RFQGFEL
VKNVDIL----------------
------AAGITEIPEKCFYQNNY
IETVTLHGGVTKIAQNAFTY-SS
LRSINLEN-VETIGQYSFQKCYN
LQSVTLKS-IKSLGAAPFYSSGI
ETASITLSDELTIPASCFENCLS
LTKVSFVSA--------------
-----QGSQNLILGDYVFNNCPQ
LQTVTSTNVNIGVGDYAFANTA
LTSFPFE-LATSIGSYSFMNTK-
MATIKPASG--------------
-------TISFELSQLTFQFNSE
LQTVDLSGITGTF----------
-----------TNSYGLFYGCSS
LTTVTLPASLTKLPDAMFKDCTA
LTSVTASGLTE-ISTDGFMSCSA
LTNLQIGSQLTTVGYRGLYDCQK
L-NVQIQNCILKERALYN--CQS
ITTISVKG-LGSWCCMNM---SN
LNTVTVSYSPTSIPTGCFMYCTK
LQSFTIPASCSSIGDLAFAYTY-
LP-IQIDLQNVEVLGNYSFMRSN
VSGVYMRKQIQEEGKPAFFECPN
LKNIRIGQRILRFWMVPFTGCNH
EFEFQCDDQFNYWVPSQKIFYS
VYSVAYVAPDAVIPYNLWAIMIL
NGAFMWYKKPFDIATNVKQYYFLDHST
VTTLFFSDDA-------------
---IGSQYYGDKYSIGDFSYCTS
LTKVHLAPTIKYISTGCFYMCTN
LTQINLENVIA-ISHSVFAGCVS
LESINI-SKATEVLSEAFINCTG
LKNVVFPSTFDLGSLVGIGHFGNTGFEE
LYFNAN-IQNLTLYKGMFVNCKK
LTKVVFGPNVVSLPDSMFENSS-
LSVVEYSDSLTSIGNMTFARTNI
 QTFTIKGSLTDIYPIAFYGAYS
INIVVSPEN
TKYEIIDGNHLIEKSSKSLVCLF
GKIPNTFKLSEEIRVLKDASLASAPEIDSNTGKVIDWGITTLIIPGNIRVEDKMEPVRYTPYLHNLCYGG
IDQPPLLNSMAHRYFVTSNYSKDFWVLSEEGDMDNENYIGVIRAPCSNFTPYDKFNQREFYNLYDC
PTDEIIEEEPTETPTPELQKDLPTNKKISTLLLILIIVAAVETVFLISSESSSLQRRRVMIPMMKILITLI
```

Note: This sequence has one low complexity stretches (light blue highlight) in addition to the shown LRR.

>TvBspA1102 --- TVAG_468690 (TpLRR positive)

LxxIxLxxxVxxIgxxAFxxCxx
MH
ELETVTIVDYDIIPEFAFYNCFK
LKSITFTQPITVIGNNAFENSA-
IKTFDFSNII-AIESYAFRNSK-
LETAILPNLK--LYPEAFAECKY
LTSVTISDKVTFV----------
------SMIGIYSPIEHFRDCYA
LTTVNY-GNTGTVSKGMFKFCYS
LKTFSSSNNSLEIQDEAFYSCTS
LTDIS-ATQVGRIGSKAFILCNS
LTSFT-PQFST-LGDKAFYGCEK
LGTID--AHDCGKYSFAF--CPS
---IKSCILRSTLHTGVMMNCTS
LES-AELVNVRAIPDKCFFGCTK
LKTITFPTTVTVESIGNYAFQDTILE
LETLDLSG-CNQIGNYSFRDVK-
IKNLKLGNNYQTFTRPQYEIGQGPFADVKS
IEHITIAANSEAFKAVDYKDSPDVTFTFETPS
IYTEDDNLITKDNEIYYFKPSSS
LDHFEFTSTHANIHYYAFSNAKN
LNKVTARKSLSSS-PFAFARCKSLKTAYISMEGIES
QRILKDNDEQPIVPTGLFANCTN
LESVTIGHGYTVISESVFEGCSK
LMSVIIIDEGVSLGAFCFANCTS
LTDIKFNS-INRVDAYCIAGCTN
LTTLNFTN-IESAAEAAFAF-SS
LENIVVPDHF--LVNFSFSYCRN
LKKVQIVKGG-------------
-------HYDYASINRGCFNCSS
LEEINLSDNIQSIGEYAFGFTK-
LKYLYLPNNVSEINSTAFYGSDD
I-QLDLDECSHLYFTLGNYELIE
RATYKLVLTFGKLPSTYIVPQQIKVIGSNSILSRPIFNEQTSKVVDFGLTTLVIRGNVEIESNPVFQDLYLHNLC
YGGNMKPFDFDVPNVKRIFVTERYIKRKWGFDQSVIFDDCDTSVPFENYANNINKYPENPTYIPYPTPRPKKKYV
CSILGEIETIIEPEIIQPEQEPSSNITE
--PSNE
QTPSSE
QTPSSE
QTPSSE
QTPSSE
QTPSSE
QTPSSE
QTPSSK
QTPSSE
QTPSSE
QTPSSE
QTSSSE
QTPSSE
QTPSSE
QTPSSE
QTPSSE
QTPSSE
QIPFNE
QTPINE
QTPINE
QTPSNE
QATSSE
FISTVVDPKKGSGFSNLIIGLIIAVIVELLLVLVMILFIIIKSRDDDSDSSFIEMDEEAVINARLDSTAITHENV
LFTMSTMVDDDPFAQDFEDAPPNESFYTGLDDENA

Note: This sequence has an additional segment made of tandem repeats particularly rich is serine, purple highlight, 14x QTPSSE

>TvBspA1660 --- TVAG_474570 (TpLRR positive, ~ 69 x LRR)

```
LxxIxLxxxVxxIgxxAFxxCxx
MIFFLFFSPFVNSESNVKVTFNDVVFLCSKREKN
LSIENNNVDLPAIDT-NFNGSIHLDGF
KSINDEVYTVTNIGQGAFAKTN-
VVSVTLPDTIIEIGAGAFAYMNRC
MFVDLENTKITKLSDKIFYNSS-
ITEVKLPNTLISIGEKSFEKSS-
IINISFLPSFLSIGRAAFRNCRN
LQNIDLIST--------------
------GITTIPAECFAFSS---
ITNLILPENLISISDHAFFNSS-
LVTISFPDKLSEIQEFSFANCEN
LTTIDISETK-------------
---------VNVISKSTYENCTK
LQRIRTSLLLTSICERSFYNSG-
LLSFTAMQSVNKIKTSAFEHCTEM
IKIDLTRCNLSVLEDYVFSSCYK
LSSIKLSPNLYSIGVGAFQNTQ-
ISSFEAKFNLYDIQSSAFENCTS
ITKVSLEES--------------
--------SLSSINERTFFNCSA
LKIIILSKTIKTICNEAFRSSG-
LESFASDTVEKLGIGVFTDCDC-
LQLVSLTSVHEIPNYMFQWCPA-
LTKLEVSGEIRKIGNLAFSFCFD
LKEIDLSKT--------------
--------RIETIGDSAFAFTSS
LEKILFPDSLISLGTCCFFESS-
IKNIK-TPNLLYIGLACFQDSE-
ISEIFLQK-VTKITKNCFKHSK-
LRKITINSAKT-IEDGAFQDTL-
IEEIELPDEIEEIGNFSFANSS-
LKNINLEKTL-------------
---------LRSLRKSLFVNASE
LTKVTLPDSISVIEDNCFENCLK
LEEINLQKT--------------
--------NLKFLKNDVFVNCQN
LKSIELPKTVLEIGERCFSRTA-
IEYFSFDKYLHRIGDFCFANNEF
LTKIDMYNT--------------
--------NLTFLGKELFVYDWN
LKEIYLPKRISKLEFGCFSFSG-
LNQIILPESLKIVSEKLF-FSSLL
TYADLSRTNLDSLNYQMFYNCTL
LKSVKLPQTVLEIGFECFKNCSS
LSKINMNSKIYKISQGSFSFCQS
LTKFDISQTNVKELSKEVFLNTP
LNKIELP-IHLTTINDRCFSGTN
LTEFIASENISFIGEKSFSNCQN
LKIIDLSKC--------------
--------DISIVPSGFVFNCSS
LSEFKLSNNTNCLGDFAFSNCVF
-KKHKFGEKIFEIKNYCFSSSPS
VEEIDLSQT--------------
--------KITKIEEGTFYNCIS
LSRILLPNTVFSFGNFAFTQTA-
LTEIKFVKTVFSVGISLFENCSL
LETVDLSET--------------
--------KLTFLSNSMFYNCVK
LTKIKLSPFCRHFSRQCLSNTF
ISEIKIPEN-CVLEDNVFYGCKR
LEAVIISN-LNQSKIPNFLFHGT
LIKTIKFPSNIISIGCQSLSETK
ITEFIASSSLTEIGEFCFEDCSC
LKTVDLQST--------------
```

```
--------KIEKLPQGLFYNCTK
IEKIKLPENLRSIGISCFAFSG-
IKYFYGTKFLDVIQTAAFKNSD-
ISEVNLNE---------------
-------CPITTISSECFMNTFN
LKSFS-YQYIEIFEEKCFMNSK-
IEKFTFQKGLVTLNPFCFSCCIN
LVDIDLSL---------------
-------TSLTTISTSLFENSTG
LFSVILPEKLQKIDQKSFYNTS-
LKKVTIPSSVTFLGSYSFSNNDK
IEEFDCS----------------
------LCTDVTINDGCFSNCNL
LKQINIPS-IKFIGNFVFEGTS-
VSKVSLPSSLTNIGKGIYKNCKS
LEEVDIYLL--------------
--------KLTELTSELFSGCTN
LKHVSLPFNVRKIAKDCFFDCRN
IKKIRYCGGSAVI-GDFILPEGT
VVFVSESYPSDLF-GGFPVIKSS
KCNDWNAFANKQQKPNMFLVIFLSVSSIFCGIFVVLLVAKIAGLKISLIVEARNHDTDRLIDRNGSSMSYPEEEE
FL
```

Figure S4. Alignment of TpLRR and/or other repeats for selected TvBspA-like proteins with transmembrane domains.

The segments corresponding to the TpLRR pattern (LxxIxLxxxVxxIgxxAFxxCxx) was aligned manually to the shown sequences when possible and matched to the 23 positions and where necessary gaps were introduced – several entries have numerous indels in their TpLRR. The TpLRR consensus sequence is shown above each TpLRR alignment. The TrichDB locus tags are indicated. Residues L, I, and V are highlighted in blue whereas C and F are highlighted in red to ease TpLRR comparisons. In several cases the sequences were not positive for either the TpLRR profile or pattern (see text). In some cases, e.g. TvBspA625 and TvBspA805, which are closely related to each other (see Figure 2), the TpLRR are well conserved within each respective protein. These two proteins also possess a P/NRR, which are related but distinct between the two proteins. Residues part of the TMD domain (TMHMM2.0 inference) are highlighted in red.
