# Supplementary material for: Trichomonas vaginalis vast BspA-like gene family: evidence for functional diversity from structural organisation and transcriptomics
Source: BMC Genomics. 2010 Feb 8;11:99. doi: 10.1186/1471-2164-11-99 (PMC2843621; doi:10.1186/1471-2164-11-99)
Supplement: Additional file 12 — Supplemental Figure S5. Alignment of the GRD from TvBspA-GRD and proteins with related GRD. Clustal alignments of the 12 TvBspA-GRD with nine proteins from RefSeq with related GRD - screen shots from SEAVIEW. [file 1471-2164-11-99-S12.PDF]

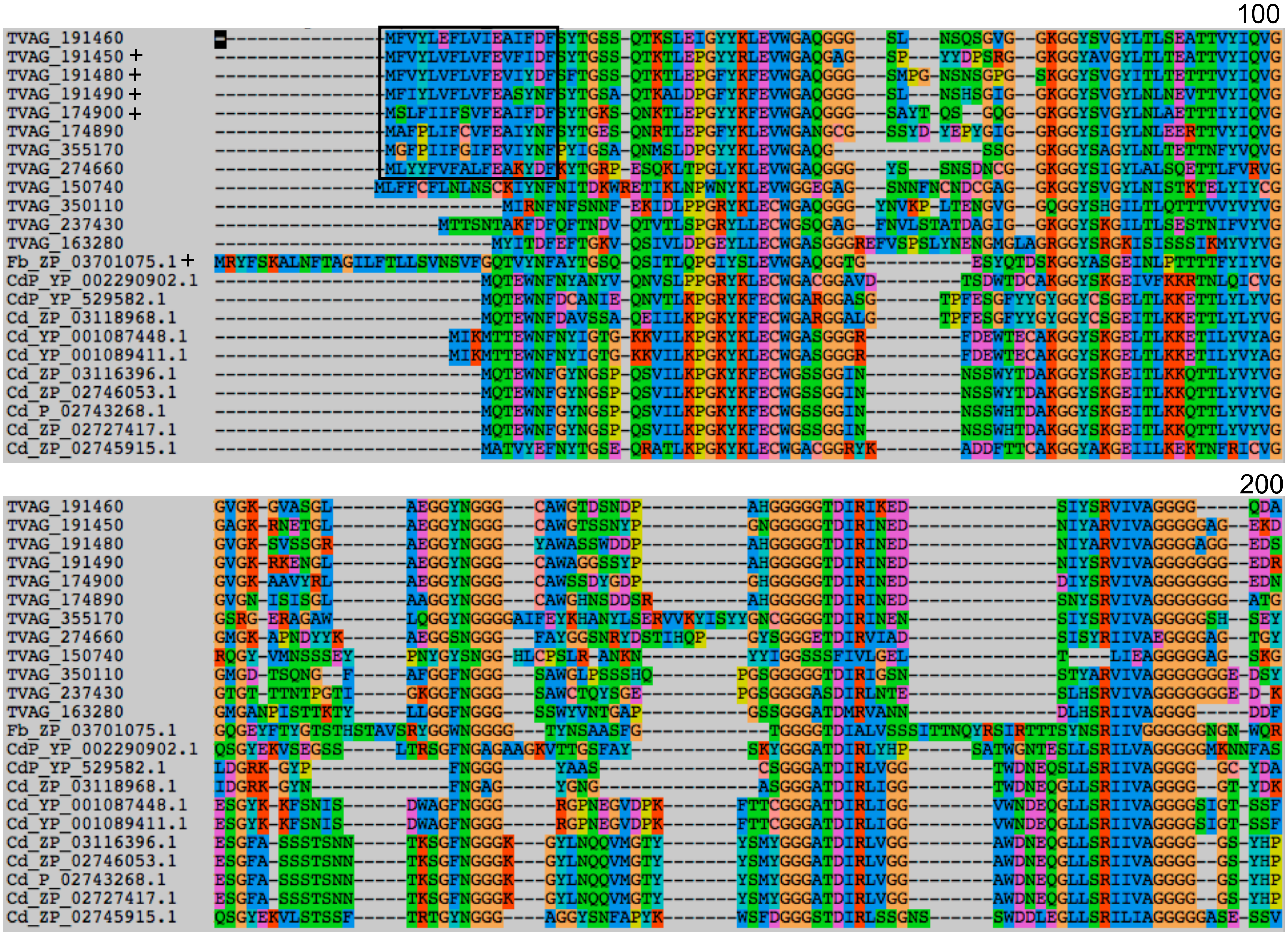
100
200
TVAG_191460
TVAG_191450 +
TVAG_191480 +
TVAG_191490 +
TVAG_174900 +
TVAG_174890
TVAG_355170
TVAG_274660
TVAG_150740
TVAG_350110
TVAG_237430
TVAG_163280
Fb_ZP_03701075.1 +
CdP_YP_002290902.1
CdP_YP_529582.1
Cd_ZP_03118968.1
Cd_YP_001087448.1
Cd_YP_001089411.1
Cd_ZP_03116396.1
Cd_ZP_02746053.1
Cd_P_02743268.1
Cd_ZP_02727417.1
Cd_ZP_02745915.1

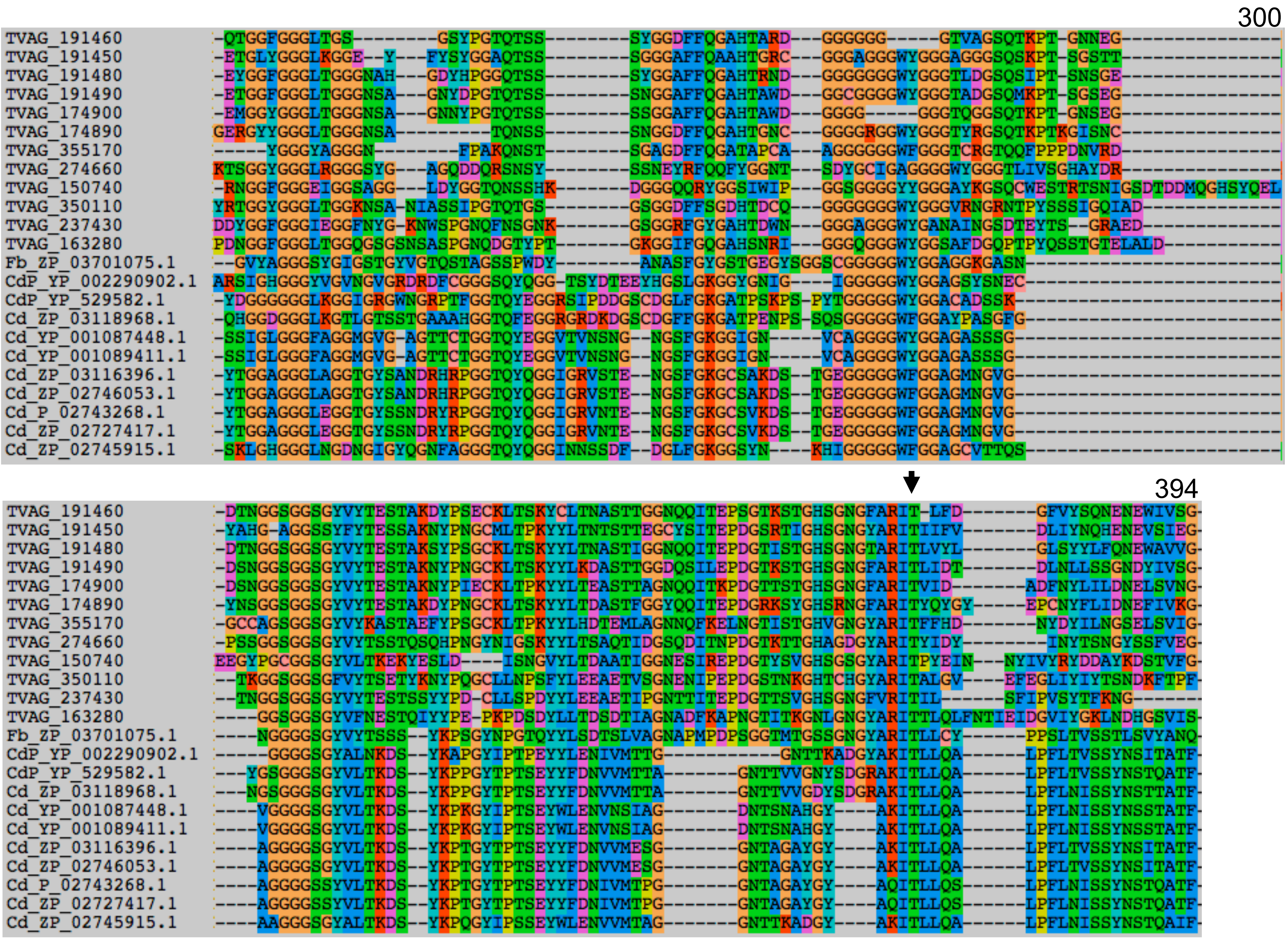
300
394
TVAG_191460
TVAG_191450
TVAG_191480
TVAG_191490
TVAG_174900
TVAG_174890
TVAG_355170
TVAG_274660
TVAG_150740
TVAG_350110
TVAG_237430
TVAG_163280
Fb_ZP_03701075.1
CdP_YP_002290902.1
CdP_YP_529582.1
Cd_ZP_03118968.1
Cd_YP_001087448.1
Cd_YP_001089411.1
Cd_ZP_03116396.1
Cd_ZP_02746053.1
Cd_P_02743268.1
Cd_ZP_02727417.1
Cd_ZP_02745915.1

Figure. S5. Comparison of the glycine-rich domain for the 12 TvBspA-GDR and nine proteins with related GRD proteins.

Protein alignment comparing the glycine-rich domain (GRD) of the 12 TvBspA-GRD and 11 related sequences from *Flavobacteria bacterium* (Fb_RefSeq accession), *Clostridium difficile* (Cd_RefSeq accession) and the *C. difficile* phages (CdP_RefSeq accession). Only the alignment covering the N-terminal GRD is shown. The more divergent, but related, GRD containing sequence from *Bacillus cereus* is not shown for simplicity (see Table 3 and additional file 13, Table S8). The number of the last aligned position is indicated on top of each alignment block. The vertical arrowhead above the alignment indicates what was considered by visual inspection of the alignment as the end of the conserved GRD and the beginning of the unrelated C-terminal domain of the TvBspA proteins and related sequences. Four *T. vaginalis* TvBspA-GRD proteins and the *F. bacterium* sequence have an inferred signal peptide (+) and five additional TvBspA-GRD sequences have similar hydrophobic N-termini to the four TvBspA-GRD entries with an inferred SP, these could also correspond to functional SP not recognized by SignalP3.0 or PHOBIUS - the eight most similar TvBspA-GRD N-termini extensions are boxed. The Blast search that identified these sequences used as query the TVAG_174900 GRD sequence, residues 1-267 (ending on the universally conserved threonine - arrowhead - see Table 3 and additional file 13, Table S8).
