# Supplementary material for: Trichomonas vaginalis vast BspA-like gene family: evidence for functional diversity from structural organisation and transcriptomics
Source: BMC Genomics. 2010 Feb 8;11:99. doi: 10.1186/1471-2164-11-99 (PMC2843621; doi:10.1186/1471-2164-11-99)
Supplement: Additional file 13 — Supplemental Table S8. BlastP taxonomic report for proteins with GRD. Full taxonomic report of BlastP search on NCBI RefSeq protein database. In html format to be open in a web browser. [file 1471-2164-11-99-S13.HTML]

### Table S8. BLASTP taxomic report for proteins with GRD

---


### Query: residues 1-267 from TVAG\_174900 (TvBspA-GRD)

### e-value <= 0.001

---

- Lineage Report
- Organism Report
- Taxonomy Report
- Help

**Lineage Report**  

```
root
. cellular organisms
. . Trichomonas vaginalis G3 ---------  529 63 hits [trichomonads]        surface antigen BspA-like [Trichomonas vaginalis G3]
. . Flavobacteria bacterium MS024-3C .   84  1 hit  [CFB group bacteria]  hypothetical protein Flav3CDRAFT_0584 [Flavobacteria bacter
. . Clostridium difficile ATCC 43255 .   70  2 hits [firmicutes]          hypothetical protein CdifA_19608 [Clostridium difficile ATC
. . Clostridium difficile QCD-63q42 ..   70  3 hits [firmicutes]          hypothetical protein CdifQCD-6_16556 [Clostridium difficile
. . Clostridium difficile QCD-37x79 ..   68  1 hit  [firmicutes]          hypothetical protein CdifQCD-6_20698 [Clostridium difficile
. . Clostridium difficile 630 ........   65  2 hits [firmicutes]          hypothetical protein CD0967 [Clostridium difficile 630] >gi
. . Bacillus cereus Rock4-18 .........   58  1 hit  [firmicutes]          FG-GAP repeat protein [Bacillus cereus Rock4-18]
. Clostridium phage phiCD27 ----------   71  1 hit  [viruses]             hypothetical protein phiCD27_gp26 [Clostridium phage phiCD2
. Clostridium phage phi CD119 ........   71  1 hit  [viruses]             hypothetical protein CDBPCV119_gp31 [Clostridium phage phi
```

---

**Organism Report**

```
  Trichomonas vaginalis G3 [trichomonads] taxid 412133
 ref|XP_001319243.1| surface antigen BspA-like [Trichomonas...     529  9e-149
 ref|XP_001317272.1| surface antigen BspA-like [Trichomonas...     335  2e-90
 ref|XP_001317275.1| surface antigen BspA-like [Trichomonas...     322  2e-86
 ref|XP_001317274.1| surface antigen BspA-like [Trichomonas...     311  5e-83
 ref|XP_001317271.1| surface antigen BspA-like [Trichomonas...     261  4e-68
 ref|XP_001319242.1| surface antigen BspA-like [Trichomonas...     256  2e-66
 ref|XP_001327646.1| loricrin [Trichomonas vaginalis G3]           219  2e-55
 ref|XP_001311122.1| PE-PGRS protein [Trichomonas vaginalis...     175  4e-42
 ref|XP_001320681.1| surface antigen BspA-like [Trichomonas...     164  5e-39
 ref|XP_001322326.1| surface antigen BspA-like [Trichomonas...     155  3e-36
 ref|XP_001582703.1| hypothetical protein [Trichomonas vagi...     145  3e-33
 ref|XP_001313444.1| hypothetical protein [Trichomonas vagi...     143  2e-32
 ref|XP_001581618.1| hypothetical protein [Trichomonas vagi...     141  7e-32
 ref|XP_001315806.1| loricrin [Trichomonas vaginalis G3]           129  3e-28
 ref|XP_001323933.1| PE-PGRS protein [Trichomonas vaginalis...     117  7e-25
 ref|XP_001319025.1| PE-PGRS protein [Trichomonas vaginalis...     108  5e-22
 ref|XP_001311421.1| hypothetical protein [Trichomonas vagi...     102  4e-20
 ref|XP_001315979.1| PE-PGRS protein [Trichomonas vaginalis...     101  6e-20
 ref|XP_001322681.1| hypothetical protein [Trichomonas vagi...      94  8e-18
 ref|XP_001314781.1| hypothetical protein [Trichomonas vagi...      93  2e-17
 ref|XP_001582840.1| PE-PGRS protein [Trichomonas vaginalis...      87  1e-15
 ref|XP_001579937.1| loricrin [Trichomonas vaginalis G3]            83  1e-14
 ref|XP_001320839.1| PE-PGRS protein [Trichomonas vaginalis...      80  1e-13
 ref|XP_001329864.1| hypothetical protein [Trichomonas vagi...      79  3e-13
 ref|XP_001317923.1| loricrin [Trichomonas vaginalis G3]            78  6e-13
 ref|XP_001329224.1| PE-PGRS protein [Trichomonas vaginalis...      77  7e-13
 ref|XP_001313749.1| loricrin [Trichomonas vaginalis G3]            77  8e-13
 ref|XP_001321767.1| loricrin [Trichomonas vaginalis G3]            77  1e-12
 ref|XP_001324239.1| glycine-rich cell wall structural prot...      77  1e-12
 ref|XP_001580689.1| PE-PGRS protein [Trichomonas vaginalis...      77  1e-12
 ref|XP_001311437.1| hypothetical protein [Trichomonas vagi...      75  3e-12
 ref|XP_001321263.1| PE-PGRS protein [Trichomonas vaginalis...      75  4e-12
 ref|XP_001313737.1| loricrin [Trichomonas vaginalis G3]            72  3e-11
 ref|XP_001305999.1| PE-PGRS protein [Trichomonas vaginalis...      69  2e-10
 ref|XP_001580859.1| PE-PGRS protein [Trichomonas vaginalis...      65  3e-09
 ref|XP_001322186.1| loricrin [Trichomonas vaginalis G3]            63  2e-08
 ref|XP_001311443.1| eggshell protein 1 precursor [Trichomo...      62  3e-08
 ref|XP_001318485.1| hypothetical protein [Trichomonas vagi...      62  4e-08
 ref|XP_001318486.1| hypothetical protein [Trichomonas vagi...      60  2e-07
 ref|XP_001311505.1| hypothetical protein [Trichomonas vagi...      58  4e-07
 ref|XP_001579041.1| PE-PGRS protein [Trichomonas vaginalis...      57  9e-07
 ref|XP_001320838.1| hypothetical protein [Trichomonas vagi...      56  2e-06
 ref|XP_001308516.1| hypothetical protein [Trichomonas vagi...      56  2e-06
 ref|XP_001301918.1| hypothetical protein [Trichomonas vagi...      56  3e-06
 ref|XP_001317566.1| hypothetical protein [Trichomonas vagi...      56  3e-06
 ref|XP_001316417.1| WAG22 antigen precursor [Trichomonas v...      56  3e-06
 ref|XP_001329945.1| hypothetical protein [Trichomonas vagi...      56  3e-06
 ref|XP_001313167.1| hypothetical protein [Trichomonas vagi...      55  7e-06
 ref|XP_001297098.1| hypothetical protein [Trichomonas vagi...      54  1e-05
 ref|XP_001584391.1| loricrin [Trichomonas vaginalis G3]            54  1e-05
 ref|XP_001329145.1| hypothetical protein [Trichomonas vagi...      53  1e-05
 ref|XP_001326800.1| hypothetical protein [Trichomonas vagi...      53  1e-05
 ref|XP_001579088.1| hypothetical protein [Trichomonas vagi...      53  2e-05
 ref|XP_001305374.1| hypothetical protein [Trichomonas vagi...      52  3e-05
 ref|XP_001320321.1| hypothetical protein [Trichomonas vagi...      51  5e-05
 ref|XP_001305295.1| loricrin [Trichomonas vaginalis G3]            51  6e-05
 ref|XP_001301662.1| loricrin [Trichomonas vaginalis G3]            51  6e-05
 ref|XP_001308095.1| loricrin [Trichomonas vaginalis G3]            51  6e-05
 ref|XP_001318466.1| hypothetical protein [Trichomonas vagi...      51  7e-05
 ref|XP_001313098.1| loricrin [Trichomonas vaginalis G3]            51  7e-05
 ref|XP_001330682.1| hypothetical protein [Trichomonas vagi...      51  8e-05
 ref|XP_001301664.1| PE-PGRS protein [Trichomonas vaginalis...      51  9e-05
 ref|XP_001293577.1| PE-PGRS protein [Trichomonas vaginalis...      51  9e-05

  Flavobacteria bacterium MS024-3C [CFB group bacteria] taxid 487797
 ref|ZP_03701075.1| hypothetical protein Flav3CDRAFT_0584 [...      84  8e-15

  Clostridium phage phiCD27 [viruses] taxid 559189
 ref|YP_002290902.1| hypothetical protein phiCD27_gp26 [Clo...      71  6e-11

  Clostridium phage phi CD119 [viruses] taxid 320122
 ref|YP_529582.1| hypothetical protein CDBPCV119_gp31 [Clos...      71  7e-11

  Clostridium difficile ATCC 43255 [firmicutes] taxid 499175
 ref|ZP_05352971.1| hypothetical protein CdifA_19608 [Clost...      70  2e-10
 ref|ZP_05352943.1| hypothetical protein CdifA_19468 [Clost...      54  7e-06

  Clostridium difficile QCD-63q42 [firmicutes] taxid 479831
 ref|ZP_05331407.1| hypothetical protein CdifQCD-6_16556 [C...      70  2e-10
 ref|ZP_05332221.1| hypothetical protein CdifQCD-6_20698 [C...      68  6e-10
 ref|ZP_05331965.1| hypothetical protein CdifQCD-6_19418 [C...      62  4e-08

  Clostridium difficile QCD-37x79 [firmicutes] taxid 479834
 ref|ZP_05399502.1| hypothetical protein CdifQCD_20626 [Clo...      68  6e-10

  Clostridium difficile 630 [firmicutes] taxid 272563
 ref|YP_001087448.1| hypothetical protein CD0967 [Clostridi...      65  6e-09
 ref|YP_001089411.1| hypothetical protein CD2897 [Clostridi...      65  6e-09

  Bacillus cereus Rock4-18 [firmicutes] taxid 526988
 ref|ZP_04209673.1| FG-GAP repeat protein [Bacillus cereus ...      58  4e-07
```

---

**Taxonomy Report**

```
root .......................................    75 hits    9 orgs 
. cellular organisms .......................    73 hits    7 orgs 
. . Trichomonas vaginalis G3 ...............    63 hits    1 orgs [Eukaryota; Parabasalidea; Trichomonada; Trichomonadida; Trichomonadidae; Trichomonadinae; Trichomonas; Trichomonas vaginalis]
. . Bacteria ...............................    10 hits    6 orgs 
. . . Flavobacteria bacterium MS024-3C .....     1 hits    1 orgs [Bacteroidetes/Chlorobi group; Bacteroidetes; Flavobacteria; unclassified Flavobacteria]
. . . Firmicutes ...........................     9 hits    5 orgs 
. . . . Clostridium difficile ..............     8 hits    4 orgs [Clostridia; Clostridiales; Clostridiaceae; Clostridium]
. . . . . Clostridium difficile ATCC 43255 .     2 hits    1 orgs 
. . . . . Clostridium difficile QCD-63q42 ..     3 hits    1 orgs 
. . . . . Clostridium difficile QCD-37x79 ..     1 hits    1 orgs 
. . . . . Clostridium difficile 630 ........     2 hits    1 orgs 
. . . . Bacillus cereus Rock4-18 ...........     1 hits    1 orgs [Bacilli; Bacillales; Bacillaceae; Bacillus; Bacillus cereus group; Bacillus cereus]
. unclassified Myoviridae ..................     2 hits    2 orgs [Viruses; dsDNA viruses, no RNA stage; Caudovirales; Myoviridae]
. . Clostridium phage phiCD27 ..............     1 hits    1 orgs 
. . Clostridium phage phi CD119 ............     1 hits    1 orgs
```
