# Supplementary material for: Trichomonas vaginalis vast BspA-like gene family: evidence for functional diversity from structural organisation and transcriptomics
Source: BMC Genomics. 2010 Feb 8;11:99. doi: 10.1186/1471-2164-11-99 (PMC2843621; doi:10.1186/1471-2164-11-99)
Supplement: Additional file 14 — Supplemental Table S9. Summary of all data on transcribed T. vaginalis genes. Table listing EST, microarray and semi-quantitative RT-PCR data obtained for various T. vaginalis isolates. [file 1471-2164-11-99-S14.PDF]

Table S9. Summary of evidences for transcribed TvBspA genes

Method	*T. vaginalis* isolates	Total entries	TvBspA genes	Growth condition	Tables or Figures
EST	T1, C1:NIH, TO16 JH314#4, G3	102,411[a]	270	Various conditions[b]	Figure 4
Microarray	T1, C1:NIH, TO16 JH314#4, G3	7,680[c]	73	High/low iron[d]	Table 4
RT-PCR	G3	9	9	ECM binding[d]	Figure 5

[a]71,428 from Chang Gung University and 26,550 from GeneBank dbEST.
[b]Since the cDNA libraries were derived from a mix of strains and normalized and non-normalized libraries, variations in EST frequencies for the different *T. vaginalis* culture conditions where only contrasted from from selected non-normalized cDNA libraries.
[c]From Chang Gung University. The total number of spotted entries is indicated. These correspond to 4,938 distinct annotated entries at TrichDB of which 73 correspond to distinct TvBspA genes.
[d]ECM (Extracellular matrix proteins) bound cells were contrasted to non-bound cells.
[e]High and low iron concentration were contrasted and qRT-PCR was used to confirm the microarray data.
