# Supplementary material for: Trichomonas vaginalis vast BspA-like gene family: evidence for functional diversity from structural organisation and transcriptomics
Source: BMC Genomics. 2010 Feb 8;11:99. doi: 10.1186/1471-2164-11-99 (PMC2843621; doi:10.1186/1471-2164-11-99)
Supplement: Additional file 15 — Supplemental Table S10. TvBspA ESTs frequency table. Frequency table for TvBspA ESTs, including in relation to TvBspA structural organisation. [file 1471-2164-11-99-S15.PDF]

Table S10. EST frequency table

Number of EST/ TvBspA gene[a]	Total counts	TvBspA no TMD[b]	TvBspA with TMD[b]
1	156	125 (10)	31 (3)
2	54	45 (5)	9 (2)
3	26	18	8 (3)
4	6	6	0
5	11	8 (1)	3
6	4	3	1
7	7	7 (1)	0
8	1	0	1 (1)
10	1	1	0
14	1	0	1 (1)
16	1	1	0
17	1	1	0
25	1	1	0
Total	270	216(17)	54(10)

[a]See experimental procedures for details for EST identification and additional file 1, Table S1 for all the details for each TvBspA entry.
[b]TMD: transmembrane domain (one or more) – see Figure 1 and see text. Values in brackets correspond to entries with a signal peptide among the indicated numbers.
