# Supplementary material for: Trichomonas vaginalis vast BspA-like gene family: evidence for functional diversity from structural organisation and transcriptomics
Source: BMC Genomics. 2010 Feb 8;11:99. doi: 10.1186/1471-2164-11-99 (PMC2843621; doi:10.1186/1471-2164-11-99)
Supplement: Additional file 16 — Supplemental Table S11. Summary annotation for the TvBspA analysed by semi-quantitative RT-PCR. Table listing locus tag, and selected protein features and annotations and RT-PCR results of the nine TvBspA analyses upon binding of the parasites to ECM proteins in vitro. [file 1471-2164-11-99-S16.PDF]

Table S11. Annotation summary of TvBspA genes analysed by RT-PCR upon *T. vaginalis* binding to ECM proteins.

Locus tag	Length (aa)	Annotation[a]	Additional notable features[b]	EST[a]	Modulation upon ECM binding[c]
TVAG_244930	605	BspA-like, TMD	SF #138 2 members	1	No signal (-/-)
TVAG_073760	625	BspA-like, TMD	SF #13, 9 members, PRR[d]	3	Up-regulated (+++/+)
TVAG_158720	724	BspA-like, TMD	SF #268, 3 members	5	Up-regulated (+++/-)
TVAG_301290	733	BspA-like, TMD	Singleton #827, divergent	1	Up-regulated (+/-)
TVAG_268070	788	BspA-like, TMD	Singleton #477	6	Up-regulated (++/-)
TVAG_154640	805	BspA-like, TMD	SF #13, 9 members, PRR[d]	2	None (+/+)
TVAG_355160	923	BspA-like, TMD	SF #31, 13 members	0	None (++/++)
TVAG_139560	950	BspA-like, TMD	Singleton #769, divergent	1	None (+/+)
TVAG_158740	1047	BspA-like, TMD	SF #228, 3 members	3	Up-regulated (++/-)

[a]See additional file 1, Table S1 for full details. TMD, transmembrane domain, all listed proteins have one inferred TMD.
[b]SF, subfamily (#number) identified from analysing all 911 TvBspA with CLUSS2. PRR, proline-rich repeat. For singletons the number in brakes indicate their position in the Clustal alignment, the higher the number the more divergent the sequence relative to other aligned TvBspA sequence and two entries are considered to possess divergent TpLRR being located towards the bottom part of the alignment.
[c]The signs between brackets indicate presence(+) or absence (-) of PCR products on bound/unbound cells - see Figure 5.
[d]Same subfamily #13, see additional file 1, Table S1.
