# Supplementary material for: Trichomonas vaginalis vast BspA-like gene family: evidence for functional diversity from structural organisation and transcriptomics
Source: BMC Genomics. 2010 Feb 8;11:99. doi: 10.1186/1471-2164-11-99 (PMC2843621; doi:10.1186/1471-2164-11-99)
Supplement: Additional file 17 — Supplemental Figure S6. Western blot analyses with mouse anti-TvBspA625 peptide antisera on T. vaginalis total proteins extracts. [file 1471-2164-11-99-S17.PDF]

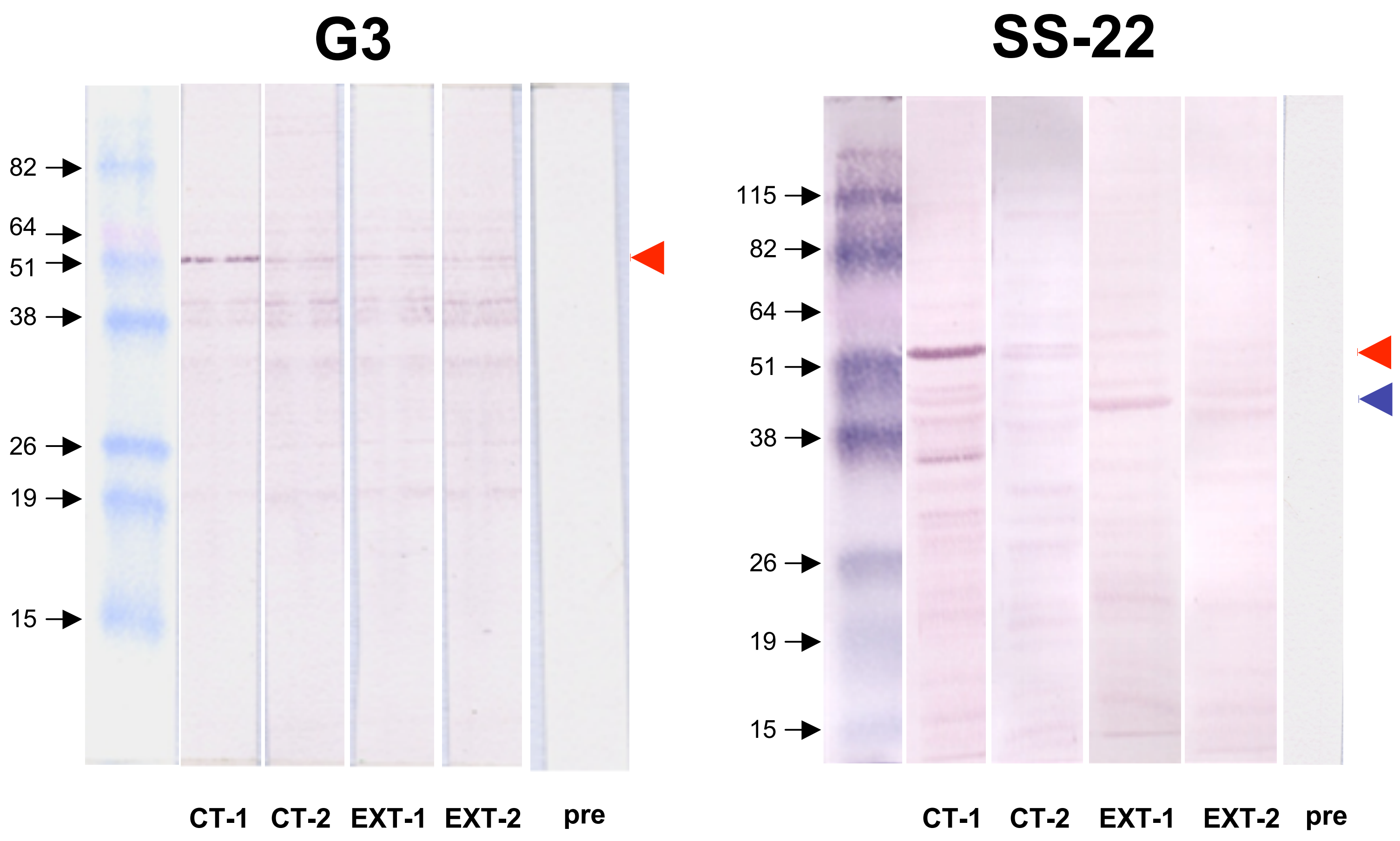

Figure S6. Western blot analyses of TvBspA625 expression in two *Trichomonas vaginalis* clinical isolates.
Total protein extracts from *T. vaginalis* isolates G3 (left) and SS-22 (right) grown *in vitro* were subjected to SDS-PAGE and transferred onto membrane for Western blot analyses. Primary mouse antisera directed against the TvBspA625 peptides derived from the cytosolic tail (CT-1 or CT-2) and the extracellular domain (EXT-1 or EXT-2) (Figure 2) were used and these were detected with secondary anti-mouse antibodies coupled with alkaline phosphatase. Antigen-antibody complexed were identified with a chromogenic substrate. In both isolates a major band with an apparent size of 52 kDa was detected with the anti-CT-1 antisera (red arrowhead). In isolate G3 a faint band with the same apparent size can be seen with the other three antisera, whereas in isolate SS-22 only the anti-CT-2 antisera identified two major band at ~52 kDa. In contrast the anti-EXT-1 antisera identified a major band at ~40 kDa in isolate SS-22 (blue arrowhead). Absence of any signal for all pre-sera **(pre)** suggest that the identified banding pattern is specific to the TvBspA625 protein.
