# Supplementary material for: Trichomonas vaginalis vast BspA-like gene family: evidence for functional diversity from structural organisation and transcriptomics
Source: BMC Genomics. 2010 Feb 8;11:99. doi: 10.1186/1471-2164-11-99 (PMC2843621; doi:10.1186/1471-2164-11-99)
Supplement: Additional file 18 — Supplemental Figure S7. Indirect immunofluorescence analyses with anti-TvBspA625 peptide antisera on T. vaginalis fixed with formaldehyde. [file 1471-2164-11-99-S18.PDF]

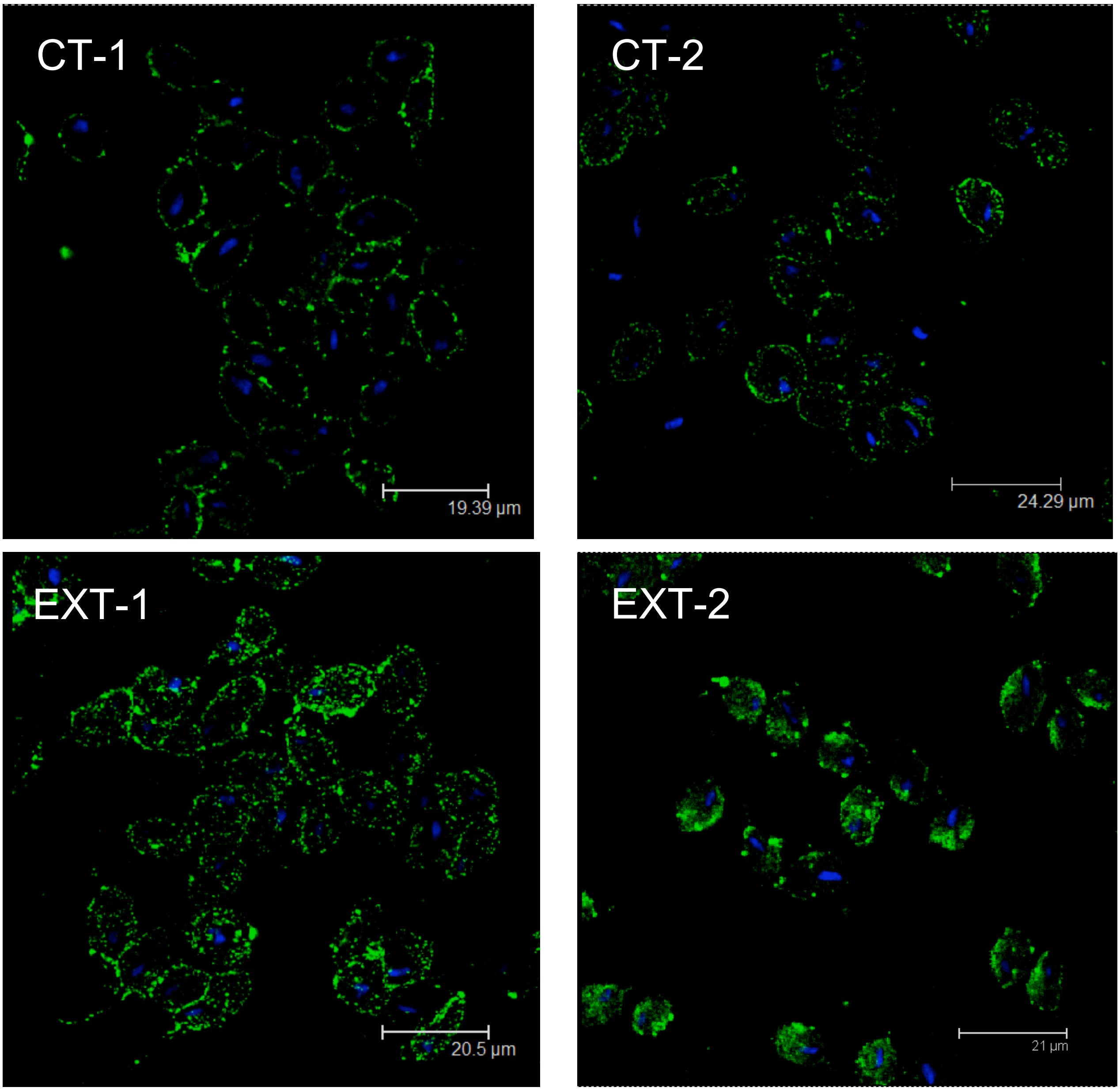

Figure S7. TvBspA625 cellular localisation by indirect immunofluorescence analysis .

T. vaginalis in vitro cultures (G3 isolate) were processed for IFA (formaldehyde fixation) with the indicated mouse antisera and revealed with a goat anti-mouse IgG Alexa488 conjugated antisera (green labelling). DAPI (blue labelling of nuclei) was added to the mounting solution. A confocal section maximising the size of the cells circonferance was taken for each antisera. The top panels correspond to the two antisera raised against the two cytosolic derived peptides (CT-1 and CT-2, Figure 2C) and the bottom two panels to the antisera raised against the extracelluar domain derived peptides (EXT-1 and EXT-2, Figure 2C). Back ground was reduced by substracting signal present in the field suronding the cells. Scale bares are indicated in each panel.
