# Supplementary material for: Trichomonas vaginalis vast BspA-like gene family: evidence for functional diversity from structural organisation and transcriptomics
Source: BMC Genomics. 2010 Feb 8;11:99. doi: 10.1186/1471-2164-11-99 (PMC2843621; doi:10.1186/1471-2164-11-99)
Supplement: Additional file 19 — Supplemental Table S12. List of primers used for RT-PCR and qRT-PCR. [file 1471-2164-11-99-S19.PDF]

Table S12. Primers used for RT-PCR and qRT-PCR.

TvBspA-like and control primers			
**Gene name**	**Primers name***	**Sequence 5′-3′**	**Amplicon Size**
TvBspA605 TVAG_244930	1266F	CATTGTGCTTCCACCTTATC	513
	1778R	CCAAGGTAATATTCCAACCC	
TvBspA625 TVAG_073760	1026F	ACCAACATTGACTCATTTCACC	760
	1785R	GGTGCCATTTGCTATCGTATCT	
TvBspA724 TVAG_158720	1632F	CTCTCTTCGCTTCACAACAG	451
	2082R	CCAGCATATTCAAAGACTGC	
TvBspA733 TVAG_301290	1654F	ATTGGAGTCAAATATTTGGG	352
	2005R	CGCTTTAGTGAACTTACACC	
TvBspA788 TVAG_268070	1773F	CCACAACACAGCTCTTACAC	537
	2309R	TGACCATGCAGCACTTAACG	
TvBspA805 TVAG_154640	1578F	GATTTACGAAACTACTGGATTCAG	795
	2372R	TGTTGCCAAAACATTTTCATTAGT	
TvBspA923 TVAG_355160	2135F	CAAATTGCTCAATAAAAGGAGATG	548
	2682R	AATTGTGATACCTATAGAACTAAG	
TvBspA950 TVAG_139560	1573F	TCCGATAAGTTTTCTTTCCCATC	1052
	2624R	GTTACTGATGAATTCGATACATG	
TvBspA1047 TVAG_158740	2502F	GAGTCTTGTCTGTCTTTTCGG	583
	3084R	GAAGATTCCGAAGAAATGAGG	
TvBspA1498 TVAG_397210	869F	CTTACATAGAAGCGAACACT	158
	1027R	CGAAGGAAGCATTATATATT	
TvBspA1209 TVAG_441420	805F	GTTATCTGCAGTTACTCGTT	66
	871R	GGTATCTGGAAGAATACTGA	
TvBspA2174 TVAG_530030	799F	ACATCACATAACGATCACTT	129
	928R	AATGATCGTACACTGTGAAT	
Malic enzyme B TVAG_238830	20F	GCTTACATCTTCAGTCAACT	95
	115R	GATCACCATCTTGAAGAAGT	
Cytosolic malate dhase TVAG_165030	115F	CTCTATTCAAGAACTCAAGA	149
	264R	TTGTATGAGCTTGTAAGGAA	
Actin+	Tvact1	TGTCGGCCGTCCAAAGTA	450
	Tvact2	ATCACGGCCAGCGAGGTTA	
Alpha-actinin+	TV44	TCGCTTCCGTTATCT	513
	TV45	AGGAGGTGCTTGATGT	

* For the TvBspA-like genes specific primers the number of the forward (F) primer corresponds to the most 5’end nucleotide or for the reverse (R) primers the number corresponds to the most 3’end nucleotide, respectively, on the given ORF.

+The actin and alpha actinin primers were described in Addis *et al*. [90]
